# Indolequinone-Based Hypoxia-Activated Proteolysis Targeting Chimeras Selectively Degrade BRD4 in Hypoxic Cancer Cells

**DOI:** 10.1021/jacs.5c10240

**Published:** 2025-09-25

**Authors:** Marta Serafini, Sophie A. Twigger, George Delfas, Maxim Mallerman, Elliot P. Bailey, Ewen D. D. Calder, Ester M. Hammond, Stuart J. Conway

**Affiliations:** † Department of Chemistry, Chemistry Research Laboratory, 6396 University of Oxford, Mansfield Road, Oxford OX1 3TA, U.K.; ‡ Department of Oncology, University of Oxford, Old Road Campus Research Building, Oxford OX3 7DQ, U.K.; § Department of Chemistry & Biochemistry, 8783 University of California Los Angeles, 607 Charles E. Young Drive East, Los Angeles, California 90095, United States; ∥ California NanoSystems Institute, University of California Los Angeles, 570 Westwood Plaza, Building 114, Los Angeles, California 90095, United States; ⊥ Jonsson Comprehensive Cancer Center, University of California Los Angeles, 8-684 Factor Building, Los Angeles, California 90095, United States; # Molecular Biology Institute, University of California Los Angeles, 611 Charles E. Young Drive East, Los Angeles, California 90095, United States

## Abstract

Proteolysis targeting chimeras (PROTACs) have helped to establish proximity induction as an exciting strategy in drug discovery, and there are multiple clinical trials focused on this modality. However, degradation of a full protein in a physiological setting might lead to dose-limiting toxicities, giving rise to the need for PROTACs that are activated in a context-dependent nature. Here, we report the development of hypoxia-activated PROTACs (HAP-TACs) which are selectively activated in conditions of low oxygen (hypoxia) such as those found in solid tumors. To develop HAP-TACs, we have attached an indolequinone bioreductive group to an essential functional group of either the VHL- or cereblon-recruiting component of the PROTAC, reducing affinity for its cognate E3 ligase and preventing degradation of the protein of interest. Using BRD4, we have conducted proof-of-concept studies which demonstrate that the indolequinone group is bioreduced under hypoxic conditions, releasing the active PROTAC, resulting in selective degradation of BRD4 in hypoxia. As the bioreductive group is attached to the VHL or cereblon ligand, this approach is potentially applicable to all PROTACs that recruit these commonly employed E3 ligases.

## Graphical abstract

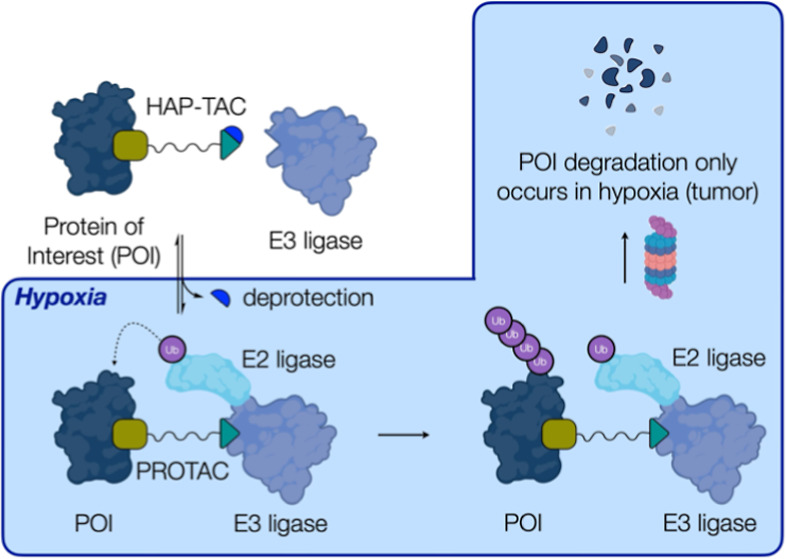

## Introduction

Proteolysis targeting chimeras (PROTACs) are heterobifunctional molecules comprising a ligand for a protein of interest (POI) attached to a ligand for an E3 ligase by a linker. They function by inducing proximity between the POI and the E3 ligase leading to polyubiquitination of the POI and subsequent proteasome-mediated degradation. This mode of action gives PROTACs advantages over traditional occupancy-based drugs, including enhanced selectivity resulting from the need for a productive ternary complex to form, and degradation of whole multidomain proteins with the consequent cellular ablation of both ligandable and unligandable domains.^1–3^ Interest in protein degradation and proximity induction is intense, and currently, over 30 PROTACs have progressed to clinical trials,^1,4,5^ demonstrating the excitement and therapeutic potential in this area.

Despite these successes, there are some potential limitations for this modality, especially in the translation of PROTACs from a preclinical to a clinical setting. One concern is the effects resulting from degradation of a complete protein in both healthy and diseased tissues might result in dose-limiting on-target toxicities.^1^ Additionally, off-target effects or toxicities might result from the E3 ligase ligand, as it can potentially retain some activity on its cognate E3 ligase. For example, it has been shown that cereblon (CRBN)-recruiting PROTACs based on immunomodulatory drugs (IMiDs), including thalidomide and its analogs, can maintain the ability to degrade the CRBN neosubstrates IKZF1 and IKZF3.^6–9^ This off-target activity can result in inhibition of cell proliferation and stimulation of the immune system independently from POI degradation.^10^

To overcome these issues, prodrugs of PROTACs that can be released selectively in the desired context or environment have been developed.^11^ One of the earliest approaches to achieving targeted protein degradation was the development of light-activated PROTACs. Attaching a photocleavable group to the VHL or CRBN E3 ligase ligands allows spatial control of the release of the active degrader.^12–15^ However, this approach has limitations in therapeutic settings resulting from the poor tissue penetration of the UV light required for photorelease of the PROTAC. Yang et al. reported a radiotherapy-triggered VHL-recruiting PROTAC, in which E3 ligase recruiter is unmasked upon X-ray irradiation of a tetrafluoro-phenyl azide group.^16^ However, the radiation doses used were much higher than those typically employed in the treatment of cancer patients. Other strategies have harnessed components of the tumor microenvironment to achieve selective protein degradation, including folate-caged^17,18^ E3 ligase ligands and ROS-responsive PROTACs.^19,20^

Hypoxia-activated prodrugs (HAPs) are an effective strategy to target therapeutic agents to tumors in vivo.^21–23^ Hypoxia is defined as insufficient oxygen, and while physiological levels of oxygen vary between tissues, severe hypoxia is a hallmark of solid tumors. Hypoxia results from the rapid growth of tumor cells, leading to a higher metabolic demand, a disorganized vasculature, and a poor blood supply to the tumor. The hypoxic environment leads to aggressive phenotypes and resistance to both chemo- and radiotherapy. HAPs employ bioresponsive chemistry to take advantage of the reducing hypoxic environment: they are inactive prodrugs in normoxic cells but undergo enzyme-catalyzed reduction in hypoxia, resulting in the release of the active drugs. This strategy has been previously applied to the development of HDAC inhibitors,^23,24^ Chk1 inhibitor,^25^ DNA-PK inhibitor,^26^ and the pan-HER inhibitor tarloxotinib,^27^ which advanced to Phase II clinical trials, to the nitrogen mustard alkylating agent TH032^28^ which reached Phase III clinical trials, and to the DNA alkylating agent CP-506 which is in Phase II clinical trials.^29^

Three hypoxia-activated PROTACs (HAP-TACs) were reported previously. In 2021, Cheng et al. published a pomalidomide-based EGFR-degrading PROTAC in which a nitroaryl group was attached to the POI ligand.^30^ This resulted in POI engagement being observed only in hypoxia, but the CRBN binder remained active in both normoxia and hypoxia. In 2023, the same group disclosed a close analogue of the EGFR-degrading PROTAC with a nitroaryl group attached to the pomalidomide glutarimide nitrogen.^31^ Despite the attachment of the bioreductive group to the E3 ligase ligand rendering this approach more generally applicable, only modest degradation was achieved in both hypoxia and normoxia, which is likely related to the instability of the pomalidomide-based scaffold and low fragmentation following bioreduction (vide infra). Lastly, Shi et al. reported a third HAP-TAC for EGFR degradation in which a 2-nitroimidazole group was employed to protect the essential hydroxyl group of a VHL ligand.^32^ However, the nitroaryl group was attached to the VHL ligand using a carbonate group, which can be unstable and might result in the unwanted release of the active PROTAC in normoxia.

We have recently shown that the indolequinone bioreductive group provides an effective mechanism for releasing fluorescence dyes in a range of hypoxic conditions.^33^ Here, we employed this trigger to develop structurally novel HAP-TACs. The indolequinone group is employed to protect either the VHL- or CRBN-recruiting ligands, rendering them inactive in normoxia. In hypoxia, the indolequinone group is bioreduced, likely by NAD­(P)­H/quinone oxidoreductase (NQO1) or cytochrome P450 oxidoreductase, to efficiently release the active PROTACs, resulting in context-dependent protein degradation (Figure 1).^33^ We have employed both CRBN- and VHL-recruiting PROTACs that promote the degradation of BRD4, a transcriptional regulator of oncogenes, such as c-Myc and BCL-6, which drives tumor progression. Clinical application of BRD4 inhibitors has been hindered by on-target toxicity, and thus, this strategy potentially provides a method for impairing the function of this protein selectively in tumors. In the case of the CRBN-recruiting PROTAC, protection of the E3 ligase ligand also prevents unwanted degradation of CRBN neosubstrates.^6–9^ Attachment of the bioreductive group to the E3 ligase ligands means that this approach can, in principle, be applied to any VHL- or CRBN-recruiting PROTAC.

**Figure 1:**
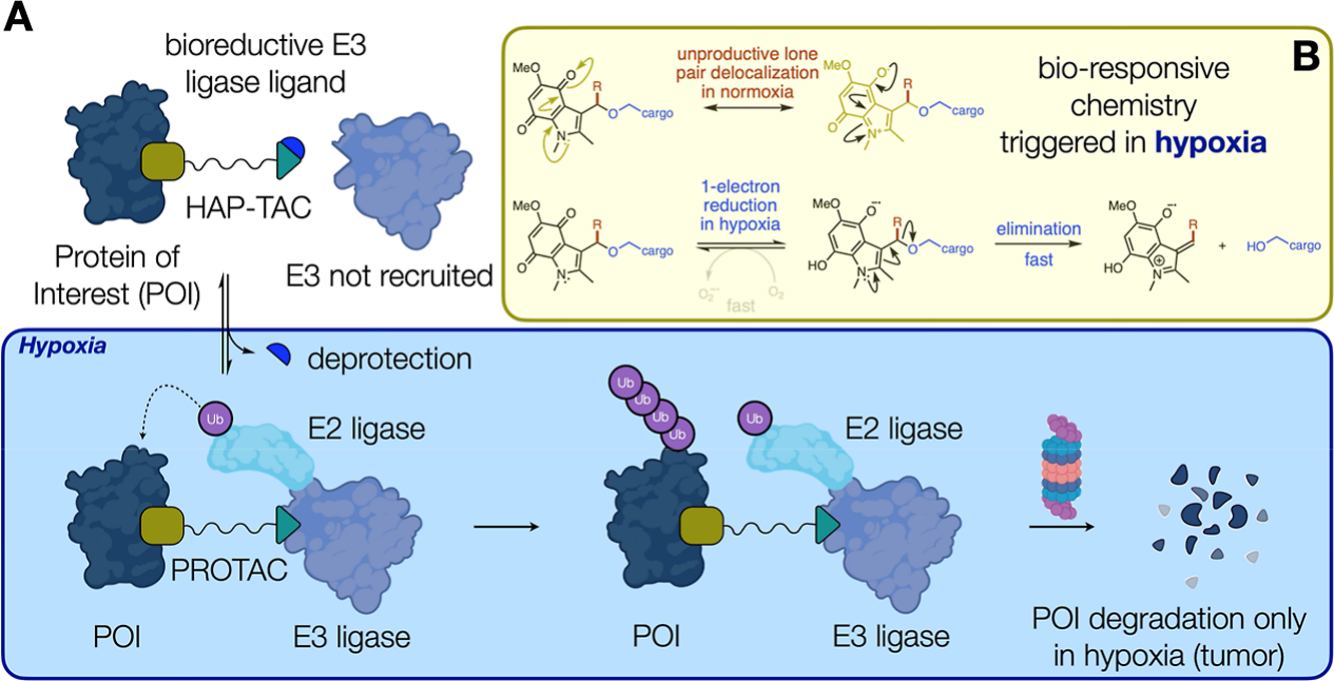
(A) A cartoon illustrating the principle of hypoxia-activated PROTACs (HAP-TACs). Created using BioRender.com. (B) The mechanism by which the indolequinone group functions as a bioreductive group.

## Results and Discussion

The initial HAP-TACs we developed were based on MZ1 ^34^ and dBet1,^35^ two BRD4-degrading PROTACs that recruit the E3 ligases VHL and CRBN, respectively. The development of each series of HAP-TACs is described below.

### Design of VHL-Recruiting HAP-TACs

The first step in the development of an HAP is the identification of a functional group to which the bioreductive moiety can be attached, which will render the compound biologically inactive. The key interactions required for a ligand to bind to VHL are well established (Figure 2A). Previous work has shown that mimics of the hydroxyproline residue, found in HIF1α and VHL ligands (e.g., VH032 Figure 2A),^36^ are essential for high affinity binding to VHL. Inversion of the stereochemistry at this position, or substitution of the hydroxyl group, prevents binding to VHL. Therefore, this functionality is ideal for the addition of a bioreductive group to produce an inactive prodrug. Bioreduction and fragmentation of the group attached at this position will reveal the active PROTAC. This position has been derivatized in other PROTAC prodrugs, providing confidence in this approach.^13,14,16,18,20,32^

**Figure 2:**
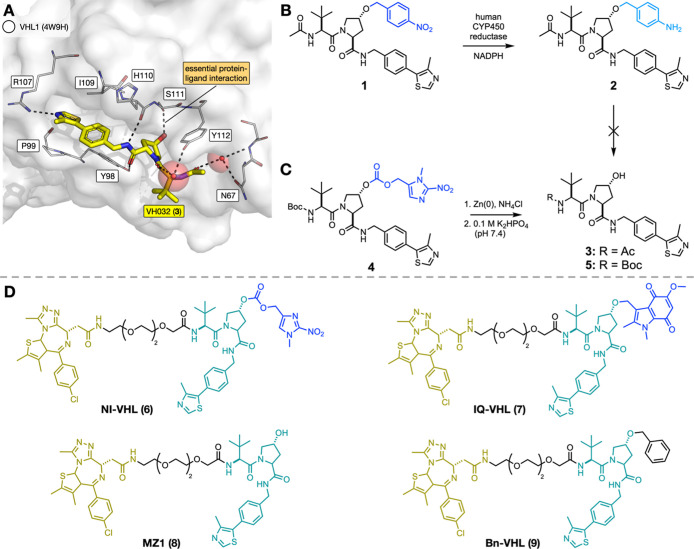
(A) The X-ray crystal structure of VH032 (**3**) bound to the pVHL–ElonginB–ElonginC complex 1 (PDB ID: 4W9H), showing the essential hydrogen bond between the hydroxyproline group and S111 of VHL1.^36^ (B) Investigation of human CYP450 reductase-catalyzed reduction of a 4-nitrobenzyl group attached to VH032 (compound **1**). While reduction of the nitro group to give aniline **2** was observed, fragmentation to give the desired product **3** did not occur. (C) Reduction of the carbonate-linked 1-methyl-2-nitroimidazole group (compound **4**) with Zn(0) and NH_4_Cl resulted in the production of the VH032 ligand **5**. (D) The structures of the VHL-recruiting HAP-TACs, NI-VHL (**6**) and IQ-VHL (**7**), MZ1 (**8**), and the negative control Bn-VHL (**9**).

We first investigated the addition of a nitroaryl-based bioreductive group to the hydroxyproline mimic of VH032 (**1**, Figure 2B). Treatment of Boc-proline methyl ester with 4-nitrobenzyl bromide and Ag_2_O gave a 99% yield of the alkylated product. Subsequent deprotection and coupling reactions gave the desired final compound **1** (Scheme S3). We next treated the 4-nitrobenzyl-protected VH032 (**1**) with human CYP450 reductase in hypoxia, using our well-established protocol.^37^ While efficient reduction of the nitro group to give aniline **2** was observed, this product did not fragment to give the free VH032 ligand **3**, demonstrating that the 4-nitrobenzyl group was not suitable for use in the development of HAP-TACs (Figure 2B). We next investigated the synthesis of the 1-methyl-2-nitroimidazole group attached to the hydroxyl group of Boc-protected VH032 derivative, which undergoes more facile bioreduction in hypoxia.^37^ However, attempts at direct alkylation of the hydroxyl group with the 2-nitroimidazole proved unsuccessful, and therefore, we instead synthesized the carbonate derivative **4**. The synthesis proceeded readily, giving the carbonate **4** in a 22% yield over two steps (Scheme S4). Treatment of compound **4** with Zn(0) and NH_4_Cl, followed by phosphate buffer, resulted in production of the Boc-protected VH032 derivative (**5**), consistent with reduction and subsequent fragmentation (Figure 2C).^37^ Prompted by this positive result, we synthesized MZ1 (**8**, Scheme S5) and then applied the same moiety to the hydroxyl group of the MZ1 VHL ligand to give compound **6** (NI-VHL) (Figure 2D and Scheme S5).

While carbonate groups have been used in prodrug design to give an improved PK profile, assist oral delivery, and improve release of the active drug (e.g., tenofovir disoproxil),^38^ it can also result in instability in a biological setting, meaning this was not an optimum approach.

We have recently employed the indolequinone group in bioreductive dyes that imaged gradients of hypoxia^33^ and so decided to investigate whether this group could also be used to produce hypoxia-activated PROTACs. We obtained compound **7**, with the indolequinone directly attached to the VHL ligand (IQ-VHL, Scheme S5) in 32% yield (Figure 2D). To provide a negative control for the VHL-recruiting HAP-TACs, a PROTAC alkylated with an inactive benzyl group at the same position was also synthesized (**9**, Bn-VHL, Figure 2D).

### Design of CRBN-Recruiting HAP-TACs

CRBN ligands often contain a glutarimide ring which forms interactions with the backbone and side chain of CRBN H379 (Figure 3A). *N*-Methylation of this ring prevents the formation of a key hydrogen bond, reducing the affinity of the ligand for CRBN,^39^ making this a suitable position to attach the bioreductive group. Previous work on kinase inhibitors has shown us that attaching a bioreductive group to a nitrogen atom, rather than an oxygen atom, can lead to inefficient release of the active compound.^37^ Therefore, we expected the development of a hypoxia-activated CRBN ligand to be challenging. To investigate a suitable bioreductive group for use with CRBN-recruiting PROTAC, we first attached the 4-nitrobenzyl or the 1-methyl-2-nitroimidazole group to the glutarimide nitrogen atom of pomalidomide (**11**, Figure 3B). Treatment of the 4-nitrobenzyl derivative (**10**) with Zn(0) and NH_4_Cl, followed by phosphate buffer, did not produce any free pomalidomide (**11**). However, treatment of the 1-methyl-2-nitroimidazole derivative (**12**) under the same conditions produced a number of products, including some free pomalidomide (based on HPLC analysis, Figure 3C). We next sought to validate compound **12** in an enzyme-based assay using an NADPH–CYP bactosomal reductase (92 pmol/mL) in hypoxia. We have previously shown that prodrug release in this assay corresponds well with prodrug release in hypoxic mammalian cells.^23–25,33,37,40–42^ In the presence of human CYP450 reductase, the 1-methyl-2-nitroimidazole derivative (**12**) gave two new products, neither of which were pomalidomide (**11**, Figure 3D). A plausible explanation is that these compounds are the two products of glutarimide ring opening, with the 1-methyl-2-nitroimidazole group still attached to the nitrogen atom. These data are consistent with a previous report that investigated the use of ROS-activated PROTACs, where direct attachment of the ROS-activated moiety to the glutarimide nitrogen resulted in ring opening.^19^ It is also well established that IMiD-based PROTACs, including dBet1, often display most effective protein degradation after 6–8 h incubation, but typically protein levels recover after 24 h.^35^ This phenomenon likely results from the metabolism of the glutarimide ring.^43,44^

**Figure 3:**
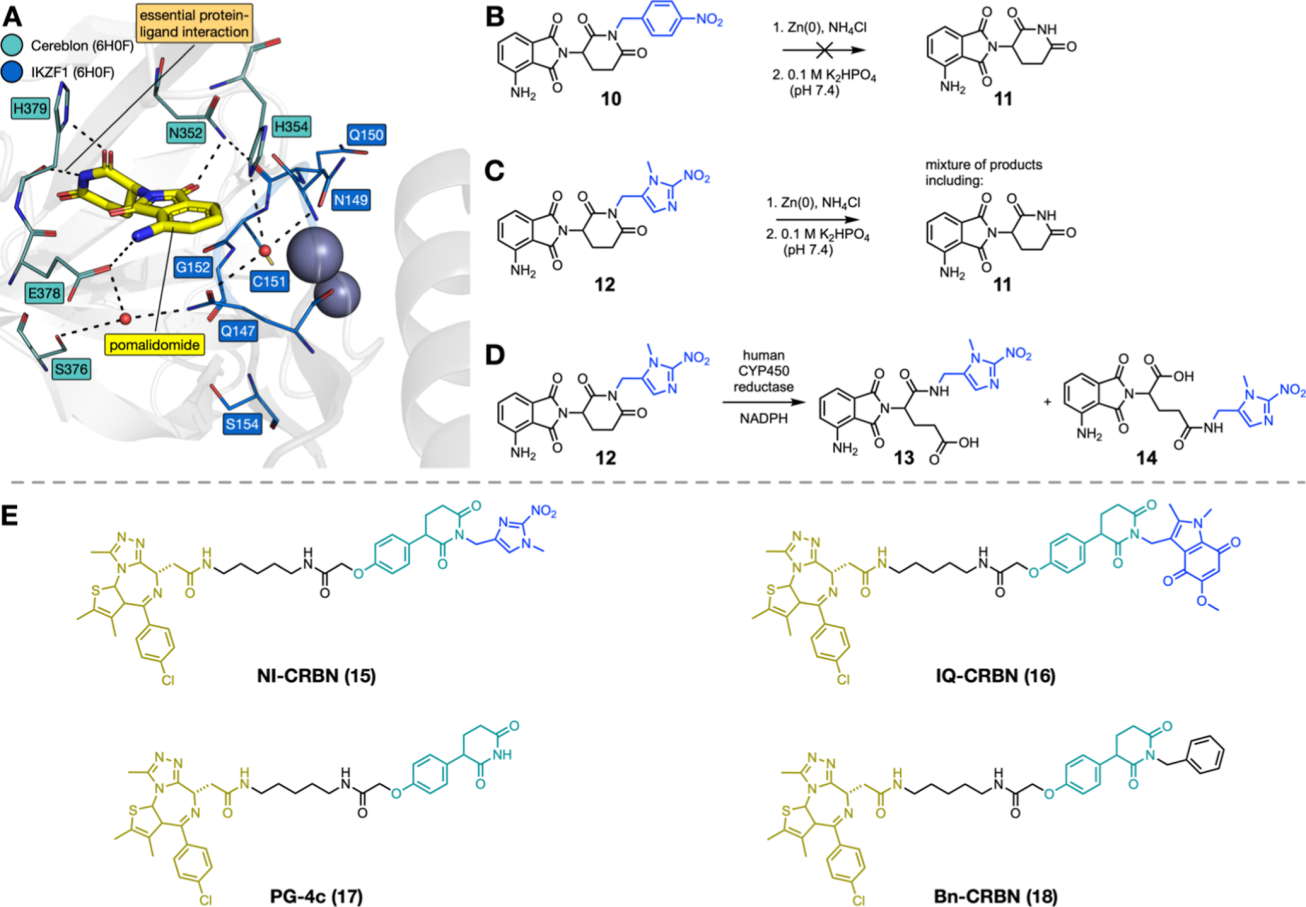
(A) The X-ray crystal structure of pomalidomide bound to cereblon and the second zinc finger of IKZF1 (PDB ID: 6H0F), showing the essential interaction between the glutarimide ring of pomalidomide, the backbone carbonyl group, and side chain of cereblon H379. (B) No reduction or fragmentation of 4-nitrobenzyl group-protected pomalidomide **10** was observed upon treatment with Zn(0) and NH_4_Cl, followed by phosphate buffer at pH 7.4. (C) Treatment of 1-methyl-2-nitroimidazole-protected pomalidomide **12** with Zn(0) and NH_4_Cl, followed by phosphate buffer at pH 7.4 resulted in a complex mixture of products that included pomalidomide. (D) Treatment of 1-methyl-2-nitroimidazole-protected pomalidomide **12** with human CYP450 reductase and NADPH resulted in the formation of two compounds, which we propose are the products of glutarimide ring opening, with the 1-methyl-2-nitroimidazole group still attached to the nitrogen atom. (E) The structures of the CRBN-recruiting HAP-TACs, NI-CRBN (**15**) and IQ-CRBN (**16**), PG-4c (**17**), and the negative control Bn-CRBN (**18**).

To overcome this instability, we investigate the use of phenyl glutarimide-based CRBN ligands (Figure 3E) which were recently reported by Min et al.^43^ as more stable alternatives to IMiD-based compounds. While we initially synthesized the nitroimidazole-based compound **15** (NI-CRBN, Figure 3E), our studies on pomalidomide (vide supra) indicated that the release of the active compound from this bioreductive group was likely to be inefficient. Therefore, we also incorporated the indolequinone group as the alternative bioreductive protecting group for CRBN-recruiting HAP-TACs. As this group lacks the withdrawing effect of the nitroimidazole, it could potentially improve compound stability, in addition to enhancing the efficiency of active PROTAC release. HAP-TAC (**16**, IQ-CRBN, Figure 3E) incorporating this motif was synthesized, along with a benzyl-protected negative control (**18**, Bn-CRBN, Figure 3E). Further information on the synthesis of the VHL- and CRBN-recruiting HAP-TACs can be found in the SI (Schemes S5 and S7).

### Enzyme-Based Bioreduction Assays

With the four HAP-TACs (**6**, **7**, **15**, and **16**) in hand, we next evaluated their hydrolytic stability and their ability to release the active PROTACs in a hypoxia-dependent manner using an enzyme-based assay that we have previously described (Figure 4).^33,37^ Compounds **6**, **7**, **15**, and **16**, the active PROTACs MZ1 (**8**) and PG-4c (**17**), and the two negative controls Bn-VHL (**9**) and Bn-CRBN (**18**) were incubated with NADPH–CYP reductase (CYP004) in hypoxia (<0.1% O_2_) or normoxia (21% O_2_) for 24 h.^37^ Parallel incubations under hypoxia without the reductase enzyme were also performed to assess the hydrolytic stability of the compounds. The residual substrate of the tested compounds and the corresponding release of active PROTACs were monitored by HPLC analysis. We have previously shown that HAPs which are activated under these conditions tend to also be activated in hypoxic cells.^23–25,33,37,40–42^

**Figure 4:**
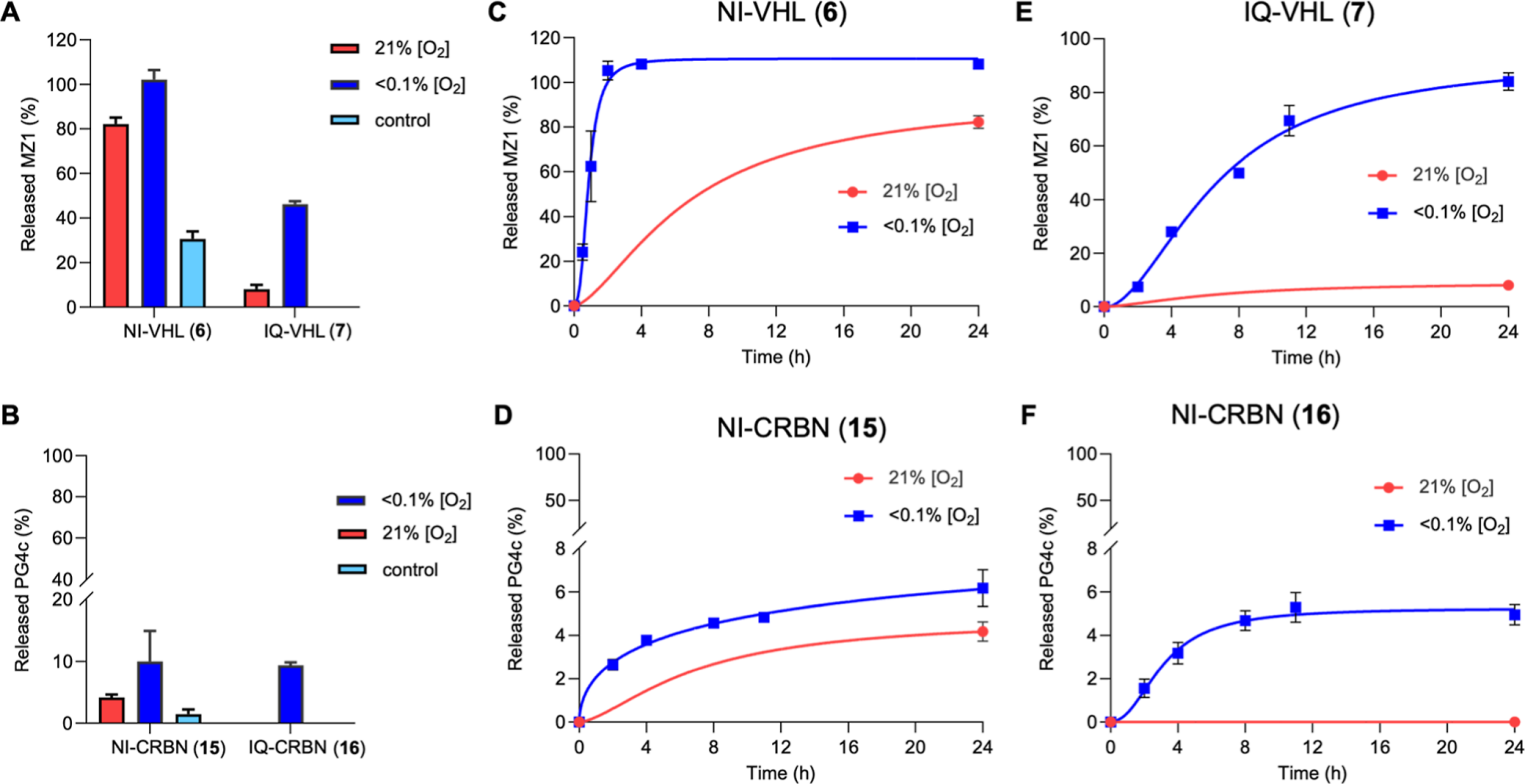
Release of active PROTAC after 24 h of incubation with either NI-VHL **6** (A), IQ-VHL **7** (A), NI-CRBN **15** (B) or IQ-CRBN **16** (B) in normoxia (21% O_2_, red bars) and hypoxia (<0.1% O_2_, blue bars) with NADPH–CYP reductase or in hypoxia in the absence of the enzyme (control, light blue bars). Data are the mean of three independent experiments ± s.e.m. The amount of the active PROTAC released was determined using a calibration curve. Time-course experiment in hypoxia (<0.1% O_2_, blue lines) in the presence of NADPH–CYP reductase for compounds NI-VHL **6** (C), NI-CRBN **15** (D), IQ-VHL **7** (E), or IQ-CRBN **16** (F) versus release of active PROTAC in normoxia (21% O_2_, red lines). Data are the mean of three independent experiments ± s.e.m.

While NI-VHL **6** released 100% of MZ1 after 24 h in hypoxic conditions, it also released 82% of MZ1 in normoxia, meaning that this compound does not show substantial selectivity for the release of MZ1 in hypoxia versus normoxia (Figure 4A). Additionally, 31% of MZ1 was released from NI-VHL in the absence of the enzyme, suggesting that this compound is unstable to hydrolysis in the buffer conditions employed (Figure 4A). In contrast, IQ-VHL **7** was stable in normoxic conditions, releasing only 8% of MZ1, while 41% of MZ1 was released after 24 h in hypoxia (Figure 4A). This compound was also stable in the hypoxic control incubation without an enzyme, where no release of MZ1 was observed (Figure 4A). These data indicate that IQ-VHL **7** will release active PROTAC selectively in hypoxia.

Only a modest release of PG-4c from NI-CRBN (**15**) was observed after 24 h in either normoxic or hypoxic conditions, with 10% and 2% of PG-4c produced, respectively (Figure 4B). Additionally, incubation with the enzyme in either normoxia or hypoxia resulted in multiple peaks observed during HPLC analysis with little PG-4c present after 24 h (Figures 4B and S9). This result is consistent with our previous observations that the electron-withdrawing nature of the nitroimidazole ring likely promotes the hydrolysis of the glutarimide ring. IQ-CRBN (**16**) did not show any release of PG-4c either in normoxia or in the absence of the enzyme. However, selective release of the active PROTAC (9% of PG-4c) was observed in hypoxia (Figure 4B). The active PROTACs MZ1 **8** and PG-4c **17**, and the two negative controls Bn-VHL **9** and Bn-CRBN **18**, were stable after 24 h of incubation in hypoxia, with no substantial differences in stability in the presence or absence of the enzyme (Figures S6C and S11).

To investigate the kinetics of HAP-TAC activation by purified human CYP450 reductase, a time course was performed in hypoxia. This analysis showed that NI-VHL (**6**) released 100% of MZ1 in less than 4 h (Figure 4C), while IQ-VHL (**7**) plateaued at approximately 80% of MZ1 release after 24 h (Figure 4E), with a concomitant reduction of the original prodrug in both cases (Figures S6A, S7, and S8). The amount of PROTACs released from CRBN-recruiting compounds was significantly less over the 24 h (Figure 4D,F), which is consistent with our previous observations that the p*K*_a_ of the HAP cargo determines the rate of reduced prodrug fragmentation to give the active component.^37^ Again, NI-CRBN (**15**) formed multiple products, as observed by HPLC analysis, after 2 h of incubation in hypoxia (Figures S9 and S12C). Conversely, IQ-CRBN (**16**) released 5% of the active PROTAC after 24 h, with no other products observed (Figures 4F and S10). Based on these data, IQ-VHL **7** and IQ-CRBN **16** showed release of the active PROTAC in hypoxia, with little or no release observed in normoxia, indicating a promising selectivity profile for use in cells.

### In-Cell HAP-TAC Evaluation

The four HAP-TACs (**6**, **7**, **15**, and **16**) were next evaluated for their ability to induce BRD4 degradation in A549 cells in hypoxic (<0.1% O_2_) or normoxic (21% O_2_) conditions. Concentrations of the HAP-TACs were chosen based on the results from the enzyme-based assay (Figure 4), and therefore, the CRBN-recruiting PROTACs were used at higher concentrations than the VHL-recruiting compounds to give sufficient PROTAC release.

NI-VHL (**6**) showed little selectivity between the levels of BRD4 degradation in normoxia and hypoxia. At a concentration of 1 μM, 94 ± 3% degradation of BRD4 was induced by NI-VHL in normoxia, and 95 ± 6% of BRD4 was degraded in hypoxia (Figure 5B,D), while at lower concentrations, this compound did not substantially affect BRD4 levels in either set of conditions (data not shown). In contrast, IQ-VHL (**7**) induced little or no degradation of BRD4 in normoxia but almost complete degradation of BRD4 in hypoxia (Figure 5C,E). The highest selectivity for this compound was observed at a concentration of 0.5 μM, where IQ-VHL induced a degradation of 98 ± 2% of BRD4 under hypoxic conditions, but only a modest degradation of 19 ± 24% was observed in normoxia (Figure 5C). A statistically significant difference between BRD4 levels in normoxia and hypoxia was observed (unpaired *t*-test) at all concentrations of IQ-VHL (**7**) used (Figure 5E).

**Figure 5:**
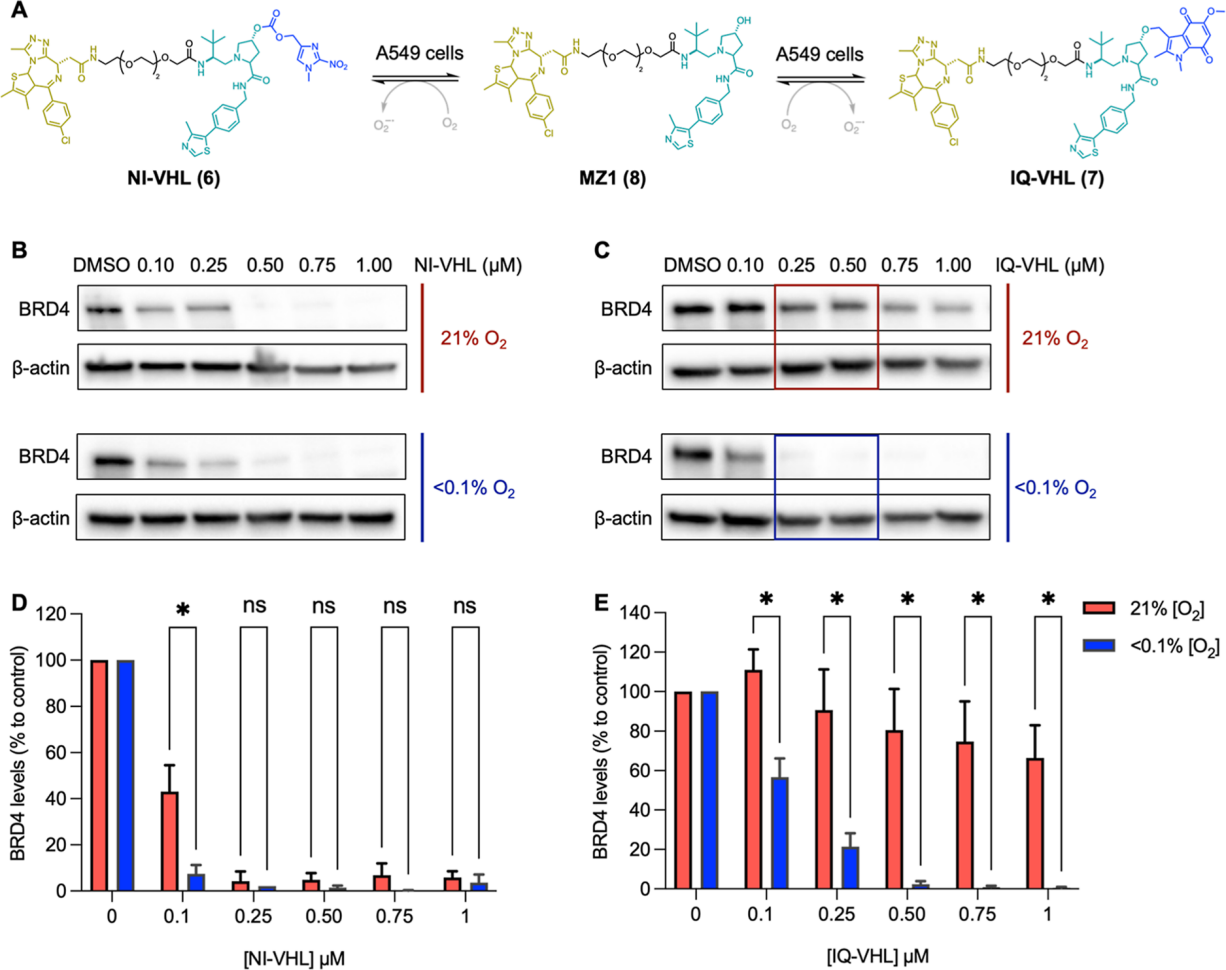
(A) Scheme of HAP-TAC release to give free MZ1. BRD4 levels relative to β-actin loading control and compared to DMSO control, assessed using Western blot analysis, in A549 cells after 24 h incubation with the indicated concentrations of NI-VHL **6** (B) or IQ-VHL **7** (C). Representative Western blots of three independent repeats are shown. β-Actin is used as a loading control. (D) A plot of the quantification of the BRD4 protein levels shown in panel (B). (E) A plot of the quantification of the BRD4 protein levels shown in panel (C). Statistical significance was assessed using multiple unpaired *t* tests. * = *q* < *Q* (indicating a statistically significant difference between normoxic and hypoxic conditions) and ns = *q* ≥ *Q* (indicating no statistically significant difference between normoxic and hypoxic conditions), where *Q* = the false discovery rate and *q* = the false discovery rate adjusted *P*-value. Calculations and graphing conducted using GraphPad Prism 10.5.0.

NI-CRBN (**15**) had no statistically significant effect (unpaired *t*-test) on BRD4 levels in either hypoxia or normoxia (Figure 6B,D), which is consistent with the data from Cheng et al.,^31^ who reported EGFR-targeted HAP-TACs comprising either the nitroimidazole or nitrobenzyl bioreductive group attached to the glutarimide nitrogen atom of a 4-hydroxylthalidomide-based PROTAC. The nitrobenzyl-based HAP-TACs showed modest EGFR degradation in both normoxia and hypoxia. Interestingly, the nitroimidazole-based HAP-TAC showed no EGFR degradation in normoxia but was less effective at degrading EGFR in hypoxia than the nitrobenzyl-based HAP-TAC. While the nitroimidazole group is more effectively bioreduced in cells than the nitrobenzyl group,^37^ this result is likely explained by the nitroimidazole group promoting the hydrolysis of the glutarimide ring. These data and our work indicate that attachment of a nitroimidazole group to a CRBN-recruiting PROTAC is an ineffective strategy for developing HAP-TACs.

**Figure 6:**
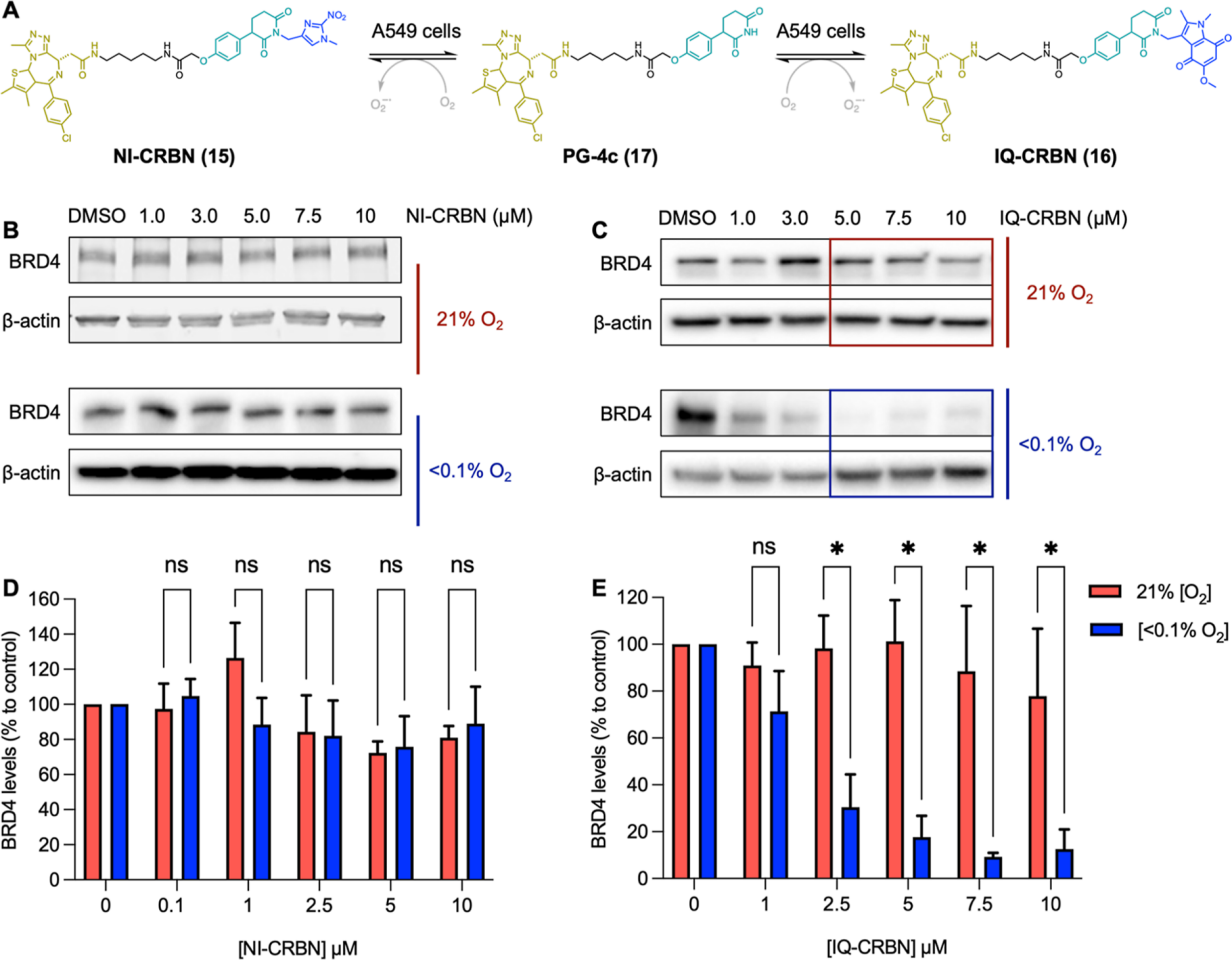
(A) Scheme of HAP-TAC release to give free PG-4c. BRD4 levels relative to β-actin loading control and compared to DMSO control, assessed using Western blot analysis, in A549 cells after 24 h incubation with the indicated concentrations of NI-CRBN **15** (B) or IQ-CRBN **16** (C). Representative Western blots of three independent repeats are shown. β-Actin is used as a loading control. (D) A plot of the quantification of the BRD4 protein levels shown in panel (B). (E) A plot of the quantification of the BRD4 protein levels shown in panel (C). Statistical significance was assessed using multiple unpaired *t* tests. * = *q* < *Q* (indicating a statistically significant difference between normoxic and hypoxic conditions) and ns = *q* ≥ *Q* (indicating no statistically significant difference between normoxic and hypoxic conditions), where *Q* = the false discovery rate and *q* = the false discovery rate adjusted *P*-value. Calculations and graphing conducted using GraphPad Prism 10.5.0.

In contrast, IQ-CRBN (**16**) showed excellent selectivity for the degradation of BRD4 in hypoxia versus normoxia. At concentrations of 5, 7.5, or 10 μM, IQ-CRBN (**16**) had little effect on BRD4 levels in normoxia while inducing almost complete ablation of BRD4 in hypoxia (Figure 6C). For example, at 5 μM, IQ-CRBN induced a degradation of 82 ± 16% of BRD4 in hypoxic conditions and only 16 ± 2% of BRD4 in normoxia (Figure 6C). A statistically significant difference between BRD4 levels in normoxia and hypoxia was observed (unpaired *t*-test) at all concentrations of IQ-CRBN (**16**) used, apart from 0.1 μM (Figure 6E). The in-cell data (Figures 5 and 6) are consistent with the results from the enzyme assay (Figure 4), with compounds **7** and **16** showing greater hypoxia-selective BRD4 degradation compared to compounds **6** and **15**.

Based on the above results, we selected a concentration of 0.25 μM for IQ-VHL (**7**) and 3 μM for IQ-CRBN (**16**) as the optimum concentration for further HAP-TAC evaluation. To investigate whether the expected mechanism of BRD4 degradation was occurring in hypoxia, compounds **7** and **16** were incubated in A549 cells in the presence of either the proteasome inhibitor carfilzomib or MLN-4629,^45^ an inhibitor of the neddylation that is required for the activation of Cullin-RING E3 ligases. Cotreatment of HAP-TACs with either of the inhibitors for 24 h decreased BRD4 degradation, which is consistent with the expected mechanism of action (Figure 7). Consistent with literature reports, HIF-1α levels were increased after treatment with the two inhibitors, resulting from further stabilization of this protein in hypoxia (Figure 7).^46^

**Figure 7:**
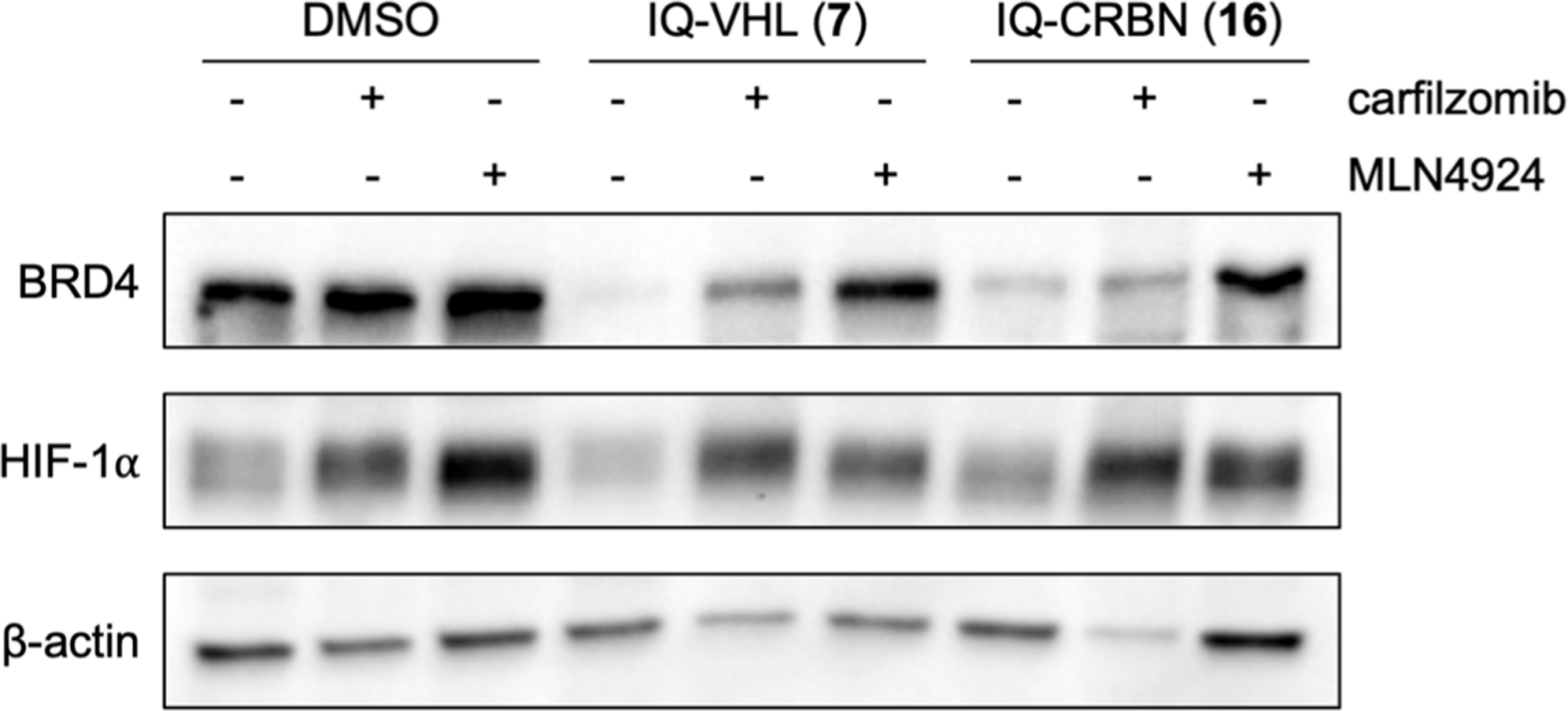
Representative Western blot analysis of three independent experiments in A549 cells after 24 h incubation with IQ-VHL **7** (0.25 μM) and IQ-CRBN **16** (3 μM) with or without the proteasome inhibitor carfilzomib (0.5 μM) or the neddylation inhibitor MLN-4924 (1 μM) in hypoxia (<0.1% O_2_). HIF-1α is used as a hypoxia marker, and β-actin is a loading control.

Based on the above results, we selected a concentration of 0.25 μM for IQ-VHL (**7**) and 3 μM for IQ-CRBN (**16**) as the optimum concentration for further HAP-TAC evaluation (Figure 8). At these concentrations, we evaluated the effect of the negative control compounds Bn-VHL (**9**) and Bn-CRBN (**18**) (Figure 8A) on the BRD4 levels in hypoxic A549 cells. Neither compound undergoes bioreduction to release the active PROTAC, but they do retain the ability to inhibit BRD4. Neither of these compounds induced the degradation of BRD4 in hypoxia (Figure 8B), confirming that the degradation observed in hypoxia is through release of the active PROTAC from the HAP-TAC. Under the same conditions, IQ-VHL (**7**) induced 9 ± 24% BRD4 degradation in normoxia vs 79% ± 8% in hypoxia, while IQ-CRBN (**16**) induced 2 ± 16% BRD4 degradation in normoxia vs 79 ± 6% in hypoxia (Figure 8B).

**Figure 8:**
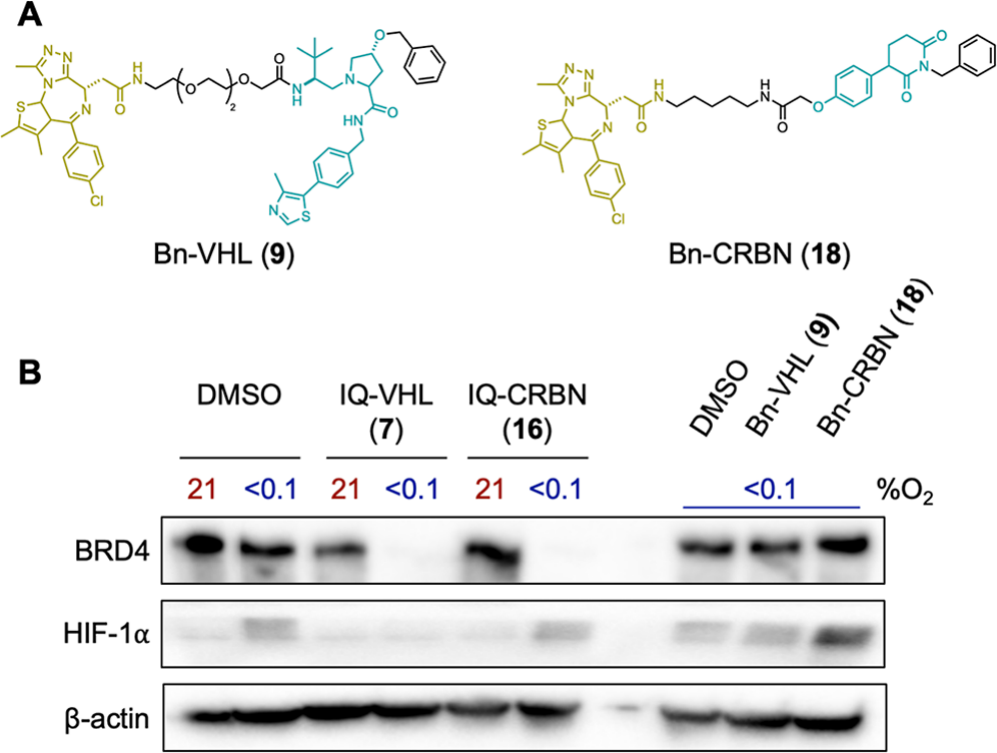
(A) The chemical structures of Bn-VHL (**9**) and Bn-CRBN (**18**). (B) Representative Western blot analysis of A549 cells treated with IQ-VHL **7** (0.25 μM) or IQ-CRBN **16** (3 μM) for 24 h at 21 or <0.1% O_2_, with β-actin used as a loading control. Representative Western blot analysis of A549 cells treated with Bn-VHL **9** (0.25 μM) or Bn-CRBN **18** (3 μM) for 24 h at <0.1% O_2_, with β-actin used as a loading control.

We also treated either A549 or HCT116 cells with a range of (+)-JQ1 concentrations (0.1–1–10 μM) in normoxia. In parallel, the two active PROTACs were tested in A549 cells (Figure S13A) or HCT116 cells (Figure S14A). While MZ1 and PG-4c degraded BRD4 as expected (values ranging from 62 to 100% and 68–91% degradation, respectively), the treatment with (+)-JQ1 alone for 24 h (up to 10 μM) did not reduce BRD4 levels. Interestingly, BRD4 levels were increased in A549 cells after treatment with (+)-JQ1 at all the tested concentrations (174–154% of protein levels after 24 h of treatment, Figure S13), while BRD4 levels were not affected in HCT116 cells.^47^ Given these data, we concluded that the modest degradation exhibited by the compounds in normoxia results from a small amount of active PROTAC release. The catalytic nature of some PROTACs means that even a very small amount of PROTAC release can result in some protein degradation, providing an extra challenge in obtaining hypoxia-selective actions compared to the development of HAPs for occupancy-based drugs.

To determine the level of hypoxia in which IQ-VHL (**7**) or IQ-CRBN (**16**) is activated, we incubated A549 cells for 24 h in 2% O_2_, 0.5% O_2_, or anoxia (≤0.1 O_2_) (Figure 9A). Interestingly, no protein degradation was observed at 2% O_2_, with BRD4 levels observed as 97 ± 8% and 120 ± 25% for **7** and **16**, respectively. At 0.5% O_2_, IQ-VHL induced a 59 ± 6% degradation of BRD4, while IQ-CRBN induced 37 ± 13% BRD4 degradation after 24 h. This result indicates that both HAP-TACs would be released in hypoxic tumors but should not deliver substantial levels of the active PROTAC in tissue that has physiologically lower levels of O_2_, such as bone marrow (1.5–7%), kidney (4–9.5%), the intestine (2–9%), the heart (2–6%), or the retina (<3%).^48^

**Figure 9:**
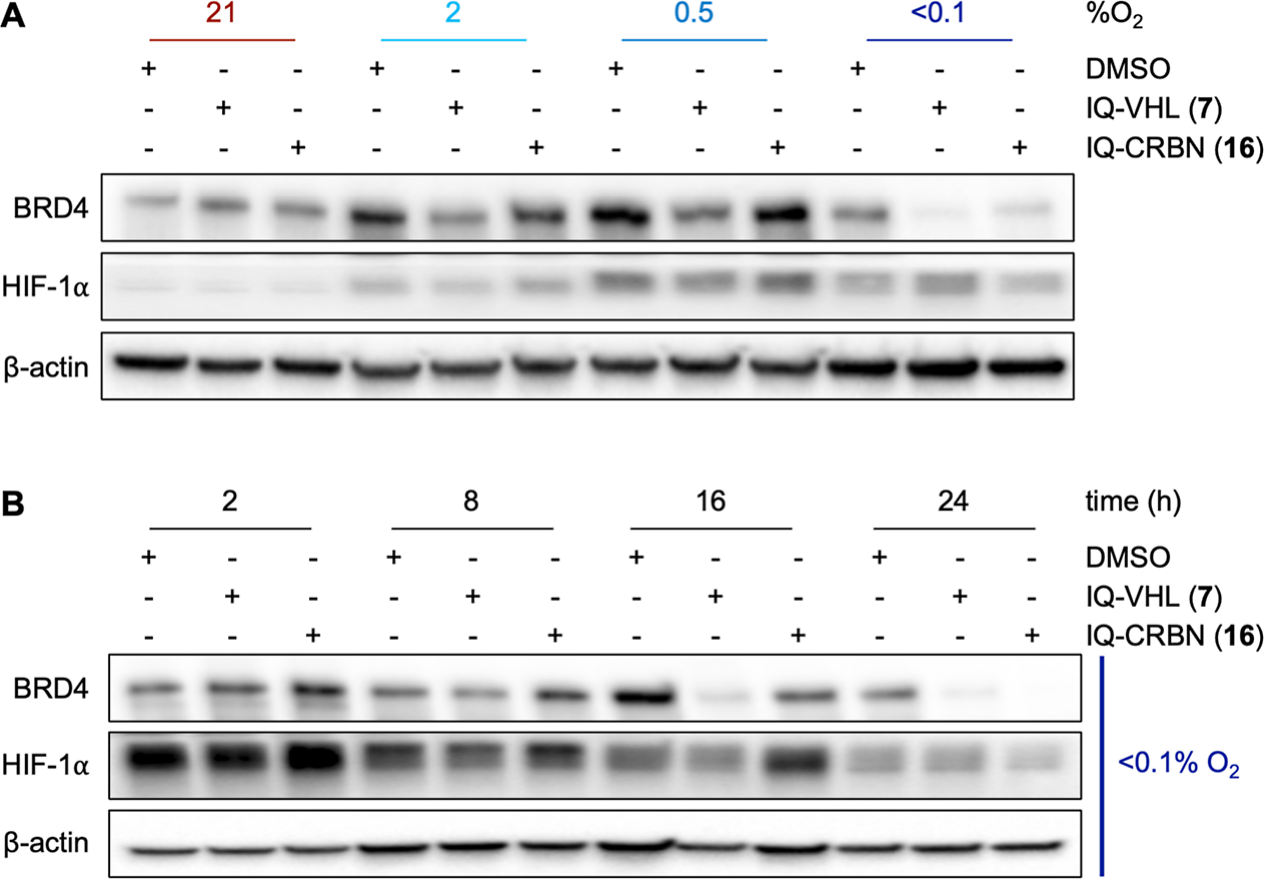
(A) Representative Western blot analysis of BRD4 levels in A549 cells after 24 h incubation with IQ-VHL **7** (0.25 μM) or IQ-CRBN **16** (3 μM) at the indicated levels of O_2_, with HIF-1α used as a hypoxia marker and β-actin used as a loading control. (B) Representative Western blot analysis of BRD4 levels in A549 cells treated in hypoxia (<0.1% O_2_) at the indicated time points, with HIF-1α used as a hypoxia marker and β-actin used as a loading control.

Having selected <0.1% O_2_ as the most effective oxygen concentration for both compounds, we performed a time course in which we evaluated BRD4 levels after 2, 8, 16, or 24 h incubation with HAP-TACs **7** or **16**. After 2 h of incubation with IQ-VHL (**7**), 26 ± 8% was degraded, and 51 ± 3% degradation was achieved after 16 h. Conversely, following treatment with IQ-CRBN **16**, BRD4 levels remained constant until 16 h, when a 37 ± 11% degradation of BRD4 was observed (Figure 9B). These data likely reflect the need for the bioreductive group to be reduced in hypoxia and the reduced product to fragment before the active PROTAC is released.

To demonstrate that the function of HAP-TACs **7** and **16** was not cell-line-dependent, we also investigated their effects in the HCT116 and SF8628 (pHGG) cell lines (Figure 10). HCT116 cells (Figure 10A) were initially treated with MZ1 (**8**), PG-4c (**17**), and (+)-JQ1 at a range of concentrations for 24 h. As expected, the active PROTACs showed high BRD4 degradation (62–100% for MZ1 and 68–92% for PG-4c of BRD4 degraded), while treatment with (+)-JQ1 did not affect BRD4 levels (91–95% of the remaining protein, Figure S14). In normoxic HCT116 cells, IQ-VHL (**7**) induced only a 19 ± 4% degradation of BRD4, with IQ-CRBN (**16**) having the same effect (19 ± 16% degradation of BRD4). In hypoxia, IQ-VHL (**7**) induced a 79 ± 3% degradation of BRD4, and IQ-CRBN (**16**) induced a 72 ± 5% degradation of BRD4, similar to their activity in A549 cells.

**Figure 10:**
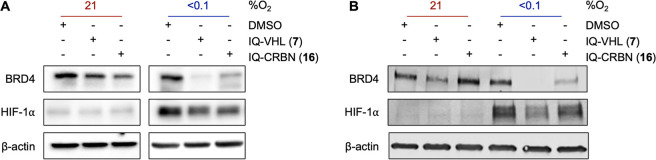
(A) Western blot analysis of BRD4 levels after 24 h incubation in HCT116 (A) and SF8628 (pHGG) cells (B) with IQ-VHL **7** (0.25 μM) or IQ-CRBN **16** (3 μM) in normoxia (21% O_2_) or hypoxia (<0.1% O_2_). HIF-1α is used as a hypoxia biomarker, and β-actin is a loading control.

Compound cytotoxicity in HCT116 cells was determined by using an MTT assay. To differentiate between the inhibitory activity related to the presence of the (+)-JQ1 moiety and the degradation activity related to the release of the active PROTAC in hypoxia, a washout step was performed after 24 h of incubation with the compounds. Previous studies have shown that it takes 20–60 h for BRD4 levels to recover following PROTAC-induced degradation;^49^ consequently, the washout removes compounds from the media, but we expected the levels of BRD4 to remain lower for a prolonged period of time. While the active PROTACs show no significant difference in their cytotoxic profile in normoxia (21% O_2_) and hypoxia (<0.1% O_2_) (Figure S15A,C), both HAP-TACs **7** and **16** show a statistical difference in cell viability in HCT116 cells treated with 1 μM or 10 μM of HAP-TAC, respectively (Figure S15B,D).

Finally, we investigated the antiproliferative effect of compounds **7** and **16** in normoxia or hypoxia. Cell growth over time was determined for HCT116 cells treated with IQ-VHL (**7**) in normoxia (21% O_2_), hypoxia (<0.1% O_2_), or with MZ1 (**8**) in normoxia. The same experiment was performed with IQ-CRBN **16** in normoxia (21% O_2_), hypoxia (<0.1% O_2_), or PG-4c (**17**) in normoxia. Again, a washout step was performed after 24 h of treatment to distinguish between the effects of BRD4 inhibition vs degradation. While IQ-VHL **7** did not substantially affect cell growth in normoxia, the cell number was significantly reduced over time in hypoxia, with a comparable trend in antiproliferative activity induced by MZ1 **8** treatment (Figure 11A). Treatment with IQ-CRBN (**16**) in normoxia affected cell growth, an effect likely related to the inhibition of BRD4; (+)-JQ1 showed a marked cytotoxicity at 1 μM under the same assay conditions (Figure S17B). Despite this, cell proliferation was significantly lower in hypoxia, similar to the treatment with PG-4c **17** in normoxia (Figure 11B). After 6 days, the cell growth had recovered to the same levels as control for the HAP-TACs in normoxia. Cell levels for the samples treated with the HAP-TACs in hypoxia or the active PROTACs remained low even after 6 days. These data indicate that HAP-TACs do provide an advantageous difference in effect between hypoxia and normoxia, even when the POI ligand retains some biological effects. The difference in effects between IQ-VHL (**7**) and IQ-CRBN (**16**) in normoxia likely reflects the difference in cell permeability between the more polar VHL-recruiting HAP-TAC and the lipophilic CRBN-recruiting HAP-TAC. IQ-CRBN (**16**) has a slightly higher cLogD than IQ-VHL (**7**) (2.93 vs 3.00) but also a substantially lower topological polar surface area (TPSA, 196 Å^2^ vs 249 Å^2^), indicating that IQ-CRBN (**16**) is more cell-permeant than IQ-VHL (**7**) (cLogD and TPSA calculated using Chemicalize). Curves displaying the absolute number of cells grown over time can be found in the Supporting Information (Figure S16).

**Figure 11:**
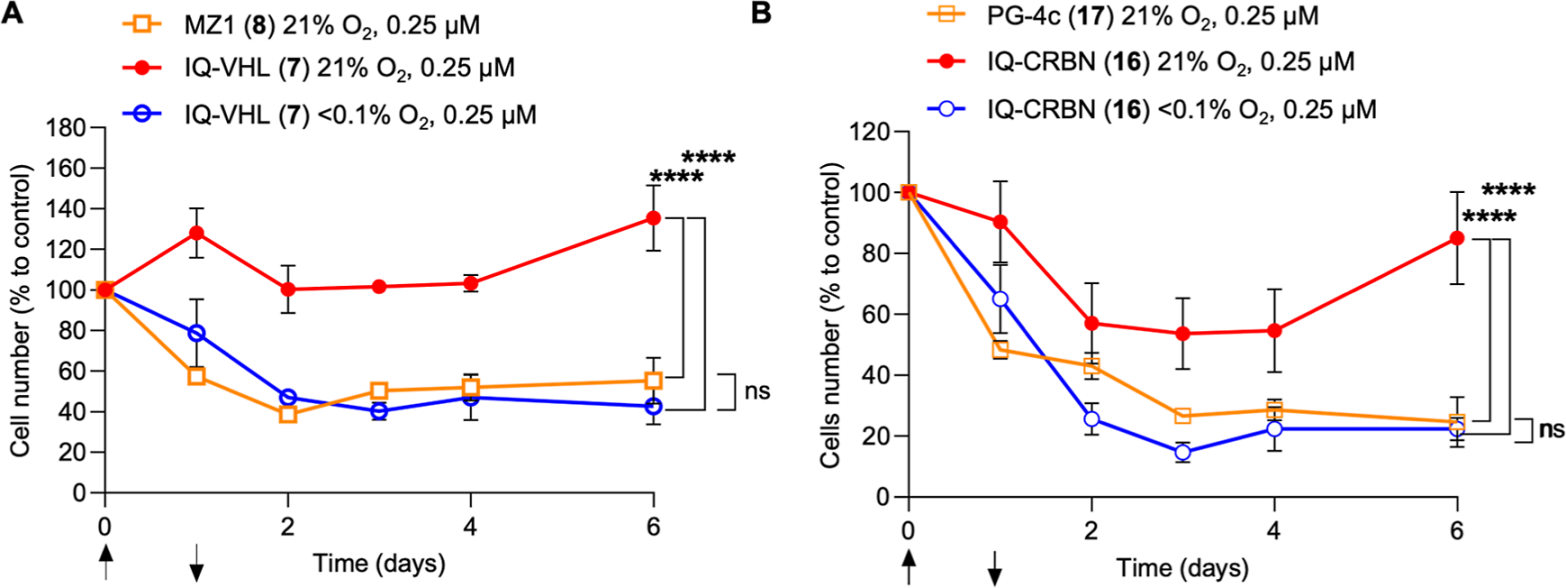
(A) Growth over time of HCT116 cells after 24 h of incubation with MZ1 **8** (0.25 μM) in normoxia (orange line) or IQ-VHL **7** (0.25 μM) in normoxia (red line) or hypoxia (blue line) over 6 days. Cell number is expressed as a percentage related to DMSO control, respectively, in normoxia or hypoxia. Data are the mean of three independent experiments ± s.e.m. (B) Growth over time of HCT116 cells after 24 h of incubation with PG-4c **17** (3 μM) in normoxia (orange line) or IQ-CRBN **16** (3 μM) in normoxia (red line) or hypoxia (blue line) over 6 days. Cell number is expressed as percentage related to DMSO control, respectively, in normoxia or hypoxia. Data are the mean of three independent experiments ± s.e.m.

To further confirm that the antiproliferative effect was related to the release of active PROTACs in hypoxia and the subsequent degradation of the target protein, BRD4 levels were monitored over 4 days by Western blot analysis. While the levels of BRD4 were constant over time in the normoxic cells (Figure 12A), BRD4 levels were maintained at a lower level in the cells that were exposed to hypoxia (Figure 12B), indicating that the observed antiproliferative effects resulted from BRD4 degradation rather than inhibition.

**Figure 12:**
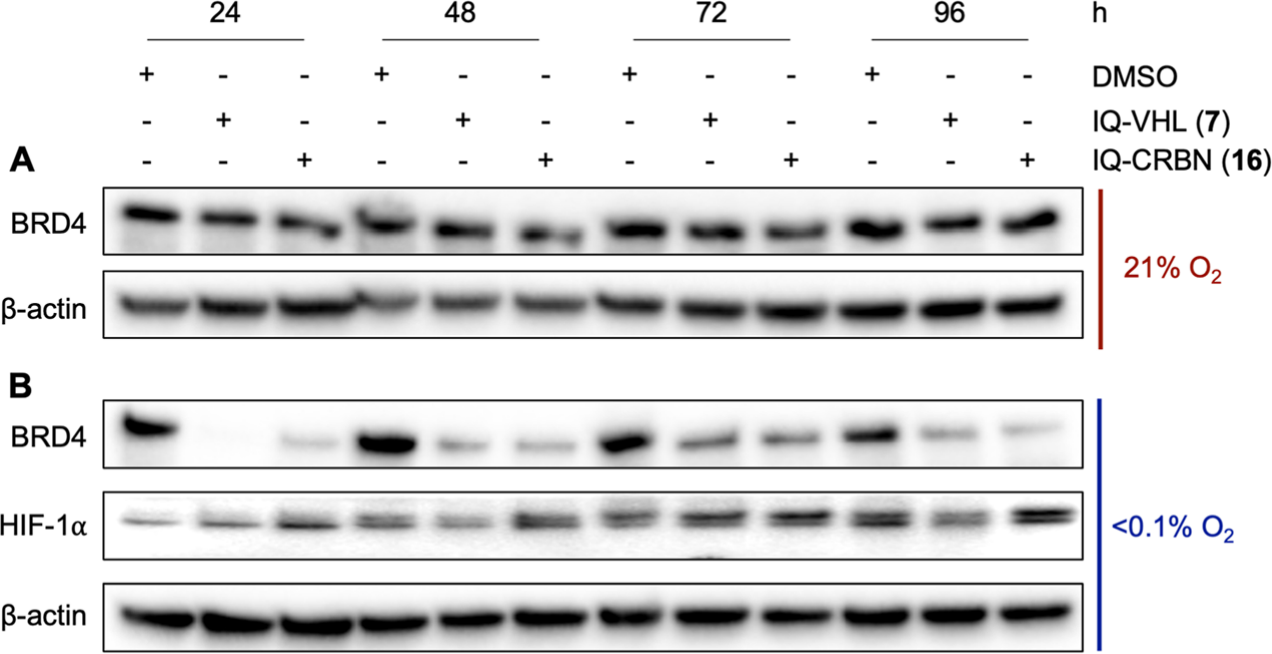
(A) Representative Western blot analysis of three independent experiments in HCT116 cells after 24 h incubation with IQ-VHL **7** (0.25 μM) and IQ-CRBN **16** (3 μM) for 24 h in normoxia, followed by a washout step. (B) Representative Western blot analysis of three independent experiments in HCT116 cells after 24 h incubation with IQ-VHL **7** (0.25 μM) and IQ-CRBN **16** (3 μM) for 24 h in hypoxia (<0.1% O_2_), followed by a washout step. Cells were lysed at the indicated time points. HIF-1α is used as a hypoxia marker, and β-actin is a loading control.

## Conclusion

Strategies to target the protein degradation activity of PROTACs to diseased tissue while sparing healthy tissue will help to maximize the therapeutic window for this drug modality. This approach will likely increase the number of POIs that can be targeted clinically by reducing the risk of on-target toxicity. While a number of strategies have been applied to this end,^12–20^ HAPs have been proven to target the release of a cargo drug to hypoxic tumors, in vivo, in a therapeutically relevant manner.^23,28,29^ Although there have been previous reports of strategies to selectively activate PROTACs in hypoxia,^30,32^ some of the compounds reported contain unstable carbonate-based linkers resulting in a small window of hypoxia/normoxia selectivity. Alternatively, compounds show very inefficient release of the active PROTAC,^31^ likely as a result of cellular instability conveyed by incorporation of the bioreductive group. By employing the indolequinone bioreductive group, we have overcome these issues to develop two HAP-TACs that degrade the proof-of-concept target BRD4 selectively in hypoxia, via recruitment of either CRBN or VHL. When applied at an appropriate concentration, these HAP-TACs show little effect on BRD4 levels in normoxia but exhibit almost complete degradation of this protein in hypoxia. The next step in this work is to evaluate HAP-TACs in vivo, and the indolequinone group is promising in this context for a number of reasons. The HAP-TACs reported here are released almost exclusively at <0.1% O_2_, which is a level of O_2_ associated with the most aggressive component of a tumor. Physiological levels of O_2_, meanwhile, are typically between 1.5% (bone marrow) and 14% (alveoli),^48^ indicating that the use of indolequinone-based HAP-TACs will provide a useful therapeutic window between diseased and healthy tissue. We have recently shown that modulation of indolequinone substitution, or leaving group p*K*_a_, affects the level at oxygen at which the cargo (e.g., PROTAC) is released.^33^ This provides an avenue for optimization of the O_2_ level the HAP-TAC is activated at, should this become necessary during in vivo studies. In addition, studies on the bioreductive release of the diterpenoid oridonin from an indolequinone-based precursor have previously been reported.^50^ The prodrug was administered intravenously into mice bearing an H22-derived tumor xenograft, resulting in a 64% reduction in tumor size, with no effect on animal weight, indicating minimal off-target toxicity. These data indicate that the indolequinone moiety is suitable for initial in vivo studies, at least. Although further refinement of the bioreductive group will likely afford enhanced cellular stability in normoxia, HAP-TACs represent an exciting strategy to target protein degradation to disease-relevant tissue while minimizing the effect on healthy tissue and thus preventing unwanted side effects. As the HAP-TACs incorporate the bioreductive group on the E3 ligase ligands, this strategy can, in principle, be applied to any protein that is degraded by either CRBN or VHL, which is currently the vast majority of PROTACs reported.

## Supplementary Material



## Notes

Biological methods, chemical experimental section, supplementary figures, and tables, NMR spectra, and HPLC traces for new compounds (PDF)

## ¶

Department of Drug Science and Technology, University of Turin, 10125 Turin, Italy

## ∇

School of Chemistry, University of Edinburgh, Joseph Black Building, David Brewster Road, City, Edinburgh, EH9 3FJ, UK.
