# Supplementary material for: Indolequinone-Based Hypoxia-Activated Proteolysis Targeting Chimeras Selectively Degrade BRD4 in Hypoxic Cancer Cells
Source: J Am Chem Soc. 2025 Sep 25;147(40):36352–64. doi: 10.1021/jacs.5c10240 (PMC12512183; doi:10.1021/jacs.5c10240)
Supplement: Supplementary file 1 [file ja5c10240_si_001.pdf]

Indolequinone-based hypoxia-activated proteolysis targeting chimeras selectively degrade BRD4 in hypoxic cancer cells

Marta Serafini,[1,†] Sophie A. Twigger,[2] George Delfas,[1] Maxim Mallerman,[1] Elliot P. Bailey,[1] Ewen D. D. Calder,[1,‡] Ester M. Hammond,[2] and Stuart J. Conway[1,3,4,5,6,*]

Supporting Information

[†]Current address: Department of Drug Science and Technology, University of Turin, 10125 Turin, Italy

[‡]Current address: School of Chemistry, University of Edinburgh, Joseph Black Building, David Brewster Road, City, Edinburgh, EH9 3FJ, UK

*To whom correspondence should be addressed: stuartconway@ucla.edu

[1]Department of Chemistry, Chemistry Research Laboratory, University of Oxford, Mansfield Road, Oxford, OX1 3TA, UK

[2]Department of Oncology, University of Oxford, Old Road Campus Research Building, Oxford, OX3 7DQ, UK

[3]Department of Chemistry & Biochemistry, University of California Los Angeles, 607 Charles E. Young Drive East, Los Angeles, California, 90095, USA

[4]California NanoSystems Institute, University of California Los Angeles, 570 Westwood Plaza, Building 114, Los Angeles, CA 90095, USA

[5]Jonsson Comprehensive Cancer Center, University of California Los Angeles, 8-684 Factor Building, Los Angeles, CA 90095, USA

[6]Molecular Biology Institute, University of California Los Angeles, 611 Charles E. Young Drive East, Los Angeles, CA 90095, USA

Table of contents

1. Chemical and enzymatic reduction of compounds 3

2. Biological methods 19

3. Chemistry experimental section 24

General chemical methods 24

Synthetic Schemes 27

Synthetic procedures and compound data 32

4. NMR spectra 76

5. HPLC traces 113

6. Abbreviations 123

7. References 123

1. Chemical and enzymatic reduction of compounds

Chemical reduction of compounds

General procedure A for the chemical reduction of nitroaryl derivatives: To a round-bottom flask containing DMF (2 mL), 1 mg of compound was added, followed by a 10% aqueous NH_4Cl solution (20 μL). An aliquot (100 μL) was collected from the reaction solution and quenched with 600 μL of CH_3CN. This serves as $T = 0$ (where T = time). Then, zinc powder (5 mg) was added, and the reaction mixture was stirred at rt. At the timepoints shown, aliquots were collected, quenched with 600 μL of CH_3CN, filtered using a syringe filter (Gilson PTFE-4-4, size × porosity: 13 mm × 0.45 μm), injected and analyzed using HPLC.

Enzymatic reduction of compounds

The oxygen-dependent enzymatic reduction assays were run in a RUSKINN InvivoO_2® 400 hypoxic workstation at 37 °C with 0.1% oxygen and 99.9% nitrogen concentrations unless otherwise stated. Bactosomal human NADPH-CYP reductase (CYP004, Cypex) was used in combination with NADPH regenerating system (solution A, Cat. No 451220 and solution B, Cat. No 451200, Corning). Phosphate buffer, MilliQ water and absolute ethanol used in the assays were equilibrated under the conditions stated above in the hypoxic workstation overnight before use. LCMS or HPLC analysis were used as readout for reaction analysis.

General procedure B for LCMS assay: To a 1.5 mL Eppendorf tube in a Bactron II anaerobic chamber (Shell labs), MilliQ water (729.3 μL), phosphate buffer (0.5 M, pH 7.4, 200 μL), regenerating solution A (50 μL), regenerating solution B (10 μL), and a 10 mM DMSO solution of compound (1 μL, final concentration 10 μM) were added. An aliquot (50 μL) was collected from the reaction solution and quenched with 50 μL of acetonitrile. This serves as $T = 0$ (where T = time). Then, NADPH-CYP reductase (9.7 μL, final concentration 92 pmol/mL) was added to the reaction mixture. At selected timepoints, aliquots were collected and quenched in the hypoxic workstation with 50 μL of acetonitrile. The samples were removed from the hypoxic workstation and placed on ice before being centrifuged at 13,000 *g* for 10 min at 4 °C. The supernatant was collected and 25 μL were injected and analyzed using LCMS.

General procedure C for LCMS assay: To a 1.5 mL Eppendorf tube in the hypoxic workstation, MilliQ water (874.7 μL), phosphate buffer (0.5 M, pH 7.4, 240 μL), regenerating solution A (60 μL), regenerating solution B (12 μL), and a 10 mM DMSO solution of compound (2 μL, final concentration 20 μM) were added. An aliquot (200 μL) was collected from the reaction solution and quenched with 200 μL of methanol. This serves as $T = 0$ (where

T = time). Then, NADPH-CYP reductase (11.3 μL, final concentration 92 pmol/mL) was added to the reaction mixture. At selected timepoints, aliquots were collected and quenched in the hypoxic workstation with 200 μL of methanol. The samples were removed from the hypoxic workstation and placed on ice before being centrifuged at 13,000 *g* for 10 min at 4 °C. The supernatants were extracted with a 90:10 mixture of CH_2Cl_2:methanol (4 × 200 μL), filtered using a syringe filter (Gilson Nylon Syringe Filter, size × porosity: 13 mm × 0.45 μm) and evaporated. The residue was dissolved in 200 μL of water with 0.5% of DMSO, and 100 μL were injected and analyzed using LCMS.

General procedure D for normoxia HPLC assay: To a 1.5 mL Eppendorf tube, MilliQ water (744.7 μL), phosphate buffer (0.5 M, pH 7.4, 240 μL), absolute ethanol (120 μL), regenerating solution A (60 μL), regenerating solution B (12 μL), and a 2 mM DMSO solution of compound (12 μL, final concentration 20 μM) were added. An aliquot (200 μL) was collected from the reaction solution and quenched with 200 μL of methanol. This serves as $T = 0$ timepoint. Then, NADPH-CYP reductase (11.3 μL, final concentration 92 pmol/mL) was added to the reaction mixture. At selected timepoints, aliquots were collected and quenched in the hypoxic workstation with 200 μL of methanol. The samples were removed from the hypoxic workstation and placed on ice before being centrifuged at 13,000 *g* for 10 min at 4 °C. The supernatants were filtered using a syringe filter (Gilson Nylon Syringe Filter, size × porosity: 13 mm × 0.45 μm), 100 μL were injected and analyzed using HPLC.

General procedure E for hypoxia HPLC assay: To a 1.5 mL Eppendorf tube in the hypoxic workstation, MilliQ water (744.7 μL), phosphate buffer (0.5 M, pH 7.4, 240 μL), absolute ethanol (120 μL), regenerating solution A (60 μL), regenerating solution B (12 μL), and a 2 mM DMSO solution of compound (12 μL, final concentration 20 μM) were added. An aliquot (200 μL) was collected from the reaction solution and quenched with 200 μL of methanol. This serves as $T = 0$ timepoint. Then, NADPH-CYP reductase (11.3 μL, final concentration 92 pmol/mL) was added to the reaction mixture. At selected timepoints, aliquots were collected and quenched in the hypoxic workstation with 200 μL of methanol. The samples were removed from the hypoxic workstation and placed on ice before being centrifuged at 13,000 *g* for 10 min at 4 °C. The supernatants were filtered using a syringe filter (Gilson Nylon Syringe Filter, size × porosity: 13 mm × 0.45 μm), 100 μL were injected and analyzed using HPLC.

General procedure F for hypoxia control HPLC assay: To a 1.5 mL Eppendorf tube in the hypoxic workstation, MilliQ water (756 μL), phosphate buffer (0.5 M, pH 7.4, 240 μL), absolute ethanol (120 μL), regenerating solution A (60 μL), regenerating solution B (12 μL),

and a 2 mM DMSO solution of compound (12 µL, final concentration 20 µM) were added. The mixture was left in the hypoxic workstation and at selected timepoints aliquots (200 µL) were collected from the reaction solution and quenched with 200 µL of methanol. The samples were removed from the hypoxic workstation and placed on ice before being centrifuged at 13,000 *g* for 10 min at 4 °C. The supernatants were filtered using a syringe filter (Gilson Nylon Syringe Filter, size × porosity: 13 mm × 0.45 µm), 100 µL were injected and analyzed using HPLC.

Figure S1. Compound 1 is reduced to the corresponding aniline but is not further fragmented to give VH032. Enzymatic reduction of compound **1** using NADPH-CYP reductase. Compound **1** was incubated with NADPH-CYP reductase over 48 h in hypoxia. Aliquots were taken at selected timepoints, processed and analyzed using LC-MS, as described in the general procedure B for the LCMS assay.

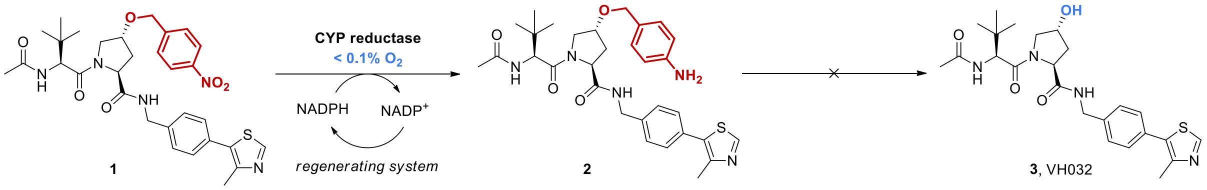

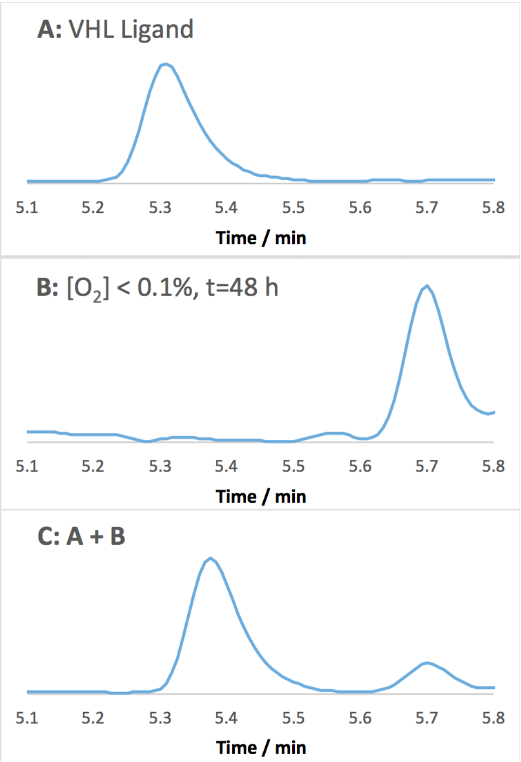

Figure S2. Compound 4 bearing a carbonate-linked 1-methyl-2-nitroimidazole group is reduced to the corresponding Boc protected VH032 with zinc. Compound **4** was incubated with zinc and NH_4Cl (aq.). Aliquots were taken at $T = 0$, 0.5, 1 and 2 h, processed and analyzed using HPLC as described in the general procedure A. Absorbance was recorded at 254 nm. For the sake of clarity, the injection peak was omitted, and the chromatogram was reported after 2 min from the injection onwards. Only $T = 0$, 0.5, and 2 h are shown.

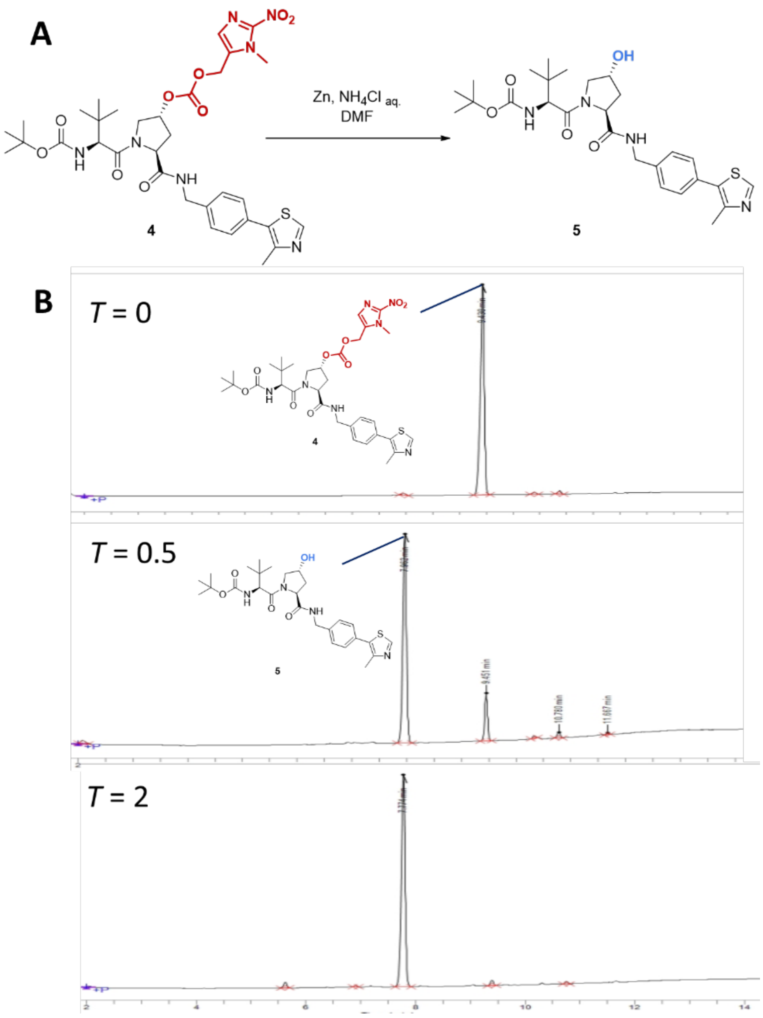

Figure S3. NB-POMA 10 is not reduced over time to release pomalidomide 11. (**A**) Chemical reduction of NB-POMA **10** with zinc. (**B**) NB-POMA **10** was incubated with zinc over 24 h. In parallel, pomalidomide **11** was treated under the same conditions. Aliquots were taken at T = 0, 1, 2, 4, 6 and 24 h, processed and analyzed using HPLC as described in the general procedure A. Absorbance was recorded at 220 nm. For the sake of clarity, the injection peak was omitted, and the chromatogram was reported after 2 min from the injection onwards. Only T = 0, 6, and or 24 h for NB-POMA **10** and T = 0 for pomalidomide are shown.

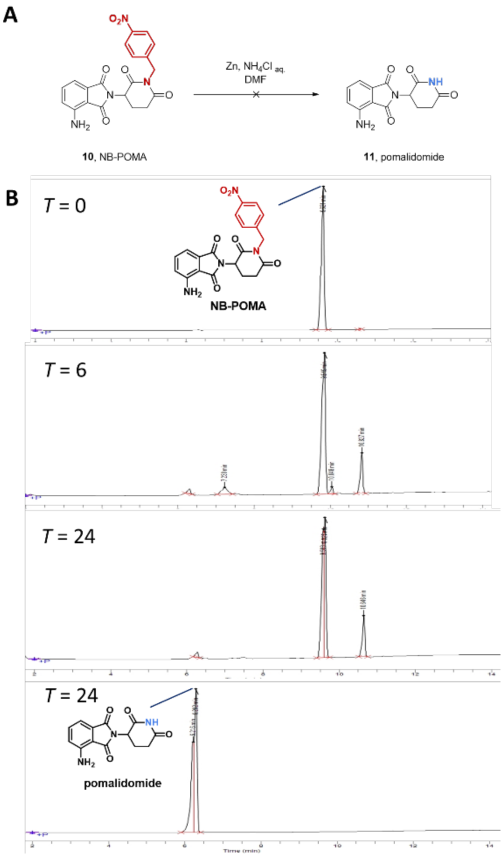

Figure S4. NI-POMA 12 is reduced over time to release pomalidomide 11. Chemical reduction of NI-POMA **12** with zinc. (**B**) NI-POMA **12** was incubated with Zn(0) over 24 h. In parallel, pomalidomide **11** was treated under the same conditions. Aliquots were taken at *T* = 0, 1, 2, 4, 6 and 24 h, processed and analyzed using HPLC, as described in the general procedure A. Absorbance was recorded at 220 nm. For the sake of clarity, the injection peak was omitted, and the chromatogram was reported after 2 min from the injection onwards. Only *T* = 0, 6, and 24 h for NI-POMA **12** and *T* = 0 for pomalidomide **11** are shown.

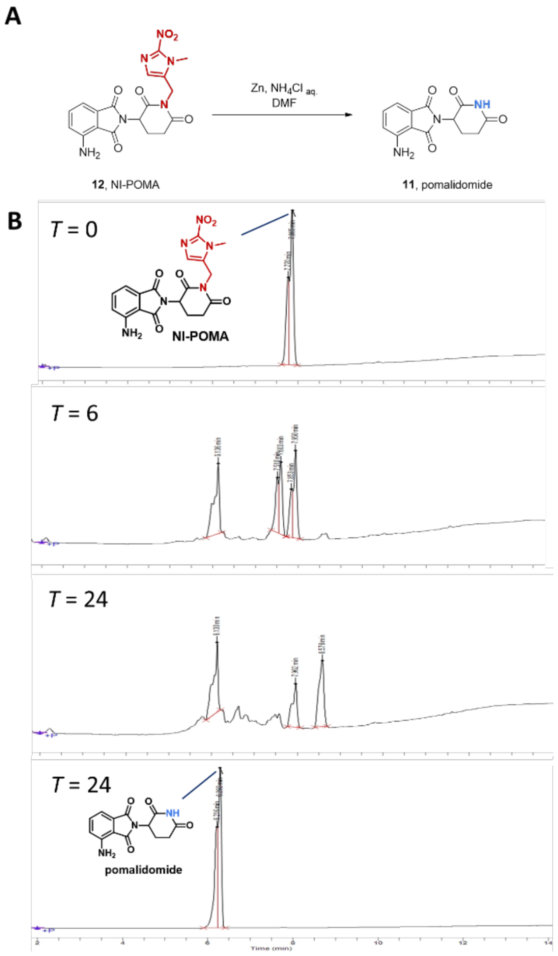

Figure S5. NI-POMA 12 is degraded over time in the presence of NADPH-CYP reductase under hypoxia. (**A**) Enzymatic reduction of NI-POMA **12** with NADPH-CYP reductase. (**B**) NI-POMA **12** was incubated with NADPH-CYP reductase over 24 h in hypoxia. Aliquots were taken at $T = 0$, 3, 7, and 24 h, processed and analyzed using LCMS, as described in the general procedure C for the LCMS assay. One representative HPLC trace of three independent experiments is reported. Absorbance was recorded at 220 nm. For the sake of clarity, the injection peak was omitted, and the chromatogram was reported after 2 min from the injection onwards.

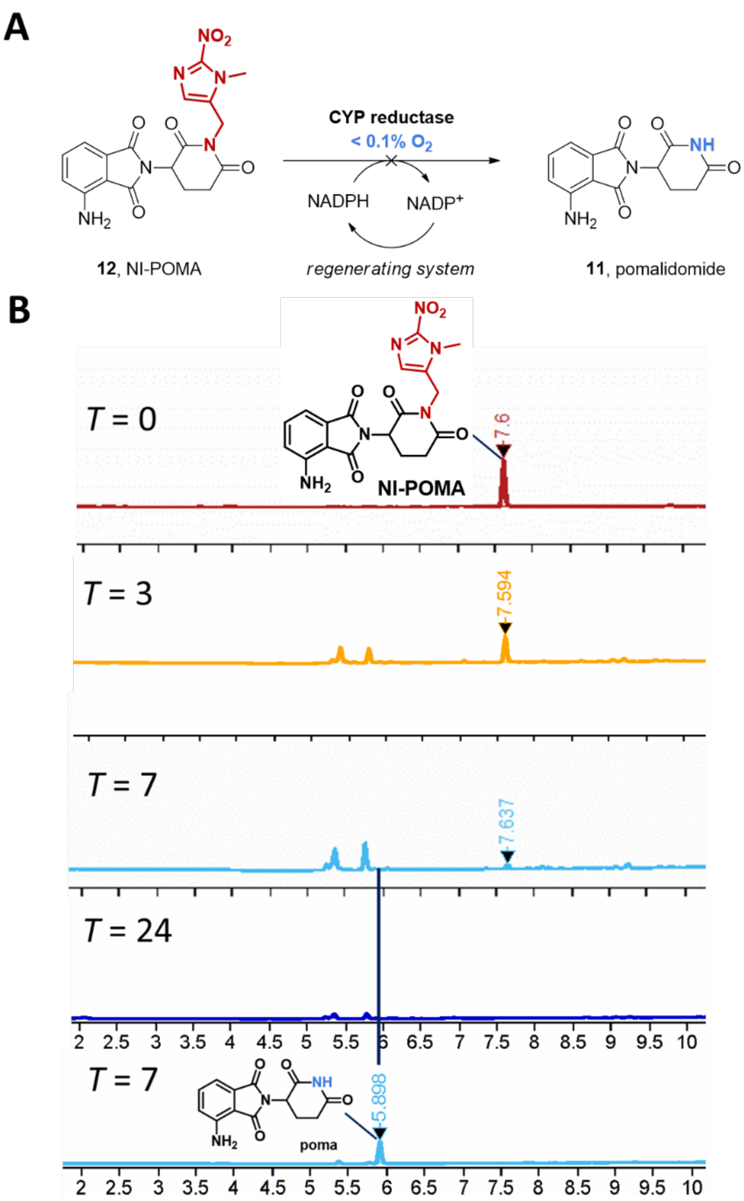

Figure S6. Residual substrate after 24 h of incubation with NI-VHL **6** or IQ-VHL **7** (**A**) and NI-CRBN **15** or IQ-VHL **16** (**B**) in normoxia (21% O_2, red bars) or hypoxia (<0.1% O_2, blue bars) with NADPH-CYP reductase or in the absence of the enzyme (control, light blue bars). Data are the mean of three independent experiments ± s.e.m. (**C**) Residual substrate of the active PROTACs MZ1 **8** and PG-4c **17** and the negative controls Bn-VHL **9** and Bn-CRBN **18** after 24 h incubation in (<0.1% O_2, blue bars) with NADPH-CYP reductase or in the absence of the enzyme (control, light blue bars). Data are the mean of three independent experiments ± s.e.m.

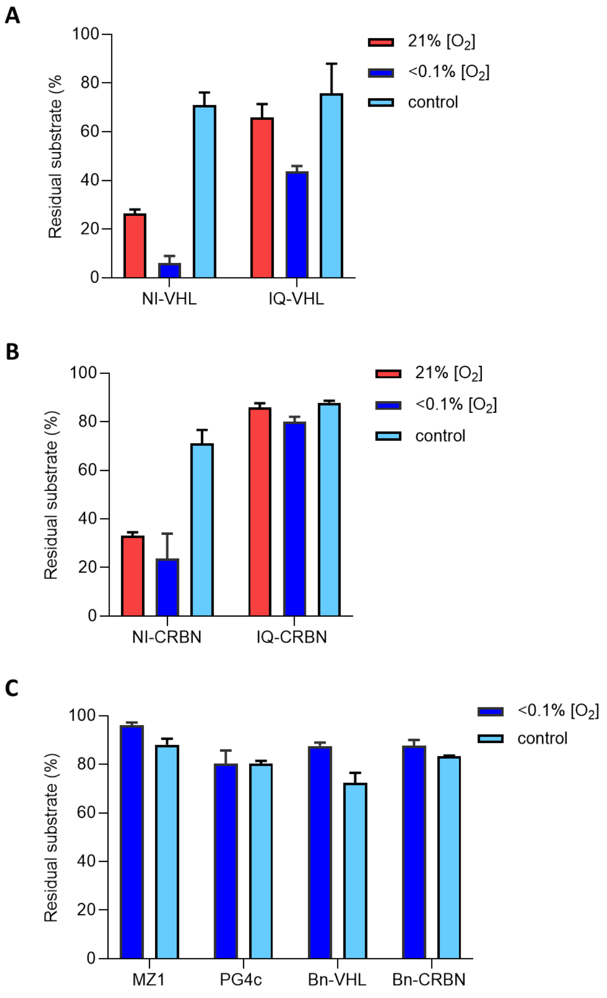

Figure S7. NI-VHL 6 does not show oxygen- and enzyme-dependent reduction and corresponding release of the active PROTAC MZ1 (8). (**A**) Enzymatic reduction of NI-VHL **6** using NADPH-CYP reductase. (**B**) NI-VHL **6** was incubated with NADPH-CYP reductase over 24 h in normoxia or hypoxia. Additionally, the compound was incubated in hypoxia in the absence of the NADPH-CYP reductase (hypoxia control). Aliquots were taken at *T* = 0 and 24 h, processed and analyzed using HPLC, as described in the general procedures D, E and F for the HPLC enzyme-based assay. One representative HPLC trace of three independent experiments is reported. Only the hypoxia *T* = 0 is shown in the figure. Absorbance was recorded at 254 nm. For the sake of clarity, the injection peak was omitted, and the chromatogram was reported after 2 min from the injection onwards.

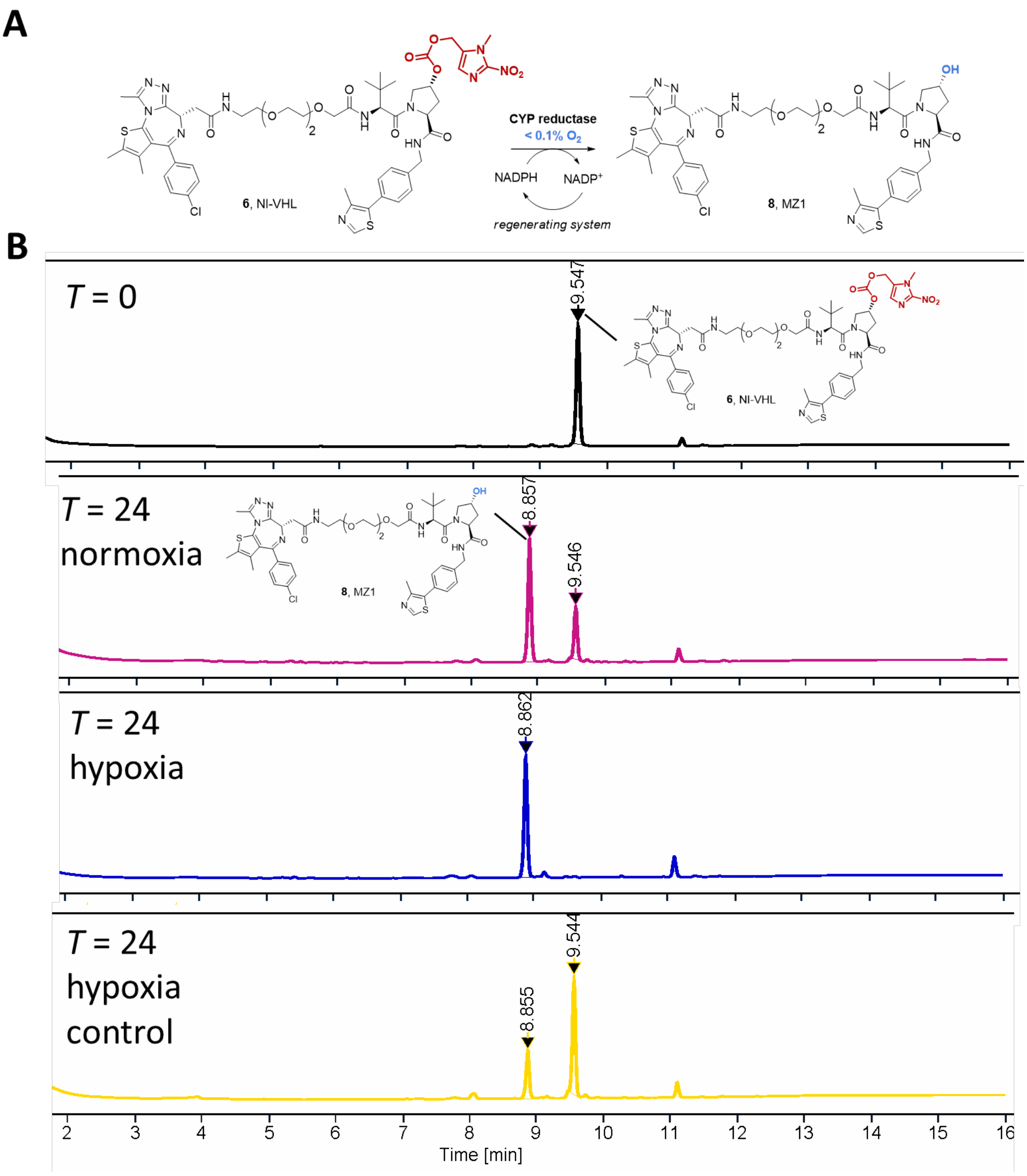

Figure S8. IQ-VHL 7 undergoes oxygen- and enzyme-dependent reduction to the corresponding active PROTAC MZ1 (8). (**A**) Enzymatic reduction of IQ-VHL **7** using NADPH-CYP reductase. (**B**) IQ-VHL **7** was incubated with NADPH-CYP over 24 h in normoxia or hypoxia. Additionally, the compound was incubated in hypoxia in the absence of the NADPH-CYP reductase (hypoxia control). Aliquots were taken at *T* = 0 and 24 h, processed and analyzed using HPLC, as described in the general procedures D, E and F for the HPLC enzyme-based assay. One representative HPLC trace of three independent experiments is reported. Only the hypoxia *T* = 0 is shown in the figure. Absorbance was recorded at 254 nm. For the sake of clarity, the injection peak was omitted, and the chromatogram was reported after 2 min from the injection onwards.

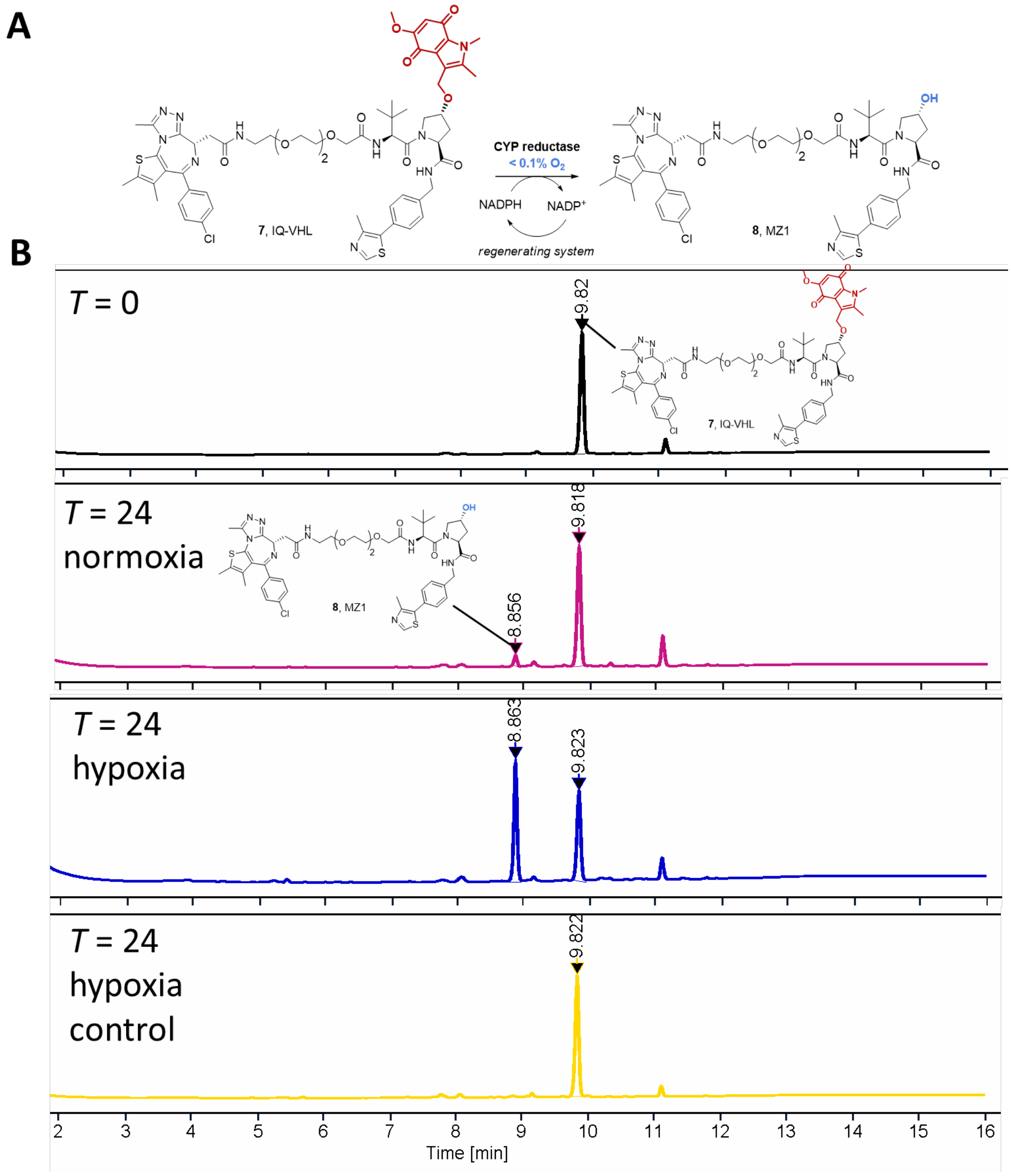

Figure S9. NI-CRBN 15 does not show oxygen- and enzyme-dependent reduction to the corresponding active PROTAC PG-4c (17). (**A**) Enzymatic reduction of NI-CRBN **15** using NADPH-CYP reductase. (**B**) NI-CRBN **15** was incubated with NADPH-CYP reductase over 24 h in normoxia or hypoxia. Additionally, the compound was incubated in hypoxia in the absence of the NADPH-CYP reductase (hypoxia control). Aliquots were taken at $T = 0$ and 24 h, processed and analyzed using HPLC, as described in the general procedures D, E and F for the HPLC enzyme-based assay. One representative HPLC trace of three independent experiments is reported. Only the hypoxia $T = 0$ is shown in the figure. Absorbance was recorded at 254 nm. For the sake of clarity, the injection peak was omitted, and the chromatogram was reported after 2 min from the injection onwards.

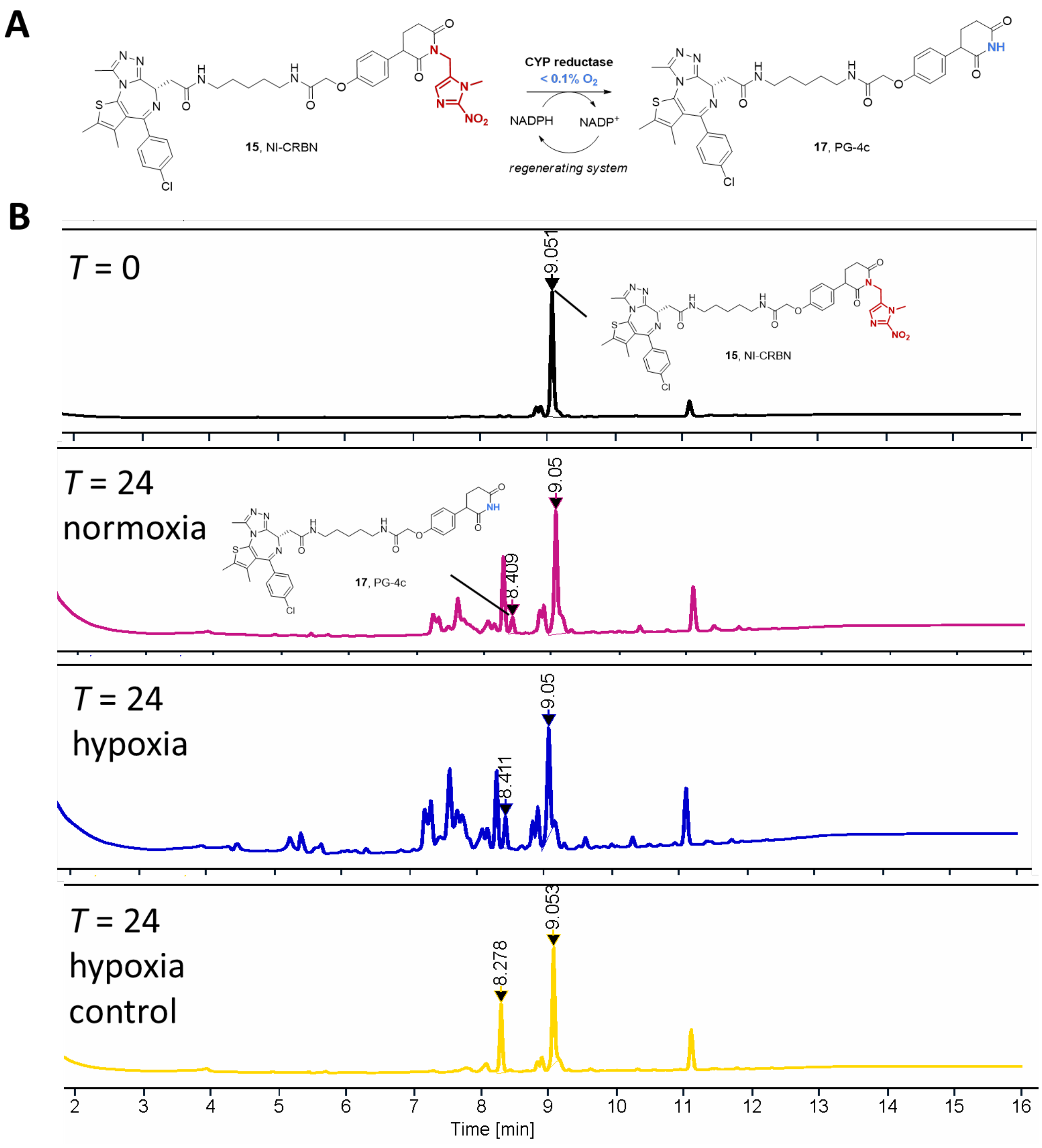

Figure S10. IQ-CRBN 16 undergoes oxygen- and enzyme-dependent reduction to the corresponding active PROTAC PG-4c (17). (**A**) Enzymatic reduction of IQ-CRBN **16** using NADPH-CYP reductase. (**B**) IQ-CRBN **16** was incubated with NADPH-CYP reductase over 24 h in normoxia and hypoxia. Additionally, the compound was incubated in hypoxia in the absence of the NADPH-CYP reductase (hypoxia control). Aliquots were taken at $T = 0$ and 24 h, processed and analyzed using HPLC, as described in the general procedures D, E and F for the HPLC enzyme-based assay. One representative HPLC trace of three independent experiments is reported. Only the hypoxia $T = 0$ is shown in the figure. Absorbance was recorded at 254 nm. For the sake of clarity, the injection peak was omitted, and the chromatogram was reported after 2 min from the injection onwards.

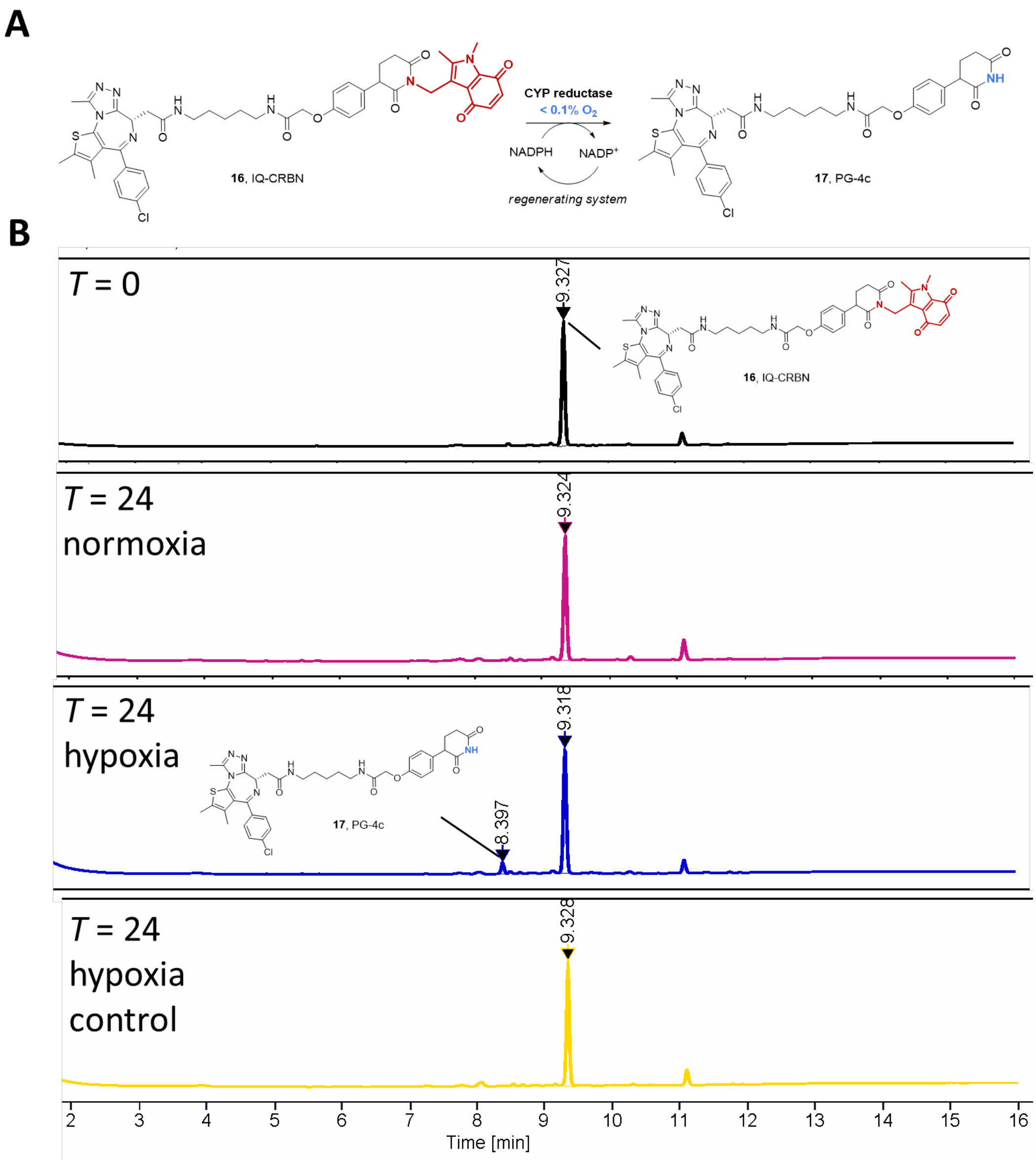

Figure S11. The active PROTACs MZ1 4 and PG-4c 11 and the negative controls Bn-VHL 3 and Bn-CRBN 10 are stable in the presence of the NADPH-CYP reductase. 20 µM of compounds MZ1 **8** (**A**), PG-4c **17** (**B**), Bn-VHL **9** (**C**), and Bn-CRBN **18** (**D**) were incubated in hypoxia with (hypoxia) or without (hypoxia control) NADPH-CYP reductase over 24 h. Aliquots were taken at *T* = 0 and 24 h, processed and analyzed using HPLC, as described in the general procedures D, E and F for the HPLC enzyme-based assay. One representative HPLC trace of three independent experiments is reported. For each compound, only the hypoxia *T* = 0 is shown in the figure. Absorbance was recorded at 254 nm. For the sake of clarity, the injection peak was omitted, and the chromatogram was reported after 2 min from the injection. onwards

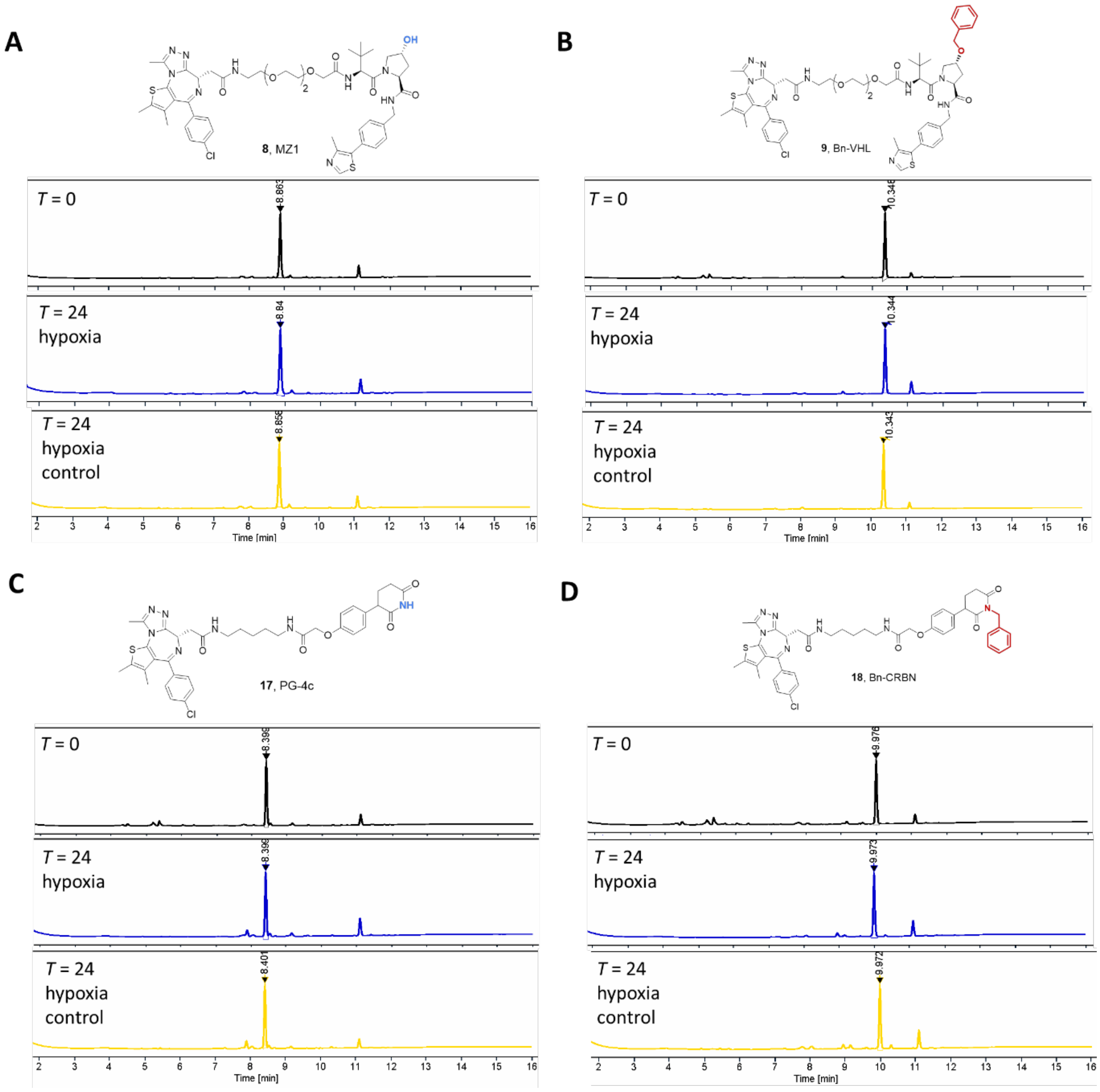

Figure S12. Time course experiment for the four HAP-TACs in hypoxia. 20 μM of compounds NI-VHL **6** (**A**), IQ-VHL **7** (**B**), NI-CRBN **15** (**C**), and IQ-CRBN **16** (**D**) were incubated with NADPH-CYP reductase over 24 h in hypoxia. For compounds IQ-VHL **7**, NI-CRBN **15** and IQ-CRBN **16**, aliquots were taken at $T = 0$, 2, 4, 8 (not shown), 11, and 24 h, while for compound NI-VHL **6** aliquots were taken at $T = 0$, 0.5, 1, 2 and 4 h, processed and analyzed using HPLC, as described in the general procedure E for the HPLC enzyme-based assay. For each timepoint, one representative HPLC trace of three independent experiments is reported. Absorbance was recorded at 254 nm. For the sake of clarity, the injection peak was omitted, and the chromatogram was reported after 2 min from the injection onwards.

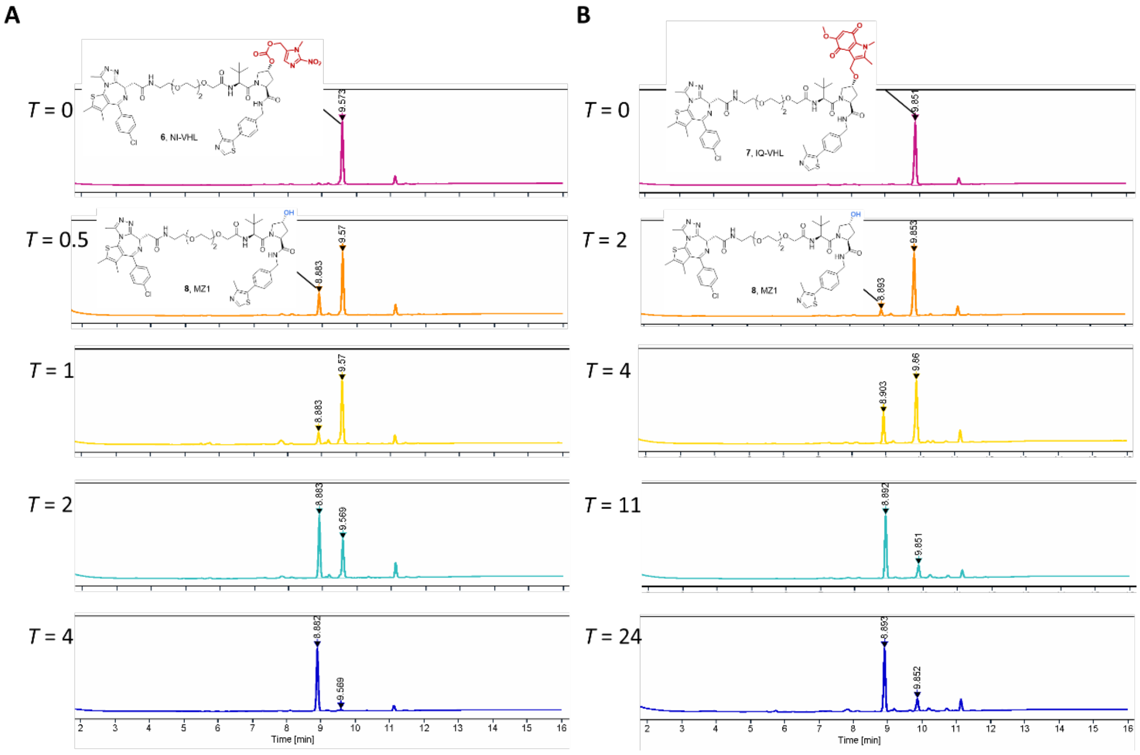

C

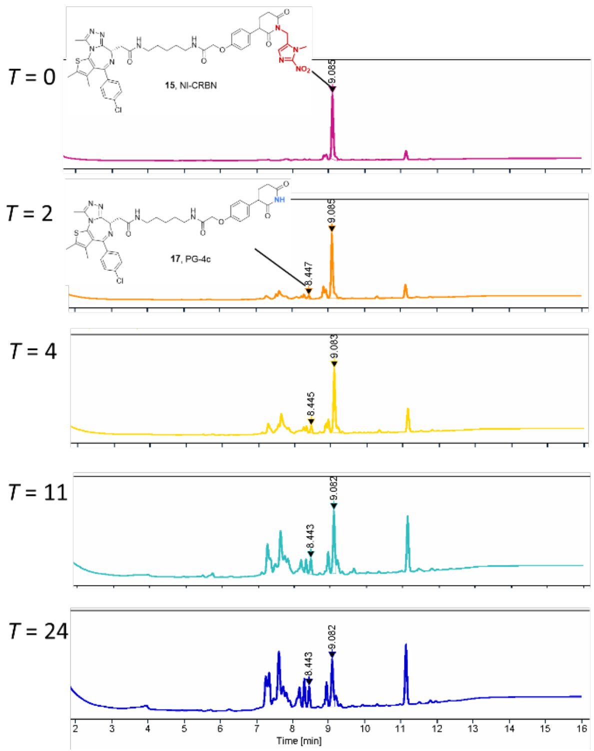
T = 0
15, NI-CRBN
9.085
T = 2
17, PG-4c
8.447
9.085
T = 4
8.445
9.083
T = 11
8.443
9.082
T = 24
8.443
9.082
Time [min]

D

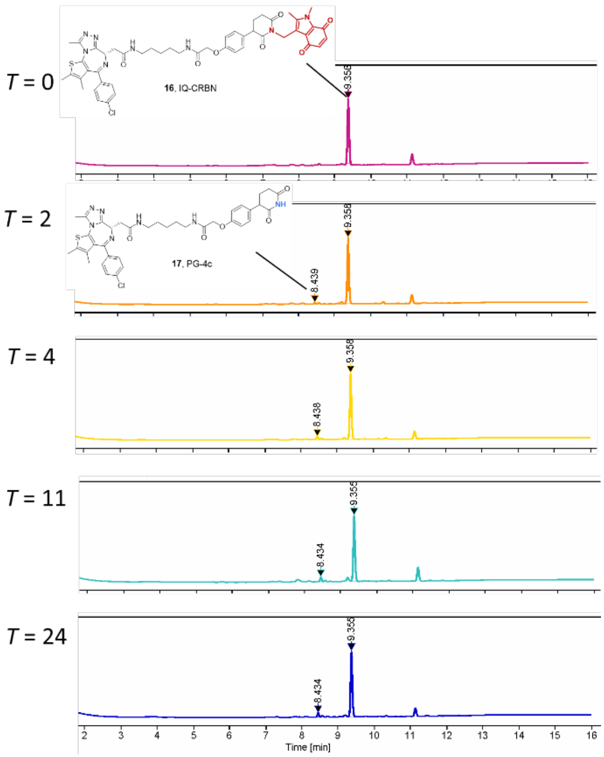
T = 0
16, IQ-CRBN
9.358
T = 2
17, PG-4c
8.439
9.358
T = 4
8.438
9.358
T = 11
8.434
9.355
T = 24
8.434
9.355
Time [min]

2. Biological methods

Cell Lines and Reagents

Colorectal HCT116 and lung adenocarcinoma A549 cells were grown in Dulbecco's Modified Eagle Medium (DMEM) with high glucose, GlutaMAX™ and pyruvate supplemented with 1% penstrep and 10% FBS. Cells were maintained in an incubator set at 37 °C and 5% CO_2. All cell lines were routinely tested for mycoplasma using MycoAlert Detection Kit (Lonza).

Normoxic Treatments

Cells were seeded on plastic dishes and allowed to settle overnight at 37 °C and 5% CO_2. Cells were treated with the tested compounds in 1% DMSO at the indicated concentrations and incubated at 37 °C and 5% CO_2 for 24 h. Cells were harvest and lysed in UTB buffer (9 M urea, 75 mM Tris-HCl, pH 7.5, 0.1M β-mercaptoethanol).

Hypoxic Treatments

Hypoxia treatments at <0.1% O_2 were carried out in a Bactron II anaerobic chamber (Shel Labs) while all oxygen concentrations from 0.5-2% O_2 were carried out in a M35 variable atmosphere workstation (Don Whitley Scientific). Oxygen concentrations were periodically validated using anaerobic oxygen indicator strips (ThermoFisher). Cells were seeded on glass dishes and allowed to settle overnight. Then, they were placed inside the chamber with equilibrated solutions for 4 h, before treatment with the tested compounds in 1% DMSO at the indicated concentrations. For the proteasome/neddylation experiment, cells were pre-treated with either the proteasome or the neddylation inhibitor for 4 h inside the chamber, then treated with the tested compounds in 1% DMSO at the indicated concentrations. After the time required for the experiment, cells were harvest, and lysed in UTB buffer while inside the chamber.

MTT assay

HCT116 cells were seeded at 6,000 cells/well in 96-well plates and allowed to adhere overnight. Cells were treated with the tested compounds in 1% DMSO at the indicated concentration for 24 hours, in hypoxia or normoxia, then the media was removed and replaced with fresh, compound-free, media. After additional 24 hours in normoxia, cells were incubated with 0.5 mg/mL MTT reagent (Sigma Aldrich) in complete media for 3 hours at 37 °C protected from light. MTT was removed and formazan crystals were dissolved in 100 µL of DMSO for 15 minutes at 37 °C while protected from light. Absorbance was read at 570 nm using a FLUOstar Omega plate reader. Data are shown as percentage viability relative to DMSO

control and are the result of three biological replicates with three technical replicates each ± s.e.m.

Cell proliferation

HCT116 cells were seeded at 8,300 cells/well in 24-well plates and allow to adhere overnight. Cells were treated with the tested compounds in 1% DMSO at the indicated concentration for 24 hours, in hypoxia or normoxia, then media was removed and replaced with fresh, compound-free, media. Cells were then incubated in normoxia. At endpoints, cells were suspended and mixed with trypan blue at 1:1 before proceeding to cell count using a haemocytometer. Mean values from three technical replicates of cell counts were calculated and three biological replicates were plotted with s.e.m.

Immunoblotting

Cells lysates were sonicated (3 × 10 sec bursts, 10 sec pause on ice) on a FB-505 sonic dismembrator with a 1/16" tapered microtip at 40% maximum power. The lysates were centrifuged (4 °C, 16,000 × g, 10 min) and the supernatants were transferred to a fresh tube. Protein concentrations were measured using a Pierce™ BCA Protein Assay Kit (Thermo Fisher Scientific). Proteins were diluted in MilliQ water and 10% β-mercaptoethanol in Laemmli sample buffer (Bio-Rad), and denatured at 100 °C for 5 min, then separated by SDS-PAGE (Mini-PROTEAN TGX gels, Bio-Rad, 8-20%) and compared to the Spectra™ Multicolor High Range Protein Ladder (Thermo Fisher Scientific). Semi-dry blotting was conducted on a Trans-Blot Turbo Transfer System (Bio-Rad) with the Trans-Blot Turbo RTA Mini 0.2 μm PVDF Transfer Kit (Bio-Rad) using the default high molecular weight mode. Membranes were blocked in 5% non-fat dry milk (Bio-Rad) in TBS-T for 1-2 h. Primary BRD4 (E2A7X, Cell Signaling Technology Europe), c-Myc (D84C12, Cell Signaling Technology Europe), HIF-1α (610958, BD Bioscience) and β-actin (sc-69879, Santa Cruz Biotechnologies) antibodies were used in concentrations recommended by the manufacturer, diluted in 5% non-fat dry milk in TBS-T. Membranes were incubated with primary antibodies overnight at 4 °C, washed with TBS-T (3×), incubated with HRP-conjugated secondary antibodies (Promega UK) for 1 h at rt, washed with TBS-T (3×) and imaged with Clarity western ECL substrate (Bio-Rad). Membranes were scanned with a Bio-Rad ChemiDoc XRS+ Imaging System and signal intensities were quantified using Image Lab software (Bio-Rad). Band densitometry was assessed, normalized to β-actin bands, and reported as percentage of the DMSO control lane.

Figure S13 A. Percentage of BRD4 levels compared to control, assessed using western blot analysis, after 24 h incubation in A549 cells with indicated concentrations of MZ1 **8**, PG-4c **17** or (+)-JQ1. Data are the mean of three independent experiments ± s.e.m. (**B**) Representative western blot analysis of the data showed in panel **A** with β-actin used as loading control.

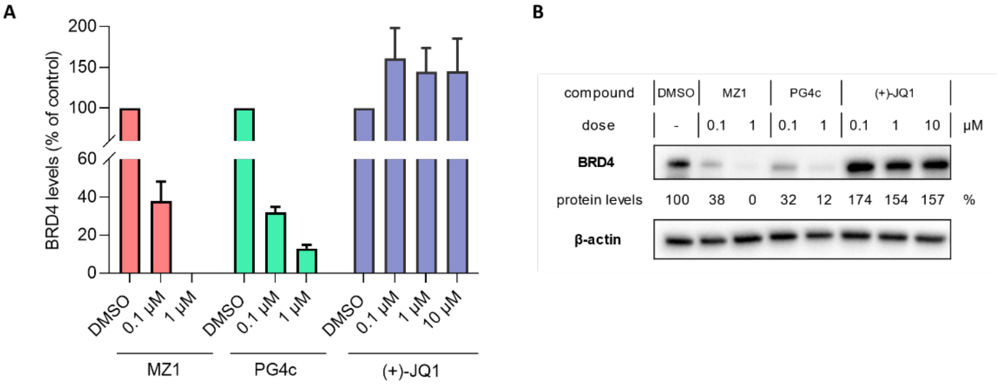

Figure S14 A. Percentage of BRD4 levels compared to control, assessed using western blot analysis, after 24 h incubation in HCT116 cells with indicated concentrations of MZ1 **8**, PG-4c **17** or (+)-JQ1. Data are the mean of three independent experiments ± s.e.m. (**B**) Representative western blot analysis of the data showed in panel **A** with β-actin used as loading control.

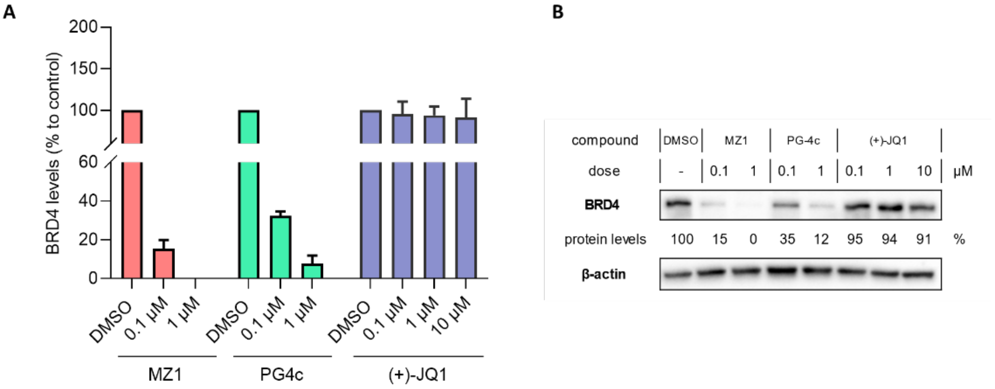

Figure S15. Concentration-response curves of MTT assay in HCT116 cells treated with compounds MZ1 **8** (**A**), IQ-VHL **7** (**B**), PG-4c **17** (**C**) or IQ-CRBN **16** (**D**) in normoxia (21% O_2, red line) or hypoxia (<0.1% O_2, blue line). Data are the mean of three independent experiments ± s.e.m.

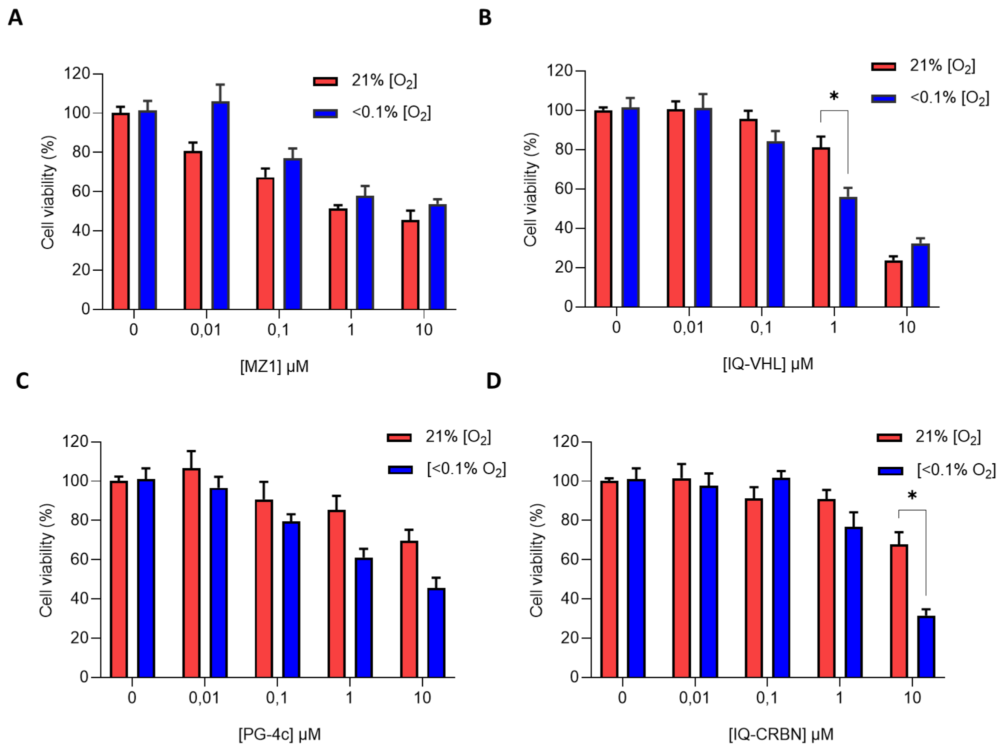

Figure S16. Growth over time of HCT116 cells treated with compounds IQ-VHL **7** (0.25 µM) (**A**) or IQ-CRBN **16** (3 µM) (**B**) in normoxia (red and orange lines) and hypoxia (blue and light blue lines). Data are the mean of three independent experiments ± s.e.m.

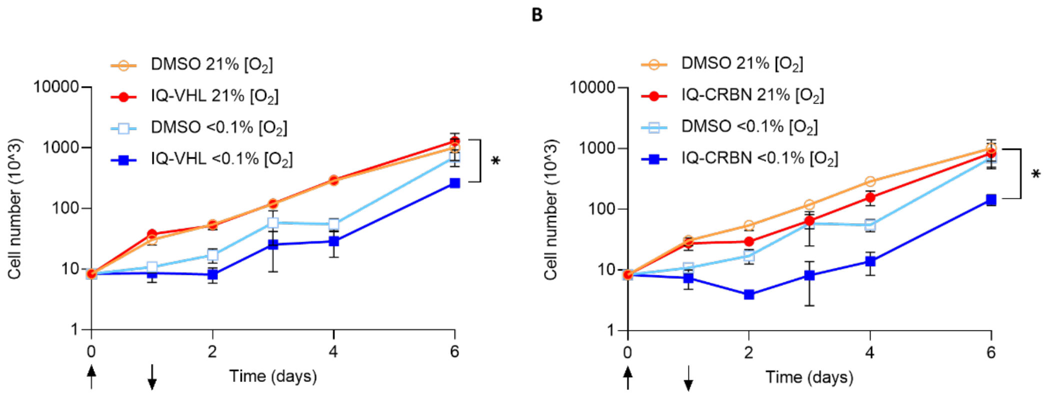

Figure S17 A. Concentration-response curves of MTT assay of HCT116 cells treated with (+)-JQ1 in normoxia (21% O_2, red line) or hypoxia (<0.1% O_2, blue line); (**B**) Cells number related to control (DMSO) of HCT116 cells treated with (+)-JQ1 at the indicated concentrations in normoxia over 6 days. Data are the mean of three independent experiments ± s.e.m.

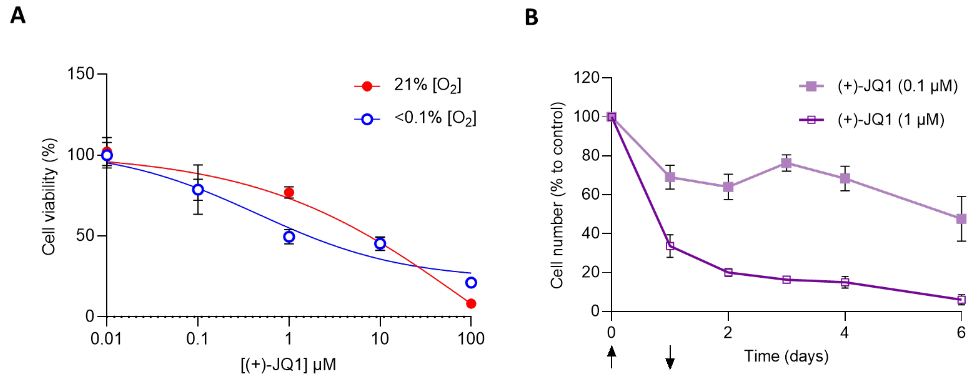

3. Chemistry experimental section

General chemical methods

Reagents and solvents were purchased from commercial sources (Sigma-Aldrich, Fluorochem, and Alfa Aesar) and were used without further purification, unless otherwise stated. Anhydrous solvents were taken from MBRAUN Solvent Purification System 5 and stored over 3 Å molecular sieves under an argon atmosphere. *In vacuo* refers to solvent removal under reduced pressure at 45 °C using a Buchi™ rotary evaporator. Petroleum ether refers to the fraction of light petroleum ether boiling in the range 40– 60 °C when distilled. The term Fremy's salt refers to potassium nitrosodisulfonate (purchased from Sigma-Aldrich). The abbreviation PS-PPh_3 refers to polymer-bound triphenylphosphine (366455, Sigma-Aldrich). The *n* numbers in italic in the "reagents and conditions" sections (*e.g.* *n* = 3) refer to the times each reaction has been repeated.

^{1}H NMR spectra were recorded using a Bruker AVH400 (400 MHz), Bruker AVC500 (500 MHz) or Bruker NEO 600 (600 MHz) spectrometers using the stated solvents as a reference for internal deuterium lock. The chemical shift data for each signal are given as δ_H in units of parts per million (ppm) relative to the tetramethyl silane (TMS) where δ_H (TMS) is 0.00 ppm. The multiplicity of each signal is indicated by s (singlet), br s (broad singlet), d (doublet), t (triplet), br t (broad triplet), q (quartet), quint (quintet), dd (doublet of doublets), dt (doublet of triplets), m (multiplet). The number of protons, n, for a given resonance signal is indicated by nH. Coupling constants (*J*) are expressed in Hz and are recorded to the nearest 0.1 Hz. Identical proton coupling constants (*J*) are averaged in each spectrum and reported to the nearest 0.1 Hz. MestReNova and TopSpin software were used for NMR analysis. Spectra were assigned using COSY, HSQC, and HMBC experiments, as necessary. Bruker TopSpin software was used to plot the spectra.

^{13}C NMR spectra were recorded using a Bruker AVH400 (101 MHz), Bruker AVC500 (126 MHz) or Bruker NEO600 (151 MHz) spectrometers in the stated solvents, with broadband proton decoupling using the stated solvent as a reference for internal deuterium lock. The chemical shift data for each signal are quoted as δ_C in parts per million (ppm) relative to the tetramethyl silane (TMS) where δ_H (TMS) is 0.00 ppm. The shift values of resonances are quoted to 1 decimal place and were determined using MestReNova and TopSpin software. Spectra were assigned using COSY, HSQC, and HMBC experiments as necessary. Bruker TopSpin software was used to plot the spectra.

Mass spectra were recorded using either an Agilent 6120 (low resolution) or a Bruker microToF (high resolution) spectrometer using electrospray ionisation (ESI). Samples were submitted as solutions in either methanol or acetonitrile. *m/z* values are given in Daltons (Da) and followed by their percentage abundance in parentheses.

Melting points were obtained using a Griffin capillary tube melting apparatus and are uncorrected. The crystallisation solvent is given in parentheses.

Specific optical rotations were measured using a Schmidt Haensch Unipol polarimeter, using a sodium lamp at 589 nm and a path length of 1.0 dm. The concentration (*c*) is expressed in g/100 mL (equivalent to g/0.1 dm^3). Specific rotations are denoted $[\alpha]_D^T$ and are given in implied units of 10^{-1} deg cm^2g^{-1} (where T = ambient temperature in °C).

Infrared (IR) spectra were obtained from neat samples. The spectra were recorded using a Bruker Tensor 27 spectrometer with a diamond ATR module. Absorption maxima are given in wavenumbers (cm^{-1}) and reported as s (strong), m (medium), or w (weak).

Analytical thin layer chromatography (TLC) was carried out on normal phase Merck silica gel 60 F_{254} aluminium-supported thin layer chromatography sheets. Visualisation was achieved by absorption of UV light (λ_{max} 254 nm), or thermal development after staining in an aqueous solution of potassium permanganate. UV light was provided by a LF – 206 LS 230V – 50 Hz from UVltec Limited.

Flash column chromatography was carried out using Geduran® silica gel 60 (40–63 µm), eluting using solvents as supplied under a positive pressure of nitrogen.

Analytical HPLC was carried out using an Agilent 1260 Infinity II® system with a quaternary LC pump and UV/vis LC detector. For the determination of compound purity, one of the following methods was used: (A) Poroshell 120 EC-C18 column 40 (4 µM, 4.6 × 100 mm) and (B) Poroshell 120 EC-C18 column 40 (4 µM, 4.6 × 150 mm); Gradient elution of 95% H_2O/5% MeCN + 0.1% formic acid modifier (1 min), 95% H_2O/ 5% MeCN to 5% H_2O/ 95% MeCN + 0.1% FA modifier (over 10 min), hold (5 min); flow rate 1 mL/min and detection at 220, 254, 280, and 365 nm.

Semi-preparative high performance liquid chromatography (Semi Prep HPLC) was carried out using an Agilent 1260 Infinity II® system using one of the following methods: (A) 5 Prep C18 column (5 µm, 21.2 × 50 mm); Gradient elution: 95% H_2O/5% MeCN (1 min), 95% H_2O/ 5% MeCN to 5% H_2O/ 95% MeCN (over 9 min), hold (1 min); flow rate 25 mL/min;

(B) Zorbax SB-C18 column (5 µm, 9.4 × 250 mm); Gradient elution: 95% H_2O/5% MeCN (1 min), 95% H_2O/ 5% MeCN to 5% H_2O/ 95% MeCN (over 10 min), hold (5 min); flow rate 4 mL/min.

Synthetic Schemes

The 2-nitroimidazol (**23** and **24**) and indolequinone (**31**) precursors were synthesized following optimized protocols used in the group,[1,2] which are summarized in Scheme S1 and S2.

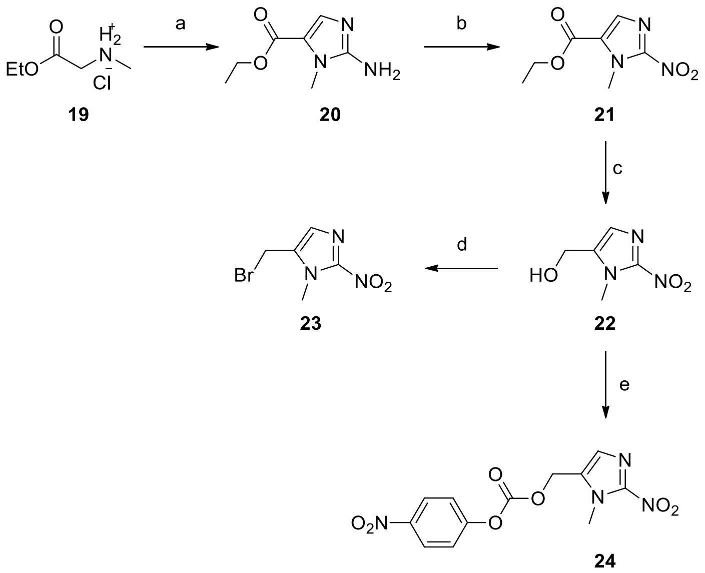

Scheme S1. Synthesis of 5-(bromomethyl)-1-methyl-2-nitro-1*H*-imidazole **23** and (1-methyl-2-nitro-1*H*-imidazol-5-yl)methyl (4-nitrophenyl) carbonate **24**. *Reagents and conditions:* (a) (1) NaH (60% in mineral oil), ethyl formate, dry THF, absolute EtOH, 0 ºC to rt, 18 h; (2) HCl (37% aq.), EtOH, 78 °C, 2 h; (3) $NCNH_2$, EtOH, water, 100 °C, 2 h, 50–68% (over three steps), $n = 3$; (b) $NaNO_2$, acetic acid, water, 0 °C to rt, 2 h, 42–53%, $n = 3$; (c) $NaBH_4$, CH_3OH, dry THF, 0 °C, 2 h, 35–40%, $n = 2$; (d) $SOBr_2$, 0 °C to rt, 20 min, 80–81%, $n = 2$; (e) 4-Nitrophenyl chloroformate, DMAP, dry pyridine, dry CH_2Cl_2, rt, 20 h, 68%.[1]

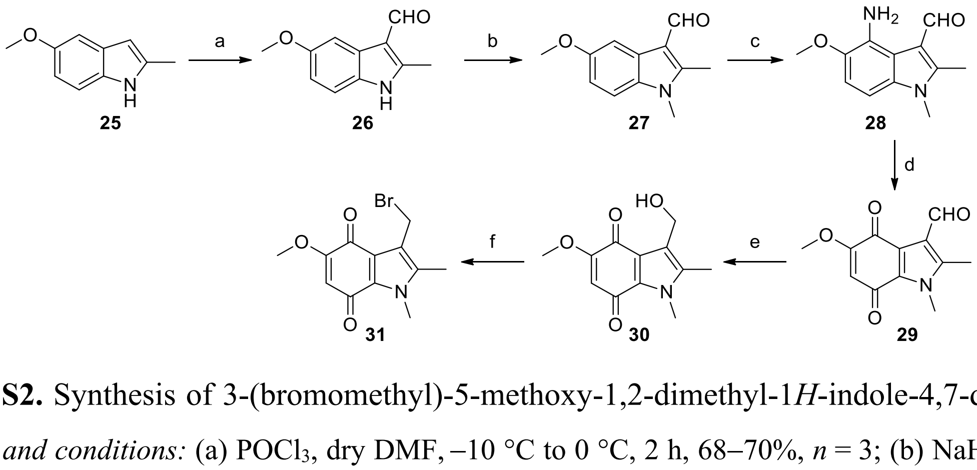

Scheme S2. Synthesis of 3-(bromomethyl)-5-methoxy-1,2-dimethyl-1*H*-indole-4,7-dione **31**. *Reagents and conditions:* (a) $POCl_3$, dry DMF, –10 °C to 0 °C, 2 h, 68–70%, $n = 3$; (b) NaH (60% in mineral oil), CH_3I, dry DMF, 0 ºC to rt, 4 h; 41–90%, $n = 3$; (c) 1. Fuming nitric acid, acetic acid, 0 °C, 4 h, 53–81%, $n = 3$; 2. Sn, HCl (3 M aq.), EtOH, 80 °C, 1 h, 71–90%, $n = 3$; (d) Fremy's salt,

NaH_2PO_4/Na_2HPO_4 buffer, acetone, rt, 1 h, 50–89%, *n* = 3; (e) $NaBH_4$, dry THF, dry CH_3OH, 0 °C, 1 h, 69–88%, *n* = 3; (f) PBr_3, dry CH_2Cl_2, 0 °C, 10 min, 90–99%, *n* = 3.[2]

The VH032 derivative **1** was synthesized according to Scheme S3. Compound **32** was treated with 4-nitrobenzyl bromide in the presence of Ag_2O and the methyl ester hydrolysed with LiOH to afford intermediate **33**. Boc deprotection of the intermediate **36**, followed by coupling reaction with **33** afforded the intermediate **37**, which was deprotected and coupled with *N*-acetyl-L-*tert*-leucine to afford the desired final compound **1**.

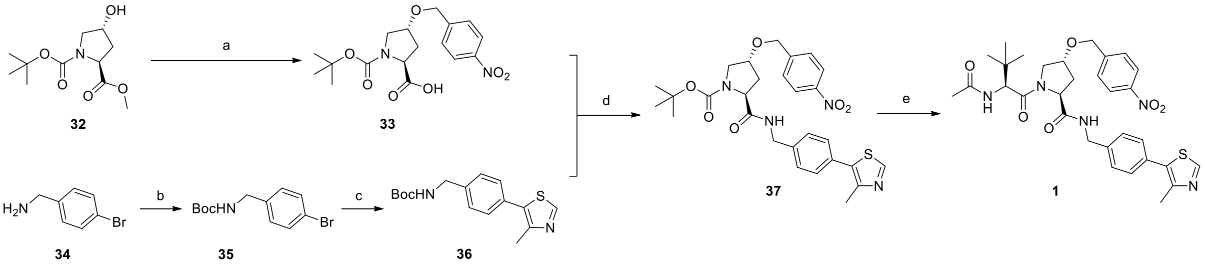

Scheme S3. Synthesis of VHL derivative **1**. *Reagents and conditions:* (a) (1) 4-Nitrobenzyl bromide, Ag_2O, dry CH_2Cl_2, 16 h, 99%; (2) LiOH, THF, MeOH, H_2O, rt, 1.5 h, 99%, *n* = 2; (b) Boc_2O, $NaHCO_3$, H_2O, EtOAc, 0 °C to rt, 2 h, 97%; (c) 4-Methylthiazole, KOAc, $Pd(OAc)_2$, dry DMA, 90 °C, 24 h, 48%; (d) (1) HCl (4 M in dioxane), CH_2Cl_2, rt, 2 h; (2) PyBOP, Et_3N, THF, rt, 22 h, 35–50% over two steps, *n* = 2; (e) (1) HCl (4 M in dioxane), CH_2Cl_2, rt, 2 h; (2) *N*-acetyl-L-*tert*-leucine, HATU, DIPEA, dry DMF, rt, 16 h, 35% over two steps.

The VHL derivative **4** was synthesized according to Scheme S4. To obtain the VHL ligand **41**, a synthetic protocol reported by Han *et al.*[3] was followed. The intermediate was then activated using 4-nitrophenyl chloroformate before being treated with compound **22** to afford the desired compound **4**.

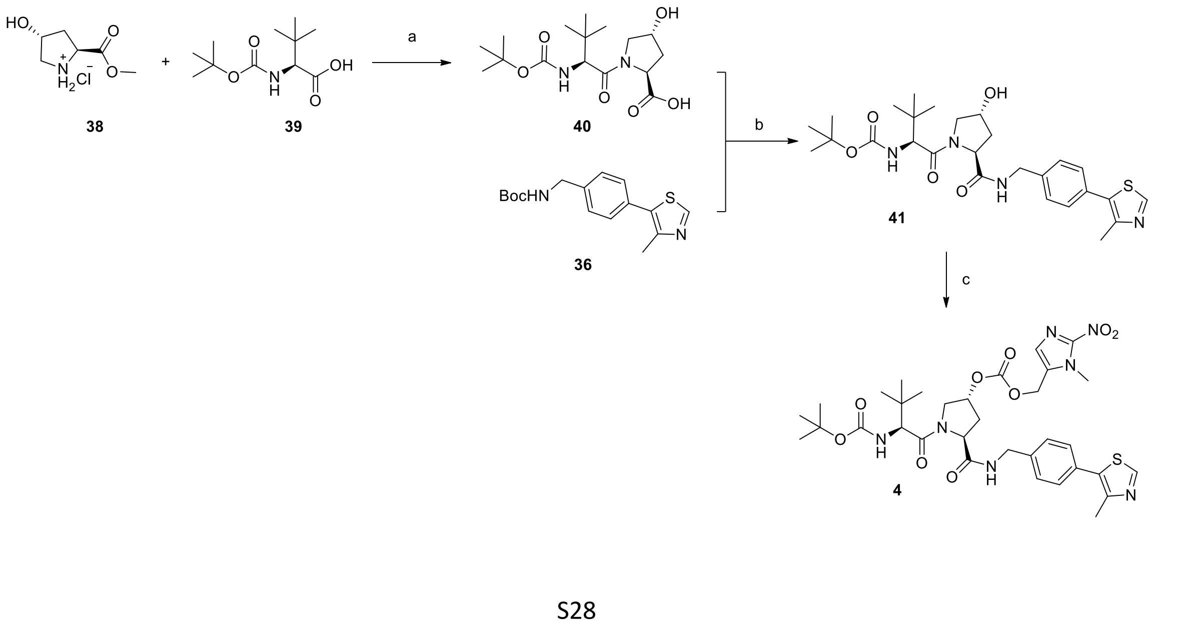

Scheme S4. Synthesis of VHL derivative **4**. *Reagents and conditions:* (a) (1) HATU, DIPEA, dry DMF, rt, 16 h, 86%; (2) LiOH, H_2O, THF, rt, 16 h, 83–100%, *n* = 2; (b) (1) HCl (4 M in dioxane), CH_2Cl_2, rt, 2 h; (2) HATU, DIPEA, dry DMF, rt, 16 h, 46% over two steps; (c) (1) 4-Nitrophenyl chloroformate, Et_3N, dry CH_2Cl_2, rt, 1 h, 25–40%, *n* = 2; (2) Compound **22**, DMAP, dry DMF, rt, 18 h, 35–55%, *n* = 2.

The VHL-recruiting PROTAC **8** and HAP-TACs **6**, **7** and **9** were synthesized as described in Scheme S5. Compound **41** was coupled to the commercially available PEG_3 linker to afford intermediate **42**. Subsequent reduction of the azide **42**, using Pd-catalyzed hydrogenation, gave the amine which was coupled with the commercially available carboxylic acid derivative of the BET bromodomain ligand (+)-JQ1 ((+)-JQ1-COOH) to afford MZ1 (**8**). Reaction of **8** with (1-methyl-2-nitro-1*H*-imidazol-5-yl)methyl (4-nitrophenyl) carbonate **24**, synthesized as described in Scheme S1, afforded compound **6** in a late-stage functionalization step, as previously reported by Shi *et al.*[4] Alternatively, alkylation of the hydroxyl group of compound **42**, using either indolequinone bromide (**31**) or commercially available benzyl bromide in the presence of TBAI afforded compounds **43** and **44**. A Staudinger reduction of these azides to give the corresponding amine, followed by (+)-JQ1-COOH coupling, afforded the final compounds **7** and **9**.

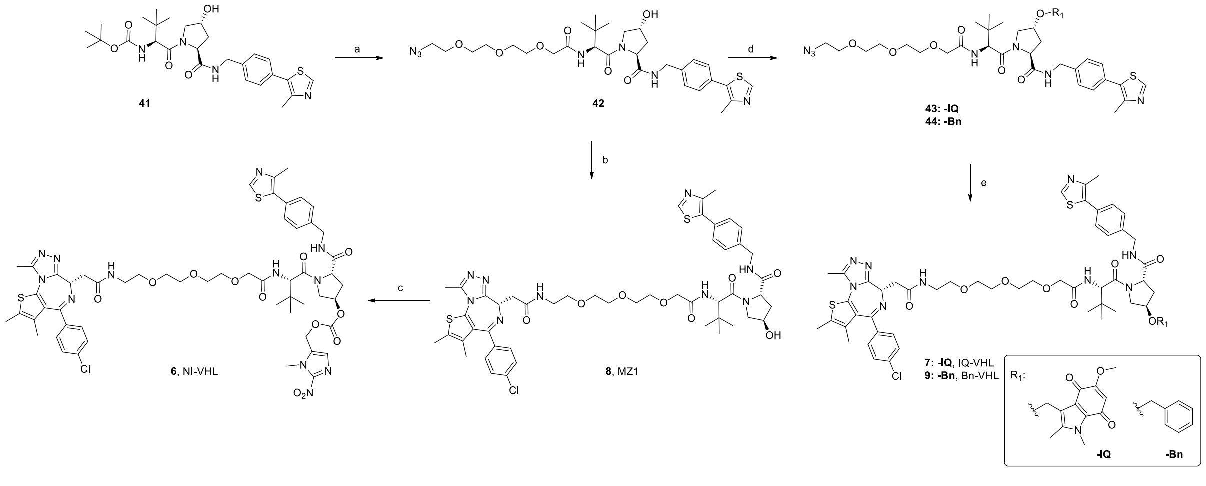

Scheme S5. Synthesis of VHL-recruiting PROTAC **8** and HAP-TACs **6**, **7** and **9**. *Reagents and conditions:* (a) (1) HCl (4 M in dioxane), CH_2Cl_2, rt, 2 h; (2) 2-(2-(2-(2-Azidoethoxy)ethoxy)ethoxy)acetic acid, HATU, DIPEA, dry DMF, rt, 16 h, 41–63% over two steps, *n* = 4; (b) (1) Pd/C 10%, H_2, CH_3OH, rt, 4 h; (2) (+)-JQ1-COOH, HATU, DIPEA, dry DMF, rt, 16 h, 66% over two steps; (c) Compound **24**, dry Et_3N, dry CH_2Cl_2, 40 °C, 40 h, 45%; (d) R_2-Br, TBAI, NaOH, H_2O, CH_2Cl_2, rt, 3 h, 42–56%, *n* = 3 for **43**; 3 h, 81% for **44**; (e) (1) PPh_3, H_2O, THF, rt, 16 h;

(2) (+)-JQ1-COOH, HATU, DIPEA, dry DMF, rt, 16 h, 36–50% over two steps, *n* = 2 for **7**; 16 h, 72% over two steps for **9**.

NB- (**10**) and NI-POMA (**12**) were obtained as summarized in Scheme S6. From pomalidomide **11**, a Mitsunobu reaction with polymer-supported PPh_3 (PS-PPh_3) and DIAD in a 4:1 mixture of dry THF and dry DMF gave the products. Both compounds were obtained in 20% of yield.

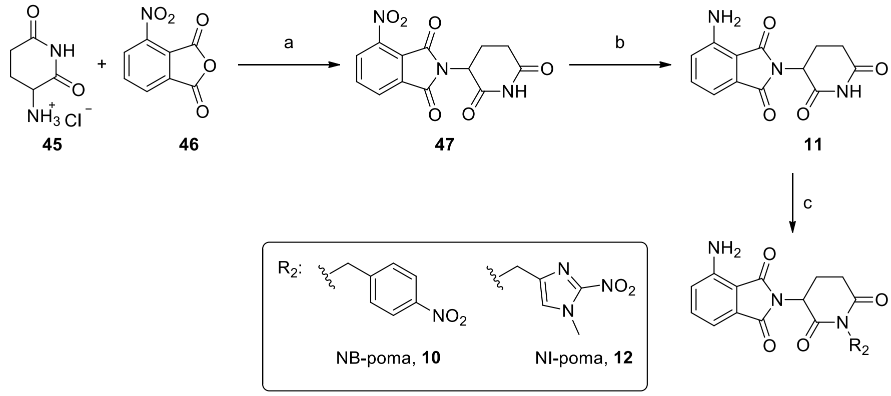

Scheme S6. Synthesis of pomalidomide **11** and pomalidomide derivatives **10** and **12**. *Reagents and conditions:* (a) Sodium acetate, acetic acid, 120 °C, 3–7 h, 70–88%, *n* = 3; (b) Pd/C 10%, H_2, DMF, rt, 24 h, 91%; (c) R_1-OH, PS-PPh_3, DIAD, dry THF, dry DMF, 0 °C to rt for **10**, 36 h, 20% and 0 °C to 60 °C for **12**, 20 h, 20%.

To synthesize the CRBN-recruiting HAP-TACs, we followed a previously reported route[5] to obtain compound **55** (Scheme S7). The Boc group of **55** was deprotected using HCl (4 M in dioxane) and a HATU-mediated amide coupling with (+)-JQ1-COOH afforded the active PROTAC PG-4c (**17**, Scheme S7). To obtain the protected PROTACs **15, 16** and **18**, alkylation of the glutarimide nitrogen with the 2-nitroimidazol bromide (**23**), indolequinone bromide (**31**), or benzyl bromide in the presence of Cs_2CO_3 and TBAI in DMF gave compounds **56-58**. Treatment of **58** with HCl (4 M in dioxane, 6 eq.), in an attempt to remove the Boc group from the terminal amine, revealed that the glutarimide ring is acid labile and opens under the strongly acidic conditions. When the intermediate was carried forward to an amide coupling to (+)-JQ1 carboxylic acid, the corresponding product was only formed in poor yield. To overcome this issue, we employed the milder combination of oxalyl chloride in methanol to remove the Boc group. Although the deprotection required 16 h, the reactions proceeded smoothy, forming one product, as judged by TLC analysis, and after evaporation and amide coupling with (+)-JQ1-COOH, the products **15, 16** and **18** were obtained in yields 47–84%.

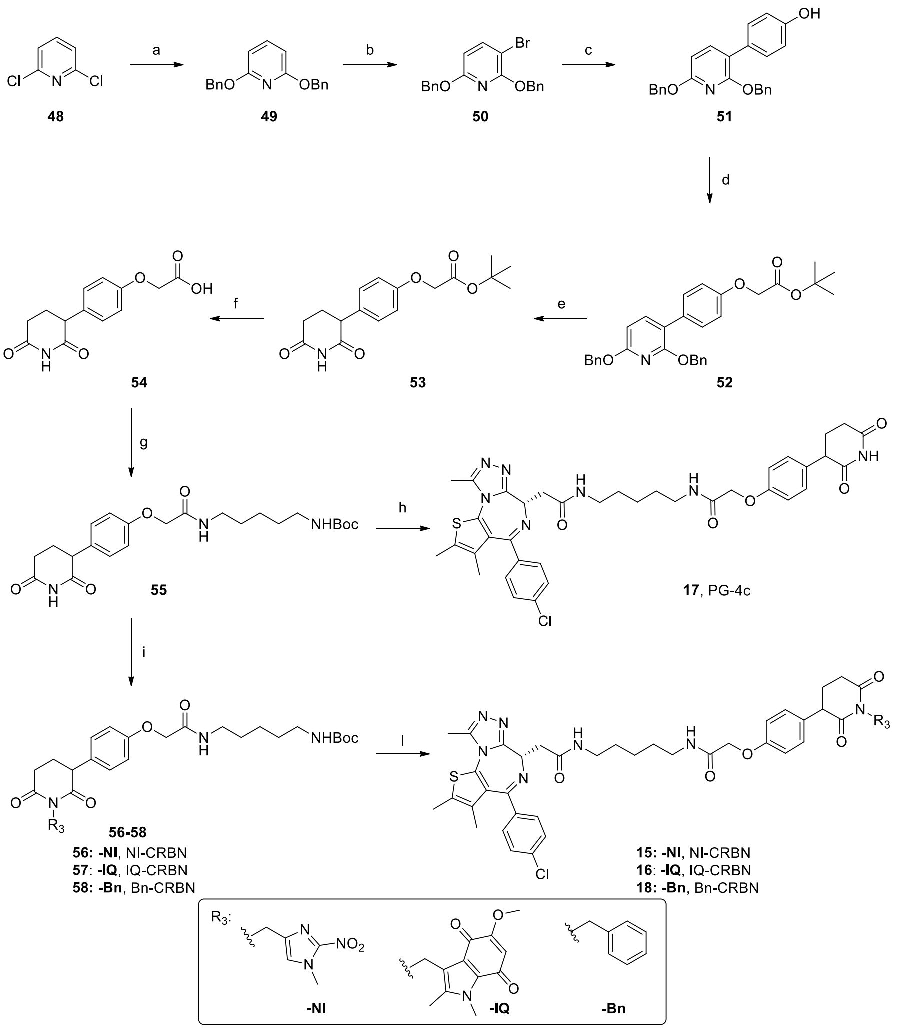

Scheme S7. Synthesis of CRBN-recruiting active PROTAC **17** and HAP-TACs **15**, **16**, **18**. *Reagents and conditions:* (a) Benzyl alcohol, NaH (60% in mineral oil), dry DMF, 0 °C to 80 °C, 16 h, 97–98%, *n* = 3; (b) NBS, CH_3CN, 80 °C, 3 h, 64–79%, *n* = 3; (c) (4-Hydroxyphenyl)boronic acid, K_3PO_4, Pd(dppf)Cl_2·CH_2Cl_2, dioxane, water, 100 °C, 16 h, 77–91%, *n* = 4; (d) *tert*-Butyl bromoacetate, K_2CO_3, dry DMF, rt, 3 h, 71–93%, *n* = 4; (e) Pd/C 10%, H_2, DMF, rt, 16 h, 56–92%, *n* = 3; (f) TFA, CH_2Cl_2, rt, 16 h, 90–99%, *n* = 4; (g) *N*-Boc-cadaverine, HATU, DIPEA, dry DMF, rt, 16 h, 49–85%, *n* = 4; (h) (1) HCl (4 M in dioxane), CH_2Cl_2, rt, 1 h; (2) (+)-JQ1-COOH, HATU, DIPEA, dry DMF, rt, 16 h, 86%; (i) R_1-Br, Cs_2CO_3, TBAI, DMF, rt, 22 h, 39–46%, *n* = 2 for **56**; 24 h, 23–32%, *n* = 3 for **57**; 32 h, 40–41%, *n* = 2 for **58**; (l) (1)Oxalyl chloride, MeOH, rt, 19 h; (2) (+)-JQ1-COOH, HATU, DIPEA, dry DMF, rt, 16 h, 36–50% over two steps, *n* = 2, for **15**, 24 h, 47–84% over two steps, *n* = 2 for **16**, 16 h, 66% over two steps for **18**.

We note that the protected intermediates 43-44 (Scheme S5) and 56-58 (Scheme S7) can potentially be applied to synthesize a wide variety of CRBN- or VHL-recruiting HAP-TACs by conjugation with the POI ligand of choice.

Synthetic procedures and compound data

Ethyl 2-amino-1-methyl-1*H*-imidazole-5-carboxylate (20)

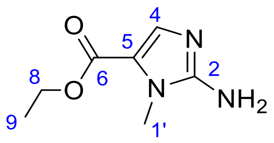

Sarcosine ethyl ester hydrochloride (6.00 g, 39.06 mmol, 1.0 eq.) was suspended in a combination of dry THF (135 mL), absolute EtOH (13 mL), and ethyl formate (135 mL) and cooled to 0 °C under a stream of argon. NaH (60% in mineral oil, 6.25 g, 156.24 mmol, 4.0 eq.) was added portion wise and, once gas evolution had ceased, the reaction mixture was warmed to rt and stirred for 18 h. The reaction was quenched by the addition of diethyl ether (200 mL) and evaporated *in vacuo*. The resulting solid was triturated with hexane (2 × 50 mL) and dried *in vacuo*. The resulting intermediate was then suspended in EtOH (130 mL) and concentrated HCl (26 mL) was slowly added to the suspension. The mixture was stirred under reflux for 2 h then filtered *in vacuo* to remove the colourless salts. The resulting solution was concentrated *in vacuo*, then dissolved in EtOH (210 mL) and water (90 mL), and the pH adjusted to 3 with 6 M aqueous NaOH solution (~50 mL). Cyanamide (3.27 g, 78.12 mmol, 2.0 eq.) was added to the solution and the reaction was heated at 100 °C for 2 h. The mixture was cooled to rt and concentrated *in vacuo* to reach ⅛ of the original volume, then solid K_2CO_3 was added until the pH reached 8-9. The resulting precipitate was filtered *in vacuo*, washed with saturated aqueous K_2CO_3 solution (1 × 20 mL) and water (1 × 20 mL) and dried *in vacuo* to yield a yellow solid (4.52 g, 68%): R_f 0.25 (CH_2Cl_2:CH_3OH 95:5); m.p. 145–147 °C (from water) [lit.[1] 130-133 °C]; ^1H NMR (400 MHz; $CDCl_3$) δ_H 7.37 (1H, s, H-4), 4.38 (2H, br s, N*H*2), 4.19 (2H, q, *J* 7.1, H-8), 3.60 (3H, s, H-1'), 1.27 (3H, t, *J* 7.1, H-9); LRMS *m/z* (ESI$^+$) 170 ([M+H]$^+$, 100%). The spectroscopic data are in good agreement with the literature values.[1]

Ethyl 1-methyl-2-nitro-1*H*-imidazole-5-carboxylate (21)

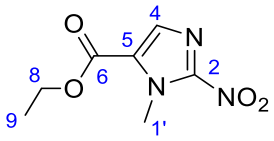

To a solution of $NaNO_2$ (18.40 g, 267.10 mmol, 10.0 eq.) in water (25 mL), intermediate **20** (4.52 g, 26.71 mmol, 1.0 eq.) in glacial acetic acid (47 mL) was added dropwise at 0 °C over a period of 45 min. The solution was stirred for 1 h at 0 °C, and then at rt for a further 2 h. The

mixture was then diluted with water (80 mL) and the product was extracted with CH_2Cl_2 (6 × 80 mL). The combined organic layers were washed with a saturated aqueous $Na_2S_2O_3$ solution (100 mL), dried over sodium sulfate, filtered, and evaporated. The residue was dissolved in CH_2Cl_2 and filtered through a short pad of silica gel, eluting with CH_2Cl_2, to yield a colourless solid (2.65 g, 50%): R_f 0.24 (CH_2Cl_2 100%); m.p. 52–54 °C (from CH_2Cl_2) [lit.[1] 56–58 °C]; ^{1}H NMR (400 MHz; $CDCl_3$) δ_H 7.75 (1H, s, H-4), 4.42 (2H, q, *J* 7.1, H-8), 4.37 (3H, s, H-1'), 1.43 (3H, t, *J* 7.1, H-9); LRMS *m/z* (ESI$^+$) 200 ([M+H]$^+$, 100%). The spectroscopic data are in good agreement with the literature values.[1]

(1-Methyl-2-nitro-1*H*-imidazol-5-yl)methanol (22)

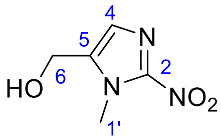

Intermediate **21** (2.60 g, 13.05 mmol, 1.0 eq.) was dissolved in a mixture of dry THF (45 mL) and CH_3OH (2.5 mL) and the reaction was cooled to 0 °C. $NaBH_4$ (1.48 g, 39.15 mmol, 3.0 eq.) was added portion wise, and the resulting mixture was stirred at 0 °C for 2 h. After this time, the mixture was quenched by dropwise addition of a solution of diethyl ether and CH_3OH (1:1, 100 mL in total) at 0 °C and stirred for 30 min. The mixture was gradually acidified to pH 5 by addition of 2 M aqueous HCl solution (10 mL), and concentrated *in vacuo* to give a mostly aqueous solution. The product was extracted with ethyl acetate (6 × 20 mL), then the aqueous phase was saturated with solid NaCl and further product extracted with EtOAc (6 × 20 mL). The combined organic layers were dried over sodium sulfate, filtered, and concentrated *in vacuo*. The crude material was purified using silica gel column chromatography, eluting with PE:EtOAc (gradient 30 to 100% EtOAc) to afford a pale yellow solid (824 mg, 40%): R_f 0.47 (EtOAc 100%); m.p. 118–120 °C (from EtOAc) [lit.[1] 141–143 °C]; ^{1}H NMR (400 MHz; $CDCl_3$) δ_H 7.19 (1H, s, H-4), 4.66 (2H, s, H-6), 4.02 (3H, s, H-1'); LRMS *m/z* (ESI$^+$) 158 ([M+H]$^+$, 100%). The spectroscopic data are in good agreement with the literature values.[1]

5-(Bromomethyl)-1-methyl-2-nitro-1*H*-imidazole (23)

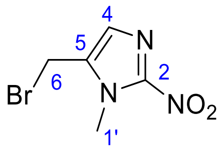

Under argon, intermediate **22** (200 mg, 1.27 mmol, 1.0 eq.) was dissolved in $SOBr_2$ (2.29 mL, 24.13 mmol, 19.0 eq.) at 0 °C and the resulting solution was warmed to rt and stirred for 20 min. The reaction was quenched by dropwise addition of ice-cold water (25 mL) and the product was extracted using EtOAc (3 × 25 mL). The combined organic layers were dried over sodium sulfate, filtered, and concentrated *in vacuo* to yield the title compound as a dark yellow solid (227 mg, 81%): R_f 0.61 (EtOAc 100%); m.p. 84–85 °C (from EtOAc); ^{1}H NMR (400 MHz; $CDCl_3$) δ_H 7.20 (1H, s, H-4), 4.47 (2H, s, H-6), 4.05 (3H, s, H-1'); LRMS *m/z* (ESI$^+$) 220 ([^{79}M+H]$^+$, 22%), 222 ([^{81}M+H]$^+$, 22%). The spectroscopic data are in good agreement with the literature values.[1]

(1-Methyl-2-nitro-1*H*-imidazol-5-yl)methyl (4-nitrophenyl) carbonate (24)

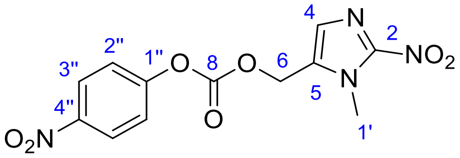

Under argon, intermediate **22** (100 mg, 0.64 mmol, 1.0 eq.) was dissolved in dry CH_2Cl_2 (8 mL) and dry pyridine (154 µL, 1.92 mmol, 3.0 eq.) was added. The solution was cooled to 0 °C and a solution of 4-nitrophenyl chloroformate (192 mg, 0.96 mmol, 1.5 eq.) in dry CH_2Cl_2 (2 mL) was added dropwise. The reaction mixture was warmed to rt and stirred for 4 h. Addition of 3.0 eq. of dry pyridine and 1.5 eq. of 4-nitrophenyl chloroformate was performed and the reaction was stirred at rt for 16 h. After this time, water (50 mL) was added, and the product was extracted with CH_2Cl_2 (1 × 50 mL). The organic layer was quickly washed with 1 M aqueous HCl solution (3 × 50 mL), then dried over sodium sulfate, filtered, and evaporated *in vacuo*. The crude material was purified using silica gel column chromatography, eluting with PE:EtOAc (gradient 25 to 60% EtOAc) to afford a pale pink solid (140 mg, 68%): R_f 0.35 (PE:EtOAc 50:50); m.p. 118–120 °C (from PE/EtOAc); $\tilde{\upsilon}_{max}$ (neat)/cm^{-1} 2919 (C-H, w), 2850 (C-H, w), 1769 (C=O, s), 1524 (N-O, w), 1491 (w), 1336 (N-O, w), 1215 (C-C-O, s), 861 (C-H, w), 837 (C-H, w); ^{1}H NMR (600 MHz; D_6-DMSO) δ_H 8.33 (2H, d, *J* 9.2, H-3"), 7.58 (2H, d, *J* 9.2, H-2"), 7.36 (1H, s, H-4), 5.46 (2H, s, H-6), 3.98 (3H, s, H-1'); ^{13}C NMR (151 MHz; D_6-DMSO) δ_C 155.6 (C-1"), 151.9 (C-8), 145.8 (2C, C-4", C-5), 131.9 (C-2), 130.0 (C-4),

125.9 (C-3''), 123.0 (C-2''), 59.9 (C-6), 34.8 (C-1'); HRMS *m/z* (ESI$^+$) [Found: 323.0619, $C_{12}H_{11}N_4O_7$ requires $[M+H]^+$ 323.0622]; LRMS *m/z* (ESI$^+$) 304 (100%), 323 ($[M+H]^+$, 12%); HPLC Retention time 8.4 min, 94.2%. The spectroscopic data are in good agreement with the literature values.[6]

5-Methoxy-2-methyl-1*H*-indole-3-carbaldehyde (26)

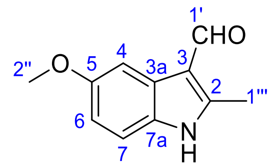

$POCl_3$ (4.05 mL, 43.4 mmol, 1.4 eq.) was slowly added to dry DMF (10 mL) at 0 °C and the resulting solution was stirred at 0 °C for 10 min. The resulting mixture was then added dropwise to a solution of 5-methoxy-2-methyl-1*H*-indole (5.00 g, 31.00 mmol, 1.0 eq.) in dry DMF (10 mL) at –10 °C over a period of 45 min. The reaction mixture was then stirred at 0 °C for 1 h and then quenched by dropwise addition of an ice-cold 2 M aqueous NaOH solution (50 mL). The reaction mixture was extracted with CH_2Cl_2 (5 × 50 mL). The combined organic layers were dried over sodium sulfate, filtered, and evaporated *in vacuo*. The resulting brown solid was washed with cold EtOAc (2 × 50 mL), and filtration *in vacuo* yielded the product as a beige solid (3.99 g, 68%): R_f 0.26 (PE/EtOAc 40:60); m.p. 180–182 °C (from EtOAc) [lit.[7] 198–199 °C, lit.[8] 191–194 °C, lit.[9] 120–122 °C]; ^{1}H NMR (400 MHz; $CDCl_3$) δ_H 10.07 (1H, s, H-1'), 7.73 (1H, d, *J* 2.5, H-4), 7.23 (1H, d, *J* 8.8, H-7), 6.84 (1H, dd, *J* 8.8, 2.5, H-6), 3.86 (3H, s, H-2''), 2.70 (3H, s, H-1'''); LRMS *m/z* (ESI$^+$) 190 ($[M+H]^+$, 68%), 401 (100%). The spectroscopic data are in good agreement with the literature values.[7–9]

5-Methoxy-1,2-dimethyl-1*H*-indole-3-carbaldehyde (27)

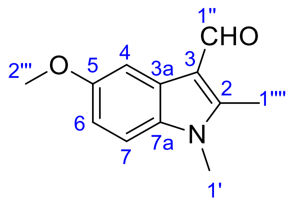

Under argon, a solution of 5-methoxy-2-methyl-1*H*-indole-3-carbaldehyde **26** (1.32 g, 6.98 mmol, 1.0 eq.) was dissolved in dry DMF (9 mL) and NaH (60% mineral oil, 0.42 g, 10.47 mmol, 1.5 eq.) was added portion wise. Once gas evolution had ceased, the reaction mixture was warmed to rt and stirred for 2 h. After this time the reaction mixture was cooled to 0 °C, and iodomethane (0.52 mL, 8.38 mmol, 1.2 eq.) was added dropwise. The resulting

mixture was warmed to rt and stirred for 2 h. The reaction was then quenched by slow addition of water (50 mL). The mixture was extracted with CH_2Cl_2 (6 × 50 mL). The combined organic layers were dried over sodium sulfate, filtered, and concentrated *in vacuo*. The crude material was purified using silica gel column chromatography, eluting with PE:EtOAc (gradient 40 to 100% EtOAc) to afford compound **27** as a beige solid (1.27 g, 90%): R_f 0.26 (PE:EtOAc 40:60); m.p. 124–126 °C (from PE/EtOAc) [lit.[10] 132–133 °C]; ^{1}H NMR (400 MHz; $CDCl_3$) δ_H 9.82 (1H, s, H-1"), 7.73 (1H, d, *J* 2.5, H-4), 7.24 (1H, d, *J* 8.8, H-7), 6.95 (1H, dd, *J* 8.8, 2.5, H-6), 3.89 (3H, s, H-2'''), 3.74 (3H, s, H-1'), 2.69 (3H, s, H-1''''); LRMS *m/z* (ESI$^+$) 204 ([M+H]$^+$, 79%), 226 ([M+Na]$^+$, 100%). The spectroscopic data are in good agreement with the literature values.[10]

4-Amino-5-methoxy-1,2-dimethyl-1*H*-indole-3-carbaldehyde (28)

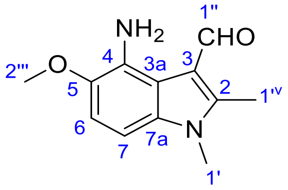

Step 1:

A solution of fuming nitric acid (11.3 mL, 279.90 mmol, 22.5 eq.) in acetic acid (46 mL) was added dropwise over 1 h to a solution of 5-methoxy-1,2-dimethyl-1*H*-indole-3-carbaldehyde **27** (2.53 g, 12.44 mmol, 1.0 eq.) in acetic acid (290 mL) at 0 °C. The reaction mixture was stirred for 3 h, then crushed ice-water (400 g) was added, and the resulting suspension was stirred at rt for 1 h. The resulting yellow precipitate was collected by vacuum filtration, washed with water (1 × 20 mL), and dried *in vacuo* to afford the crude material as a pale yellow solid (1.94 g, 63%), which was used in the next step without further purification.

Step 2:

The intermediate obtained in Step 1 (1.94 g, 7.82 mmol, 1.0 eq.) was dissolved in ethanol (146 mL), and powdered tin (4.87 g, 41.05 mmol, 5.25 eq.) was added followed by a 3 M aqueous HCl solution (60 mL). The reaction mixture was heated to 80 °C for 1 h. Then, water (400 mL) was added, and the pH adjusted to 8 with solid $NaHCO_3$. The aqueous layer was extracted with $CHCl_3$ (3 × 500 mL) and the combined organic phases were dried over sodium sulfate, filtered, and concentrated *in vacuo* to yield the title compound as a brown-yellow solid (1.54 g, 90%): R_f 0.60 (100% EtOAc); m.p. 155–157 °C (from $CHCl_3$); ^{1}H NMR (400 MHz; $CDCl_3$) δ_H 9.83 (1H, s, H-1"), 6.88 (1H, d, *J* 8.6, H-7), 6.50 (1H, d, *J* 8.6, H-6), 3.89 (3H, s, H-

2'''), 3.62 (3H, s, H-1'), 2.63 (3H, s, H-1^{v}); LRMS m/z (ESI$^+$) 219 ([M+H]$^+$, 100%). The spectroscopic data are in good agreement with the literature values.[10]

5-Methoxy-1,2-dimethyl-4,7-dioxo-4,7-dihydro-1*H*-indole-3-carbaldehyde (29)

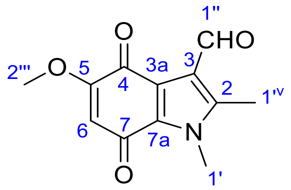

4-Amino-5-methoxy-1,2-dimethyl-1*H*-indole-3-carbaldehyde (1.53 g, 7.01 mmol, 1.0 eq.) was dissolved in acetone (450 mL) and a solution of Fremy's salt (9.40 g, 35.05 mmol, 5.0 eq.) in NaH_2PO_4/Na_2HPO_4 buffer (450 mL, 0.4 M, pH 6.0) was added. The resulting mixture was stirred at rt for 1 h, then acetone was removed *in vacuo* and the compound was extracted with EtOAc (6 × 400 mL). The combined organic layers were dried over sodium sulfate, filtered, and evaporated *in vacuo*. Purification using silica gel column chromatography, eluting with acetone:CH_2Cl_2 (gradient 2 to 10% CH_2Cl_2) yielded the title compound as an orange-red solid (1.23 g, 75%): R_f 0.65 (100% EtOAc); m.p. 236–238 °C (from acetone/CH_2Cl_2) [lit.[9] 239–242 °C]; ^{1}H NMR (400 MHz; $CDCl_3$) δ_H 10.55 (1H, s, H-1''), 5.70 (1H, s, H-6), 3.94 (3H, s, H-1'), 3.85 (3H, s, H-2'''), 2.62 (3H, s, H-1^{iv}); LRMS m/z (ESI$^+$) 234 ([M+H]$^+$, 100%), 256 ([M+Na]$^+$, 75%). The spectroscopic data are in good agreement with the literature values.[10]

3-(Hydroxymethyl)-5-methoxy-1,2-dimethyl-1*H*-indole-4,7-dione (30)

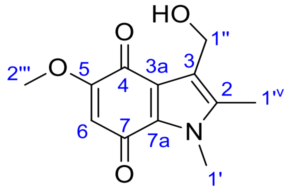

$NaBH_4$ (0.99 g, 26.15 mmol, 5.0 eq.) was added portion wise to a suspension of 5-methoxy-1,2-dimethyl-4,7-dioxo-4,7-dihydro-1*H*-indole-3-carbaldehyde (1.22 g, 5.23 mmol, 1.0 eq.) in dry THF (100 mL) and dry CH_3OH (100 mL) at 0 °C, under argon. The mixture was stirred at 0 °C for 45 min and then water (100 mL) was added slowly. The compound was then quickly extracted with CH_2Cl_2 (4 × 100 mL). The combined organic layers were dried over sodium sulfate, filtered, and concentrated *in vacuo*. Purification using silica gel column chromatography, eluting with PE:EtOAc (gradient 50 to 100% EtOAc) yielded the title compound as a red solid (1.08 g, 88%): R_f 0.47 (100% EtOAc); m.p. 198–200 °C (from

PE/EtOAc) [lit.[11] 199–200 °C]; ^{1}H NMR (400 MHz; CD_3OD) δ_H 5.61 (1H, s, H-6), 4.71 (2H, s, H-1''), 3.90 (3H, s, H-1'), 3.81 (3H, s, H-2'''), 2.29 (3H, s, H-1^{iv}); LRMS *m/z* (ESI$^+$) 258 ([M+Na]$^+$, 100%). The spectroscopic data are in good agreement with the literature values.[10,11]

3-(Bromomethyl)-5-methoxy-1,2-dimethyl-1*H*-indole-4,7-dione (31)

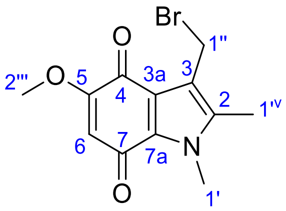

3-(Hydroxymethyl)-5-methoxy-1,2-dimethyl-1*H*-indole-4,7-dione (0.5 g, 2.12 mmol, 1.0 eq.) was dissolved in dry CH_2Cl_2 (22 mL) under argon. The reaction mixture was cooled to 0 °C and PBr_3 (403 µL, 4.24 mmol, 2.0 eq.) was added dropwise. After 10 min, the reaction was complete and was quenched by slow addition of saturated aqueous $NaHCO_3$ solution (30 mL). The product was extracted with CH_2Cl_2 (3 × 30 mL) and the combined organic phases were dried over sodium sulfate, filtered, and evaporated *in vacuo*, yielding the title compound as a dark red solid (0.61 g, 97%): R_f 0.25 (PE/EtOAc 60:40); m.p. 190–192 °C (from CH_2Cl_2); ^{1}H NMR (400 MHz; $CDCl_3$) δ_H 5.63 (1H, s, H-6), 4.78 (2H, s, H-1''), 3.90 (3H, s, H-1'), 3.81 (3H, s, H-2'''), 2.26 (3H, s, H-1^{iv}); LRMS *m/z* (ESI$^-$) 296 ([^{79}M-H]$^-$, 24%), 298 ([^{81}M-H]$^-$, 24%). The spectroscopic data are in good agreement with the literature values.[12]

(2*S*,4*R*)-1-(*tert*-Butoxycarbonyl)-4-((4-nitrobenzyl)oxy)pyrrolidine-2-carboxylic acid (33)

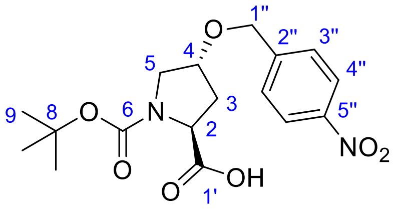

Step 1:

The reaction was carried out in the dark in a flame-dried flask under an atmosphere of argon. Freshly synthesised Ag_2O (1.31 g, 5.67 mmol, 3 eq.) was added to a solution of (2*S*,4*R*)-1-*tert*-butyl 2-methyl 4-hydroxypyrrolidine-1,2-dicarboxylate (0.52 g, 1.89 mmol, 1 eq.), 4-nitrobenzyl bromide (0.82 g, 3.78 mmol, 2 eq.) and powdered molecular sieves (3 Å) in dry CH_2Cl_2 (15 mL). The reaction mixture was stirred at rt for 16 h. The suspension was filtered through CeliteR, rinsing with CH_2Cl_2 (15 mL) and concentrated *in vacuo*. The resulting oil was purified by silica gel column chromatography, eluting with PE/EtOAc (gradient 20 to 40%

EtOAc) to afford (2*S*,4*R*)-1-*tert*-butyl 2-methyl 4-((4-nitrobenzyl)oxy)pyrrolidine-1,2-dicarboxylate as an off-white solid (0.71 g, 99%).

Step 2:

A solution of LiOH (0.13 g, 5.26 mmol, 10 eq.) in H_2O (3 mL) was added dropwise to a solution of the intermediate obtained in Step 1 (0.20 g, 0.53 mmol, 1 eq.) in THF (3 mL) and MeOH (3 mL). After 1.5 h, the reaction was concluded. The solution was acidified with 2 M aqueous HCl solution (5 mL) and extracted with CH_2Cl_2 (2 × 15 mL). The combined organic layers were washed with brine (15 mL), dried over sodium sulfate, filtered, and concentrated *in vacuo* to yield the title product as a colourless solid (0.19 g, 99%): *Rf* 0.70 (EtOAc - MeOH - acetone - H_2O 4:1:1:1); m.p. 38–40 °C (from CH_2Cl_2); $[\alpha]_D^{25} = -47.8$ (*c* 1.2, $CHCl_3$); $\tilde{\upsilon}_{max}$ (thin film)/cm^{-1} 2980 (C-H, w), 1725 (C=O, s), 1680 (C=O, s), 1521 (N-O, w), 1418 (C-H, w), 1346 (N-O, s); ^{1}H NMR (500 MHz; $CDCl_3$) δ_H 8.20 (2H, d, *J* 8.6, H-3"), 7.48 (2H, d, *J* 8.6, H-2"), 4.64 (1H, d, *J* 13.0, H-1"a), 4.59 (1H, d, *J* 13.0, H-1"b), 4.49 (1H, dd, J_s 7.3, 7.3, H-2), 4.24–4.19 (1H, m, H-4), 3.79–3.56 (2H, m, H-5a, H-5b), 2.62–2.15 (2H, m, H-3a, H-3b), 1.47 (9H, s, H-9); ^{13}C NMR (126 MHz, $CDCl_3$) δ_C 178.5 (C-1'), 156.4 (C-6), 147.6 (C-5"), 145.3 (C-2"), 127.7 (C-3"), 123.9 (C-4"), 82.1 (C-8), 77.4 (C-4), 70.1 (C-1"), 57.9 (C-2), 52.0 (C-5), 34.6 (C-3), 28.5 (C-9); HRMS *m/z* (ESI$^+$) [Found: 389.1319, $C_{17}H_{22}N_2O_7Na$ requires $[M+Na]^+$ 389.1319]; LRMS *m/z* (ESI$^-$) 1098 ($[3M-H]^-$, 100%). HPLC Retention Time 9.0 min, 96.1%.

***tert*-Butyl 4-bromobenzylcarbamate (35)**

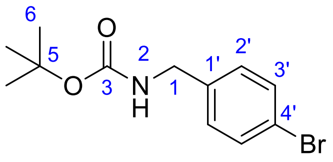

To a solution of (4-bromophenyl)methanamine (3.39 mL, 26.87 mmol, 1.0 eq.) and $NaHCO_3$ (1.67 g, 19.88 mmol, 0.74 eq.) in water (50 mL) and EtOAc (50 mL), was added Boc_2O (7.04 g, 32.24 mmol, 1.2 eq.) at 5 °C. The reaction mixture was stirred at rt for 2 h, then the aqueous layer was extracted with EtOAc (2 × 50 mL). The combined organic layers were dried over sodium sulfate, filtered, and evaporated *in vacuo* to obtain a colourless solid. This solid was suspended in hexane (20 mL) and the resulting suspension was stirred at 0 °C for 30 min, then filtered *in vacuo* to yield the title compound as a colourless solid (7.47 g, 97%): R_f 0.58 (PE/EtOAc 80:20); m.p. 80–82 °C (from PE/EtOAc) [lit.[13] 86–88 °C]; $\tilde{\upsilon}_{max}$ (neat)/cm^{-1} 3316 (N-H, w), 2979 (C-H, w), 1679 (C=O, s), 1535 (N-C=O, s), 1281 (C-O, s), 1166 (s), 1059 (w), 681 (C-H, s); ^{1}H NMR (400 MHz; $CDCl_3$) δ_H 7.44 (2H, d, *J* 8.5, H-3'), 7.15 (2H, d, *J* 8.5, H-

2'), 4.86 (1H, br s, N*H*-2), 4.26 (2H, d, *J* 6.0, H-1), 1.45 (9H, s, H-6); ^{13}C NMR (101 MHz; $CDCl_3$) δ_C 155.7 (C-3), 138.0 (C-1'), 131.7 (C-3'), 129.2 (C-2'), 121.1 (C-4'), 79.7 (C-5), 44.0 (C-1), 28.3 (C-6); LRMS *m/z* (ESI$^+$) 307 ([^{79}M+Na]$^+$, 38%), 309 ([^{81}M+Na]$^+$, 38%); HPLC Retention time 10.2 min, 98.3%. The spectroscopic data are in good agreement with the literature values.[13]

***tert*-Butyl 4-(4-methylthiazol-5-yl)benzylcarbamate (36)**

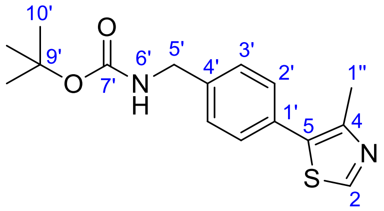

4-Methylthiazole (2.4 mL, 26.21 mmol, 1.5 eq.) was added to a solution of *tert*-butyl 4-bromobenzylcarbamate (5.00 g, 17.47 mmol, 1.0 eq.), KOAc (3.43 g, 34.94 mmol, 2.0 eq.) and $Pd(OAc)_2$ (39 mg, 0.17 mmol, 0.01 eq.) in dry DMA (12.5 mL) under argon. The reaction mixture was stirred for 24 h at 90 °C, cooled to rt, and concentrated *in vacuo*. Water (100 mL) was added, and the mixture was extracted with EtOAc (3 × 80 mL). The combined organic phases were washed with 0.5 M aqueous LiCl solution (1 × 200 mL), dried over sodium sulfate, filtered, and concentrated *in vacuo*. Purification using silica gel column chromatography, eluting with PE:EtOAc (gradient 10 to 30% EtOAc) yielded the title compound as a yellow solid (2.58 g, 48%): R_f 0.26 (PE/EtOAc 60:40); m.p. 102–104 °C (from PE/EtOAc) [lit.[15] 112–114 °C]; $\tilde{\upsilon}_{max}$ (neat)/cm^{-1} 3233 (N-H, w), 3053 (C-H, w), 2977 (C-H, w), 1703 (C=O, s), 1555 (N-C=O, s), 1270 (C-O, s), 1166 (s), 1050 (C-S, s), 936 (C-H, s), 722 (C-H, s); ^{1}H NMR (400 MHz; $CDCl_3$) δ_H 8.70 (1H, s, H-2), 7.41 (2H, d, *J* 8.8, H-2'), 7.35 (2H, d, *J* 8.8, H-3'), 4.92 (1H, br s, *N*H-6'), 4.36 (2H, d, *J* 6.0, H-5'), 2.53 (3H, s, H-1"), 1.47 (9H, s, H-10'); LRMS *m/z* (ESI$^+$) 305 ([M+H]$^+$, 100%). The spectroscopic data are in good agreement with the literature values.[14]

(2*S*,4*R*)-*Tert*-butyl 2-((4-(4-methylthiazol-5-yl)benzyl)carbamoyl)-4-((4-nitrobenzyl)oxy)pyrrolidine-1-carboxylate (37)

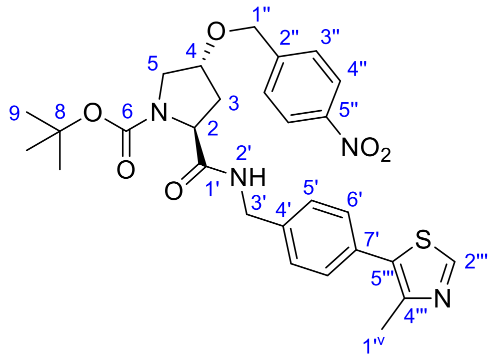

Step 1:

Compound **36** (1.77 g, 5.80 mmol, 1.0 eq.) was dissolved in CH_2Cl_2 (15 mL) and HCl (4 M in dioxane, 8.70 mL, 34.80 mmol, 6.0 eq.) was added dropwise. The reaction was stirred for 2 h at rt. After this time, the volatile components were evaporated *in vacuo* to afford the crude material as a yellow solid, which was used in the next step without further purification.

Step 2:

Compound **33** (183 mg, 0.50 mmol, 1 eq.) was solubilized in dry THF (2 mL) under argon, and dry Et_3N (0.21 mL, 1.50 mmol, 3 eq.) and PyBOP (416 mg, 0.80 mmol, 1.6 eq.) were added. After 10 min of stirring at rt, the intermediate (184 mg, 0.90 mmol, 1.8 eq.) obtained in Step 1 was added. The reaction mixture was stirred at rt for 22 h, after which water (10 mL) was added and extracted with EtOAc (3 × 10 mL). The combined organic layers were washed with saturated aqueous K_2CO_3 solution, dried over sodium sulfate, filtered, and concentrated *in vacuo*. The crude material was purified using silica gel column chromatography, eluting with toluene:acetone (gradient 15 to 30% acetone) yielded the title compound as a pale yellow solid (140 mg, 50%): *Rf* 0.09 (15% acetone in toluene); m.p. 78–80 °C (from CH_2Cl_2); $[a]_D^{25} = -16.7$ (*c* 1.0, $CHCl_3$); $\tilde{\upsilon}_{max}$ (thin film)/cm^{-1} 3305 (N-H, w), 2979 (C-H, w), 1694 (C=O, s), 1665 (C=O, s), 1543 (N-H, w), 1520 (N-O, s), 1404 (C-H, w), 1346 (N-O, s); ^{1}H NMR (500 MHz; CD_3OD) δ_H 8.88 (1H, s, H-2'''), 8.20 (2H, d, *J* 8.6, H-4''), 7.57 (2H, d, *J* 8.6, H-3''), 7.45–7.41 (4H, m, H-5', H-6'), 4.72–4.62 (2H, m, H-1''a, H-1''b), 4.53–4.24 (4H, m, H-2, H-4, H-3'a, H-3'b), 3.74 (1H, d, *J* 11.9, H-5a), 3.58 (1H, dd, *Js* 11.9 4.0, H-5b), 2.53–2.43 (1H, m, H-3a), 2.47 (3H, s, H-1^{iv}), 2.08 (1H, ddd, *Js* 13.4, 8.5, 4.7, H-3b), 1.33 (9H, s, H-9); ^{13}C NMR (126 MHz, CD_3OD) δc 175.3 (C-1'), 156.2 (C-6), 152.9 (C-2'''), 149.1 (C-4'''), 148.8 (C-7'), 147.5 (C-2''), 140.3 (C-3'''), 133.2 (C-5'''), 131.9 (C-4'), 130.5 (C-6'), 129.7 (C-5'), 129.0 (C-3''), 124.5 (C-4''), 81.8 (C-8), 78.4 (C-4), 70.6 (C-1''), 60.9 (C-2), 53.1 (C-5), 43.8 (C-3'), 38.3 (C-3), 28.5 (C-9), 15.8 (C-

1'v); HRMS *m/z* (ESI+) [Found: 553.2115, $C_{28}H_{33}N_4O_6S$ requires $[M+H]^+$ 553.2155]; LRMS *m/z* (ESI−) 551 $(M-H]^-$, 100%). HPLC Retention Time 9.4 min, 95.7%.

(2*S*,4*R*)-1-((*S*)-2-Acetamido-3,3-dimethylbutanoyl)-*N*-(4-(4-methylthiazol-5-yl)benzyl)-4-((4-nitrobenzyl)oxy)pyrrolidine-2-carboxamide (1)

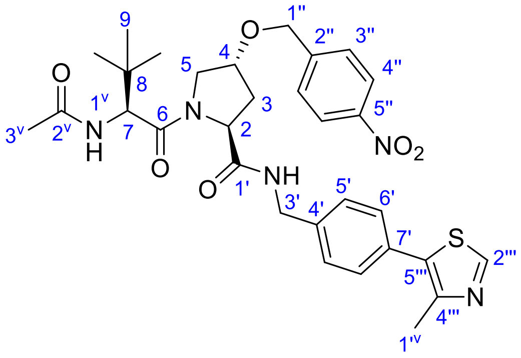

Step 1:

Compound **37** (65 mg, 0.12 mmol, 1 eq.) was dissolved in CH_2Cl_2 (0.4 mL) and HCl (4 M in dioxane, 0.44 mL, 1.76 mmol, 15 eq.) was added dropwise. The reaction was stirred for 1 h at rt. After this time, the reaction was not completed, as judged by TLC analysis, and further and HCl (4 M in dioxane, 0.22 mL, 0.88 mmol, 7.5 eq) was added. After 1 h at rt, TLC indicated full consumption of the starting material. The volatile components were evaporated *in vacuo* to afford the crude material as a yellow solid, which was used in the next step without further purification.

Step 2:

(2*S*)-2-Acetamido-3,3-dimethylbutanoic acid (20 mg, 0.12 mmol, 1 eq.) was solubilized in dry DMF (1 mL) under argon, and DIPEA (41 μL, 0.23 mmol, 2 eq.) and HATU (49 mg, 0.13 mmol, 1.1 eq.) were added. After 10 min of stirring at rt, the intermediate (57.5 mg, 0.117 mmol, 1 eq.) obtained in Step 1 was added. The reaction mixture was stirred at rt for 16 h, after which water (10 mL) was added and extracted with EtOAc (5 × 10 mL). The combined organic layers were washed with 0.5 M aqueous LiCl solution, dried over sodium sulfate, filtered, and concentrated *in vacuo*. The crude material was purified using silica gel column chromatography, eluting with toluene:acetone (gradient 30 to 50% acetone) yielded the title compound as a colourless solid (25 mg, 35%): *Rf* 0.24 (50% acetone in toluene); m.p. 78–80 °C (from CH_2Cl_2); $[\alpha]_D^{25} = -29.6$ (*c* 1.0, $CHCl_3$); $\tilde{\upsilon}_{max}$ (neat)/cm^{-1} 3306 (N-H, w), 2961 (C-H, w), 1645 (C=O, s), 1542 (N-H, w), 1520 (N-O, w), 1430 (C-H, w), 1419 (C-H, w), 1345 (N-O, s); ^1H NMR (500 MHz; $CDCl_3$) δ_H 8.67 (1H, s, H-2'''), 8.21 (2H, d, *J* 8.7, H-4''), 7.47 (2H, d, *J*

8.7, H-3''), 7.42–7.40 (1H, m, *N*H-2'), 7.38 (2H, d, *J* 8.2, H-6'), 7.29 (2H, d, *J* 8.2, H-5'), 5.97 (1H, d, *J* 7.4, *N*H-1ᵛ), 4.77 (1H, dd, J_s 8.7, 3.7, H-2), 4.67 (1H, d, *J* 12.9, H-1''a), 4.60 (1H, d, *J* 12.9, H-1''b), 4.59 (1H, dd, J_s 15.3, 7.0, H-3'a), 4.37 (1H, dddd, J_s 7.0, 6.3, 5.7, 5.1, H-4), 4.34 (1H, d, *J* 7.4, H-7), 4.29 (1H, dd, J_s 15.3, 5.1, H-3'b), 4.19 (1H, dd, J_s 10.6, 6.3, H-5a), 3.74 (1H, dd, J_s 10.6, 5.1, H-5b), 2.61 (1H, ddd, J_s 13.0, 5.7, 3.7, H-3a), 2.52 (3H, s, H-1ⁱᵛ), 2.23 (1H, ddd, J_s 13.0, 8.7, 7.0, H-3b), 1.76 (3H, s, H-3ᵛ), 1.06 (9H, s, H-9); ^{13}C NMR (126 MHz, $CDCl_3$) δc 171.5 (C-2ᵛ), 171.3 (C-6), 170.7 (C-1'), 150.4 (C-2'''), 148.6 (C-4'''), 147.7 (C-5''), 145.1 (C-2''), 138.5 (C-7'), 131.8 (C-5'''), 130.9 (C-4'), 129.5 (C-6'), 127.8 (C-5'), 127.7 (C-3''), 123.9 (C-4''), 77.4 (C-4), 70.5 (C-1''), 59.3 (C-7), 58.7 (C-2), 52.9 (C-5), 43.0 (C-3'), 34.5 (C-3), 34.2 (C-8), 26.7 (C-9), 22.9 (C-3ᵛ), 16.2 (C-1ⁱᵛ); HRMS *m/z* (ESI$^+$) [Found: 608.2535, $C_{31}H_{38}N_5O_6S$ requires [M+H]$^+$ 608.2537]; LRMS *m/z* (ESI$^+$) 608 (M+H]$^+$, 100%). HPLC Retention Time 7.2 min, 96.0%.

(2*S*,4*R*)-1-((*S*)-2-((*tert*-Butoxycarbonyl)amino)-3,3-dimethylbutanoyl)-4-hydroxypyrrolidine-2-carboxylic acid (40)

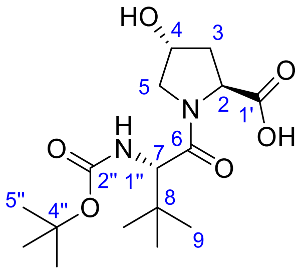

Step 1:

L-4-Hydroxyproline methyl ester hydrochloride (3.45 g, 19.02 mmol, 1.1 eq.) was added to a solution of Boc-L-*tert*-leucine (4.00 g, 17.29 mmol, 1.0 eq.), HATU (7.89 g, 20.75 mmol, 1.2 eq.) and DIPEA (18 mL, 103.74 mmol, 6.0 eq.) in dry DMF (80 mL). The reaction was stirred at rt for 16 h, after which it was diluted with water (250 mL) and extracted with EtOAc (3 × 200 mL). The combined organic phases were washed with saturated aqueous $NaHCO_3$ solution (2 × 400 mL), 1 M aqueous citric acid solution (3 × 400 mL), then dried over sodium sulfate, filtered, and concentrated *in vacuo* to afford (2*S*,4*R*)-methyl 1-((*S*)-2-((*tert*-butoxycarbonyl)amino)-3,3-dimethylbutanoyl)-4-hydroxypyrrolidine-2-carboxylate (5.85 g, 86%) which was used in the next step without further purification.

Step 2:

The obtained intermediate (5.69 g, 15.87 mmol, 1.0 eq.) was dissolved in THF (80 mL), and a solution of LiOH (3.81 g, 158.75 mmol, 10.0 eq.) in H_2O (70 mL) was added slowly. The

mixture was stirred at rt for 16 h, then THF was removed *in vacuo* and ice (50 g) was added. The pH was adjusted to 2-3 with 6 M aqueous HCl solution (10 mL) resulting in formation of a colourless precipitate. This solid was collected using *vacuum* filtration, washed with H_2O (1 × 20 mL) and dried *in vacuo* to yield a colourless solid (5.46 g, 100%): R_f 0.15 (EtOAc/CH_3OH 70:30); m.p. 84–86 °C (from water); $[\alpha]_D^{25} = -34.2$ (*c* 1.0, CH_3OH); $\tilde{\upsilon}_{max}$ (neat)/cm^{-1} 3398 (O-H, s), 2976 (C-H, w), 1723 (C=O, s), 1689 (C=O, s), 1615 (C=O, s), 1254 (C-O, s), 1171 (s), 1058 (C-O, s), 690 (s); ^{1}H NMR (400 MHz; D$_6$-DMSO) δ_H 6.46 (1H, d, *J* 9.4, N*H*-1''), 5.19 (1H, br s, O*H*), 4.34–4.30 (1H, m, H-4), 4.26 (1H, dd, J_s 8.3, 8.3, H-2), 4.15 (1H, d, *J* 9.4, H-7), 3.64 (1H, dd, *J* 10.6, 4.6, H-5^a), 3.57 (1H, dd, *J* 10.6, 4.6, H-5^b), 2.09 (1H, ddd, *J* 13.2, 8.3, 4.6, H-3^a), 1.88 (1H, ddd, *J* 13.2, 8.3, 4.6, H-3^b), 1.38 (9H, s, H-5''), 0.93 (9H, s, H-9); ^{13}C NMR (101 MHz, D$_6$-DMSO) δ 173.5 (C-1'), 170.0 (C-6), 155.4 (C-2''), 78.3 (C-4''), 68.8 (C-4), 58.3 (C-7), 57.9 (C-2), 56.1 (C-5), 37.4 (C-8), 35.5 (C-3), 28.3 (C-5''), 26.3 (C-9); HRMS *m/z* (ESI$^+$) [Found: 367.1838, $C_{16}H_{28}N_2O_6Na$ requires [M+Na]$^+$ 367.1840]; LRMS *m/z* (ESI$^-$) 343 ([M−H]$^-$, 100%). The spectroscopic data are in good agreement with the literature values.[3]

tert-Butyl ((*S*)-1-((2*S*,4*R*)-4-hydroxy-2-((4-(4-methylthiazol-5-yl)benzyl)carbamoyl)pyrrolidin-1-yl)-3,3-dimethyl-1-oxobutan-2-yl)carbamate (41)

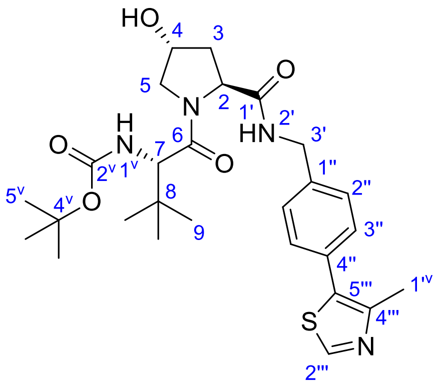

Step 1:

Compound **36** (1.77 g, 5.80 mmol, 1.0 eq.) was dissolved in CH_2Cl_2 (15 mL) and HCl (4 M in dioxane, 8.70 mL, 34.80 mmol, 6.0 eq.) was added dropwise. The reaction was stirred for 2 h at rt. After this time, the volatile components were evaporated *in vacuo* to afford the crude material as a yellow solid, which was used in the next step without further purification.

Step 2:

Compound **40** (2.00 g, 5.80 mmol, 1.0 eq.) was dissolved in dry DMF (20 mL) and DIPEA (6.06 mL, 34.80 mmol, 6.0 eq.) and HATU (2.65 g, 6.96 mmol, 1.2 eq.) were added under nitrogen. After 10 min under stirring at rt, the intermediate (5.80 mmol, 1.0 eq.) obtained in

Step 1 was added. The reaction was stirred at rt overnight, after which time water (50 mL) was added, and the mixture was extracted with EtOAc (4 × 30 mL). The combined organic phases were washed with saturated aqueous $NaHCO_3$ solution (5 × 100 mL), 5% aqueous citric acid solution (1 × 100 mL), and 0.5 M aqueous LiCl solution (2 × 100 mL), then dried over sodium sulfate, filtered, and concentrated *in vacuo*. The crude material was purified using silica gel column chromatography, eluting with PE:EtOAc (gradient 20 to 100% EtOAc) yielded the title compound as a colourless solid (1.43 g, 46%): R_f 0.17 (EtOAc); m.p. 68–70 °C (from EtOAc/CH_3OH) [lit.[15] 82–83 °C]; $[\alpha]_D^{25}$ = −29.5 (*c* 1.0, CH_3OH); $\tilde{\nu}_{max}$ (neat)/cm^{-1} 3435 (O-H, s), 3313 (N-H, w), 2964 (C-H, w), 2937 (C-H, w), 1686 (C=O, s), 1633 (C=O, s), 1525 (N-C=O, s), 1435 (C-H, w), 1238 (C-O, s), 1167 (C-S, s), 804 (C-H, s); ^1H NMR (400 MHz; $CDCl_3$) δ_H 8.67 (1H, s, H-2'''), 7.51 (1H, br t, *J* 5.3, N*H*-2'), 7.34–7.30 (4H, m, H-2'', H-3''), 5.24 (1H, d, *J* 9.2, N*H*-1^v), 4.71 (1H, dd, *J* 7.9, 8.0, H-2), 4.56–4.48 (2H, m, H-4, H-3'^{a}), 4.30 (1H, dd, *J* 9.8, 5.3, H-3'^{b}), 4.17 (1H, d, *J* 9.2, H-7), 3.99 (1H, dd, *J* 11.3, 3.7, H-5^a), 3.60 (1H, dd, *J* 11.3, 3.7, H-5^b), 2.49–2.42 (4H, m, H-3^a, H-1'^{v}), 2.10 (1H, ddd, *J* 11.8, 7.9, 5.5, H-3^b), 1.39 (9H, s, H-5^v), 0.90 (9H, s, H-9); ^{13}C NMR (101 MHz; $CDCl_3$) δ_C 172.5 (C-6), 171.0 (C-1'), 156.4 (C-2^v), 150.5 (C-2'''), 148.5 (C-4'''), 138.2 (C-4''), 131.8 (C-1''), 130.9 (C-5'''), 129.6 (C-3''), 128.1 (C-2''), 80.5 (C-4^v), 70.2 (C-4), 59.0 (C-7), 58.6 (C-2), 56.7 (C-5), 43.3 (C-3'), 36.1 (C-3), 35.2 (C-8), 28.4 (C-5^v), 26.4 (C-9), 16.1 (C-1'^{v}); HRMS *m/z* (ESI^+) [Found: 531.2627, $C_{27}H_{39}N_4O_5S$ requires $[M+H]^+$ 531.2636]; LRMS *m/z* (ESI^+) 531 ($[M+H]^+$, 100%); HPLC Retention time 8.4 min, 98.9%. The spectroscopic data are in good agreement with the literature values.[15]

tert-Butyl ((_S_)-3,3-dimethyl-1-((2_S_,4_R_)-4-((((1-methyl-2-nitro-1_H_-imidazol-5-yl)methoxy)carbonyl)oxy)-2-((4-(4-methylthiazol-5-yl)benzyl)carbamoyl)pyrrolidin-1-yl)-1-oxobutan-2-yl)carbamate (4)

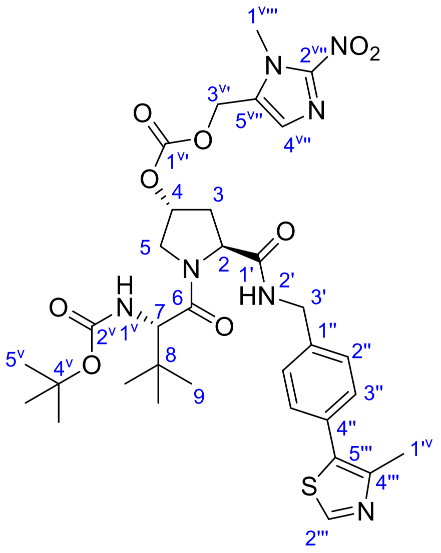

Step 1:

To a solution of **41** (200 mg, 0.38 mmol, 1.0 eq.) in dry CH_2Cl_2 (10 mL), a solution of 4-nitrophenyl chloroformate (760 mg, 3.77 mmol, 10.0 eq.) and triethylamine (670 µL, 3.77 mmol, 10.0 eq.) in dry CH_2Cl_2 (20 mL) was added dropwise at 0 °C. The resulting solution was warmed to rt and stirred for 1 h, then diluted with water (20 mL) before extracting with EtOAc (3 × 40 mL). The combined organic layers were washed with brine (3 × 20 mL), dried over sodium sulfate, filtered, and concentrated *in vacuo.* The crude material was purified using silica gel column chromatography, eluting with acetone in toluene (gradient 10 to 15% acetone) yielded the title compound as a yellow solid (65 mg, 25%).

Step 2:

The intermediate obtained in Step 1 (60 mg, 86.2 µmol, 1.3 eq.) was solubilized in dry DMF (0.2 mL) and DMAP (12 mg, 99.6 µmol, 1.5 eq.) was added. A solution of **22** (10.5 mg, 66.4 µmol, 1.0 eq.) in dry DMF (0.2 mL) was then added. The reaction was stirred at rt for 18 h, after which the solution was diluted with water (10 mL) and extracted with CH_2Cl_2 (3 × 10 mL). The combined organic layers were dried over sodium sulfate, filtered, and concentrated *in vacuo.* The crude material was purified using silica gel column chromatography, eluting with acetone in toluene (gradient 10 to 25% acetone) yielded the title compound as a colourless solid (34 mg, 55%): R_f 0.48 (CH_2Cl_2/CH_3OH 97:3); m.p. 70–72 °C (from toluene); $[\alpha]_D^{25}$ = −6.3 (*c* 1.0, $CHCl_3$); $\tilde{\upsilon}_{max}$ (neat)/cm^{-1} 2959 (C-H, w), 1749 (C=O, s), 1683 (C=O, s), 1640 (C=O, s), 1539 (N-O, s), 1366 (N-O, s) 1255 (C-O, w); ^{1}H NMR (400 MHz, $CDCl_3$) δ_H 8.86 (1H, s, H-

2'''), 7.36–7.32 (4H, m, H-2'', H-3''), 7.24 (1H, s, H-4^{v}''), 5.31–5.29 (1H, m, H-4), 5.27 (1H, d, *J* 13.8 Hz, H-3^{v}'a), 5.16 (1H, d, *J* 9.1 Hz, *N*H-1^{v}), 5.13 (1H, d, *J* 13.8 Hz, H-3^{v}'b), 4.75 (1H, t, *J* 7.8 Hz, H-2), 4.59 (1H, dd, J_s 14.9, 6.7 Hz, H-3'a), 4.32 (1H, dd, J_s 14.9, 5.1 Hz, H-3'b), 4.18 (1H, d, *J* 11.5 Hz, H-5a), 4.16 (1H, d, *J* 9.1 Hz, H-7), 4.04 (3H, s, H-1^{v}'''), 3.78 (1H, dd, J_s 11.5, 3.5 Hz, H-5b), 2.82 (1H, ddd, J_s 13.5, 7.8, 4.5 Hz, H-3a), 2.55 (3H, s, H-1'^{v}), 2.27–2.25 (1H, m, H-3b), 1.38 (9H, s, H-5^{v}), 0.89 (9H, s, H-9); ^{13}C NMR (101 MHz; $CDCl_3$) δ_C 172.5 (C-1'), 169.8 (C-6), 155.8 (C-2^{v}), 153.8 (C-2'''), 153.7 (C-1^{v}'), 147.8 (C-4'''), 147.1 (C-2^{v}''), 138.4 (C-1''), 132.8 (C-5'''), 132.0 (C-5^{v}'') 130.1 (C-4^{v}''), 129.6 (C-4''), 128.4 (2C, C-2'', C-3''), 79.8 (C-4^{v}), 77.8 (C-4), 58.7 (C-7), 58.4 (C-3^{v}'), 58.1 (C-2), 53.4 (C-5), 43.4 (C-3'), 35.3 (C-8), 32.4 (C-3), 28.3 (2C, C-5^{v}, C-1^{v}'''), 26.3 (C-9), 15.5 (C-1'^{v}); HRMS *m/z* (ESI^{+}) [Found: 714.2910, $C_{33}H_{44}N_7O_9S$ requires $[M+H]^+$ 714.2916]; LRMS *m/z* (ESI^{+}) 715 ($[M+H]^+$, 100%); HPLC Retention time 9.5 min, 96.0%.

(2*S*,4*R*)-1-((*S*)-14-Azido-2-(*tert*-butyl)-4-oxo-6,9,12-trioxa-3-azatetradecan-1-oyl)-4-hydroxy-*N*-(4-(4-methylthiazol-5-yl)benzyl)pyrrolidine-2-carboxamide (42)

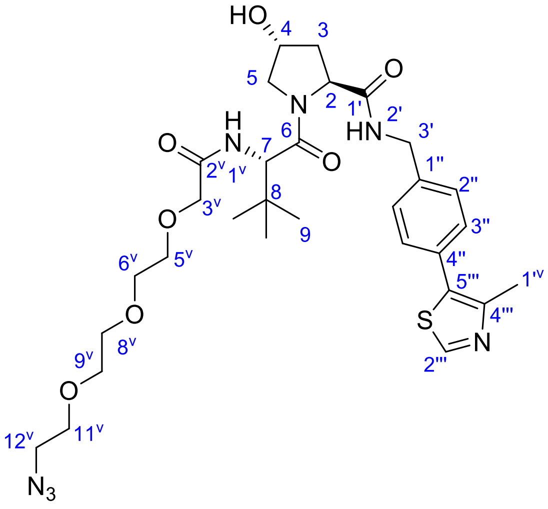

Step 1:

Compound **41** (570 mg, 1.07 mmol, 1.0 eq.) was dissolved in CH_2Cl_2 (5.6 mL) and HCl (4 M in dioxane, 3.21 mL, 12.84 mmol, 12.0 eq.) was added. The mixture was stirred at rt for 2 h, then the volatile components were evaporated *in vacuo* to afford the crude material as a yellow solid, which was used in the next step without further purification.

Step 2:

The intermediate obtained in Step 1 (1.07 mmol, 1.0 eq.) was added to a stirring solution of 2-(2-(2-(2-azidoethoxy)ethoxy)ethoxy)acetic acid (249 mg, 1.07 mmol, 1.0 eq.), HATU (813 mg, 2.14 mmol, 2.0 eq.) and DIPEA (1.12 mL, 6.42 mmol, 6.0 eq.) in dry DMF (7.8 mL).

The reaction was stirred at rt for 16 h, after which it was diluted with water (40 mL) and extracted with EtOAc (3 × 40 mL). The organic layers were combined, washed with 0.5 M aqueous LiCl solution (1 × 100 mL), dried over sodium sulfate, filtered, and concentrated *in vacuo*. The crude product was purified using silica gel column chromatography, eluting with EtOAc:CH_3OH (gradient 0 to 10% CH_3OH) to isolate the title compound as an amorphous yellow solid (433 mg, 63%): R_f 0.27 (EtOAc/CH_3OH 90:10); $[\alpha]_D^{25}$ = −15.8 (*c* 1.0, CH_3OH); $\tilde{\upsilon}_{max}$ (neat)/cm^{-1} 3306 (O-H, s), 2918 (C-H, w), 2107 (N=N=N, s), 1665 (C=O, s), 1636 (C=O, s), 1525 (N-H, s), 1436 (N-H, s), 1112 (C-O, s) 850 (C-H, w); ^{1}H NMR (400 MHz, $CDCl_3$) δ_H 8.67 (1H, s, H-2'''), 7.39–7.31 (4H, m, H-2'', H-3''), 7.28 (1H, br s, N*H*-2'), 4.73 (1H, dd, J_s 7.8, H-2), 4.57–4.47 (3H, m, H-4, H-7, H-3'^{a}), 4.33 (1H, ddd, *J* 15.4, 7.8, 5.3, H-3'^{b}), 4.08 (1H, dd, *J* 11.5, 2.4, H-5^a), 4.02 (1H, d, *J* 15.8, H-3va), 3.96 (1H, d, *J* 15.8, H-3vb), 3.68–3.59 (11H, m, H-5^a, H-5^v, H-6^v, H-8^v, H-9^v, H-11^v), 3.36 (2H, t, *J* 5.1, H-12^v), 3.26 (1H, br s, O*H*), 2.58–2.51 (4H, m, H-3^a, H-1'^{v}), 2.10 (1H, ddd, *J* 7.7, 5.4, 1.7, H-3^b), 0.95 (9H, s, H-9); ^{13}C NMR (101 MHz; $CDCl_3$) δ_C 171.6 (C-6), 170.8 (C-1'), 170.7 (C-2^v), 150.4 (C-2'''), 148.6 (C-4'''), 138.2 (C-4''), 131.7 (C-1''), 131.1 (C-5'''), 129.7 (C-3''), 128.3 (C-2''), 71.3 (C-5^v), 70.8 (2C, C-8^v, C-9^v), 70.7 (C-6^v), 70.5 (C-11^v), 70.3 (C-3^v), 70.2 (C-4), 58.5 (C-2), 57.3 (C-7), 56.7 (C-5), 50.8 (C-12^v), 43.4 (C-3'), 35.9 (C-8), 35.0 (C-3), 26.5 (C-9), 16.2 (C-1'^{v}); HRMS *m/z* (ESI$^+$) [Found: 646.3014, $C_{30}H_{44}N_7O_7S$ requires $[M+H]^+$ 646.3017]; LRMS *m/z* (ESI$^+$) 646 ($[M+H]^+$, 100%), 668 ($[M+Na]^+$, 93%); HPLC Retention time 7.8 min, 93.4%.[16]

(2*S*,4*R*)-1-((*S*)-2-(*tert*-Butyl)-17-((*S*)-4-(4-chlorophenyl)-2,3,9-trimethyl-6*H*-thieno[3,2-*f*][1,2,4]triazolo[4,3-*a*][1,4]diazepin-6-yl)-4,16-dioxo-6,9,12-trioxa-3,15-diazaheptadecan-1-oyl)-4-hydroxy-*N*-(4-(4-methylthiazol-5-yl)benzyl)pyrrolidine-2-carboxamide (8, MZ1)

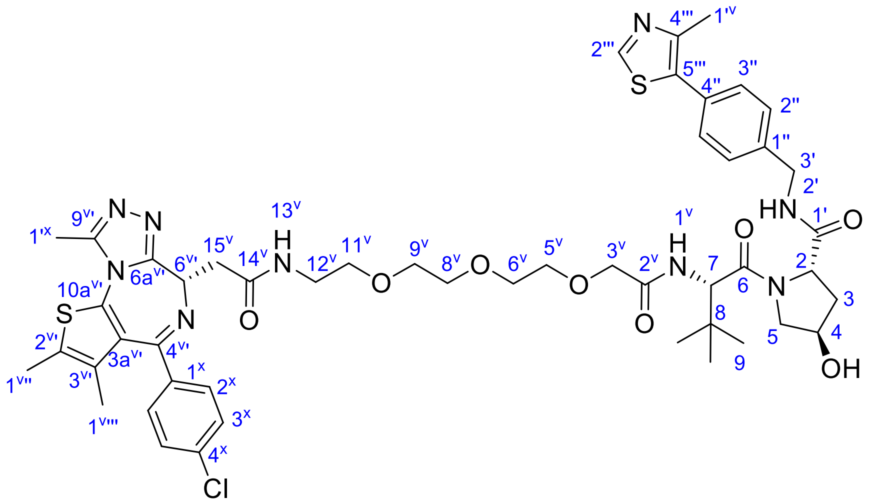

Step 1:

Compound **42** (100 mg, 0.15 mmol, 1.0 eq.) was added to a round-bottom flask containing Pd/C 10% (10 mg, 10% *w/w*) and CH_3OH (15 mL). The reaction vessel was flushed with hydrogen and the mixture was stirred at rt for 4 h. The mixture was then filtered over a pad of Celite® and washed with CH_3OH (2 × 20 mL). The volatile components were evaporated to afford the crude material, which was used in the next step without further purification.

Step 2:

The obtained intermediate was added to a stirring solution of DIPEA (104 µL, 0.60 mmol, 4.0 eq.), HATU (68 mg, 0.18 mmol, 1.2 eq.) and (+)-JQ1-COOH (54 mg, 0.14 mmol, 0.9 eq.) in dry DMF (2 mL) under argon. The reaction mixture was stirred at rt overnight. After this time, water (20 mL) was then added and the mixture was extracted with EtOAc (6 × 20 mL). The combined organic phases were dried over sodium sulfate, filtered, and evaporated *in vacuo*. The crude material was purified using silica gel column chromatography, eluting with EtOAc:CH_3OH (gradient 0 to 15% CH_3OH) yielding compound **8** as a colourless solid (99 mg, 66%): R_f 0.14 (EtOAc:CH_3OH 80:20); m.p. 100–102 °C (from EtOAc:CH_3OH); $[a]_D^{25}$ = +7.7 (*c* 1.0, CH_3OH); $\tilde{\upsilon}_{max}$ (neat)/cm^{-1} 3309 (O-H, s), 2923 (C-H, w), 1640 (C=O, s), 1532 (N-H, s), 1420 (N-H, s), 1370 (s), 1091 (C-O, s) 846 (C-H, w), 806 (C-H, w); ^{1}H NMR (400 MHz; $CDCl_3$) δ_H 8.78 (1H, s, H-2'''), 8.03 (1H, br s, N*H*-13^{v}), 7.45 (1H, br s, N*H*-1^{v}), 7.41 (2H, d, *J* 8.2, H-3^{x}), 7.35–7.31 (6H, m, H-2'', H-3'', H-2^{x}), 4.83 (1H, dd, J_s 7.9, H-2), 4.73–4.68 (2H, m, H-7, H-6^{vi}), 4.57–4.47 (2H, m, H-4, H-3'^{a}), 4.33–4.27 (2H, m, H-3va, H-3'^{b}), 4.15–4.11 (2H, m, H-3vb, H-5^{a}), 3.72–3.54 (13H, m, H-5^{b}, H-5^{v}, H-6^{v}, H-8^{v}, H-9^{v}, H-11^{v}, H-12va, H-15va), 3.38–

3.32 (2H, m, H-12[vb], H-15[vb]), 2.66 (3H, s, H-1'[x]), 2.54 (3H, s, H-1[iv]), 2.45–2.39 (4H, m, H-1[v''], H-3[a]), 2.19–2.14 (1H, m, H-3[b]), 1.66 (3H, s, H-1[v''']), 0.99 (9H, s, H-9); ^{13}C NMR (101 MHz; $CDCl_3$) δ_C 171.5 (C-1'), 171.1 (C-14[v]), 170.9 (C-2[v]), 170.7 (C-6), 163.9 (C-4[v']), 155.9 (C-6a[v']), 150.3 (C-9[v']), 149.9 (C-2'''), 148.4 (C-4'''), 138.4 (C-1''), 136.8 (C-1[x]), 136.6 (C-4[x]), 131.9 (C-10a[v']), 131.8 (C-3a[v']), 131.1 (C-5'''), 131.0 (C-4''), 130.8 (C-2[v']), 130.7 (C-3[v']), 130.0 (C-3[x]), 129.5 (C-2[x]), 128.8 (C-3''), 128.0 (C-2''), 71.4 (C-5[v]), 70.8 (C-9[v]), 70.5 (C-6[v]), 70.4 (2C, C-3[v], C-8[v]), 70.3 (C-11[v]), 70.0 (C-4), 59.0 (C-2), 57.2 (C-5), 56.8 (C-7), 54.2 (C-6[v']), 43.1 (C-3'), 39.8 (C-12[v]), 38.2 (C-15[v]), 36.5 (C-3), 35.6 (C-8), 26.5 (C-9), 16.1 (C-1[iv]), 14.5 (C-1[v''']), 13.2 (C-1[v'']), 11.8 (C-1'[x]); HRMS *m/z* (ESI$^+$) [Found: 1002.3735, $C_{49}H_{61}ClN_9O_8S_2$ requires $[M+H]^+$ 1002.3768]; LRMS mass not detected; HPLC Retention time 8.8 min, 94.6%; The spectroscopic data are in good agreement with the literature values.[16]

(3*R*,5*S*)-1-((*S*)-2-(*tert*-Butyl)-17-((*S*)-4-(4-chlorophenyl)-2,3,9-trimethyl-6*H*-thieno[3,2-*f*][1,2,4]triazolo[4,3-*a*][1,4]diazepin-6-yl)-4,16-dioxo-6,9,12-trioxa-3,15-diazaheptadecan-1-oyl)-5-((4-(4-methylthiazol-5-yl)benzyl)carbamoyl)pyrrolidin-3-yl ((1-methyl-2-nitro-1*H*-imidazol-5-yl)methyl) carbonate (6, NI-VHL)

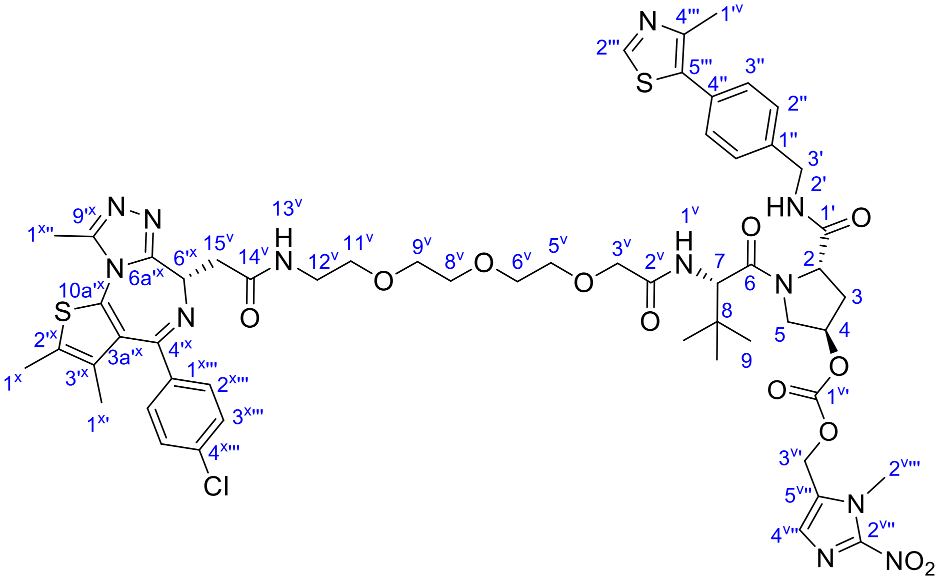

Compound **8** (90 mg, 0.09 mmol, 1.0 eq.) was dissolved in dry CH_2Cl_2 (5 mL) and dry Et_3N (38 µL, 0.27 mmol, 3.0 eq.) was added, followed by intermediate **24** (40 mg, 0.13 mmol, 1.4 eq.). The resulting solution was stirred at 40 °C for 16 h. After this time, the reaction was not complete, as judged by TLC analysis, and so additional dry Et_3N (38 µL, 0.27 mmol, 3.0 eq.) and intermediate **24** (40 mg, 0.13 mmol, 1.4 eq.) were added and the reaction mixture was stirred at 40 °C for a further 8 h. After this time, the reaction was not complete, as judged by

TLC analysis, and therefore further intermediate **24** (14 mg, 0.045 mmol, 0.5 eq.) was added. The reaction mixture was stirred at 40 °C for another 16 h. After this time, the reaction mixture was cooled to rt and water (5 mL) was added. The product was extracted with CH_2Cl_2 (3 × 5 mL). The combined organic layers were dried over sodium sulfate, filtered, and dried *in vacuo*. The crude material was purified using silica gel column chromatography, eluting with EtOAc:CH_3OH (gradient 0 to 15% CH_3OH) yielding compound **7** as a colourless solid (48 mg, 45%). The compound was further purified using semi-preparative HPLC, method A: R_f 0.26 (EtOAc:CH_3OH 80:20); m.p. 93–95 °C (from EtOAc:CH_3OH); $[\alpha]_D^{25}$ = +18.9 (*c* 1.0, CH_3OH); $\tilde{\upsilon}_{max}$ (neat)/cm^{-1} 3413 (N-H, w), 1750 (O-C(=O)-O, s), 1647 (C=O, s), 1538 (N-H, s), 1489 (N-H, s), 1433 (w), 1256 (C-O, s) 836 (C-H, w); ^{1}H NMR (600 MHz; CD_3OD) δ_H 8.86 (1H, s, H-2'''), 7.43–7.37 (8H, m, H-2'', H-3'', H-2$^{x'''}$, H-3$^{x'''}$), 7.23 (1H, s, H-4$^{v''}$), 5.33–5.26 (3H, m, H-4, H-3$^{v'}$), 4.61–4.51 (4H, m, H-2, H-7, H-3$^{'a}$, H-6$^{'x}$), 4.35–4.27 (2H, m, H-5^{a}, H-3$^{'b}$), 4.03 (1H, d, *J* 7.8, H-3va), 4.00 (1H, d, *J* 7.8, H-3vb), 3.98 (3H, s, H-2$^{v'''}$), 3.92 (1H, dd, *J* 12.2, 3.7, H-5^{b}), 3.71–3.65 (9H, m, H-5^{v}, H-6^{v}, H-8^{v}, H-9^{v}, H-11va), 3.59–3.57 (2H, m, H-11vb, H-12va), 3.45–3.41 (2H, m, H-12va, H-15vb), 3.30–3.28 (1H, m, H-15va), 2.67 (3H, s, H-1$^{x''}$), 2.47–2.45 (4H, m, H-3^{a}, H-1$^{'v}$), 2.44 (3H, s, H-1^{x}), 2.26 (1H, ddd, *J* 14.2, 9.5, 4.6, H-3^{b}), 1.69 (3H, s, H-1$^{x'}$), 1.04 (9H, s, H-9); ^{13}C NMR (151 MHz; CD_3OD) δ_C 173.6 (C-1'), 172.9 (C-14^{v}), 172.2 (C-2^{v}), 171.8 (C-6), 166.1 (C-4$^{'x}$), 157.0 (C-6a$^{'x}$), 155.1 (C-1$^{v'}$), 152.8 (C-2'''), 152.1 (C-9$^{'x}$), 149.1 (C-4'''), 147.6 (C-2$^{v''}$), 140.1 (C-1''), 138.1 (C-1$^{x'''}$), 137.9 (C-4$^{x'''}$), 133.6 (C-10a$^{'x}$), 133.5 (C-5$^{v''}$), 133.4 (C-5'''), 133.2 (C-4''), 132.0 (C-3$^{'x}$), 131.9 (C-2$^{'x}$), 131.6 (C-3a$^{'x}$), 131.4 (C-3$^{x'''}$), 130.4 (C-2$^{x'''}$), 130.1 (C-4$^{v''}$), 129.8 (C-3''), 129.0 (C-2''), 78.9 (C-4), 72.2 (C-5^{v}), 71.6 (C-9^{v}), 71.5 (C-6^{v}), 71.4 (C-8^{v}), 71.1 (C-3^{v}), 70.7 (C-11^{v}), 60.5 (C-2), 59.4 (C-3$^{v'}$), 58.4 (C-7), 55.3 (C-6$^{'x}$), 55.2 (C-5), 43.8 (C-3'), 40.6 (C-12^{v}), 38.7 (C-15^{v}), 36.5 (C-8), 36.0 (C-3), 35.0 (C-2$^{v'''}$), 26.9 (C-9), 15.8 (C-1$^{'v}$), 14.4 (C-1$^{x'}$), 12.9 (C-1^{x}), 11.6 (C-1$^{x''}$); HRMS *m/z* (ESI^+) [Found: 1185.4087, $C_{55}H_{66}ClN_{12}O_{12}S_2$ requires $[M+H]^+$ 1185.4048]; LRMS *m/z* (ESI^+) 1185 ($[^{35}M+H]^+$, 97%), 1187 ($[^{37}M+H]^+$, 83%), 1207 ($[^{35}M+Na]^+$, 100%), 1209 ($[^{37}M+Na]^+$, 90%); HPLC Retention time 9.4 min, 99.0%.

(2*S*,4*R*)-1-((*S*)-14-Azido-2-(*tert*-butyl)-4-oxo-6,9,12-trioxa-3-azatetradecan-1-oyl)-4-((5-methoxy-1,2-dimethyl-4,7-dioxo-4,7-dihydro-1*H*-indol-3-yl)methoxy)-*N*-(4-(4-methylthiazol-5-yl)benzyl)pyrrolidine-2-carboxamide (43)

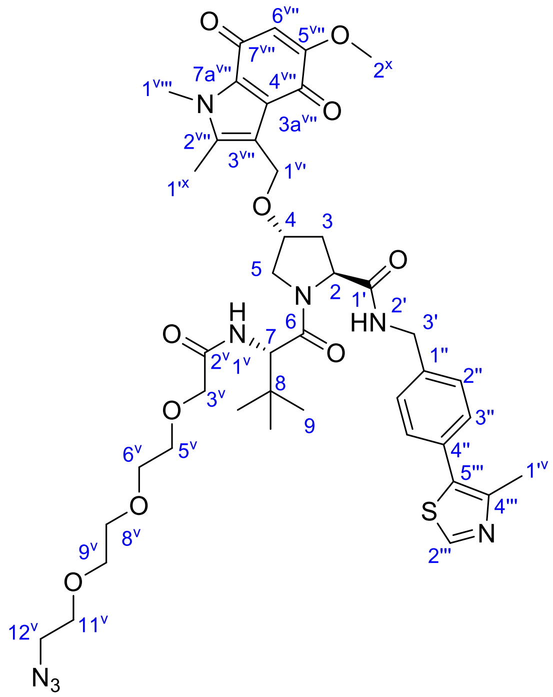

To a solution of compound **32** (100 mg, 0.15 mmol, 1.0 eq.) and TBAI (18 mg, 0.05 mmol, 0.3 eq.) in CH_2Cl_2 (5 mL), a 50% *v/wt* aqueous NaOH solution (1.25 mL) was added. After stirring this solution for 15 min at rt, compound **31** (51 mg, 0.17 mmol, 1.1 eq.) was added. The mixture was stirred for 1 h at rt while protected from light, then compound **31** (51 mg, 0.17 mmol, 1.1 eq.) was added and the reaction mixture was stirred for a further 1 h at rt. After this time, the reaction was not concluded, as judged by TLC analysis, and further compound **31** (18 mg, 0.06 mmol, 0.4 eq.) was added and the reaction mixture was stirred for a further 1 h at rt. Water (5 mL) was added and the mixture was extracted with CH_2Cl_2 (3 × 5 mL). The combined organic phases were dried over sodium sulfate, filtered, and concentrated *in vacuo*. Purification using silica gel column chromatography, eluting with EtOAc:CH_3OH (gradient 0 to 10% CH_3OH) yielded an orange solid, which was dissolved in EtOAc (15 mL) and the organic layer was washed with saturated aqueous NH_4Cl solution (4 × 15 mL). The organic layer was dried over sodium sulfate, filtered, and concentrated *in vacuo* to afford the title compound as an orange solid (47 mg, 36%): R_f 0.28 (EtOAc/CH_3OH 90:10); m.p. 42–44 °C (from EtOAc/CH_3OH); $[\alpha]_D^{25}$ = −2.5 (*c* 1.0, CH_3OH); $\tilde{\upsilon}_{max}$ (neat)/cm^{-1} 3404 (N-H, w), 2980 (C-H, s), 2888 (C-H, w), 2106 (N=N=N, w), 1671 (C=O, s), 1635 (C=O, s), 1600 (C=O, s), 1507 (N-H, s), 1461 (N-H, s), 1225 (s), 1151 (C-O, s), 1088 (C-O, w), 1018 (C-O, w), 850 (C-H, w); ^{1}H

NMR (600 MHz; $CDCl_3$) δ_H 8.67 (1H, s, H-2'''), 7.38–7.33 (4H, m, H-2'', H-3''), 7.23 (1H, br s, N*H*-1^{v}), 7.08 (1H, br t, *J* 6.0, N*H*-2'), 5.59 (1H, s, H-6$^{v''}$), 4.75 (1H, d, *J* 11.1, H-1$^{v'b}$), 4.70 (1H, d, *J* 9.5, H-7), 4.64–4.55 (3H, m, H-2, H-3$^{'a}$, H-1$^{v'a}$), 4.44–4.39 (2H, m, H-4, H-3$^{'b}$), 4.07 (1H, dd, *J* 10.7, 2.6, H-5^{a}), 4.01 (1H, d, *J* 15.5, H-3va), 3.95 (1H, d, *J* 15.5, H-3vb), 3.86 (3H, s, H-1$^{v'''}$), 3.78 (3H, s, H-2^{x}), 3.71–3.61 (11H, m, H-5^{a}, H-5^{v}, H-6^{v}, H-8^{v}, H-9^{v}, H-11^{v}), 3.34 (2H, t, *J* 5.2, H-12^{v}), 2.55–2.49 (4H, m, H-3^{a}, H-1$^{'v}$), 2.29–2.22 (4H, m, H-3^{b}, H-1$^{'x}$), 0.94 (9H, s, H-9); ^{13}C NMR (151 MHz; $CDCl_3$) δ_C 178.9 (C-4$^{v''}$), 178.2 (C-7$^{v''}$), 171.3 (C-6), 171.0 (C-1'), 169.4 (C-2^{v}), 159.8 (C-5$^{v''}$), 150.5 (C-2'''), 148.1 (C-4'''), 138.4 (C-1''), 138.0 (C-2$^{v''}$), 131.7 (C-5'''), 130.7 (C-4''), 129.7 (C-3''), 129.0 (C-7a$^{v''}$), 128.4 (C-2''), 121.8 (C-3a$^{v''}$), 117.9 (C-3$^{v''}$), 106.9 (C-6$^{v''}$), 76.9 (C-4), 71.3 (C-5^{v}), 70.9 (2C, C-8^{v}, C-9^{v}), 70.7 (2C, C-3^{v}, C-6^{v}), 70.2 (C-11^{v}), 60.7 (C-1$^{v'}$), 59.2 (C-2), 56.6 (C-2^{x}), 56.5 (C-7), 53.6 (C-5), 50.8 (C-12^{v}), 43.4 (C-3'), 36.0 (C-8), 33.6 (C-3), 32.5 (C-1$^{v'''}$), 26.5 (C-9), 16.1 (C-1$^{'v}$), 9.7 (C-1$^{'x}$); HRMS *m/z* (ESI^{+}) [Found: 863.3756, $C_{42}H_{55}N_8O_{10}S$ requires [M+H]$^{+}$ 863.3735]; LRMS mass not detected; HPLC Retention time 9.1 min, 96.4%.

(2*S*,4*R*)-1-((*S*)-14-Azido-2-(*tert*-butyl)-4-oxo-6,9,12-trioxa-3-azatetradecan-1-oyl)-4-(benzyloxy)-*N*-(4-(4-methylthiazol-5-yl)benzyl)pyrrolidine-2-carboxamide (44)

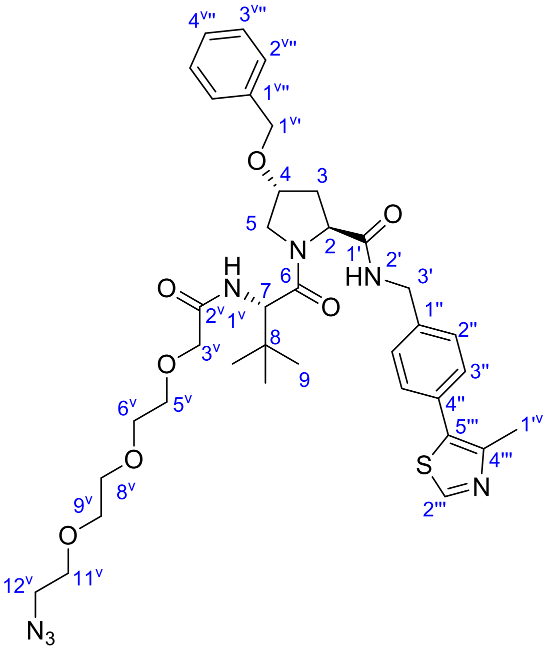

Compound **44** was synthesized as previously described for compound **43**, starting from compound **42** (100 mg, 0.15 mmol, 1.0 eq.) and benzyl bromide (20 µL, 0.17 mmol, 1.1 eq.). The mixture was stirred for 1h at rt while protected from light, then further benzyl bromide (20 µL, 0.17 mmol, 1.1 eq.) was added and the reaction mixture was stirred for a further 1 h at rt.

A second addition of benzyl bromide (7 μL, 0.06 mmol, 0.4 eq.) was performed and the reaction mixture was stirred for a further 1 h at rt. After this time, water (5 mL) was added and the mixture was extracted with CH_2Cl_2 (6 × 5 mL). The combined organic phases were dried over sodium sulfate, filtered, and concentrated *in vacuo*. Purification using silica gel column chromatography, eluting with PE:EtOAc (gradient 50 to 100% EtOAc) yielded the title compound as a yellow oil (90 mg, 81%): R_f 0.14 (EtOAc 100%); $[\alpha]_D^{25} = -2.4$ (*c* 1.0, CH_3OH); $\tilde{\upsilon}_{max}$ (neat)/cm^{-1} 2939 (C-H, s), 2869 (C-H, w), 2107 (N=N=N, s), 1678 (C=O, s), 1639 (C=O, s), 1520 (N-H, s), 1435 (N-H, s), 1112 (C-O, s), 1030 (C-O, w), 738 (C-H, w), 699 (C-H, w); ^{1}H NMR (400 MHz; $CDCl_3$) δ_H 8.67 (1H, s, H-2'''), 7.37–7.22 (11H, m, H-2'', H-3'', H-2$^{v''}$, H-3$^{v''}$, H-4$^{v''}$, N*H*-2', N*H*-1^v), 4.70–4.66 (2H, m, H-2, H-7), 4.57–4.45 (3H, m, H-3$^{'a}$, H-1$^{v'}$), 4.37–4.29 (2H, m, H-4, H-3$^{'b}$), 4.07 (1H, dd, *J* 10.8, 3.3, H-5^a), 4.02 (1H, d, *J* 15.5, H-3va), 3.92 (1H, d, *J* 15.5, H-3vb), 3.67–3.61 (11H, m, H-5^a, H-5^v, H-6^v, H-8^v, H-9^v, H-11^v), 3.34 (2H, t, *J* 5.2, H-12^v), 2.59 (1H, ddd, *J* 11.6, 5.8, 1.6, H-3^a), 2.51 (3H, s, H-1$^{'v}$), 2.13 (1H, ddd, *J* 11.6, 8.1, 4.0, H-3^b), 0.92 (9H, s, H-9); ^{13}C NMR (101 MHz; $CDCl_3$) δ_C 171.5 (C-6), 170.7 (C-1'), 169.6 (C-2^v), 150.4 (C-2'''), 148.5 (C-4'''), 138.1 (C-1''), 137.8 (C-1$^{v''}$), 131.7 (C-5'''), 131.1 (C-4''), 129.6 (C-3''), 128.5 (C-2''), 128.3 (C-2$^{v''}$), 127.9 (C-4$^{v''}$), 127.8 (C-3$^{v''}$), 77.0 (C-4), 71.2 (2C, C-5^v, C-1$^{v'}$), 70.8 (2C, C-8^v, C-9^v), 70.6 (C-3^v), 70.5 (C-6^v), 70.1 (C-11^v), 58.7 (C-2), 56.3 (C-7), 53.0 (C-5), 50.7 (C-12^v), 43.3 (C-3'), 35.7 (C-8), 33.5 (C-3), 26.4 (C-9), 16.1 (C-1$^{'v}$); HRMS *m/z* (ESI$^+$) [Found: 736.3466, $C_{37}H_{50}N_7O_7S$ requires $[M+H]^+$ 736.3487]; LRMS mass not detected; HPLC Retention time 9.8 min, 94.2%.

(2*S*,4*R*)-1-((*S*)-2-(*tert*-Butyl)-17-((*S*)-4-(4-chlorophenyl)-2,3,9-trimethyl-6*H*-thieno[3,2-*f*][1,2,4]triazolo[4,3-*a*][1,4]diazepin-6-yl)-4,16-dioxo-6,9,12-trioxa-3,15-diazaheptadecan-1-oyl)-4-((5-methoxy-1,2-dimethyl-4,7-dioxo-4,7-dihydro-1*H*-indol-3-yl)methoxy)-*N*-(4-(4-methylthiazol-5-yl)benzyl)pyrrolidine-2-carboxamide (7, IQ-VHL)

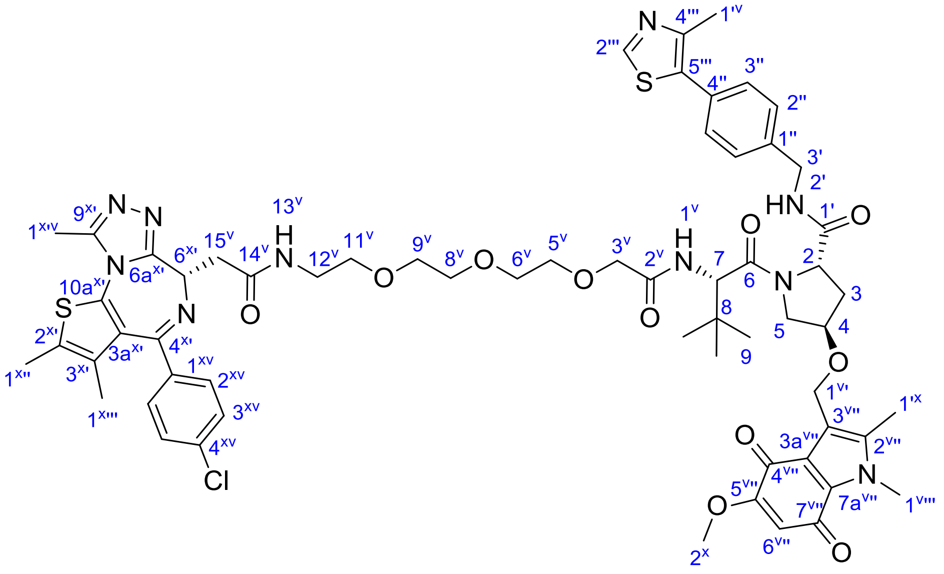

Step 1:

Compound **43** (42 mg, 0.05 mmol, 1.0 eq.) was dissolved in THF (1 mL) and PPh_3 (26 mg, 0.10 mmol, 2.0 eq.) was added. The reaction was stirred for 10 min at rt, then water (10 µL, 0.50 mmol, 10.0 eq.) was added. After 8 h at rt, 10 eq. of water were added, and the reaction mixture was stirred at rt overnight. After this time, the volatile components were evaporated *in vacuo* to afford the crude material as an orange solid, which was used in the next step without further purification.

Step 2:

(+)-JQ1-COOH (18 mg, 0.05 mmol, 0.9 eq.) was dissolved in dry DMF (0.5 mL) and DIPEA (35 µL, 0.20 mmol, 4.0 eq.) followed by HATU (23 mg, 0.06 mmol, 1.2 eq.) were added under argon. After 10 min, the intermediate (0.05 mmol, 1.0 eq.) obtained in Step 1 in dry DMF (0.5 mL) was added and the reaction mixture was stirred at rt overnight. Water (5 mL) was then added, and the mixture was extracted with EtOAc (10 × 5 mL). The combined organic phases were dried over sodium sulfate, filtered, and evaporated *in vacuo*. The crude material was purified using silica gel column chromatography, eluting with EtOAc:CH_3OH (gradient 0 to 15% CH_3OH) to yield the title compound as an orange solid (22 mg, 36%). The compound was further purified using semi-preparative HPLC, method B: R_f 0.15 (EtOAc:CH_3OH 80:20); m.p. 112–114 °C (from EtOAc:CH_3OH); $[\alpha]_D^{25} = -5.3$ (*c* 0.5, CH_3OH); $\tilde{\nu}_{max}$ (neat)/cm^{-1} 3390 (N-H,

w), 2924 (C-H, w), 2855 (C-H, w), 1669 (C=O, s), 1636 (C=O, s), 1599 (C=O, s), 1461 (N-H, s), 1437 (N-H, s), 1224 (s), 1090 (C-O, s), 1041 (C-O, w), 1015 (C-O, w), 842 (C-H, w), 804 (C-H, w); ^{1}H NMR (600 MHz; CD_3OD) δ_H 8.86 (1H, s, H-2'''), 7.45–7.35 (8H, m, H-2'', H-3'', H-2^{xv}, H-3^{xv}), 5.64 (1H, s, H-6$^{v''}$), 4.74 (1H, s, H-7), 4.66 (1H, d, *J* 11.2, H-1$^{v'a}$), 4.61–4.57 (2H, m, H-1$^{v'b}$,H-6$^{x'}$), 4.54–4.48 (2H, m, H-2,H-3$^{'a}$), 4.34 (1H, d, *J* 15.4, H-3$^{'b}$), 4.31–4.30 (1H, m, H-4), 4.14 (1H, dd, *J* 11.4, 4.4, H-5^{a}), 4.07 (1H, d, *J* 15.5, H-3va), 4.03 (1H, d, *J* 15.5, H-3vb), 3.83 (3H, s, H-1$^{v'''}$), 3.78–3.76 (4H, m, H-5^{b}, H-2^{x}), 3.71–3.62 (8H, m, H-5^{v}, H-6^{v}, H-8^{v}, H-9^{v}), 3.57–3.55 (2H, m, H-11va, H-11vb), 3.46–3.40 (3H, m, H-12va, H-12vb, H-15va), 3.28 (1H, dd, *J* 15.1, 5.1, H-15vb), 2.68 (3H, s, H-1$^{x'v}$), 2.50–2.46 (4H, m, H-3^{a}, H-1$^{'v}$), 2.43 (3H, s, H-1$^{x''}$), 2.22 (3H, s, H-1$^{'x}$), 2.06 (1H, ddd, *J* 13.6, 9.5, 4.4, H-3^{b}), 1.68 (3H, s, H-1$^{x'''}$), 1.04 (9H, s, H-9); ^{13}C NMR (151 MHz; CD_3OD) δ_C 180.1 (C-4$^{v''}$), 179.5 (C-7$^{v''}$), 174.3 (C-1'), 172.9 (C-14^{v}), 172.1 (C-2^{v}), 171.7 (C-6), 166.0 (C-4$^{x'}$), 161.1 (C-5$^{v''}$), 157.1 (C-6a$^{x'}$), 152.8 (C-2'''), 152.1 (C-9$^{x'}$), 149.1 (C-4'''), 140.2 (C-2$^{v''}$), 140.0 (C-1''), 138.1 (C-1^{xv}), 137.9 (C-4^{xv}), 133.5 (C-10a$^{x'}$), 133.4 (C-5'''), 133.2 (C-4''), 132.1 (C-3$^{x'}$), 132.0 (C-2$^{x'}$), 131.5 (C-3a$^{x'}$), 131.3 (C-3^{xv}), 130.4 (C-2^{xv}), 129.9 (C-7a$^{v''}$), 129.7 (C-3''), 129.0 (C-2''), 122.7 (C-3a$^{v''}$), 118.8 (C-3$^{v''}$), 107.6 (C-6$^{v''}$), 78.4 (C-4), 72.2 (C-5^{v}), 71.7 (C-9^{v}), 71.6 (C-6^{v}), 71.4 (C-8^{v}), 71.2 (C-3^{v}), 70.7 (C-11^{v}), 61.3 (C-1$^{v'}$), 60.9 (C-2), 58.1 (C-7), 57.1 (C-2^{x}), 55.2 (2C, C-5, C-6$^{x'}$), 43.7 (C-3'), 40.6 (C-12^{v}), 38.7 (C-15^{v}), 37.1 (C-8), 36.3 (C-3), 32.8 (C-1$^{v'''}$), 27.0 (C-9), 15.8 (C-1$^{'v}$), 14.4 (C-1$^{x'''}$), 12.9 (C-1$^{x''}$), 11.6 (C-1$^{x'v}$), 9.4 (C-1$^{'x}$); HRMS *m/z* (ESI^+) [Found: 1219.4509, $C_{61}H_{72}ClN_{10}O_{11}S_2$ requires $[M+H]^+$ 1219.4507]; LRMS *m/z* (ESI^+) 1219 ($[^{35}M+H]^+$, 77%), 1221 ($[^{37}M+H]^+$, 40%), 1241 ($[^{35}M+Na]^+$, 78%), 1243 ($[^{37}M+Na]^+$, 43%); HPLC Retention time 9.7 min, 98.5%.

(2*S*,4*R*)-4-(Benzyloxy)-1-((*S*)-2-(*tert*-butyl)-17-((*S*)-4-(4-chlorophenyl)-2,3,9-trimethyl-6*H*-thieno[3,2-*f*][1,2,4]triazolo[4,3-*a*][1,4]diazepin-6-yl)-4,16-dioxo-6,9,12-trioxa-3,15-diazaheptadecan-1-oyl)-*N*-(4-(4-methylthiazol-5-yl)benzyl)pyrrolidine-2-carboxamide (8, Bn-VHL)

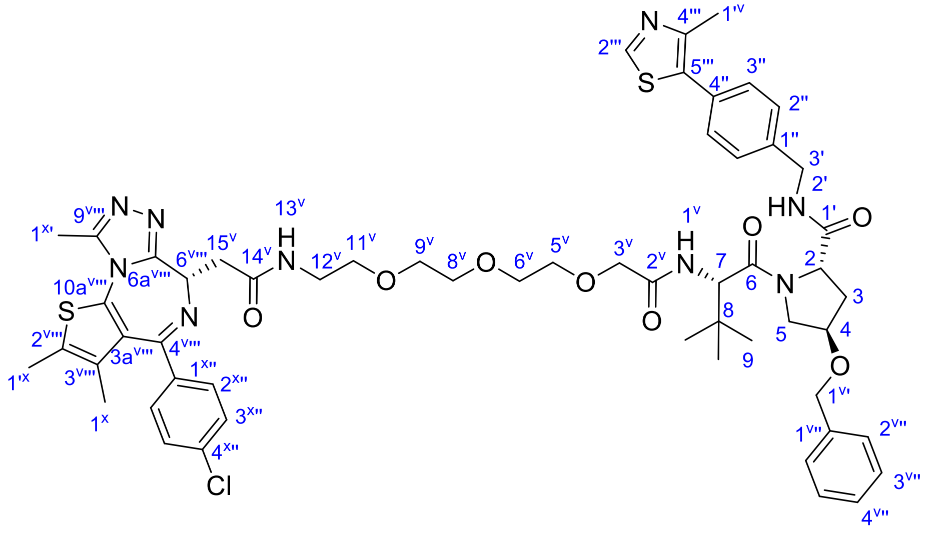

Step 1:

Compound **44** (78 mg, 0.11 mmol, 1.0 eq.) was dissolved in THF (1 mL) and PPh_3 (58 mg, 0.22 mmol, 2.0 eq.) was added. The reaction mixture was stirred for 10 min at rt, then water (20 µL, 1.10 mmol, 10.0 eq.) was added. The reaction was stirred at rt overnight. The volatile components were evaporated to afford the crude material as a yellow solid, which was used in the next step without further purification.

Step 2:

(+)-JQ1-COOH (40 mg, 0.10 mmol, 0.9 eq.) was dissolved in dry DMF (0.5 mL) and DIPEA (77 µL, 0.44 mmol, 4.0 eq.) followed by HATU (50 mg, 0.13 mmol, 1.2 eq.) were added under argon. After 10 min, the intermediate (0.11 mmol, 1.0 eq.) obtained in Step 1 in dry DMF (0.5 mL) was added, and the reaction mixture was stirred at rt overnight. After this time, water (5 mL) was added and the mixture was extracted with EtOAc (3 × 5 mL), then the aqueous phase was saturated with solid NaCl and the product was further extracted with EtOAc (3 × 5 mL). The combined organic phases were washed with 0.5 M aqueous LiCl solution (1 × 5 mL), dried over sodium sulfate, filtered, and concentrated *in vacuo*. The crude material was purified using silica gel column chromatography, eluting with EtOAc:CH_3OH (gradient 0 to 15% CH_3OH) to yield the title compound as a colourless solid (87 mg, 72%). The compound was further purified using semi-preparative HPLC, method A: R_f 0.17 (EtOAc:CH_3OH 85:15); m.p. 90–92 °C (from EtOAc:CH_3OH); $[\alpha]_D^{25}$ = +12.0 (*c* 1.0, CH_3OH); $\tilde{\nu}_{max}$ (neat)/cm^{-1} 3316

(N-H, w), 2921 (C-H, w), 2870 (C-H, w), 1644 (C=O, s), 1487 (N-H, s), 1434 (N-H, s), 1417 (N-H, s), 1091 (C-O, s), 1042 (C-O, w), 1014 (C-O, w); ^{1}H NMR (600 MHz; CD_3OD) δ_H 8.86 (1H, s, H-2'''), 7.44–7.37 (8H, m, H-2'', H-3'', H-2^{x}'', H-3^{x}''), 7.30–7.24 (5H, m, H-2^{v}'', H-3^{v}'', H-4^{v}''), 4.74 (1H, s, H-7), 4.61–4.55 (3H, m, H-2, H-6^{v}''', H-1$^{v'a}$), 4.53–4.46 (2H, m, H-1$^{v'b}$, H-3$^{'a}$), 4.34 (1H, d, *J* 15.4, H-3$^{'b}$), 4.33–4.29 (1H, m, H-4), 4.21 (1H, dd, *J* 11.4, 7.3, H-5^{a}), 4.07 (1H, d, *J* 15.6, H-3va), 3.99 (1H, d, *J* 15.6, H-3vb), 3.75 (1H, dd, *J* 11.4, 7.3, H-5^{b}), 3.69–3.62 (8H, m, H-5^{v}, H-6^{v}, H-8^{v}, H-9^{v}), 3.57 (2H, t, *J* 6.6, H-11^{v}), 3.45–3.41 (3H, m, H-12^{v}, H-15vb), 3.29–3.27 (1H, m, H-15va), 2.67 (3H, s, H-1$^{x'}$), 2.46 (3H, s, H-1$^{'v}$), 2.43–2.38 (4H, m, H-1$^{'x}$, H-3^{a}), 2.12–2.07 (1H, m, H-3^{b}), 1.68 (3H, s, H-1^{x}), 1.04 (9H, s, H-9); ^{13}C NMR (151 MHz; CD_3OD) δ_C 174.2 (C-1'), 172.9 (C-14^{v}), 172.2 (C-2^{v}), 171.8 (C-6), 166.1 (C-4^{v}'''), 157.0 (C-6^{v}'''), 152.8 (C-2'''), 152.1 (C-9^{v}'''), 149.1 (C-4'''), 140.2 (C-1''), 139.4 (C-1^{v}''), 138.1 (C-1^{x}''), 137.9 (C-4^{x}''), 133.5 (C-10a^{v}'''), 133.4 (C-5'''), 133.2 (C4''), 132.0 (C-3^{v}'''), 132.0 (C-2^{v}'''), 131.5 (C-3a^{v}'''), 131.3 (C-3^{x}''), 130.4 (C-2^{x}''), 129.8 (C-3''), 129.4 (C-2''), 129.0 (C-3^{v}''), 129.0 (C-2^{v}''), 128.7 (C-4^{v}''), 78.5 (C-4), 72.2 (C-5^{v}), 71.7 (2C, C-1^{v}', C-9^{v}), 71.5 (C-6^{v}), 71.4 (C-8^{v}), 71.1 (C-3^{v}), 70.7 (C-11^{v}), 60.8 (C-2), 58.1 (C-7), 55.2 (C-6^{v}'''), 54.7 (C-5), 43.7 (C-3'), 40.6 (C-12^{v}), 38.7 (C-15^{v}), 36.9 (C-8), 36.6 (C-3), 26.9 (C-9), 15.8 (C-1$^{'v}$), 14.4 (C-1^{x}), 12.9 (C-1$^{'x}$), 11.6 (C-1$^{x'}$); HRMS *m/z* (ESI^{+}) [Found: 1092.4244, $C_{56}H_{67}ClN_9O_8S_2$ requires $[M+H]^+$ 1092.4237]; LRMS *m/z* (ESI^{+}) 1092 ($[^{35}M+H]^+$, 88%), 1094 ($[^{37}M+H]^+$, 48%), 1114 ($[^{35}M+Na]^+$, 100%), 1116 ($[^{37}M+Na]^+$, 45%); HPLC Retention time 10.2 min, 98.4%.

2-(2,6-Dioxopiperidin-3-yl)-4-nitroisoindoline-1,3-dione (47)

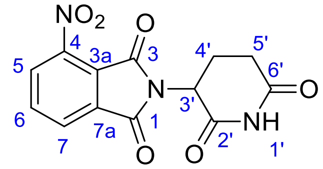

3-Nitrophthalic anhydride (3.66 g, 18.96 mmol, 1.2 eq.) was dissolved in acetic acid (50 mL) and sodium acetate (1.55 g, 18.96 mmol, 1.2 eq.) and 3-aminopiperidine-2,6-dione hydrogen chloride (2.60 g, 15.80 mmol, 1.0 eq.) were added sequentially. The resulting mixture was heated at 120 °C for 7 h. After this time, the reaction mixture was then cooled to rt and concentrated *in vacuo*. Cold water (20 mL) was added and the resulting precipitated was filtered *in vacuo* and washed with cold water (2 × 5 mL). The resulting solid was dried *in vacuo* to yield a pale grey solid (4.19 g, 88%): *Rf* 0.44 (PE/EtOAc 40:60); m.p. 270–272 °C (from water) [lit.[17] 256.5–257.5 °C]; ^{1}H NMR (400 MHz; D_6-DMSO) δ_H 11.17 (1H, s, N*H*-1'), 8.36 (1H, d, *J* 7.9, H-5), 8.25 (1H, d, *J* 7.2, H-7), 8.13 (1H, dd, *J* 7.9, 7.2, H-6), 5.21 (1H, dd, *J* 12.9,

5.4, H-3'), 2.90 (1H, ddd, *J* 19.6, 12.9, 5.4, H-5'[a]), 2.64–2.49 (2H, m, H-5'[b], H-4'[a]), 2.11–2.05 (1H, m, H-4'[b]); LRMS *m/z* (ESI$^-$) 302 ([M-H]$^-$, 100%). The spectroscopic data are in good agreement with the literature values.[17]

4-Amino-2-(2,6-dioxopiperidin-3-yl)isoindoline-1,3-dione (11)

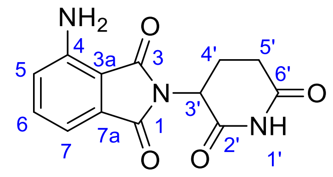

Intermediate **47** (2.00 g, 6.60 mmol, 1.0 eq.) was added to a round-bottom flask containing Pd/C 10% (0.20 g, 10% *w/w*) and DMF (40 mL). The reaction was flushed with hydrogen and the mixture was stirred overnight at rt. The mixture was filtered over a pad of Celite® and washed with EtOAc (3 × 10 mL). The solvent was removed *in vacuo*, and the dark green residue was then suspended in EtOAc and heated under reflux for 20 min, then left to cool to rt and further cooled to 0 °C. The solid precipitate was collected by vacuum filtration and further dried *in vacuo* to afford a yellow solid (1.64 g, 91%): *Rf* 0.45 (PE:EtOAc 40:60); m.p. >300 °C (from EtOAc) [lit.[18] 315.5–317.5° C]; ^{1}H NMR (400 MHz; D_6-DMSO) δ_H 11.08 (1H, s, N*H*-1'), 7.47 (1H, dd, *J* 7.0, 6.9, H-6), 7.03–6.99 (2H, m, H-5, H-7), 6.52 (2H, s, N*H_2*), 5.05 (1H, dd, *J* 12.8, 5.2, H-3'), 2.89 (1H, ddd, *J* 15.6, 12.8, 5.2, H-5'[a]), 2.61–2.49 (2H, m, H-5'[b], H-4'[a]), 2.06–2.00 (1H, m, H-4'[b]); LRMS *m/z* (ESI$^-$) 272 ([M−H]$^-$, 100%); HPLC Retention time 6.7 min, 99.9%. The spectroscopic data are in good agreement with the literature values.[18]

4-Amino-2-(1-(4-nitrobenzyl)-2,6-dioxopiperidin-3-yl)isoindoline-1,3-dione (10)

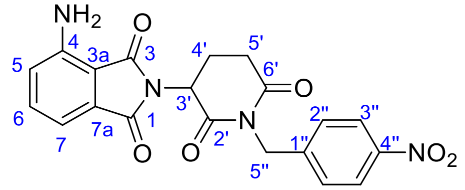

Compound **11** (0.30 g, 1.10 mmol, 1.0 eq.) was dissolved in dry THF (6 mL) and dry DMF (1 mL) and 4-nitrobenzyl alcohol (0.22 g, 1.43 mmol, 1.3 eq.) and PS-PPh_3 (0.37 g, 1.43 mmol, 1.3 eq.) were added. The mixture was cooled at 0 °C and DIAD (0.28 mL, 1.43 mmol, 1.3 eq.) was added slowly. After 1 h at 0 °C, the mixture was warmed to rt and stirred for further 16 h. The following day the reaction was not complete and so further PS-PPh_3 (0.3 eq.) and of DIAD (0.3 eq.) were added. After 20 h at rt, the reaction was filtered over a pad of Celite®, washed with EtOAc (2 × 5 mL) and the volatile components were removed *in vacuo*. Water (5 mL)

was added, and the aqueous phase was extracted with EtOAc (3 × 20 mL). The combined organic layers were dried over sodium sulfate, filtered, and evaporated. The crude material was purified using silica gel column chromatography, eluting with PE:EtOAc (gradient 10 to 30% EtOAc) afforded the title compound as a yellow solid (88 mg, 20%): *Rf* 0.54 (PE:EtOAc 40:60); m.p. 170–172 °C (from PE:EtOAc); $\tilde{\upsilon}_{max}$ (thin film)/cm^{-1} 3449 (N-H, s), 3358 (N-H, s), 2921 (C-H, w), 1729 (C=O, s), 1682 (C=O, s), 1518 (N-O, s), 1371 (s), 1333 (N-O, s), 1168 (s), 770 (C-H, s); ^{1}H NMR (600 MHz; D$_6$-DMSO) δ_H 8.19 (2H, d, *J* 8.8, H-3''), 7.54 (2H, d, *J* 8.8, H-2''), 7.46 (1H, dd, *J* 8.5, 7.0, H-6), 7.03–7.00 (2H, m, H-5, H-7), 6.54 (2H, s, N*H*$_2$), 5.29 (1H, dd, *J* 13.0, 5.4, H-3'), 5.02 (1H, d, *J* 15.7, H-5''^{a}), 4.95 (1H, d, *J* 15.7, H-5''^{b}), 3.09 (1H, ddd, *J* 17.2, 13.0, 5.4, H-5'^{a}), 2.86–2.79 (1H, m, H-5'^{b}), 2.62 (1H, ddt, *J* 17.2, 13.0, 5.4, H-4'^{a}), 2.13–2.07 (1H, m, H-4'^{b}); ^{13}C NMR (151 MHz; D$_6$-DMSO) δ_C 171.7 (C-6'), 170.0 (C-2'), 168.5 (C-3), 166.7 (C-1), 147.4 (C-4), 146.1 (C-4''), 144.9 (C-1''), 136.4 (C-6), 131.9 (C-7a), 128.2 (C-2''), 123.4 (C-3''), 121.8 (C-5), 110.5 (C-7), 108.4 (C-3a), 48.6 (C-3'), 42.5 (C-5''), 31.1 (C-5'), 21.9 (C-4'); HRMS *m/z* (ESI$^-$) [Found: 407.0996, $C_{20}H_{15}N_4O_6$ requires [M−H]$^-$ 407.0997]; LRMS *m/z* (ESI$^-$) 407 ([M-H]$^-$, 100%); HPLC Retention time 9.8 min, 99.4%.

4-Amino-2-(1-((1-methyl-2-nitro-1*H*-imidazol-5-yl)methyl)-2,6-dioxopiperidin-3-yl)isoindoline-1,3-dione (12)

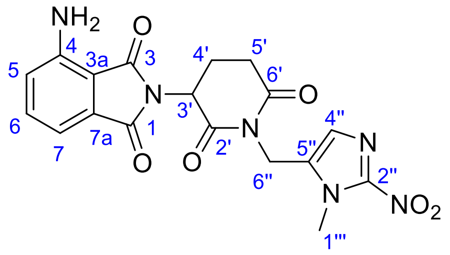

Compound **12** was synthesized as previously described for compound **10**, starting from pomalidomide **11** (0.20 g, 0.73 mmol, 1.0 eq.) and intermediate **22** (0.15 g, 0.95 mmol, 1.3 eq.). The reaction mixture was heated to 60 °C for 20 h. Then, the reaction was filtered over a pad of Celite®, washed with EtOAc (2 × 5 mL) and the volatile components were removed *in vacuo*. Water (10 mL) was added, and the product was extracted with EtOAc (3 × 20 mL). The combined organic layers were dried over sodium sulfate, filtered and evaporated. The crude material was purified using neutral alumina column chromatography, eluting with toluene:acetone (gradient 10 to 20% acetone) to afford the title compound as a pale yellow solid (61 mg, 20%): R_f 0.39 (toluene:acetone 80:20); m.p. 186–188 °C (from toluene:acetone); $\tilde{\upsilon}_{max}$ (neat)/cm^{-1} 3468 (N-H, s), 3364 (N-H, s), 2934 (C-H, w), 1731 (C=O, s), 1684 (C=O, s),

1533 (N-O, s), 1371 (s), 1328 (N-O, s), 1242 (s), 787 (C-H, s); ^{1}H NMR (600 MHz; D_6-DMSO) δ_H 7.53 (1H, dd, *J* 8.6, 6.9, H-6), 7.11 (1H, s, H-4''), 7.09–7.06 (2H, m, H-5, H-7), 6.59 (2H, s, N*H*$_2$), 5.33 (1H, dd, *J* 13.0, 5.4, H-3'), 5.03 (1H, d, *J* 14.7, H-6''^{a}), 4.96 (1H, d, *J* 14.7, H-6''^{b}), 4.00 (3H, s, H-1'''), 3.10 (1H, ddd, *J* 17.3, 13.0, 5.4, H-5'^{a}), 2.91–2.85 (1H, m, H-5'^{b}), 2.65 (1H, ddt, *J* 17.5, 13.0, 5.4, H-4'^{a}), 2.17–2.12 (1H, m, H-4'^{b}); ^{13}C NMR (151 MHz; D_6-DMSO) δ_C 171.5 (C-6'), 169.8 (C-2'), 168.5 (C-3), 167.3 (C-1), 146.8 (C-4), 145.2 (C-2''), 135.5 (C-6), 134.3 (C-5''), 131.9 (C-7a), 127.1 (C-4''), 121.8 (C-5), 111.1 (C-7), 108.4 (C-3a), 49.0 (C-3'), 34.2 (C-1'''), 33.8 (C-6''), 31.1 (C-5'), 21.2 (C-4'); HRMS *m/z* (ESI$^+$) [Found: 413.1204, $C_{18}H_{17}N_6O_6$ requires $[M+H]^+$ 413.1204]; LRMS *m/z* (ESI$^+$) 304, (100%), 413 ($[M+H]^+$, 36%), 435 ($[M+Na]^+$, 20%); HPLC Retention time 8.2 min, 96.9%.

2,6-Bis(benzyloxy)pyridine (49)

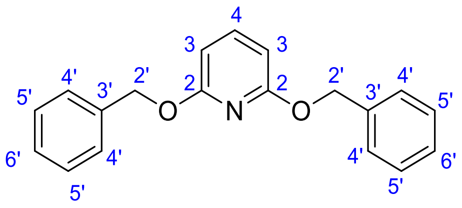

Benzyl alcohol (2.11 mL, 20.28 mmol, 3.0 eq.) was dissolved in dry DMF (27 mL) and the solution was cooled to 0 °C under a stream of argon. NaH (60% mineral oil, 1.08 g, 27.04 mmol, 4.0 eq.) was added portion wise and, once gas evolution had ceased, the reaction mixture was warmed to rt and stirred for 30 min. 2,6-Dichloropyridine (1.00 g, 6.76 mmol, 1.0 eq.) was then added and the reaction mixture was heated at 80 °C for 16 h. The reaction was cooled to rt and quenched by addition of water (30 mL). The precipitate formed was filtered *in vacuo*, washed with water (1 × 20 mL), and dried *in vacuo* to yield a yellow solid (1.92 g, 98%): R_f 0.54 (PE:EtOAc 98:2); m.p. 55–57 °C (from water); $\tilde{\nu}_{max}$ (neat)/cm^{-1} 2921 (C-H, w), 2887 (C-H, w), 1616 (w), 1591 (w), 1453 (C-O, s), 1224 (s), 1078 (w), 788 (C-H, s) 732 (C-H, s); ^{1}H NMR (400 MHz; $CDCl_3$) δ_H 7.54 (1H, t, *J* 7.9, H-4), 7.50–7.47 (4H, m, H-4'), 7.43–7.36 (6H, m, H-5', H-6'), 6.44 (2H, d, *J* 7.9, H-3), 5.40 (4H, s, H-2'); ^{13}C NMR (101 MHz; $CDCl_3$) δ_C 162.4 (C-2), 141.2 (C-4), 137.7 (C-3'), 128.5 (C-5'), 127.9 (C-4'), 127.8 (C-6'), 102.1 (C-3), 67.7 (C-2'); HRMS *m/z* (ESI$^+$) [Found: 292.1330, $C_{19}H_{18}NO_2$ requires $[M+H]^+$ 292.1332]; LRMS *m/z* (ESI$^+$) 292 ($[M+H]^+$, 28%), 380 (100%); HPLC Retention time 11.9 min, 97.0%. The spectroscopic data are in good agreement with the literature values.[5]

2,6-Bis(benzyloxy)-3-bromopyridine (50)

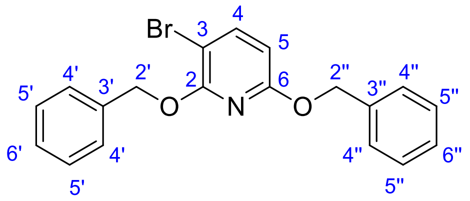

To a stirred solution of 2,6-bis(benzyloxy)pyridine (1.00 g, 3.43 mmol, 1.0 eq.) in acetonitrile (10 mL), *N*-bromosuccinimide (0.49 g, 2.74 mmol, 0.8 eq.) was added portion wise, and the reaction mixture was heated at 80 °C for 3 h. After this time, the reaction mixture was cooled to rt and the volatile components were evaporated *in vacuo*. EtOAc (25 mL) was added, and the organic phase was washed with water (1 × 25 mL). The organic phase was dried over sodium sulfate, filtered, and concentrated *in vacuo*. Purification using silica gel column chromatography, eluting with PE:EtOAc (gradient 0 to 5% EtOAc) yielded compound **50** as a colourless solid (820 mg, 64%): R_f 0.54 (PE:EtOAc 98:2); m.p. 45–47 °C (from PE:EtOAc); $\tilde{\upsilon}_{max}$ (neat)/cm^{-1} 2942 (C-H, w), 1604 (w), 1441 (C-O, s), 1378 (s), 1120 (w), 825 (w) 760 (C-H, s); ^{1}H NMR (400 MHz; $CDCl_3$) δ_H 7.68 (1H, d, *J* 8.3, H-4), 7.46–7.31 (10H, m, H-4', H-5', H-6', H-4'', H-5'', H-6''), 6.32 (1H, d, *J* 8.3, H-5), 5.43 (2H, s, H-2'), 5.31 (2H, s, H-2''); ^{13}C NMR (101 MHz; $CDCl_3$) δ_C 161.3 (C-2), 157.8 (C-6), 144.1 (C-4), 137.3 (C-3''), 137.2 (C-3'), 128.6 (2C, C-5', C-5''), 127.9 (C-4''), 128.0 (C-6''), 127.8 (C-6'), 127.4 (C-4'), 103.9 (C-5), 96.9 (C-3), 68.4 (C-2''), 68.3 (C-2'); HRMS *m/z* (ESI$^+$) [Found: 370.0437 and 372.0416, $C_{19}H_{17}BrNO_2$ requires [M+H]$^+$ 370.0437 and 372.0417]; LRMS *m/z* (ESI$^+$) 370 ([^{79}M+H]$^+$, 93%), 372 ([^{81}M+H]$^+$, 93%), 789 (100%); HPLC Retention time 12.6 min, 93.0%. The spectroscopic data are in good agreement with the literature values.[5]

4-(2,6-Bis(benzyloxy)pyridin-3-yl)phenol (51)

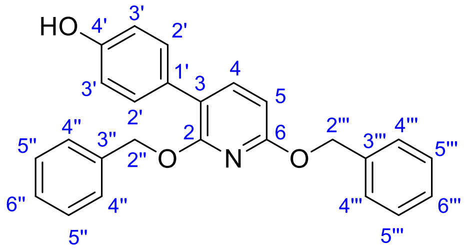

2,6-Bis(benzyloxy)-3-bromopyridine (1.00 g, 2.70 mmol, 1.0 eq.), (4-hydroxyphenyl)boronic acid (0.74 g, 5.40 mmol, 2.0 eq.) and potassium phosphate tribasic (1.20 g, 5.67 mmol, 2.1 eq.) in a 6:1 mixture of dioxane (30 mL) and water (5 mL) were degassed under argon and 1,1'-bis(diphenylphosphino)ferrocene-palladium(II)dichloride dichloromethane complex (220 mg,

0.27 mmol, 0.1 eq.) was then added. The reaction mixture was stirred at 100 °C for 16 h, cooled to rt, filtered over a pad of Celite®, and washed with EtOAc (2 × 50 mL). The filtrate was then diluted with water (150 mL) and the product was extracted with EtOAc (1 × 200 mL). The combined organic phases were dried over sodium sulfate, filtered, and concentrated *in vacuo*. Purification using silica gel column chromatography, eluting with PE:EtOAc (gradient 10 to 20% EtOAc) yielded compound **51** as a pale pink solid (910 mg, 88%): R_f 0.58 (PE:EtOAc 70:30); m.p. 113–115 °C (from PE:EtOAc); $\tilde{\upsilon}_{max}$ (neat)/cm^{-1} 3263 (O-H, w), 1598 (C-O, s), 1470 (C-O, s), 1356 (w), 1242 (s), 1005 (w), 841 (C-H, w), 697 (C-H, w); ^{1}H NMR (400 MHz; $CDCl_3$) δ_H 7.57 (1H, d, *J* 8.1, H-4), 7.46–7.42 (4H, m, H-3', H-6'', H-6'''), 7.39–7.33 (4H, m, H-4'', H-4'''), 7.32–7.27 (4H, m, H-5'', H-5'''), 6.85 (2H, d, *J* 8.6, H-2'), 6.36 (1H, d, *J* 8.1, H-5), 5.42 (2H, s, H-2'''), 5.36 (2H, s, H-2''), 4.74 (1H, br s, O*H*); ^{13}C NMR (101 MHz; $CDCl_3$) δ_C 161.0 (C-6), 158.2 (C-4'), 154.4 (C-2), 141.5 (C-4), 137.9 (C-3'''), 137.6 (C-3''), 130.3 (C-2'), 129.5 (C-1'), 128.5 (C-5'''), 128.3 (C-5''), 127.8 (2C, C-6'', C-6'''), 127.4 (C-4''), 127.2 (C-4'''), 115.7 (C-3), 115.1 (C-3'), 102.4 (C-5), 67.8 (C-2''), 67.6 (C-2'''); HRMS *m/z* (ESI$^+$) [Found: 384.1597, $C_{25}H_{22}NO_3$ requires $[M+H]^+$ 384.1594]; LRMS *m/z* (ESI$^+$) 384 ($[M+H]^+$, 100%), 406 ($[M+Na]^+$, 34%); HPLC Retention time 11.7 min, 93.7%. The spectroscopic data are in good agreement with the literature values.[5]

***tert*-Butyl 2-(4-(2,6-bis(benzyloxy)pyridin-3-yl)phenoxy)acetate (52)**

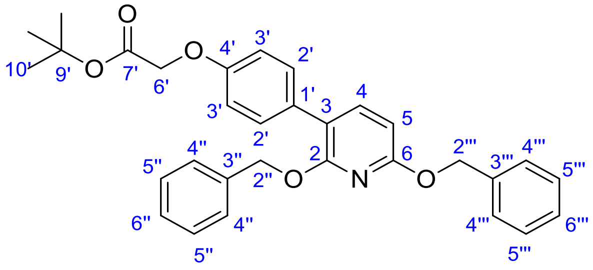

4-(2,6-Bis(benzyloxy)pyridin-3-yl)phenol (790 mg, 2.06 mmol, 1.0 eq.) was dissolved in dry DMF (19 mL) and K_2CO_3 (427 mg, 3.09 mmol, 1.5 eq.) was added. The mixture was stirred at rt for 10 min, and then *tert*-butyl bromoacetate (319 µL, 2.16 mmol, 1.05 eq.) was added dropwise. The reaction mixture was stirred at rt for 3 h, then diluted with water (80 mL) and the product was extracted with EtOAc (2 × 100 mL). The combined organic phases were dried over sodium sulfate, filtered, and concentrated *in vacuo*. Purification using silica gel column chromatography, eluting with PE:EtOAc (gradient 2 to 10% EtOAc) yielded compound **5** as a colourless solid (866 mg, 84%): R_f 0.39 (PE:EtOAc 90:10); m.p. 65–67 °C (from PE:EtOAc); $\tilde{\upsilon}_{max}$ (neat)/cm^{-1} 2918 (C-H, w), 1752 (C=O, s), 1470 (C-O, s), 1452 (C-O, s), 1240 (C-O, s),

1223 (C-O, s), 1165 (w), 1047 (w), 825 (w); ^{1}H NMR (400 MHz; $CDCl_3$) δ_H 7.46 (1H, d, *J* 8.0, H-4), 7.39 (2H, d, *J* 8.8, H-3'), 7.34–7.32 (2H, m, H-6'', H-6'''), 7.28–7.18 (8H, m, H-4'', H-5'', H-4''', H-5'''), 6.82 (2H, d, *J* 8.8, H-2'), 6.36 (1H, d, *J* 8.0, H-5), 5.31 (2H, s, H-2''), 5.26 (2H, s, H-2'''), 4.43 (2H, s, H-6'), 1.39 (9H, s, H-10'); ^{13}C NMR (101 MHz; $CDCl_3$) δ_C 168.2 (C-7'), 161.1 (C-6), 158.3 (C-4'), 157.0 (C-2), 141.6 (C-4), 138.0 (C-3'''), 137.7 (C-3''), 130.2 (C-2'), 130.1 (C-1'), 128.6 (C-5'''), 128.5 (C-5''), 127.9 (2C, C-6'', C-6'''), 127.5 (C-4'''), 127.3 (C-4''), 115.7 (C-3), 114.4 (C-3'), 102.5 (C-5), 82.4 (C-9'), 67.9 (C-2'''), 67.7 (C-2''), 65.9 (C-6'), 28.2 (C-10'); HRMS *m/z* (ESI^+) [Found: 498.2271, $C_{31}H_{32}NO_5$ requires $[M+H]^+$ 498.2275]; LRMS *m/z* (ESI^+) 498 ($[M+H]^+$, 79%), 520 ($[M+Na]^+$, 90%); HPLC Retention time 13.3 min, 98.6%. The spectroscopic data are in good agreement with the literature values.[5]

tert-Butyl 2-(4-(2,6-dioxopiperidin-3-yl)phenoxy)acetate (53)

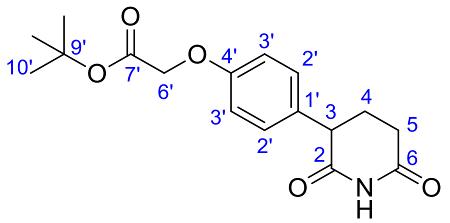

tert-Butyl 2-(4-(2,6-bis(benzyloxy)pyridin-3-yl)phenoxy)acetate **52** (855 mg, 1.72 mmol, 1.0 eq.) was added to a round-bottom flask containing Pd/C 10% (86 mg, 10% *w/w*) and DMF (12 mL). The reaction mixture was flushed with hydrogen and then stirred overnight at rt. The mixture was filtered over a pad of Celite® and washed with EtOAc (2 × 20 mL). The solvent was removed *in vacuo* and the crude material was purified using silica gel column chromatography, eluting with PE:EtOAc (gradient 30 to 60% EtOAc) yielding compound **53** as a colourless solid (504 mg, 92%): R_f 0.43 (PE:EtOAc 40:60); m.p. 60–62 °C (from PE:EtOAc); $\tilde{\upsilon}_{max}$ (neat)/cm^{-1} 3194 (N-H, w), 2979 (C-H, w), 1722 (C=O, s), 1712 (C=O, s), 1701 (C=O, s), 1513 (C-O, s), 1368 (w), 1154 (C-O, s), 831 (w); ^{1}H NMR (400 MHz; $CDCl_3$) δ_H 8.36 (1H, br s, N*H*), 7.11 (2H, d, *J* 8.7, H-2'), 6.88 (2H, d, *J* 8.7, H-3'), 4.49 (2H, s, H-6'), 3.72 (1H, dd, *J* 9.6, 5.2, H-3), 2.67 (1H, ddd, *J* 17.7, 12.4, 5.2, H-5^a), 2.62 (1H, ddd, *J* 17.7, 12.4, 5.2, H-5^b), 2.28–2.13 (2H, m, H-4^a, H-4^b), 1.48 (9H, s, H-10'); ^{13}C NMR (101 MHz; $CDCl_3$) δ_C 173.4 (C-2), 172.5 (C-6), 167.9 (C-7'), 157.5 (C-4'), 130.0 (C-1'), 129.2 (C-2'), 115.1 (C-3'), 82.5 (C-9'), 65.8 (C-6'), 47.1 (C-3), 30.8 (C-5), 28.1 (C-10'), 26.4 (C-4); HRMS *m/z* (ESI^+) [Found: 342.1317, $C_{17}H_{21}NNaO_5$ requires $[M+Na]^+$ 342.131]; LRMS *m/z* (ESI^+) 342 ($[M+Na]^+$, 100%); HPLC Retention time 8.3 min, 91.6%. The spectroscopic data are in good agreement with the literature values.[5]

2-(4-(2,6-Dioxopiperidin-3-yl)phenoxy)acetic acid (54)

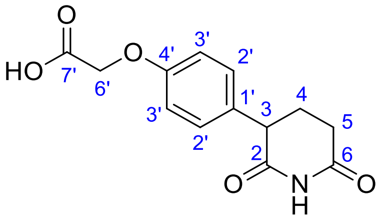

tert-Butyl 2-(4-(2,6-dioxopiperidin-3-yl)phenoxy)acetate **53** (288 mg, 0.90 mmol, 1.0 eq.) was dissolved in CH_2Cl_2 (9 mL) and trifluoroacetic acid (2.7 mL) was added. The mixture was stirred at rt overnight, then concentrated *in vacuo* to afford the title compound as a pale pink solid (229 mg, 97%): R_f 0.13 (EtOAc/CH_3OH 80:20); m.p. 190–192 °C (from CH_2Cl_2); $\tilde{\upsilon}_{max}$ (neat)/cm^{-1} 3197 (N-H, w), 3086 (O-H, w), 2918 (C-H, w), 1765 (C=O, s), 1706 (C=O, s), 1665 (C=O, s), 1515 (C-O, s), 1280 (w), 1195 (C-O, s), 1182 (C-O, s), 1084 (w), 835 (s); ^{1}H NMR (400 MHz; CD_3OD) δ_H 7.18 (2H, d, *J* 8.7, H-2'), 6.93 (2H, d, *J* 8.7, H-3'), 4.66 (2H, s, H-6'), 3.81 (1H, dd, *J* 9.8, 5.9, H-3), 2.74–2.58 (2H, m, H-5^a, H-5^b), 2.27–2.15 (2H, m, H-4^a, H-4^b); ^{13}C NMR (101 MHz; CD_3OD) δ_C 175.2 (C-2), 174.3 (C-6), 171.2 (C-7'), 157.3 (C-4'), 131.3 (C-1'), 129.2 (C-2'), 114.4 (C-3'), 62.7 (C-6'), 47.0 (C-3), 30.7 (C-5), 26.4 (C-4); HRMS *m/z* (ESI^-) [Found: 262.0719, $C_{13}H_{12}NO_5$ requires $[M-H]^-$ 262.0721]; LRMS *m/z* (ESI^-) 262 ($[M-H]^-$, 61%), 525 (100%); HPLC Retention time 5.2 min, 89.3%. The spectroscopic data are in good agreement with the literature values.[5]

***tert*-Butyl (5-(2-(4-(2,6-dioxopiperidin-3-yl)phenoxy)acetamido)pentyl)carbamate (55)**

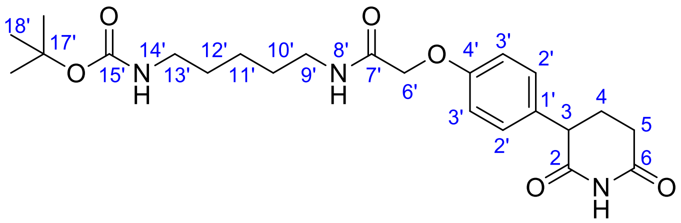

Compound **54** (150 mg, 0.57 mmol, 1.0 eq.) was dissolved in dry DMF (3 mL) and DIPEA (496 µL, 2.85 mmol, 5.0 eq.), HATU (433 mg, 1.14 mmol, 2.0 eq.) and then *N*-Boc-cadaverine (142 µL, 0.68 mmol, 1.2 eq.) were added under argon. The reaction was stirred at rt overnight. After this time, water (20 mL) was added and the mixture was extracted with CH_2Cl_2 (2 × 20 mL). The combined organic phases were dried over sodium sulfate, filtered, and concentrated *in vacuo*. The solvent was removed *in vacuo* and the crude material was purified using silica gel column chromatography, eluting with CH_2Cl_2:CH_3OH (gradient 0 to 10% CH_3OH) yielding compound **55** as a colourless solid (192 mg, 75%): R_f 0.24 (100% EtOAc);

m.p. 90–92 °C (from PE:EtOAc); $\tilde{\upsilon}_{max}$ (neat)/cm^{-1} 3351 (N-H, w), 3078 (N-H, w), 2932 (C-H, w), 2853 (C-H, w), 1721 (C=O, s), 1699 (C=O, s), 1683 (C=O, s), 1661 (C=O, s), 1518 (C-O, s), 1364 (w), 1234 (C-O, s), 1175 (C-O, s), 845 (s); ^{1}H NMR (400 MHz; CD_3OD) δ_H 8.11 (1H, br s, N*H*-14'), 7.20 (2H, d, *J* 8.6, H-2'), 6.97 (2H, d, *J* 8.6, H-3'), 4.49 (2H, s, H-6'), 3.81 (1H, dd, *J* 10.3, 5.6, H-3), 3.27 (2H, q, *J* 7.0, H-13'), 3.00 (2H, t, *J* 7.0, H-9'), 2.67 (1H, ddd, *J* 17.6, 10.3, 5.6, H-5^a), 2.62 (1H, ddd, *J* 17.6, 10.3, 5.6, H-5^b), 2.27–2.14 (2H, m, H-4^a, H-4^b), 1.57–1.45 (4H, m, H-10', H-12'), 1.42 (9H, s, 18'), 1.34–1.26 (2H, m, H-11'); ^{13}C NMR (101 MHz; CD_3OD) δ_C 176.6 (C-2), 175.7 (C-6), 171.0 (C-7'), 158.6 (C-15'), 158.4 (C-4'), 133.1 (C-1'), 130.8 (C-2'), 116.0 (C-3'), 79.8 (C-17'), 68.3 (C-6'), 47.0 (C-3), 41.2 (C-13'), 40.0 (C-9'), 32.2 (C-10'), 30.6 (C-5), 30.1 (C-12'), 28.8 (C-18'), 27.8 (C-4), 25.1 (C-11'); HRMS *m/z* (ESI$^-$) [Found: 446.2296, $C_{23}H_{32}N_3O_6$ requires [M−H]$^-$ 446.2297]; LRMS *m/z* (ESI$^-$) 446 ([M−H]$^-$, 100%); HPLC Retention time 7.7 min, 97.9%. The spectroscopic data are in good agreement with the literature values.[5]

2-((*S*)-4-(4-Chlorophenyl)-2,3,9-trimethyl-6*H*-thieno[3,2-*f*][1,2,4]triazolo[4,3-*a*][1,4]diazepin-6-yl)-*N*-(5-(2-(4-(2,6-dioxopiperidin-3-yl)phenoxy)acetamido)pentyl)acetamide (17, PG-4c)

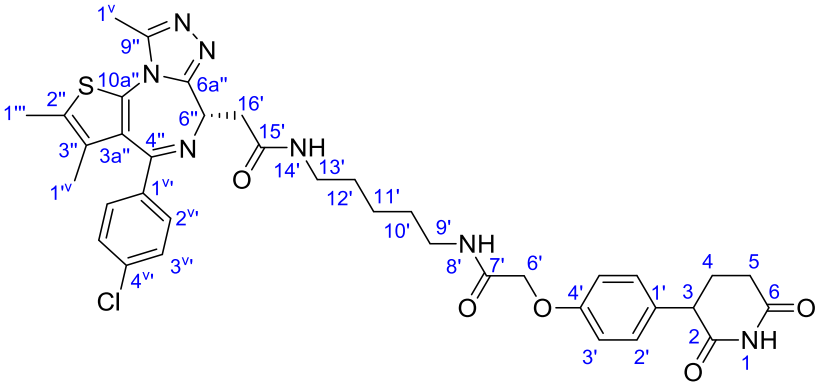

Step 1:

Compound **55** (51 mg, 0.11 mmol, 1.0 eq.) was dissolved in CH_2Cl_2 (0.5 mL) and HCl (4 M in dioxane, 172 µL, 0.67 mmol 6.0 eq.) was added. The reaction was stirred for 1 h at rt. Then, the volatile components were evaporated to afford the crude material as a colourless solid, which was used in the next step without further purification.

Step 2:

The obtained intermediate (0.11 mmol, 1.0 eq.) was dissolved in dry DMF (1.2 mL) and DIPEA (100 µL, 0.57 mmol, 5.0 eq.), HATU (87 mg, 0.23 mmol, 2.0 eq.), and then (+)-JQ1-COOH (55 mg, 0.14 mmol, 1.2 eq.) were added under argon. The reaction mixture was stirred at rt

overnight. After this time, water (5 mL) was added and the mixture was extracted with EtOAc (6 × 5 mL). The combined organic phases were dried over sodium sulfate, filtered, and evaporated *in vacuo*. The crude material was purified using silica gel column chromatography, eluting with EtOAc:CH_3OH (gradient 0 to 10% CH_3OH) yielding compound **17** as a colourless solid (69 mg, 86%). The compound was further purified using semi-preparative HPLC, method A: R_f 0.41 (EtOAc:CH_3OH:NH_3 85:10:0.5); m.p. 117–119 °C (from EtOAc:CH_3OH); $[\alpha]_D^{25}$ = +11.0 (*c* 1.0, CH_3OH); $\tilde{\upsilon}_{max}$ (neat)/cm^{-1} 3335 (N-H, w), 3075 (N-H, w), 2928 (C-H, w), 2859 (C-H, w), 1702 (C=O, s), 1662 (C=O, s), 1536 (C-H, s), 1488 (C-H, s), 1230 (w), 1184 (C-O, s), 894 (C-H, w), 838 (C-H, w); ^1H NMR (600 MHz; CD_3OD) δ_H 7.43 (2H, d, *J* 8.5, H-2'^{v}), 7.39 (2H, d, *J* 8.5, H-3'^{v}), 7.15 (2H, d, *J* 8.8, H-2'), 6.91 (2H, d, *J* 8.8, H-3'), 4.63 (1H, dd, *J* 8.9, 5.5, H-6''), 4.48 (2H, s, H-6'), 3.79 (1H, dd, *J* 10.4, 5.4, H-3), 3.41 (1H, dd, *J* 14.8, 8.9 H-16'^{a}), 3.30–3.20 (5H, m, H-13', H-9', H-16'^{b}), 2.70–2.64 (4H, m, H-1^{v}, H-5^{a}), 2.61–2.56 (1H, m, H-5^{b}), 2.44 (3H, s, H-1'''), 2.20–2.12 (2H, m, H-4^{a}, H-4^{b}), 1.69 (3H, s, H-1'^{v}), 1.61–1.55 (4H, m, H-10', H-12'), 1.40–1.36 (2H, m, H-11'); ^{13}C NMR (151 MHz; CD_3OD) δ_C 176.5 (C-2), 175.7 (C-6), 172.7 (C-15'), 171.1 (C-7'), 166.2 (C-4''), 158.3 (C-4'), 157.0 (C-6a''), 152.2 (C-9''), 138.1 (C-4$^{v'}$), 138.0 (C-1$^{v'}$), 133.5 (C-10a''), 133.2 (C-3a''), 133.1 (C-1'), 132.0 (C-3''), 132.0 (C-2''), 131.3 (C-2$^{v'}$), 130.7 (C-2'), 129.8 (C-3$^{v'}$), 115.9 (C-3'), 68.3 (C-6'), 55.3 (C-6''), 48.4 (C-3), 40.3 (C-9'), 39.8 (C-13'), 38.9 (C-16'), 32.2 (C-5), 30.0 (C-12'), 29.9 (C-10'), 27.8 (C-4), 25.0 (C-11'), 14.4 (C-1'^{v}), 12.9 (C-1'''), 11.6 (C-1^{v}); HRMS *m/z* (ESI^+) [Found: 730.2567, $C_{37}H_{41}ClN_7O_5S$ requires $[M+H]^+$ 730.2573]; LRMS *m/z* (ESI^+) 730 ($[^{35}M+H]^+$, 100%), 732 ($[^{37}M+H]^+$, 86%), 752 ($[^{35}M+Na]^+$, 72%), 754 ($[^{37}M+Na]^+$, 45%); HPLC Retention time 8.3 min, 97.0%; The spectroscopic data are in good agreement with the literature values.[5]

***tert*-Butyl (5-(2-(4-(1-((1-methyl-2-nitro-1*H*-imidazol-5-yl)methyl)-2,6-dioxopiperidin-3-yl)phenoxy)acetamido)pentyl)carbamate (56)**

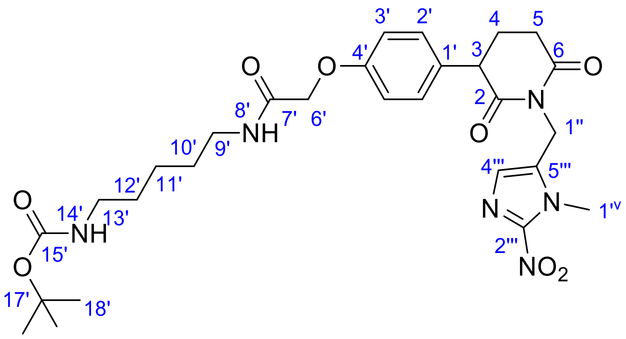

Compound **55** (72 mg, 0.16 mmol, 1.0 eq.) was dissolved in dry DMF (2 mL) and Cs_2CO_3 (63 mg, 0.19 mmol, 1.2 eq.) and TBAI (12 mg, 0.03 mmol, 0.2 eq.) were added under an argon

atmosphere. After 10 min, 2-nitroimidazol bromide **23** (39 mg, 0.18 mmol, 1.1 eq.) was added. The reaction mixture was stirred at rt for 6 h. After this time, the reaction was incomplete and so further Cs_2CO_3 (26 mg, 0.08 mmol, 0.5 eq.) and compound **23** (17 mg, 0.08 mmol, 0.5 eq.) were added and the reaction mixture was stirred at rt for additional 16 h. After this time, water (5 mL) was added and the mixture was extracted with EtOAc (6 × 5 mL). The combined organic phases were washed with 0.5 M aqueous LiCl solution (1 × 5 mL), dried over sodium sulfate, filtered, and concentrated *in vacuo*. The crude material was purified using silica gel column chromatography, eluting with PE:EtOAc (gradient 60 to 90% EtOAc) yielding compound **56** as a yellow solid (37 mg, 39%): R_f 0.47 (EtOAc 100%); m.p. 46–47 °C (from PE:EtOAc); $\tilde{\upsilon}_{max}$ (neat)/cm^{-1} 3322 (N-H, w), 2929 (C-H, w), 2858 (C-H, w), 1679 (C=O, s), 1531 (N-H, w), 1512 (N-H, w), 1487 (C-O, s), 1365 (s), 1273 (w), 1246 (C-O, s), 1156 (C-O, s), 731 (s); ^{1}H NMR (400 MHz; $CDCl_3$) δ_H 7.18 (1H, s, H-4'''), 7.10 (2H, d, *J* 8.6, H-3'), 6.93 (2H, d, *J* 8.6, H-2'), 6.54 (1H, br t, *J* 6.9, N*H*-14'), 5.04 (1H, d, *J* 15.1, H-1''^{b}), 5.00 (1H, d, *J* 15.1, H-1''^{a}), 4.54 (1H, br s, N*H*-8'), 4.47 (2H, s, H-6'), 4.11 (3H, s, H1^{iv}), 3.80 (1H, dd, *J* 10.3, 5.2, H-3), 3.34 (2H, q, *J* 7.0, H-13'), 3.08 (2H, q, *J* 7.0, H-9'), 2.89 (1H, dt, *J* 13.0, 5.2, H-5^a), 2.76 (1H, ddd, *J* 13.0, 10.3, 5.2, H-5^b), 2.28–2.18 (2H, m, H-4^a, H-4^b), 1.56 (2H, quint, *J* 7.0, H-12'), 1.49 (2H, quint, *J* 7.0, H-10'), 1.43 (9H, s, 18'), 1.32 (2H, quint, *J* 7.0, H-11'); ^{13}C NMR (126 MHz; $CDCl_3$) δ_C 173.5 (C-2), 172.0 (C-6), 168.0 (C-7'), 157.0 (C-4'), 156.2 (C-15'), 145.5 (C-2'''), 133.8 (C-5'''), 130.9 (C-1'), 130.0 (C-4'''), 129.6 (C-3'), 115.4 (C-2'), 79.3 (C-17'), 67.6 (C-6'), 48.2 (C-3), 40.5 (C-9'), 39.0 (C-13'), 34.5 (C-1^{iv}), 32.8 (C-1''), 32.0 (C-5), 29.8 (C-10'), 29.4 (C-12'), 28.6 (C-18'), 25.1 (C-4), 24.1 (C-11'); HRMS *m/z* (ESI$^-$) [Found: 585.2666, $C_{28}H_{37}N_6O_8$ requires [M−H]$^-$ 585.2678]; LRMS *m/z* (ESI$^-$) 585 ([M−H]$^-$, 100%); HPLC Retention time 9.4 min, 88.9%.

***tert*-Butyl (5-(2-(4-(1-((5-methoxy-1,2-dimethyl-4,7-dioxo-4,7-dihydro-1*H*-indol-3-yl)methyl)-2,6-dioxopiperidin-3-yl)phenoxy)acetamido)pentyl)carbamate (57)**

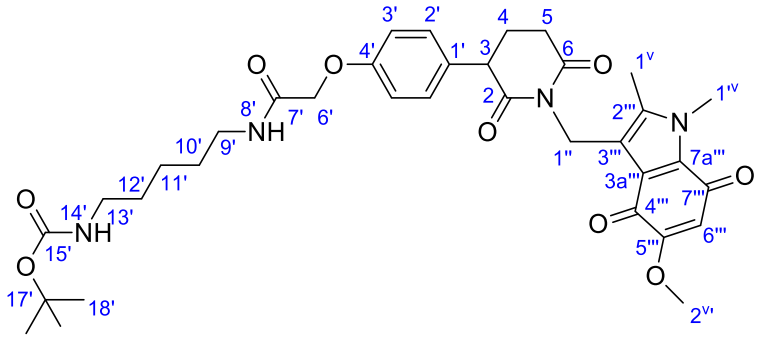

Compound **57** was synthesized as previously described for compound **56**, starting from compound **55** (62 mg, 0.14 mmol, 1.0 eq.) and indolequinone bromide **31** (45 mg, 0.15 mmol, 1.1 eq.). The reaction was stirred at rt for 16 h, then further Cs_2CO_3, (0.4 eq.), indolequinone bromide **31** (0.3 eq.), and TBAI (0.1 eq.) were added and the reaction mixture was stirred at rt for an additional 8 h. After this time, water (5 mL) was added and the mixture was extracted with EtOAc (6 × 5 mL). The combined organic phases were dried over sodium sulfate, filtered, and concentrated *in vacuo*. The crude material was purified using silica gel column chromatography, eluting with PE:EtOAc (gradient 60 to 100% EtOAc) yielding compound **57** as an orange solid (21 mg, 23%): R_f 0.41 (EtOAc:CH_3OH 90:10); m.p. 55–56 °C (from PE:EtOAc); $\tilde{\upsilon}_{max}$ (neat)/cm^{-1} 3339 (N-H, w), 2928 (C-H, w), 2860 (C-H, w), 1674 (C=O, s), 1636 (C=O, s), 1511 (N-H, w), 1244 (C-O, s), 1221 (C-O, s), 1175 (C-O, s), 837 (s); ^{1}H NMR (600 MHz; $CDCl_3$) δ_H 7.12 (2H, d, *J* 8.8, H-3'), 6.87 (2H, d, *J* 8.8, H-2'), 6.53 (1H, br t, *J* 6.7, N*H*-14'), 5.59 (1H, s, H-6'''), 5.06 (1H, d, *J* 14.7, H-1''^{b}), 4.98 (1H, d, *J* 14.7, H-1''^{a}), 4.45 (2H, s, H-6'), 3.99 (1H, br s, N*H*-8'), 3.87 (3H, s, H-1^{iv}), 3.80–3.77 (4H, m, H-3, H-2$^{v\prime}$), 3.34 (2H, q, *J* 6.7, H-13'), 3.07 (2H, q, *J* 6.7, H-9'), 2.86 (1H, dt, *J* 17.6, 5.1, H-5^a), 2.78 (1H, ddd, *J* 17.6, 9.6, 5.1, H-5^b), 2.27 (3H, s, H-1^v), 2.19–2.11 (2H, m, H-4^a, H-4^b), 1.55 (2H, quint, *J* 6.7, H-12'), 1.48 (2H, quint, *J* 6.7, H-10'), 1.43 (9H, s, 18'), 1.32 (2H, quint, *J* 6.7, H-11'); ^{13}C NMR (151 MHz; $CDCl_3$) δ_C 178.8 (C-4'''), 177.6 (C-7'''), 173.5 (C-2), 172.4 (C-6), 168.2 (C-7'), 159.8 (C-5'''), 156.6 (C-4'), 156.2 (C-15'), 137.0 (C-2'''), 132.2 (C-1'), 129.8 (C-3'), 129.0 (C-7a'''), 121.6 (C-3a'''), 117.7 (C-3'''), 115.1 (C-2'), 106.7 (C-6'''), 79.3 (C-17'), 67.6 (C-6'), 56.6 (C-2$^{v\prime}$), 48.3 (C-3), 40.6 (C-9'), 39.0 (C-13'), 35.3 (C-1''), 32.6 (C-1^{iv}), 32.0 (C-5), 29.8 (C-10'), 29.4 (C-12'), 28.6 (C-18'), 25.6 (C-4), 24.1 (C-11'), 9.8 (C-1^v); HRMS *m/z* (ESI$^+$) [Found: 665.3179, $C_{35}H_{45}N_4O_9$ requires $[M+H]^+$ 665.3181]; LRMS *m/z* (ESI$^+$) 665 ($[M+H]^+$, 29%), 687 ($[M+Na]^+$, 100%); HPLC Retention time 8.9 min, 77.7%.

***tert*-Butyl (5-(2-(4-(1-benzyl-2,6-dioxopiperidin-3-yl)phenoxy)acetamido)pentyl)carbamate (58)**

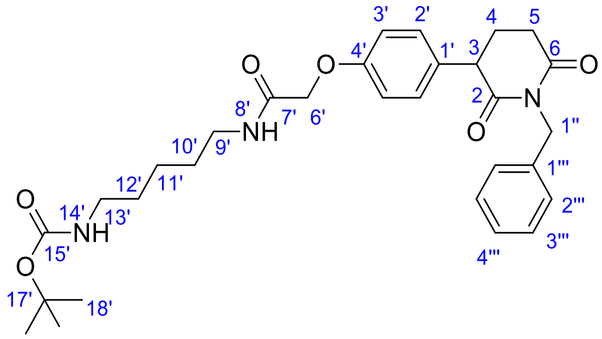

Compound **58** was synthesized as previously described for compound **56**, starting from compound **55** (92 mg, 0.20 mmol, 1.0 eq.) and benzyl bromide (26 µL, 0.22 mmol, 1.1 eq.). The reaction was stirred at rt for 16 h, then an further Cs_2CO_3 (of 0.5 eq.) and benzyl bromide (0.5 eq.) were added and the reaction mixture was stirred at rt for additional 16 h. After this time, water (5 mL) was added and the mixture was extracted with EtOAc (1 × 5 mL). The combined organic phases were dried over sodium sulfate, filtered, and concentrated *in vacuo*. The solvent was removed *in vacuo* and the crude material was purified using silica gel column chromatography, eluting with PE:EtOAc (gradient 60 to 100% EtOAc) yielding compound **58** as a colourless oil (43 mg, 40%): R_f 0.47 (EtOAc 100%); $\tilde{\upsilon}_{max}$ (neat)/cm^{-1} 3335 (N-H, w), 2927 (C-H, w), 2857 (C-H, w), 1673 (C=O, s), 1512 (N-H, w), 1364 (w), 1247 (w), 1165 (C-O, s), 702 (C-H, w); ^{1}H NMR (400 MHz; $CDCl_3$) δ_H 7.40–7.38 (2H, m, H-3'"), 7.31–7.24 (3H, m, H-2'", H-4'"), 7.07 (2H, d, *J* 8.6, H-3'), 6.87 (2H, d, *J* 8.6, H-2'), 6.54 (1H, br t, *J* 6.9, N*H*-14'), 5.02 (1H, d, *J* 13.4, H-1"^{b}), 4.98 (1H, d, *J* 13.4, H-1"^{a}), 4.57 (1H, br s, N*H*-8'), 4.46 (2H, s, H-6'), 3.78 (1H, dd, *J* 9.8, 5.2, H-3), 3.33 (2H, q, *J* 6.9, H-13'), 3.08 (2H, q, *J* 6.9, H-9'), 2.81 (1H, dt, *J* 17.6, 5.2, H-5^a), 2.71 (1H, ddd, *J* 17.6, 9.8, 5.2, H-5^b), 2.26–2.11 (2H, m, H-4^a, H-4^b), 1.55 (2H, quint, *J* 6.9, H-12'), 1.47 (2H, quint, *J* 6.9, H-10'), 1.43 (9H, s, 18'), 1.31 (2H, quint, *J* 6.9, H-11'); ^{13}C NMR (101 MHz; $CDCl_3$) δ_C 173.4 (C-2), 172.2 (C-6), 168.1 (C-7'), 156.7 (C-4'), 156.1 (C-15'), 137.3 (C-1'"), 131.8 (C-1'), 129.6 (C-3'), 129.2 (C-3'"), 128.5 (C-2'"), 127.7 (C-4'"), 115.1 (C-2'), 79.1 (C-17'), 67.6 (C-6'), 48.1 (C-3), 43.4 (C-1"), 40.5 (C-9'), 39.0 (C-13'), 31.9 (C-5), 29.8 (C-10'), 29.3 (C-12'), 28.6 (C-18'), 25.4 (C-4), 24.1 (C-11'); HRMS *m/z* (ESI$^-$) [Found: 536.2767, $C_{30}H_{38}N_3O_6$ requires [M−H]$^-$ 536.2766]; LRMS *m/z* (ESI$^-$) 536 ([M−H]$^-$, 100%); HPLC Retention time 9.7 min, 96.9%.

2-((*S*)-4-(4-Chlorophenyl)-2,3,9-trimethyl-6*H*-thieno[3,2-*f*][1,2,4]triazolo[4,3-*a*][1,4]diazepin-6-yl)-*N*-(5-(2-(4-(1-((1-methyl-2-nitro-1*H*-imidazol-5-yl)methyl)-2,6-dioxopiperidin-3-yl)phenoxy)acetamido)pentyl)acetamide (15, NI-CRBN)

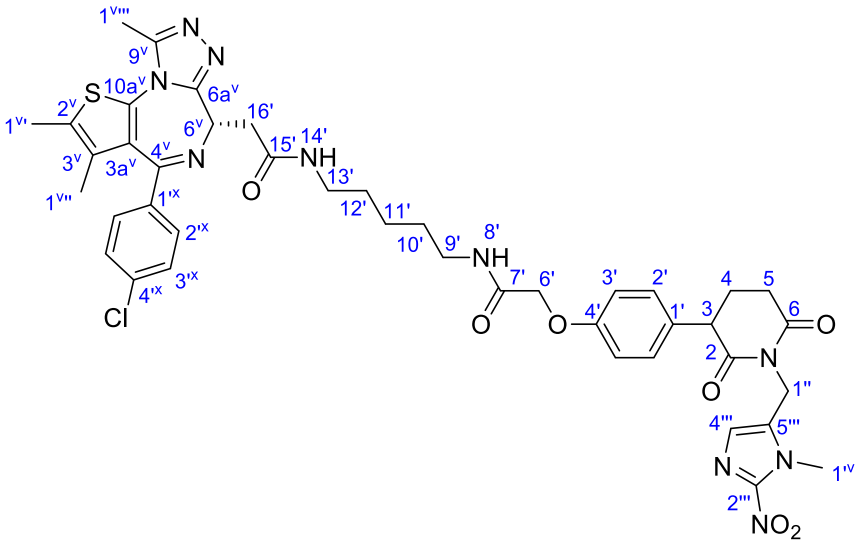

Step 1:

Compound **56** (33 mg, 0.06 mmol, 1.0 eq.) was dissolved in dry CH_3OH (1 mL) and oxalyl chloride (15 µL, 0.18 mmol, 3.0 eq.) was added while maintaining an argon atmosphere. After 3 h at rt, the reaction was complete and so further oxalyl chloride (3.0 eq.) was added and the reaction mixture was stirred at rt for 16 h. After this time, the volatile components were evaporated to afford the crude material as a yellow oil, which was used in the next step without further purification.

Step 2:

The obtained intermediate (0.06 mmol, 1.0 eq.) was added to a solution of (+)-JQ1-COOH (26 mg, 0.07 mmol, 1.1 eq.), DIPEA (63 µL, 0.36 mmol, 6.0 eq.) and HATU (46 mg, 0.12 mmol, 2.0 eq.) in dry DMF (1 mL) under argon. The reaction mixture was stirred at rt for 16 h, then diluted with water (15 mL) and extracted with EtOAc (3 × 15 mL). The combined organic layers were washed with 0.5 M aqueous LiCl solution (1 × 30 mL), dried over sodium sulfate, filtered, and evaporated *in vacuo*. The crude material was purified using silica gel column chromatography, eluting with EtOAc:CH_3OH (0 to 15% CH_3OH) to afford compound **15** as a yellow solid (26 mg, 50%). The compound was further purified using semi-preparative HPLC, method A: R_f 0.18 (EtOAc:CH_3OH 85:15); m.p. 94–96 °C (from EtOAc:CH_3OH); $[\alpha]_D^{25}$ = +7.6 (*c* 1.0, CH_3OH); $\tilde{\upsilon}_{max}$ (neat)/cm^{-1} 3307 (N-H, w), 2928 (C-H, w), 2857 (C-H, w), 1730 (C=O, s), 1534 (N-H, s), 1511 (N-H, w), 1487 (C-O, s), 1420 (s), 1371 (s), 1244 (w), 1191 (C-O, s), 1155 (w), 834 (C-H, s); ^{1}H NMR (400 MHz; CD_3OD) δ_H 7.44–7.37 (4H, m, H-

2'ix, H-3'ix), 7.11 (2H, d, *J* 8.8, H-3'), 7.06 (1H, s, H-4'''), 6.90 (2H, d, *J* 8.8, H-2'), 5.03 (2H, s, H-1''), 4.62 (1H, dd, *J* 5.8, 3.0, H-6v), 4.48 (2H, s, H-6'), 4.06 (3H, s, H-1'v), 3.91 (1H, dd, *J* 11.3, 4.9, H-3), 3.40 (1H, dd, *J* 5.8, 2.6, H-16'a), 3.29–3.19 (5H, m, H-9', H-13', H-16'b), 2.79 (1H, ddd, *J* 17.5, 7.0, 4.8, H-5a), 2.70–2.67 (4H, m, H-5b, H-1v'''), 2.44 (3H, s, H-1v'), 2.29–2.10 (2H, m, H-4a, H-4b), 1.68 (3H, s, H-1v''), 1.61–1.54 (4H, m, H-10', H-12'), 1.36 (2H, quint, *J* 6.2, H-11'); ^{13}C NMR (151 MHz; CD_3OD) δ_C 175.6 (C-2), 175.1 (C-6), 174.2 (C-2'''), 172.7 (C-15'), 171.0 (C-7'), 166.2 (C-4v), 158.5 (C-4'), 157.0 (C-6av), 152.2 (C-9v), 138.1 (C-4'x), 137.9 (C-1'x), 137.0 (C-5'''), 133.9 (C-1'), 133.5 (C-10av), 133.2 (C-3av), 132.0 (C-2v), 131.3 (C-3v), 131.2 (C-2'x), 130.0 (C-4'''), 129.8 (C-3'), 127.7 (C-3'x), 116.0 (C-2'), 68.3 (C-6'), 55.3 (C-6v), 51.7 (C-3), 40.3 (C-9'), 39.9 (C-13'), 38.9 (C-16'), 34.6 (C-1'v), 34.2 (C-1''), 32.6 (C-5), 30.0 (C-10'), 29.9 (C-12'), 29.3 (C-4), 25.0 (C-11'), 14.4 (C-1v''), 12.9 (C-1v'''), 11.6 (C-1v'); HRMS *m/z* (ESI$^+$) [Found: 869.2959, $C_{42}H_{46}ClN_{10}O_7S$ requires $[M+H]^+$ 869.2955]; LRMS *m/z* (ESI$^+$) 383 (100%), 428 (90%), 869 ($[^{35}M+H]^+$, 34%), 871 ($[^{37}M+H]^+$, 17%); HPLC Retention time 8.9 min, 96.1%.

2-((*S*)-4-(4-Chlorophenyl)-2,3,9-trimethyl-6*H*-thieno[3,2-*f*][1,2,4]triazolo[4,3-*a*][1,4]diazepin-6-yl)-*N*-(5-(2-(4-(1-((5-methoxy-1,2-dimethyl-4,7-dioxo-4,7-dihydro-1*H*-indol-3-yl)methyl)-2,6-dioxopiperidin-3-yl)phenoxy)acetamido)pentyl)acetamide (16, IQ-CRBN)

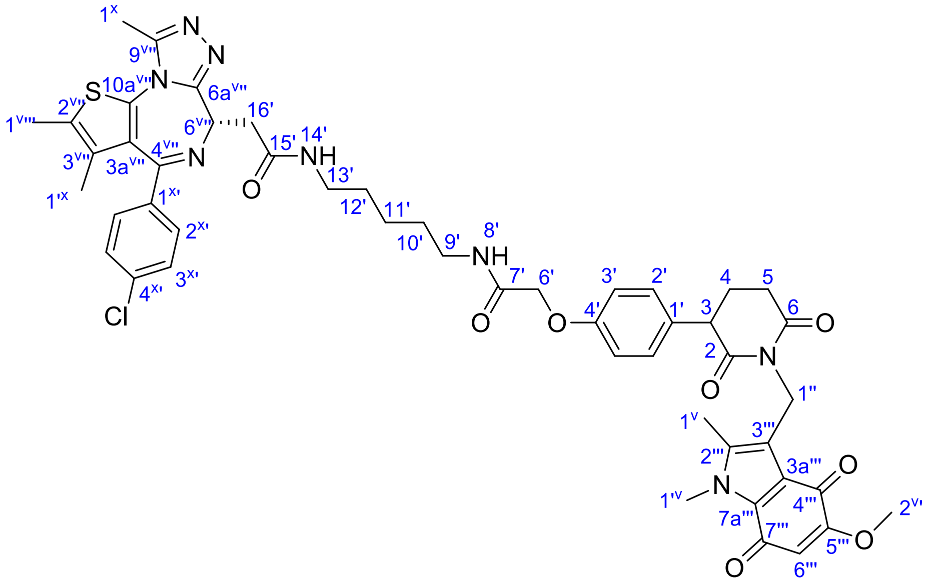

Step 1:

Compound **57** (30 mg, 0.05 mmol, 1.0 eq.) was dissolved in dry CH_3OH (1 mL) and oxalyl chloride (13 µL, 0.15 mmol, 3.0 eq.) was added while maintaining an argon atmosphere. After

3 h at rt, the reaction was incomplete, and so further oxalyl chloride (3.0 eq.) was added and the reaction mixture was stirred at rt for 16 h. After this time, the volatile components were evaporated to afford the crude material as an orange solid, which was used in the next step without further purification.

Step 2:

The obtained intermediate (0.05 mmol, 1.0 eq.) was added to a solution of (+)-JQ1-COOH (20 mg, 0.05 mmol, 1.1 eq.), DIPEA (52 µL, 0.30 mmol, 6.0 eq.) and HATU (38 mg, 0.1 mmol, 2.0 eq.) in dry DMF (1 mL) under argon. The reaction mixture was stirred at rt for 24 h, then diluted with water (5 mL) and extracted with EtOAc (3 × 5 mL). The combined organic layers were dried over sodium sulfate, filtered, and evaporated *in vacuo*. The crude material was purified using silica gel column chromatography, eluting with EtOAc:CH_3OH (0 to 10% CH_3OH) to afford compound **16** as an orange solid (22 mg, 47%). The compound was further purified using semi-preparative HPLC, method B: R_f 0.16 (EtOAc:CH_3OH 85:15); m.p. 113–115 °C (from EtOAc:CH_3OH); $[\alpha]_D^{25}$ = +8.2 (*c* 0.5, CH_3OH); $\tilde{\upsilon}_{max}$ (neat)/cm^{-1} 3311 (N-H, w), 2926 (C-H, s), 2855 (C-H, w), 1731 (C=O, s), 1670 (C=O, s), 1600 (C=O, s), 1538 (N-H, s), 1509 (N-H, w), 1222 (C-O, s), 1176 (C-O, s), 840 (s); ^1H NMR (600 MHz; CD_3OD) δ_H 7.43–7.35 (4H, m, H-2$^{x'}$, H-3$^{x'}$), 7.08 (2H, d, *J* 8.6, H-3'), 6.85 (2H, d, *J* 8.6, H-2'), 5.65 (1H, s, H-6'''), 5.06 (1H, d, *J* 14.8, H-1''^{a}), 4.95 (1H, d, *J* 14.8, H-1''^{b}), 4.61 (1H, dd, *J* 3.8, 1.7, H-6$^{v''}$), 4.46 (2H, s, H-6'), 3.86 (3H, s, H-1'^{v}), 3.84–3.80 (1H, m, H-3), 3.79 (3H, s, H-2$^{v'}$), 3.43–3.36 (1H, m, H-16'^{a}), 3.28–3.18 (5H, m, H-9', H-13', H-16'^{b}), 2.76–2.73 (2H, m, H-5^{a}, H-5^{b}), 2.68 (3H, s, H-1^{x}), 2.43 (3H, s, H-1$^{v'''}$), 2.23 (3H, s, H-1^{v}), 2.12–2.06 (2H, m, H-4^{a}, H-4^{b}), 1.67 (3H, s, H-1'^{x}), 1.58–1.54 (4H, m, H-10', H-12'), 1.38–1.33 (2H, m, H-11'); ^{13}C NMR (151 MHz; CD_3OD) δ_C 180.1 (C-4'''), 179.0 (C-7'''), 175.7 (C-2), 174.5 (C-6), 172.7 (C-15'), 171.1 (C-7'), 166.2 (C-4$^{v''}$), 161.1 (C-5'''), 158.3 (C-4'), 157.0 (C-6a$^{v''}$), 152.2 (C-9$^{v''}$), 139.0 (C-3'''), 138.1 (C-4$^{x'}$), 137.9 (C-1$^{x'}$), 133.5 (2C, C-3a$^{v''}$, C-10a$^{v''}$), 133.2 (C-1'), 132.0 (2C, C-2$^{v''}$, C-3$^{v''}$), 131.3 (C-2$^{x'}$), 130.7 (C-3'), 129.8 (C-7a'''), 129.8 (C-3$^{x'}$), 122.6 (C-3a'''), 118.9 (C-2), 115.8 (C-2'), 107.4 (C-6'''), 68.3 (C-6'), 57.1 (C-2$^{v'}$), 55.3 (C-6$^{v''}$), 49.6 (C-3), 40.3 (C-9'), 39.8 (C-13'), 38.9 (C-16'), 35.8 (C-1''), 32.9 (C-5), 32.7 (C-1'^{v}), 29.9 (C-12'), 29.8 (C-10'), 26.6 (C-4), 24.9 (C-11'), 14.4 (C-1'^{x}), 12.9 (C-1^{v}), 11.6 (C-1^{x}), 9.6 (C-1$^{v'''}$); HRMS *m/z* (ESI^+) [Found: 947.3326, $C_{49}H_{52}ClN_8O_8S$ requires $[M+H]^+$ 947.3312]; LRMS *m/z* (ESI^+) 304 (100%), 428 (93%), 947 ($[^{35}M+H]^+$, 88%), 949 ($[^{37}M+H]^+$, 43%), 969 ($[^{35}M+Na]^+$, 49%), 971 ($[^{37}M+H]^+$, 15%); HPLC Retention time 9.2 min, 96.8%.

2-(4-(1-Benzyl-2,6-dioxopiperidin-3-yl)phenoxy)-*N*-(5-(2-((S)-4-(4-chlorophenyl)-2,3,9-trimethyl-6*H*-thieno[3,2-*f*][1,2,4]triazolo[4,3-*a*][1,4]diazepin-6-yl)acetamido)pentyl)acetamide (18, Bn-CRBN)

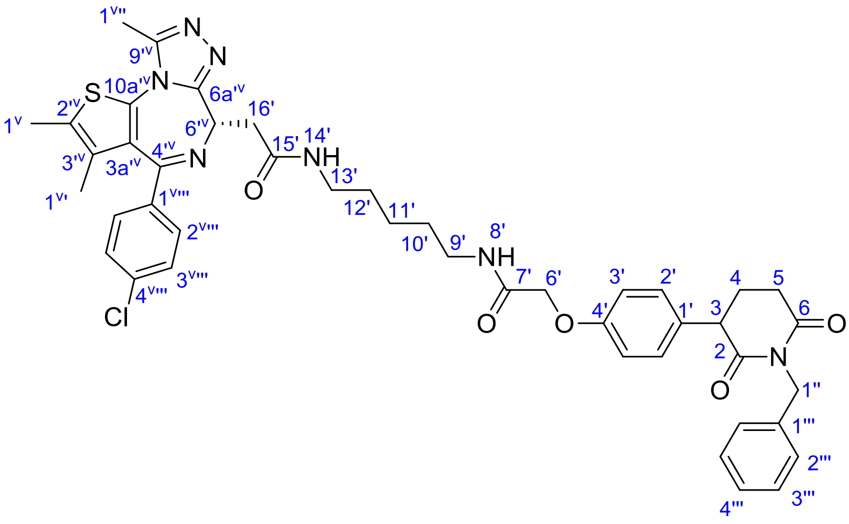

Step 1:

Compound **58** (37 mg, 0.07 mmol, 1.0 eq.) was dissolved in dry CH_3OH (1 mL) and oxalyl chloride (18 µL, 0.21 mmol, 3.0 eq.) was added while maintaining an argon atmosphere. After 3 h at rt, the reaction was incomplete and so further oxalyl chloride (3.0 eq.) was added and the reaction mixture was stirred at rt for 16 h. After this time, the volatile component were evaporated to afford the crude material as a yellow oil, which was used in the next step without further purification.

Step 2:

The obtained intermediate (0.07 mmol, 1.0 eq.) was added to a solution of (+)-JQ1-COOH (31 mg, 0.08 mmol, 1.1 eq.), DIPEA (73 µL, 0.42 mmol, 6.0 eq.) and HATU (53 mg, 0.14 mmol, 2.0 eq.) in dry DMF (1 mL) under argon. The reaction mixture was stirred at rt for 16 h, then diluted with water (5 mL) and extracted with EtOAc (2 × 5 mL). The combined organic layers were washed with 0.5 M aqueous LiCl solution (1 × 10 mL), dried over sodium sulfate, filtered, and evaporated *in vacuo*. The crude material was purified using silica gel column chromatography, eluting with EtOAc:CH_3OH (gradient 0 to 5% CH_3OH) to afford the title compound as a colourless solid (38 mg, 66%). The compound was further purified using semi-preparative HPLC, method A: R_f 0.19 (EtOAc:CH_3OH 85:15); m.p. 68–70 °C (from EtOAc:CH_3OH); $[\alpha]_D^{25}$ = +12.7 (*c* 1.0, CH_3OH); $\tilde{\upsilon}_{max}$ (neat)/cm^{-1} 3309 (N-H, w), 2927 (C-H, w), 2860 (C-H, w), 1725 (C=O, s), 1673 (C=O, s), 1534 (N-H, s), 1511 (N-H, w), 1420 (w), 1226 (w), 1164 (C-O, s), 1013 (w), 839 (C-H, w), 730 (C-H, s); ^{1}H NMR (600 MHz; CD_3OD)

δ_H 7.43–7.37 (4H, m, H-2$^{v'''}$, H-3$^{v'''}$), 7.30–7.20 (5H, m, H-2''', H-3''', H-4'''), 7.06 (2H, d, *J* 8.7, H-3'), 6.87 (2H, d, *J* 8.7, H-2'), 4.95 (2H, s, H-1''), 4.61 (1H, dd, *J* 3.6, 2.6, H-6$^{'v}$), 4.46 (2H, s, H-6'), 3.88 (1H, dd, *J* 10.6, 5.3, H-3), 3.40 (1H, dd, *J* 8.9, 2.6 H-16$^{'a}$), 3.29–3.19 (5H, m, H-9', H-13', H-16$^{'b}$), 2.82–2.67 (5H, m, H-5^{a}, H-5^{b}, H-1$^{v''}$), 2.43 (3H, s, H-1^{v}), 2.21–2.11 (2H, m, H-4^{a}, H-4^{b}), 1.68 (3H, s, H-1$^{v'}$), 1.62–1.53 (4H, m, H-10', H-12'), 1.40–1.35 (2H, m, H-11'); ^{13}C NMR (151 MHz; CD_3OD) δ_C 175.8 (C-2), 174.5 (C-6), 172.7 (C-15'), 171.1 (C-7'), 166.2 (C-4$^{'v}$), 158.3 (C-4'), 157.0 (C-6a$^{'v}$), 152.2 (C-9$^{'v}$), 138.9 (C-1'''), 138.1 (C-4$^{v'''}$), 137.9 (C-1$^{v'''}$), 133.5 (C-10a$^{'v}$), 133.4 (C-3a$^{'v}$), 133.2 (C-1'), 132.0 (C-3$^{'v}$), 132.0 (C-2$^{'v}$), 131.3 (C-2$^{v'''}$), 130.6 (C-3'), 129.8 (C-3$^{v'''}$), 129.4 (C-2'''), 129.3 (C-3'''), 128.3 (C-4'''), 115.9 (C-2'), 68.3 (C-6'), 55.3 (C-6$^{'v}$), 49.6 (C-3), 44.0 (C-1''), 40.3 (C-13'), 39.8 (C-9'), 38.9 (C-16'), 32.8 (C-5), 29.9 (C-12') ,29.8 (C-10'), 26.5 (C-4), 25.0 (C-11'), 14.4 (C-1$^{v''}$), 12.9 (C-1^{v}), 11.6 (C-1$^{v'}$); HRMS *m/z* (ESI$^+$) [Found: 820.3067, $C_{44}H_{47}ClN_7O_5S$ requires [M+H]$^+$ 820.3042]; LRMS *m/z* (ESI$^+$) 820 ([^{35}M+H]$^+$, 100%), 822 ([^{37}M+H]$^+$, 71%), 842 ([^{35}M+Na]$^+$, 83%), 844 ([^{37}M+Na]$^+$, 53%); HPLC Retention time 9.9 min, 99.3%.

4. NMR spectra

^{1}H NMR (500 MHz; $CDCl_3$): compound **33**

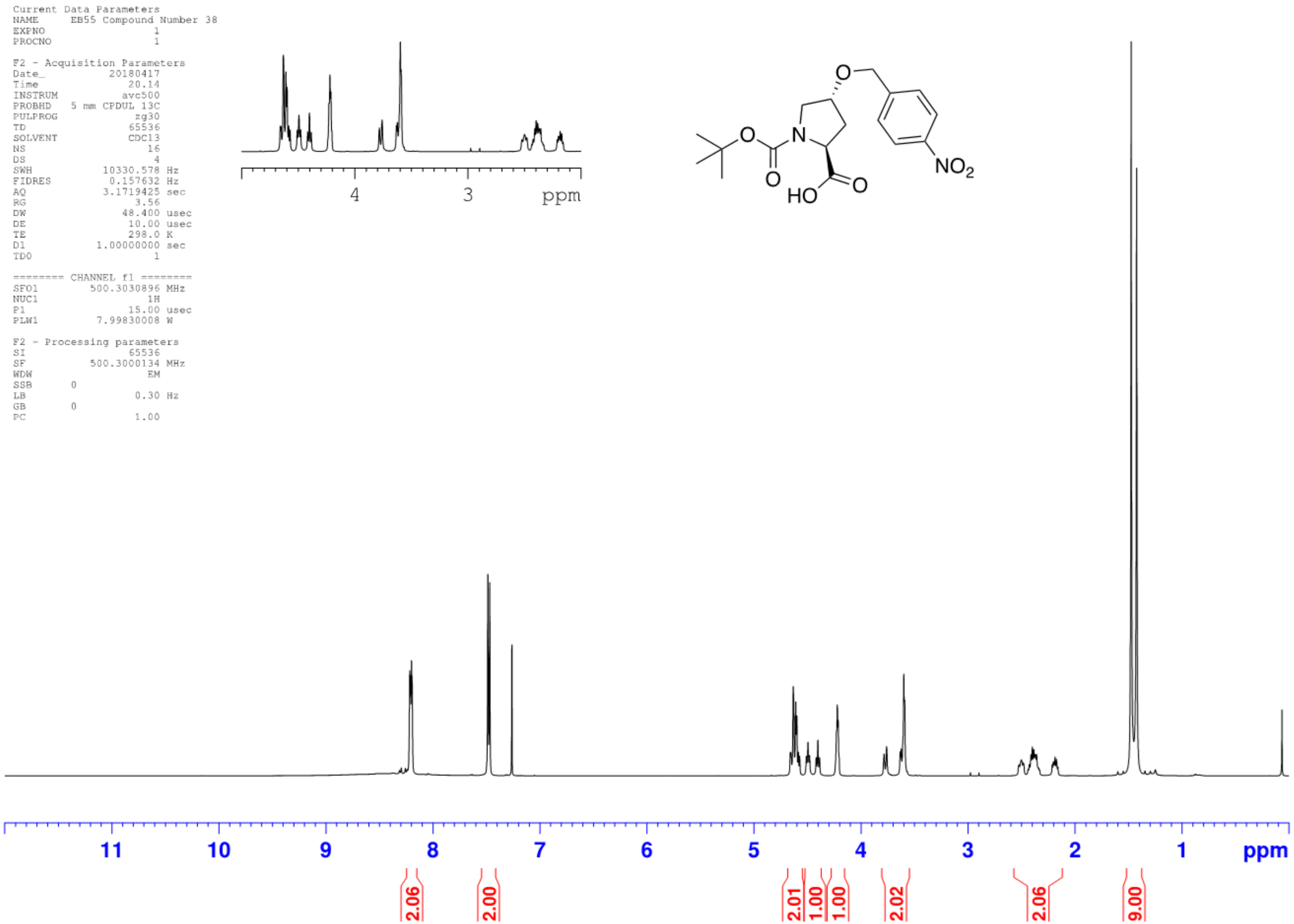

^{13}C NMR (125 MHz; $CDCl_3$): compound **33**

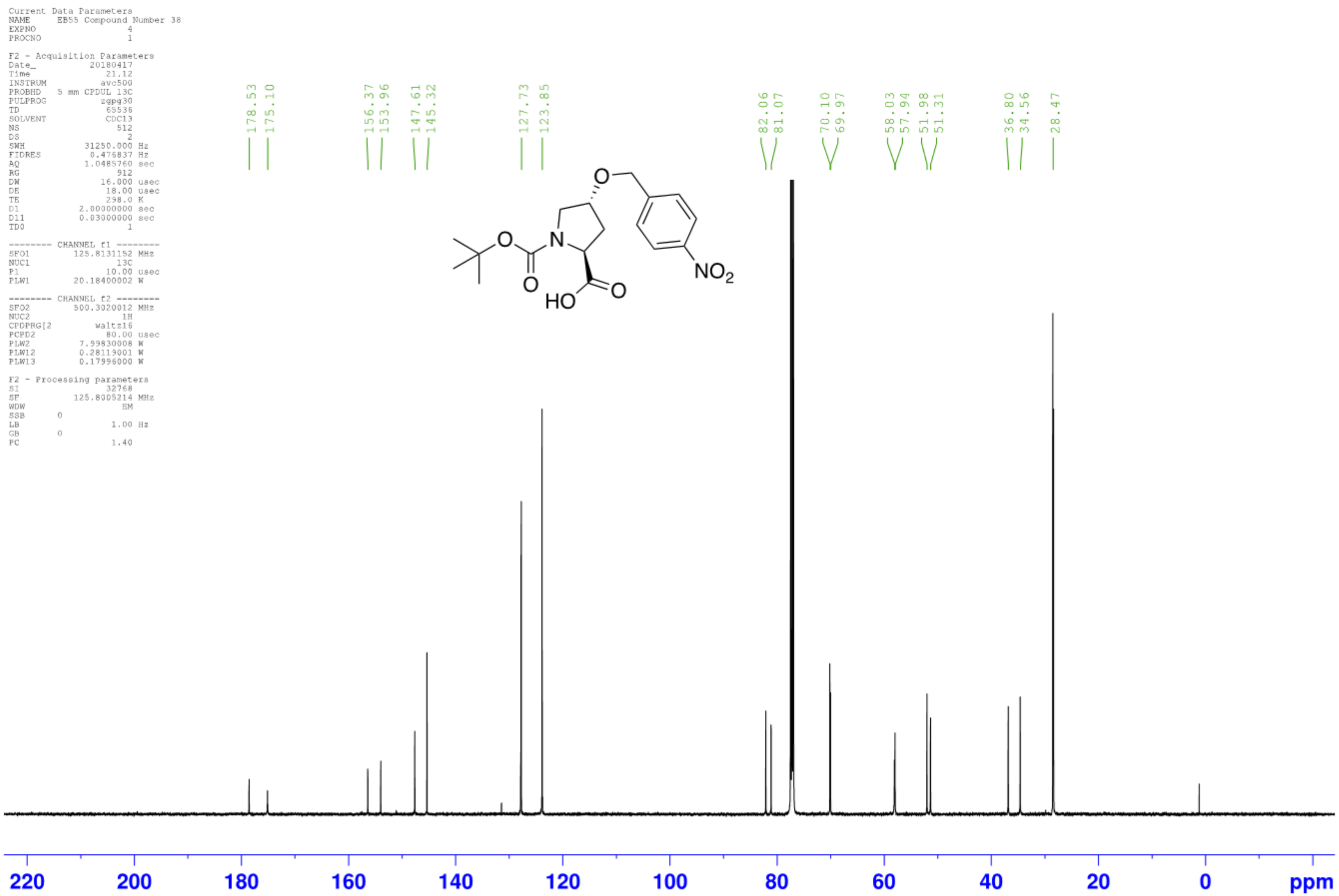

^{1}H NMR (500 MHz; $CDCl_3$): compound **37**

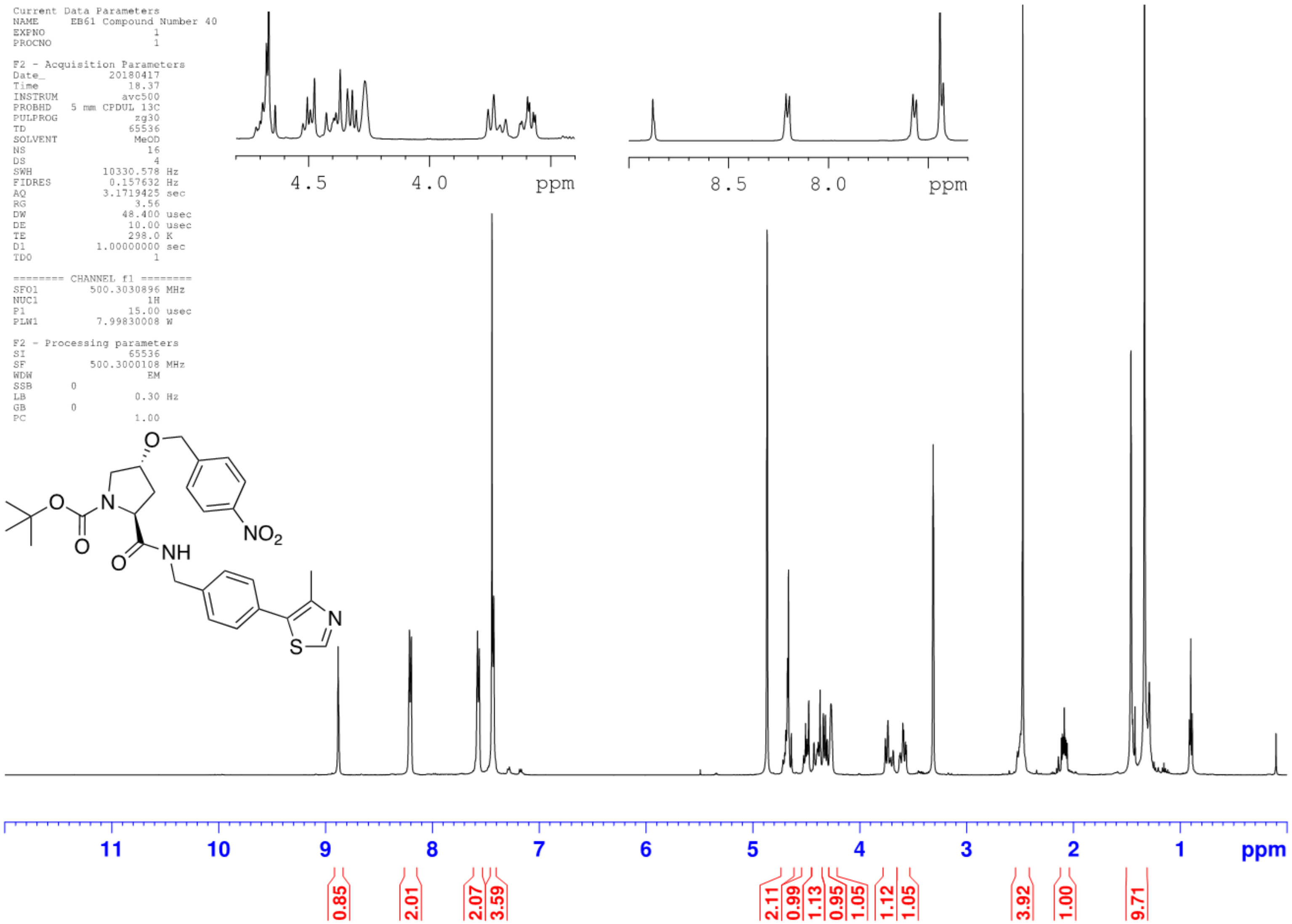

^{13}C NMR (125 MHz; $CDCl_3$): compound **37**

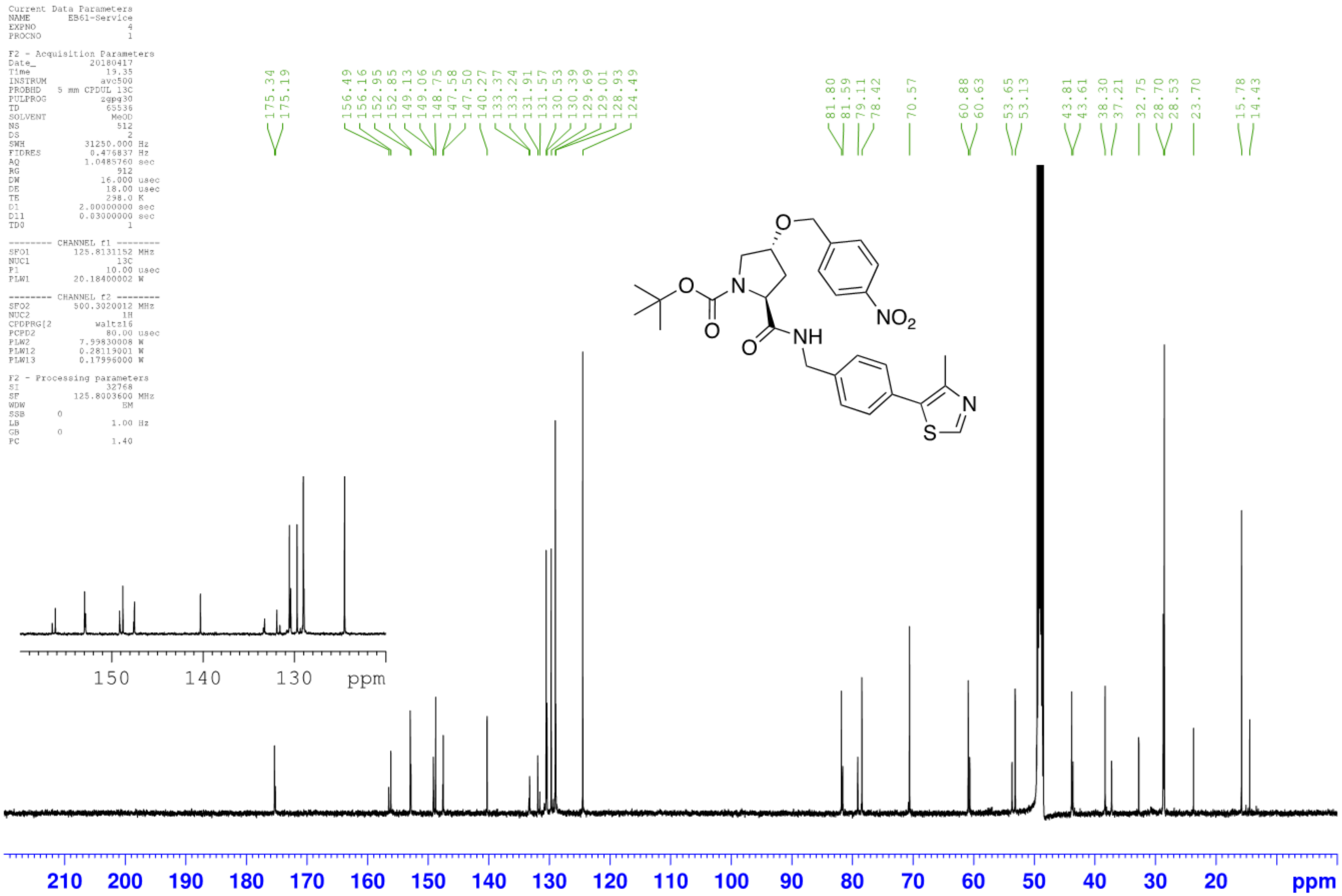

^{1}H NMR (500 MHz; $CDCl_3$): compound **1**

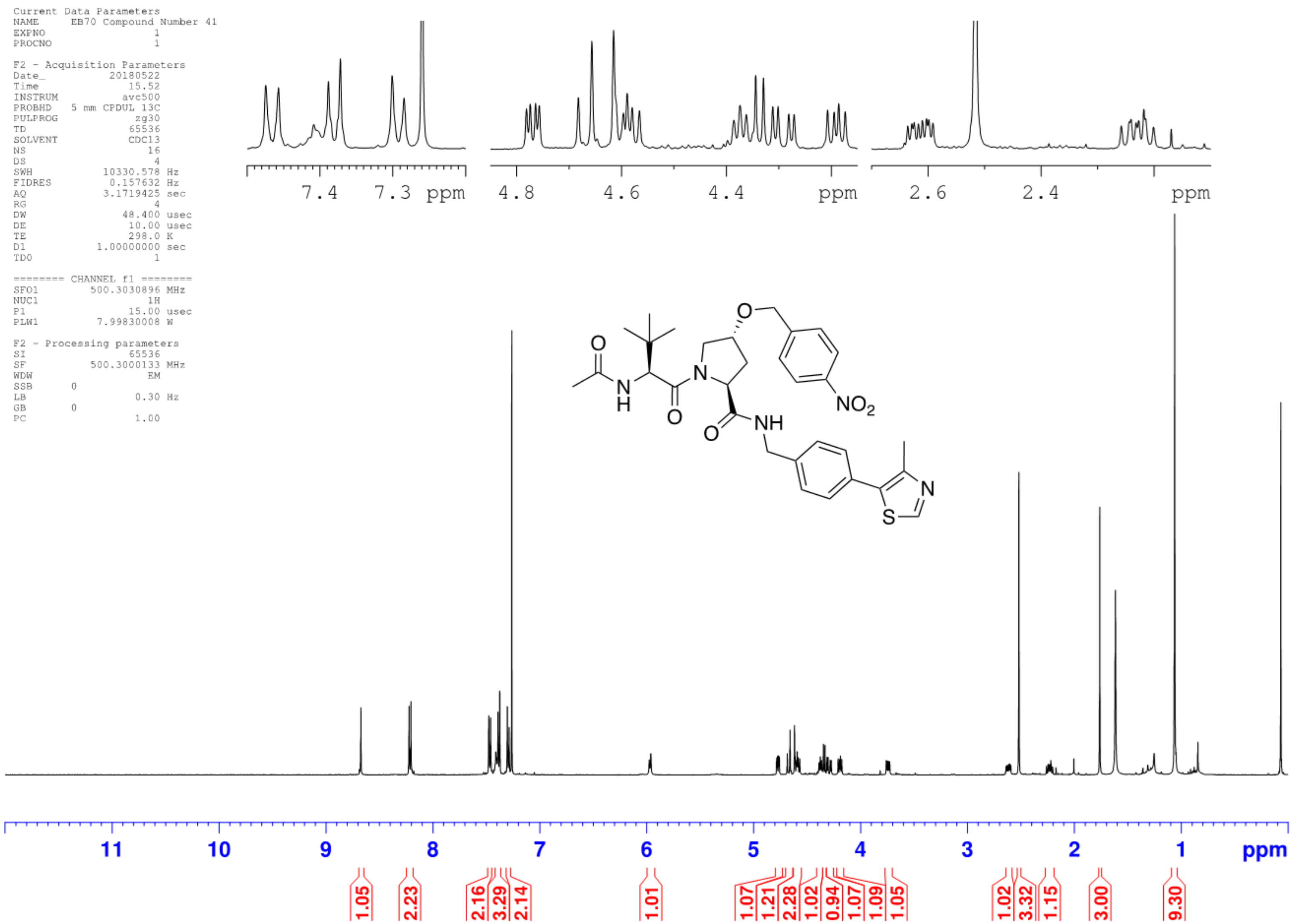

^{13}C NMR (125 MHz; $CDCl_3$): compound **1**

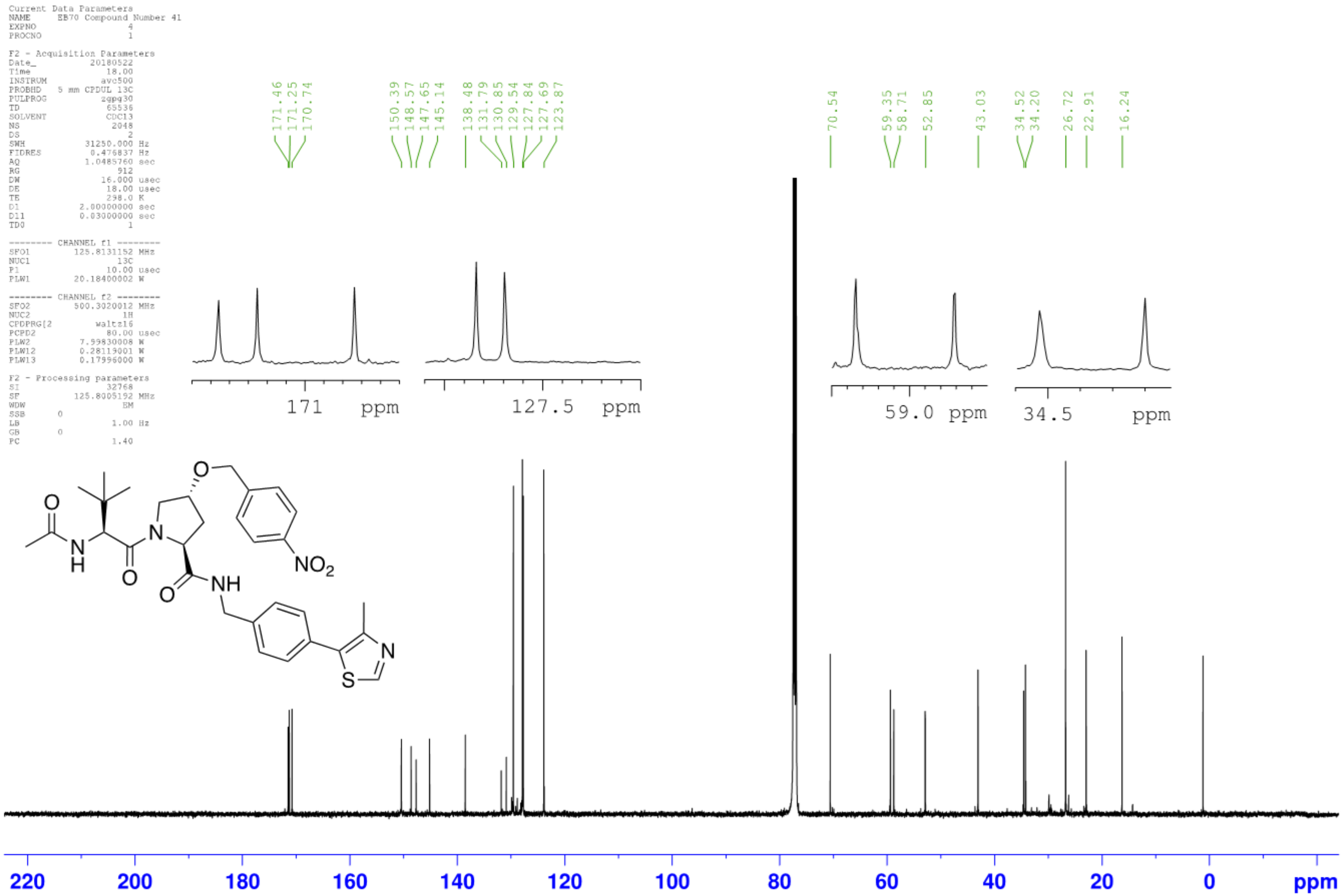

^{1}H NMR (600 MHz; $CDCl_3$): compound **4**

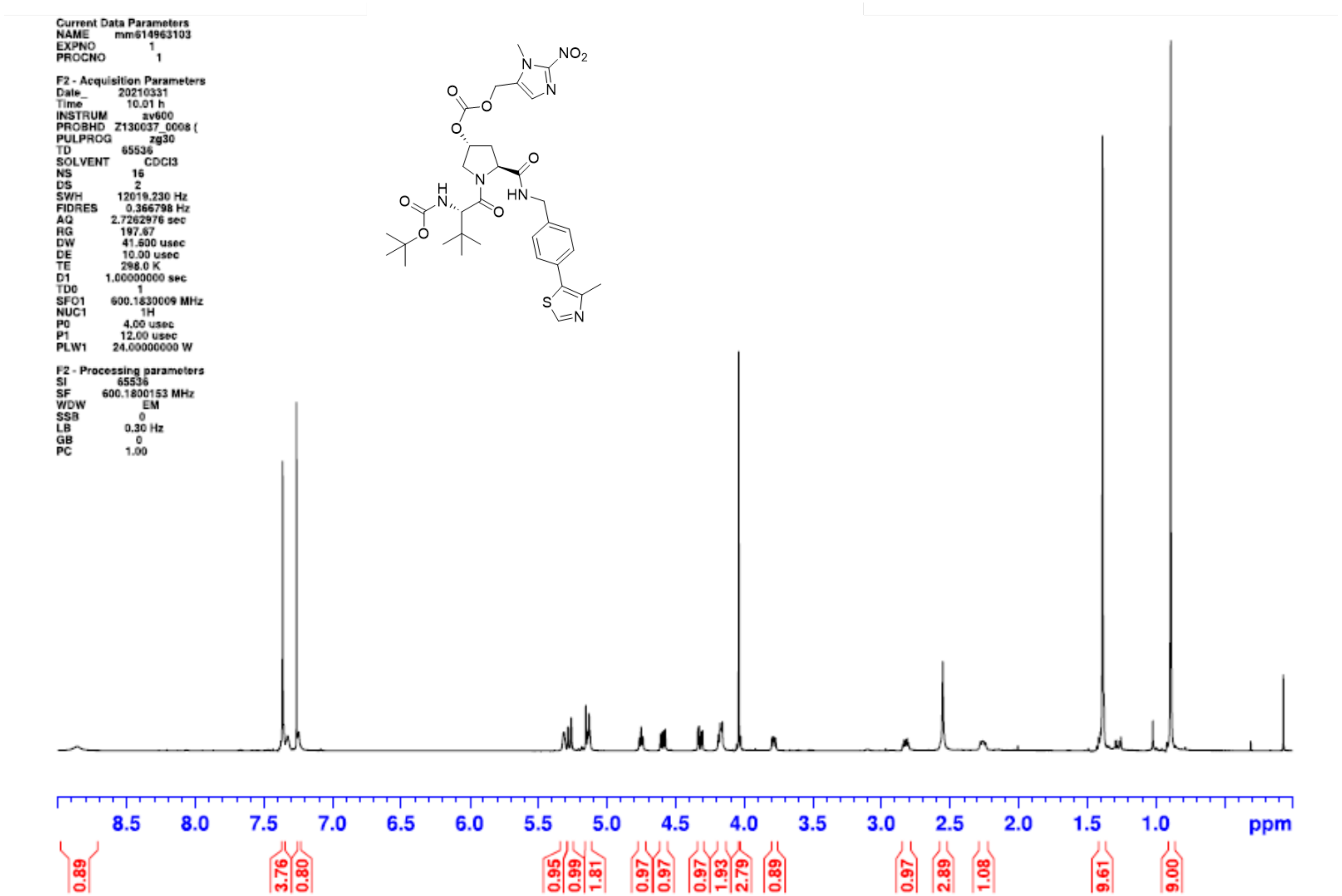

^{13}C NMR (151 MHz; $CDCl_3$): compound **4**

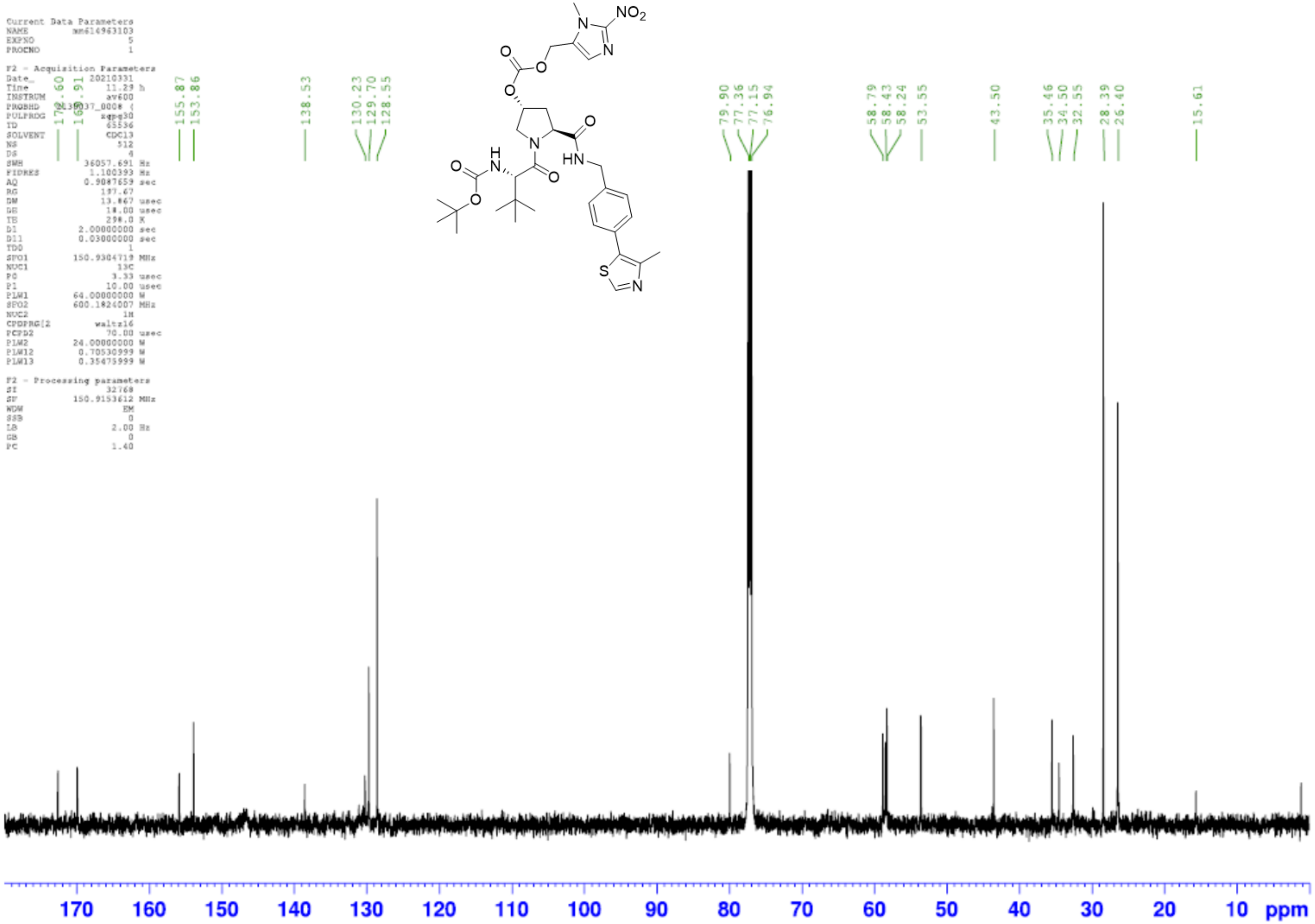

^{1}H NMR (600 MHz; CD_3OD): compound **6** (NI-VHL)

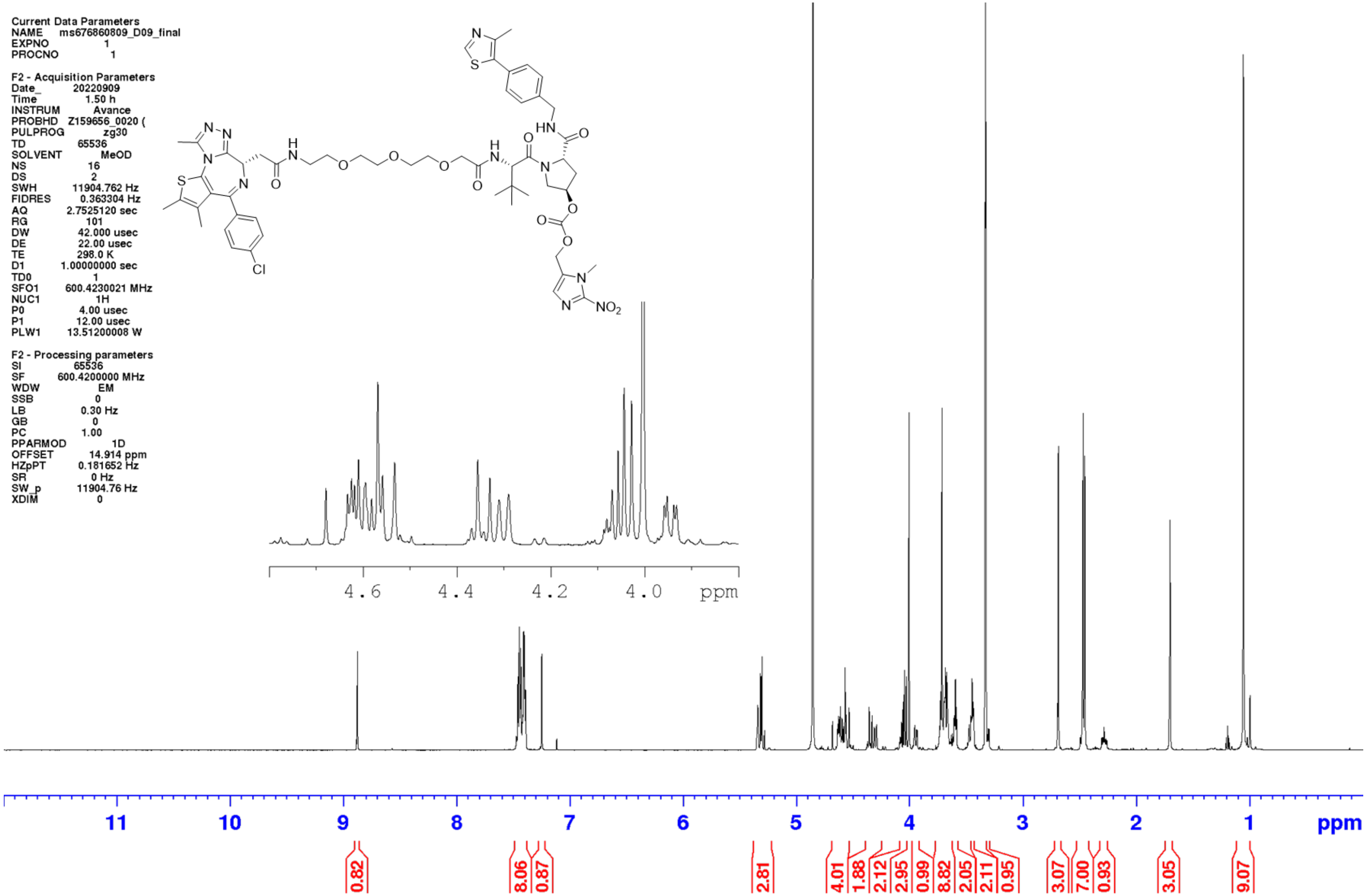

^{13}C NMR (151 MHz; CD_3OD): compound **6** (NI-VHL)

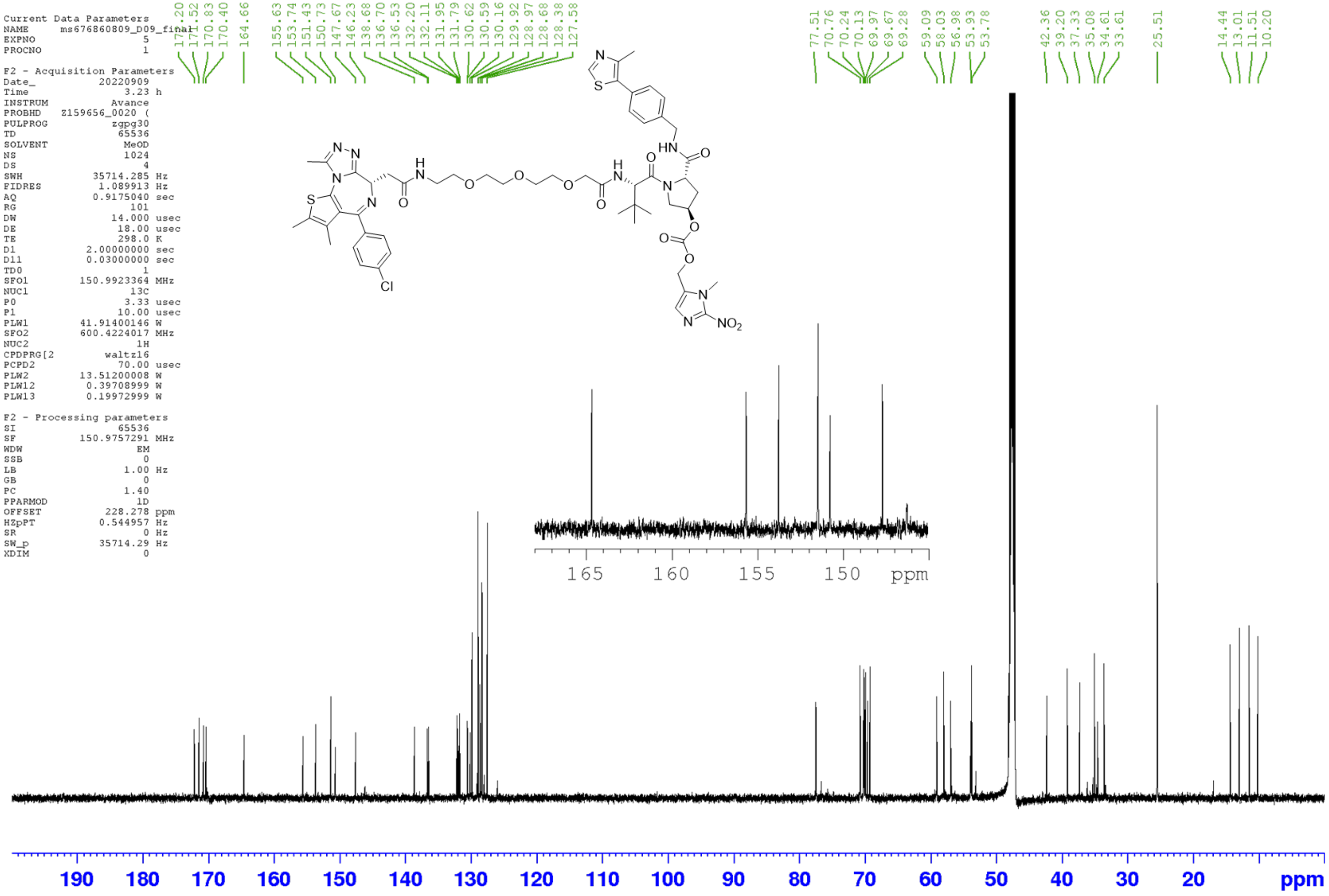

^{1}H NMR (600 MHz; $CDCl_3$): compound **43**

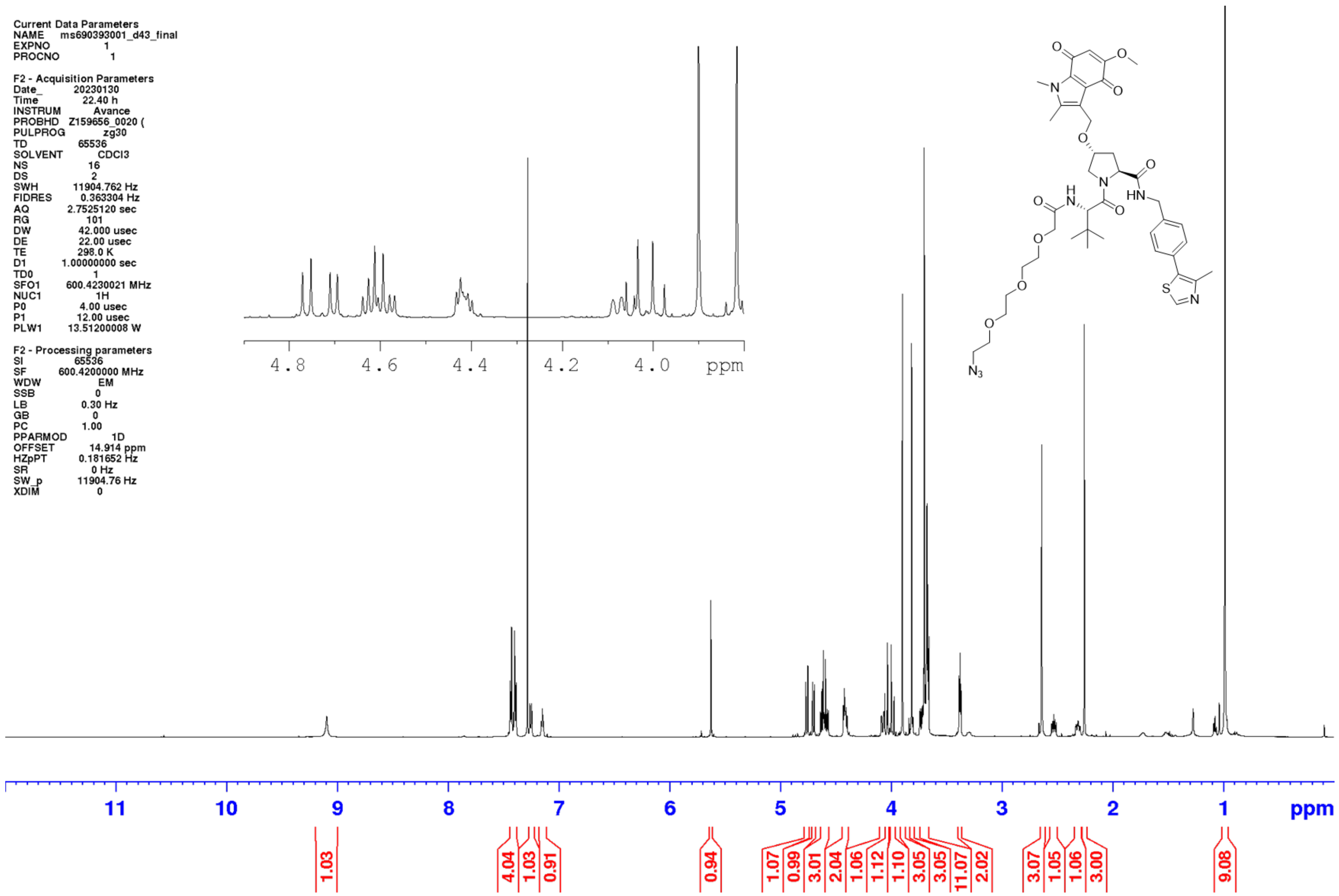

^{13}C NMR (151 MHz; $CDCl_3$): compound **43**

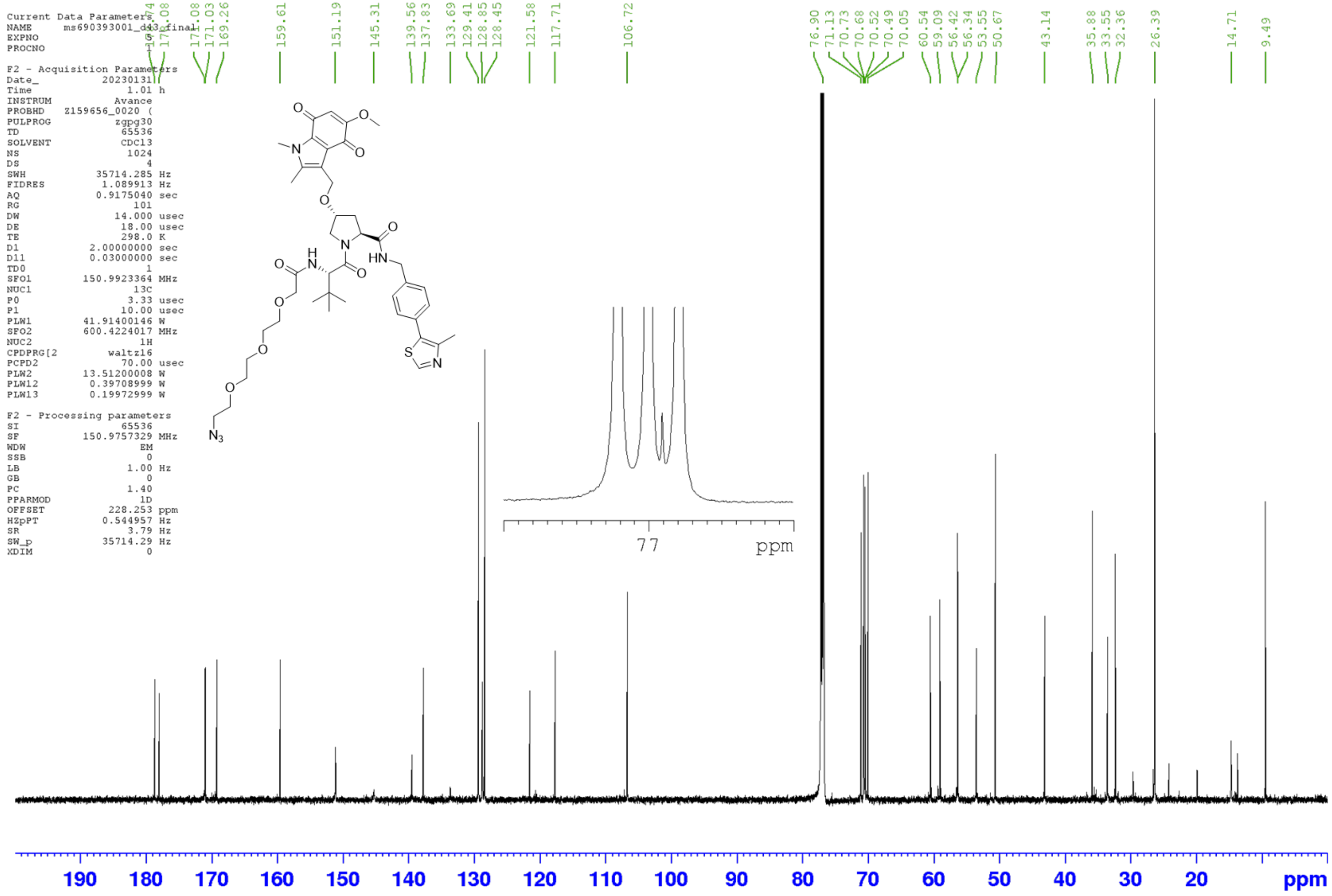

^{1}H NMR (400 MHz; $CDCl_3$): compound **44**

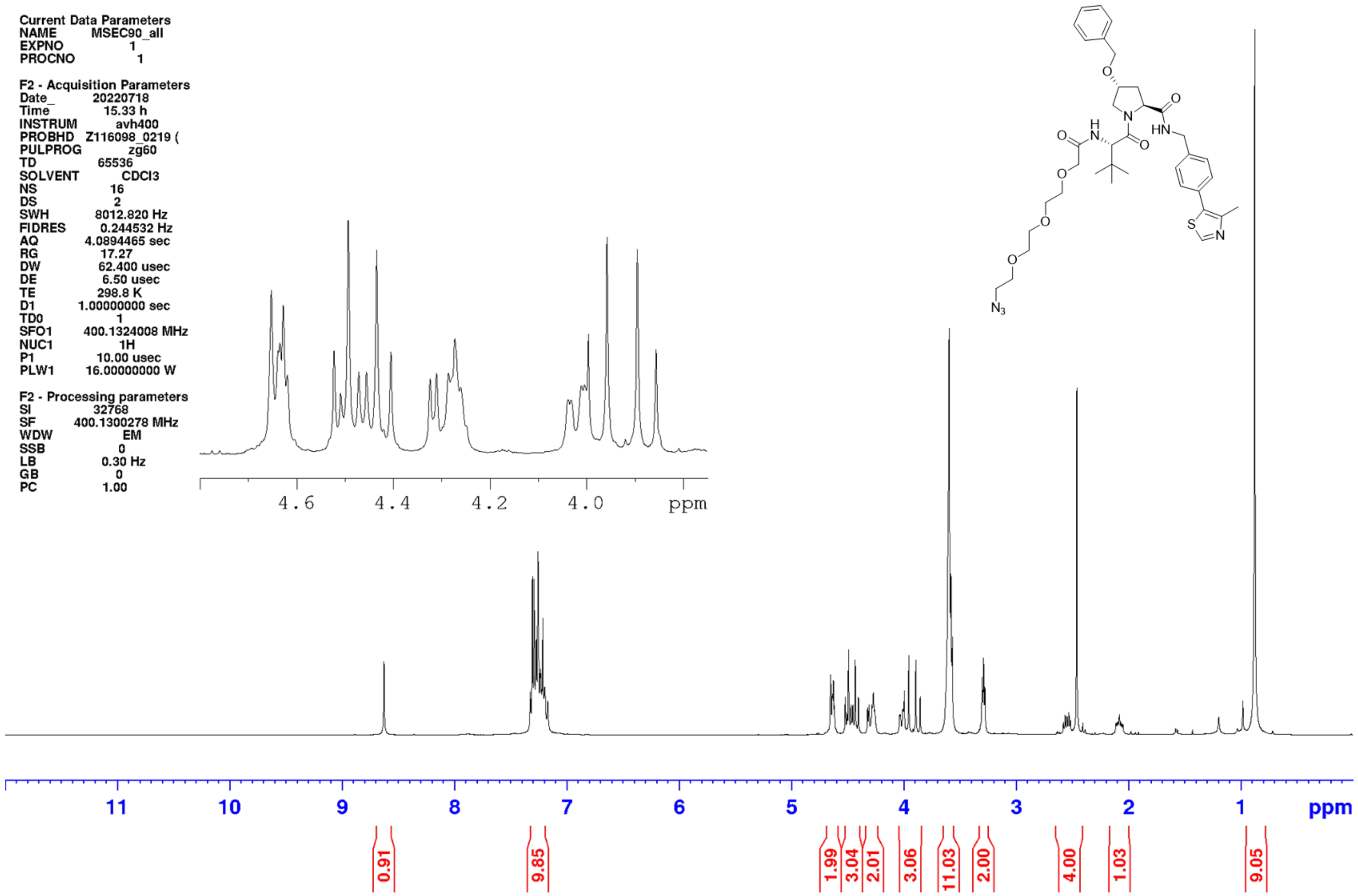

^{13}C NMR (101 MHz; $CDCl_3$): compound **44**

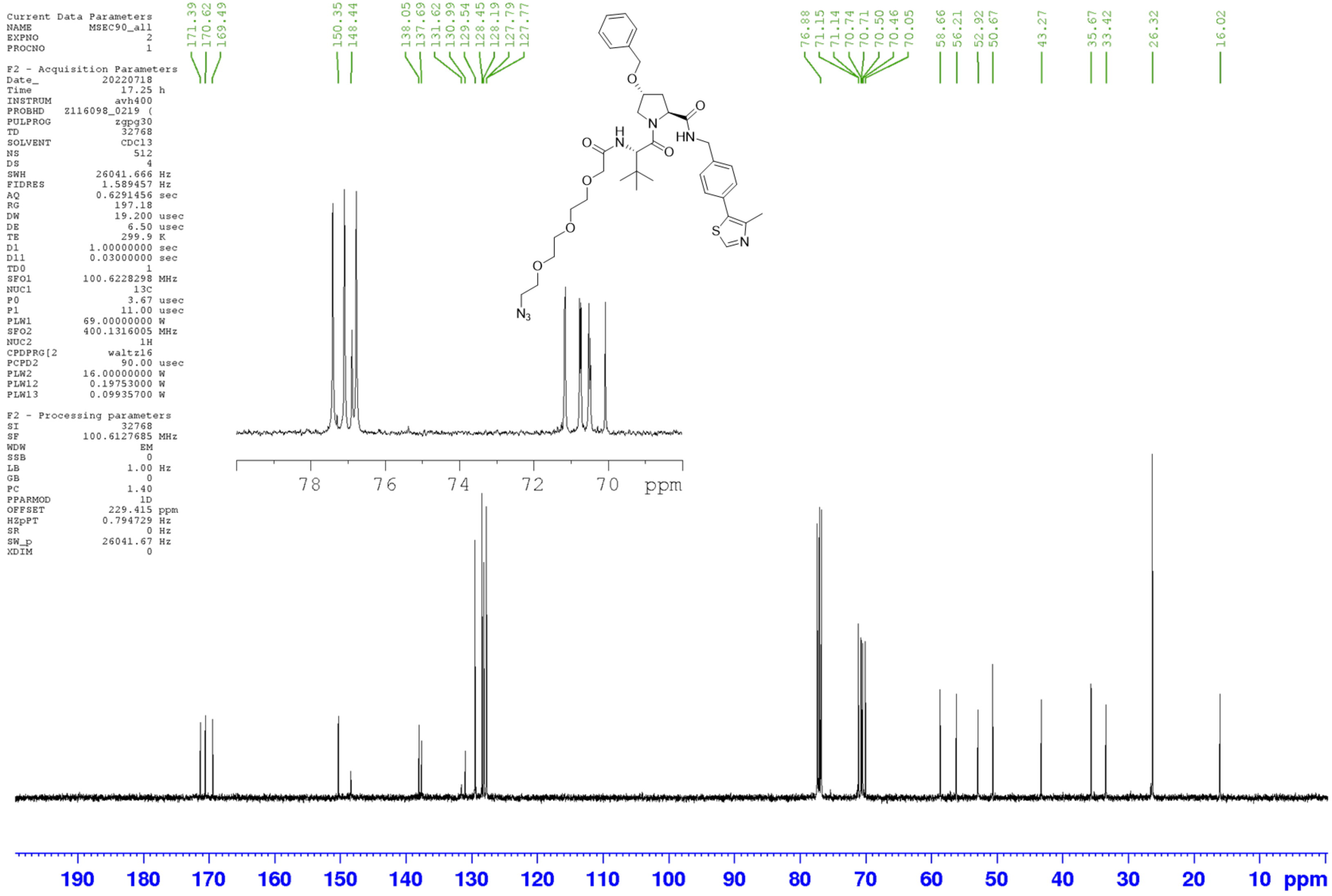

^{1}H NMR (600 MHz; CD_3OD): compound **7** (IQ-VHL)

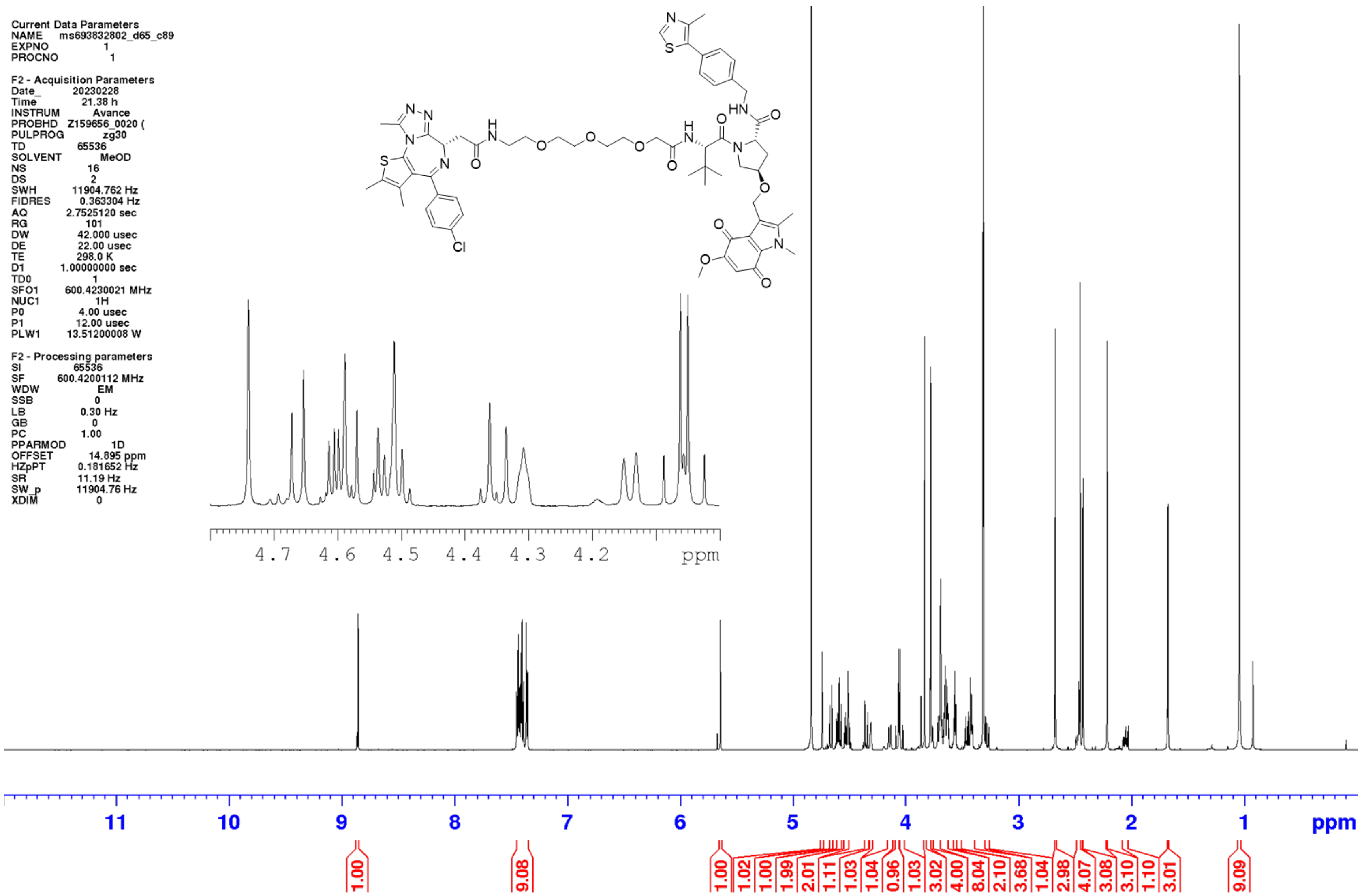

^{13}C NMR (151 MHz; CD_3OD): compound **7** (IQ-VHL)

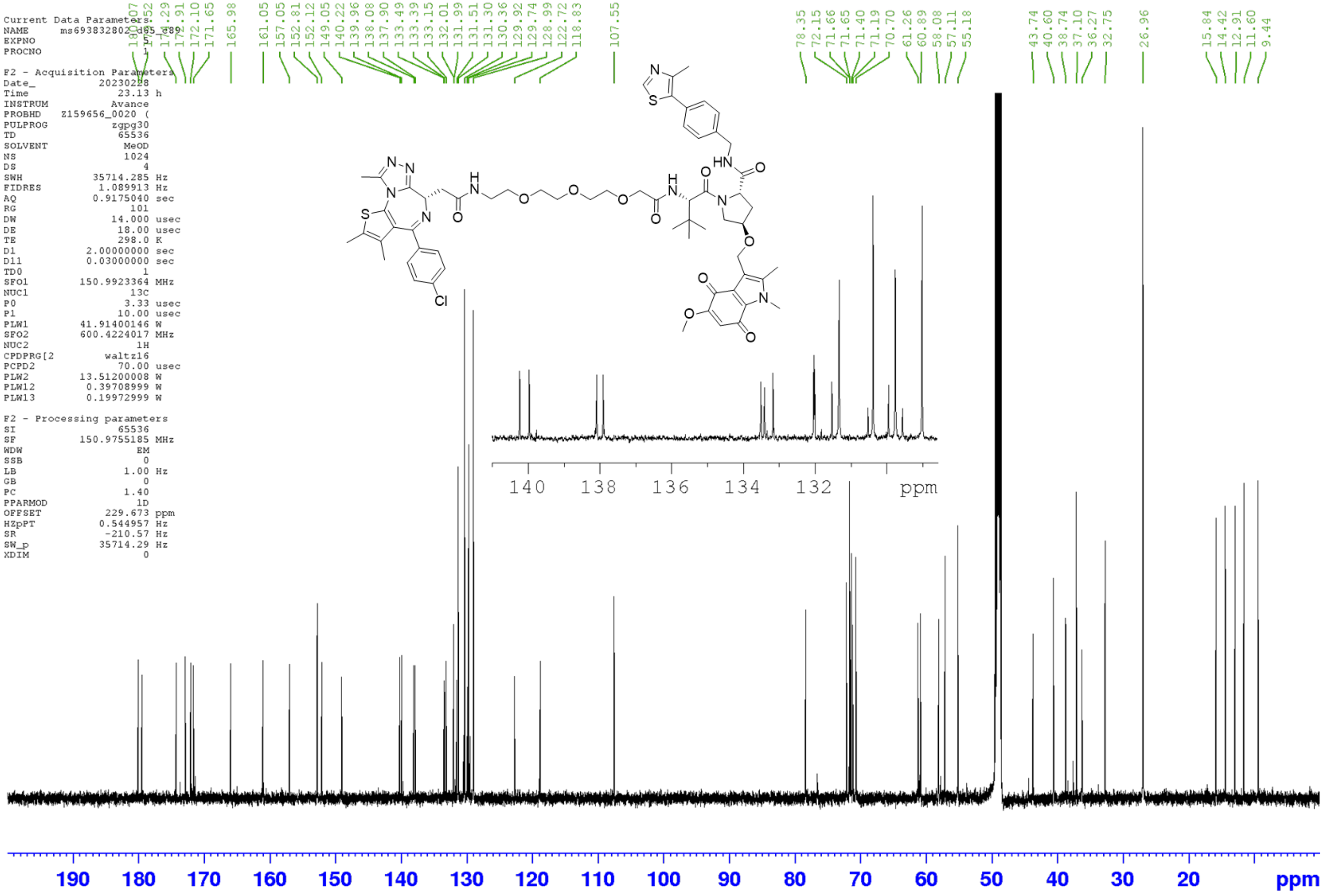

^{1}H NMR (600 MHz; CD_3OD): compound **9** (Bn-VHL)

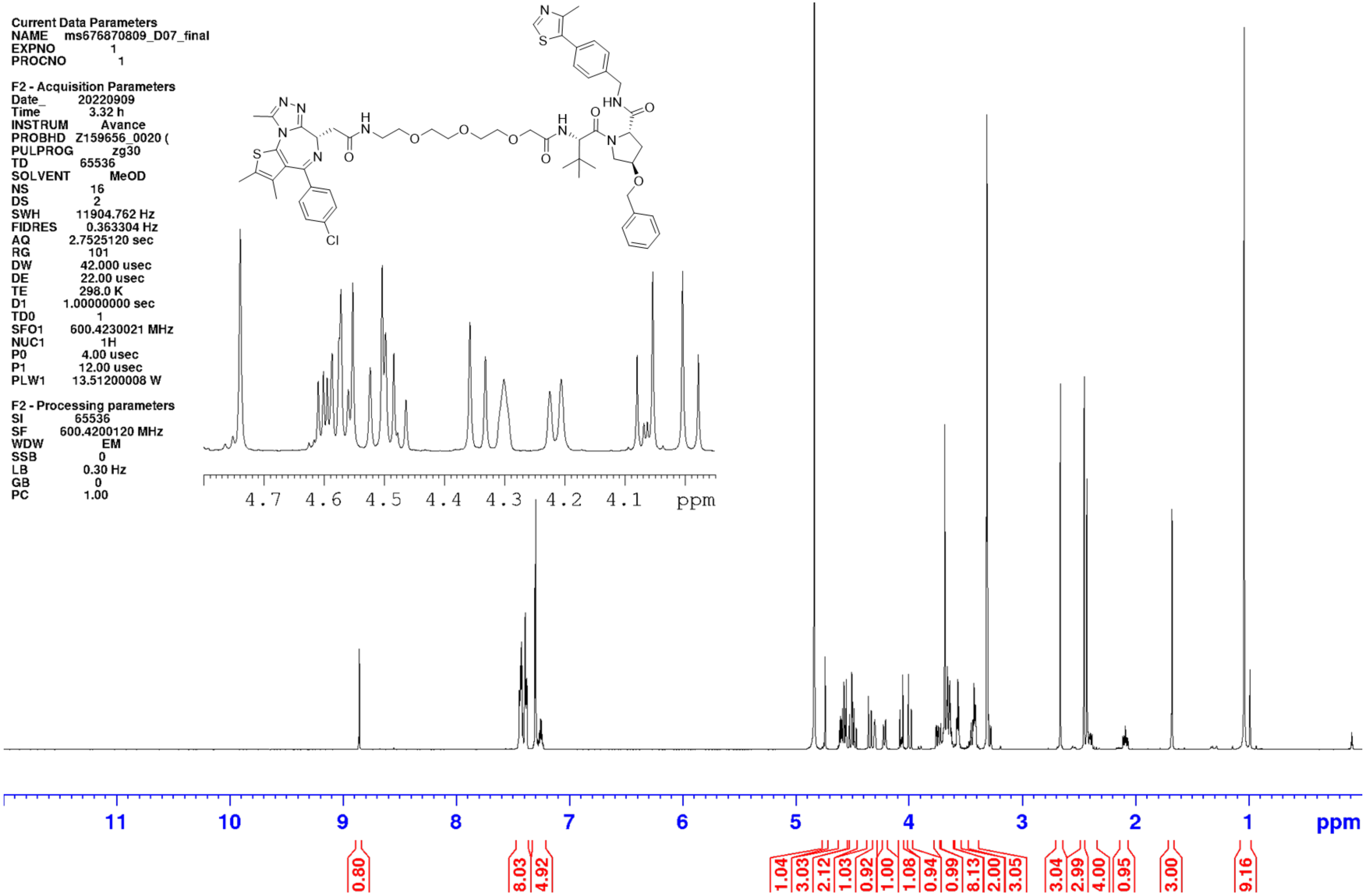

^{13}C NMR (151 MHz; CD_3OD): compound **9** (Bn-VHL)

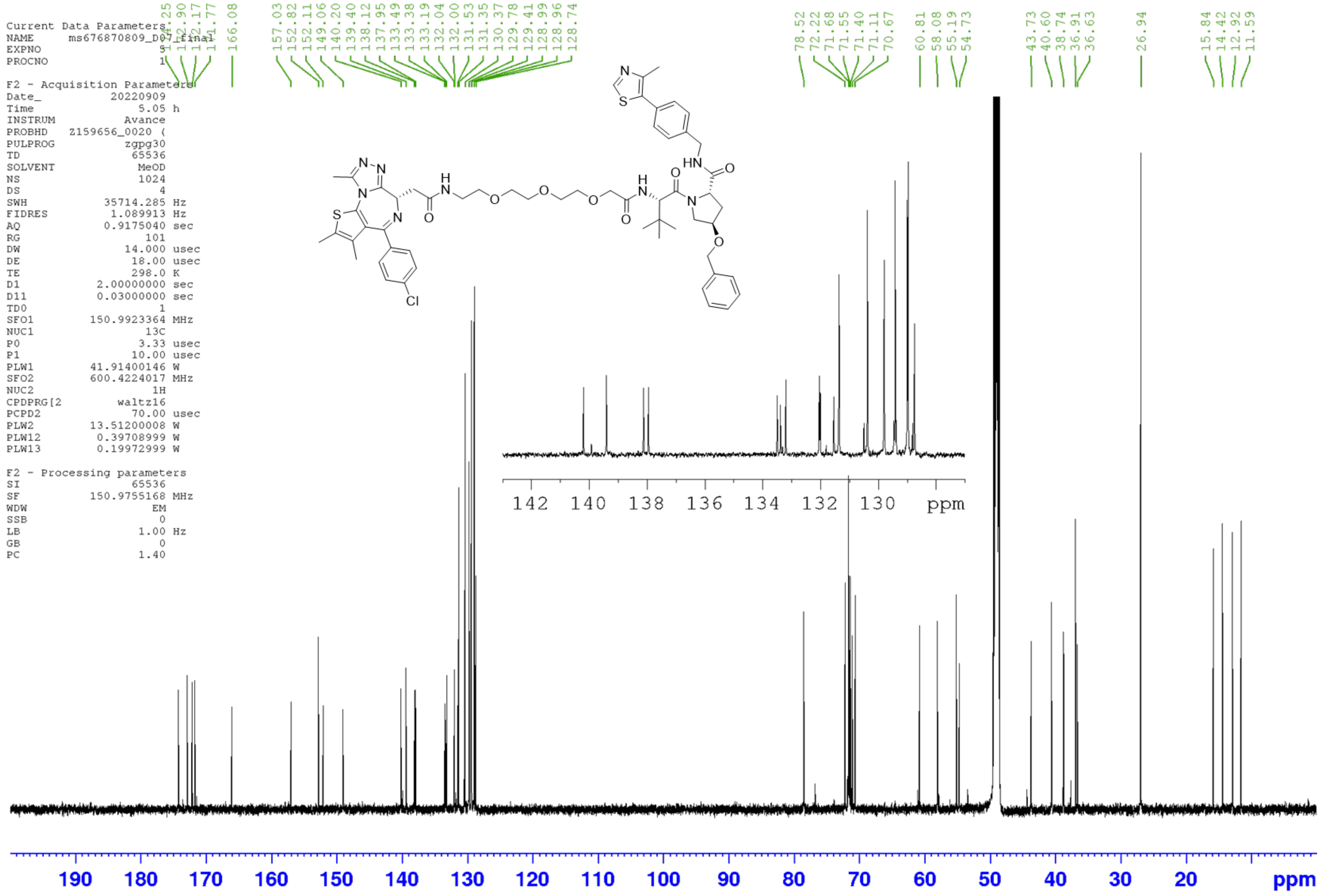

^{1}H NMR (600 MHz; D_6-DMSO): compound **10** (NB-POMA)

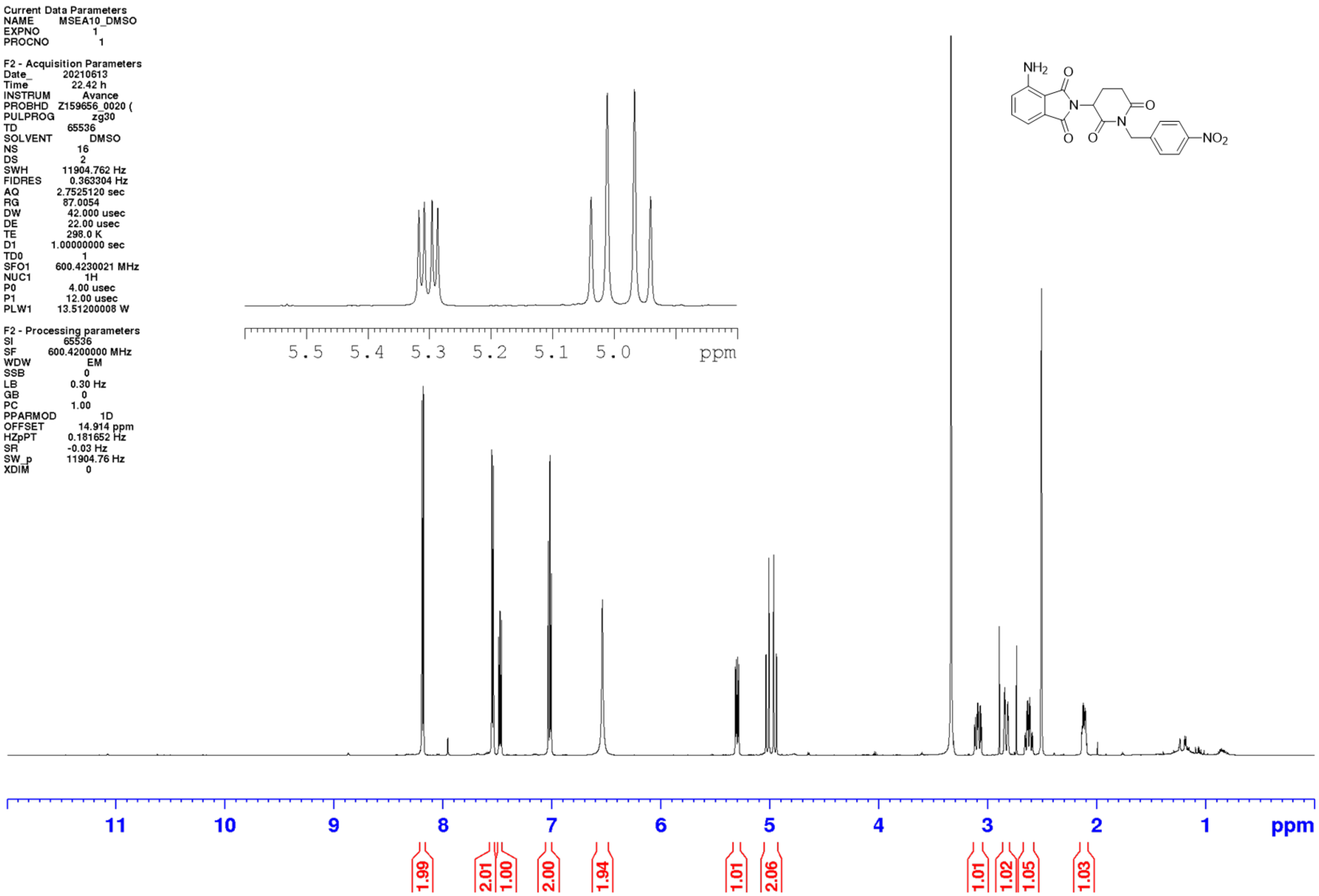

^{13}C NMR (151 MHz; D_6-DMSO): compound **10** (NB-POMA)

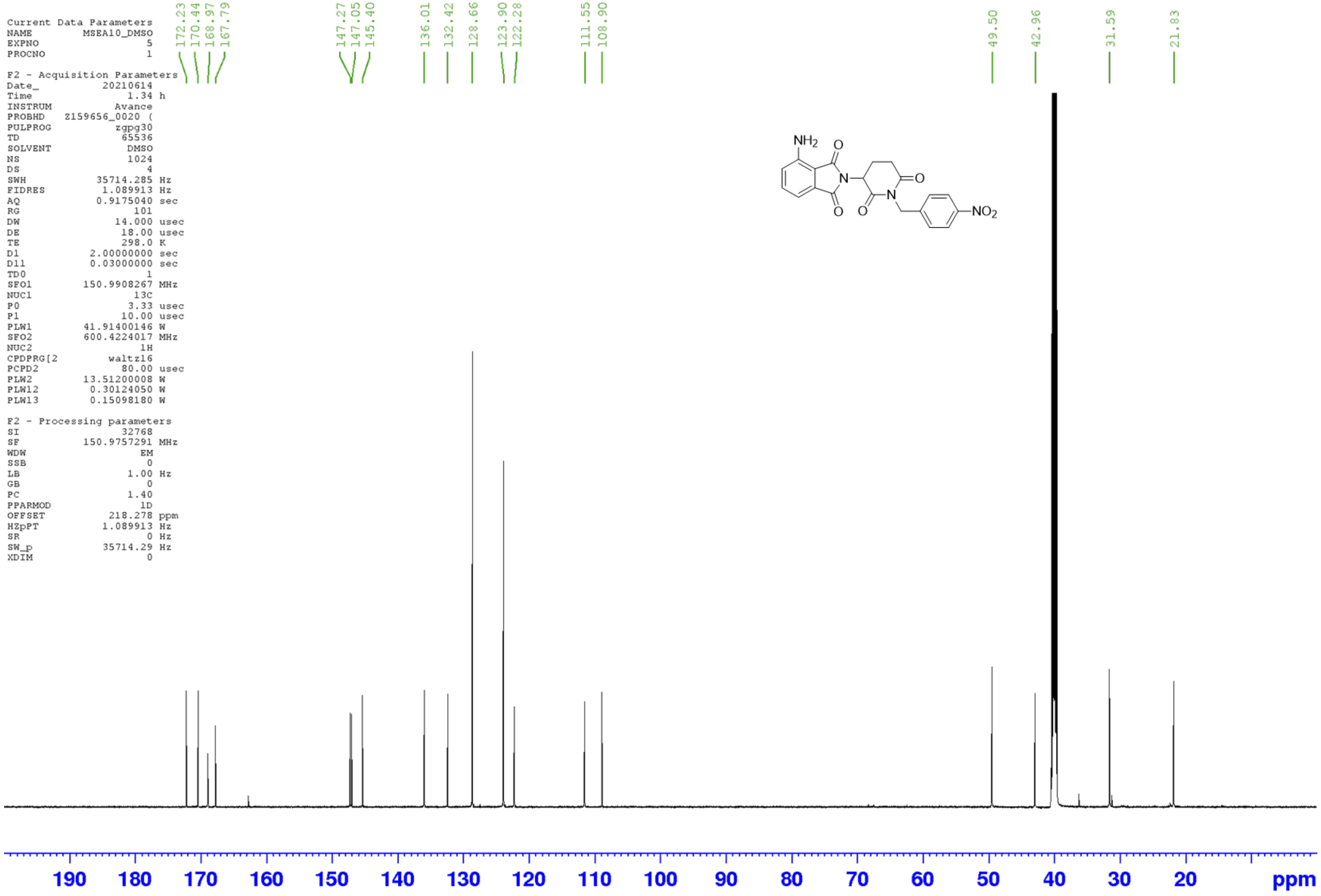

^{1}H NMR (600 MHz; D_6-DMSO): compound **12** (NI-POMA)

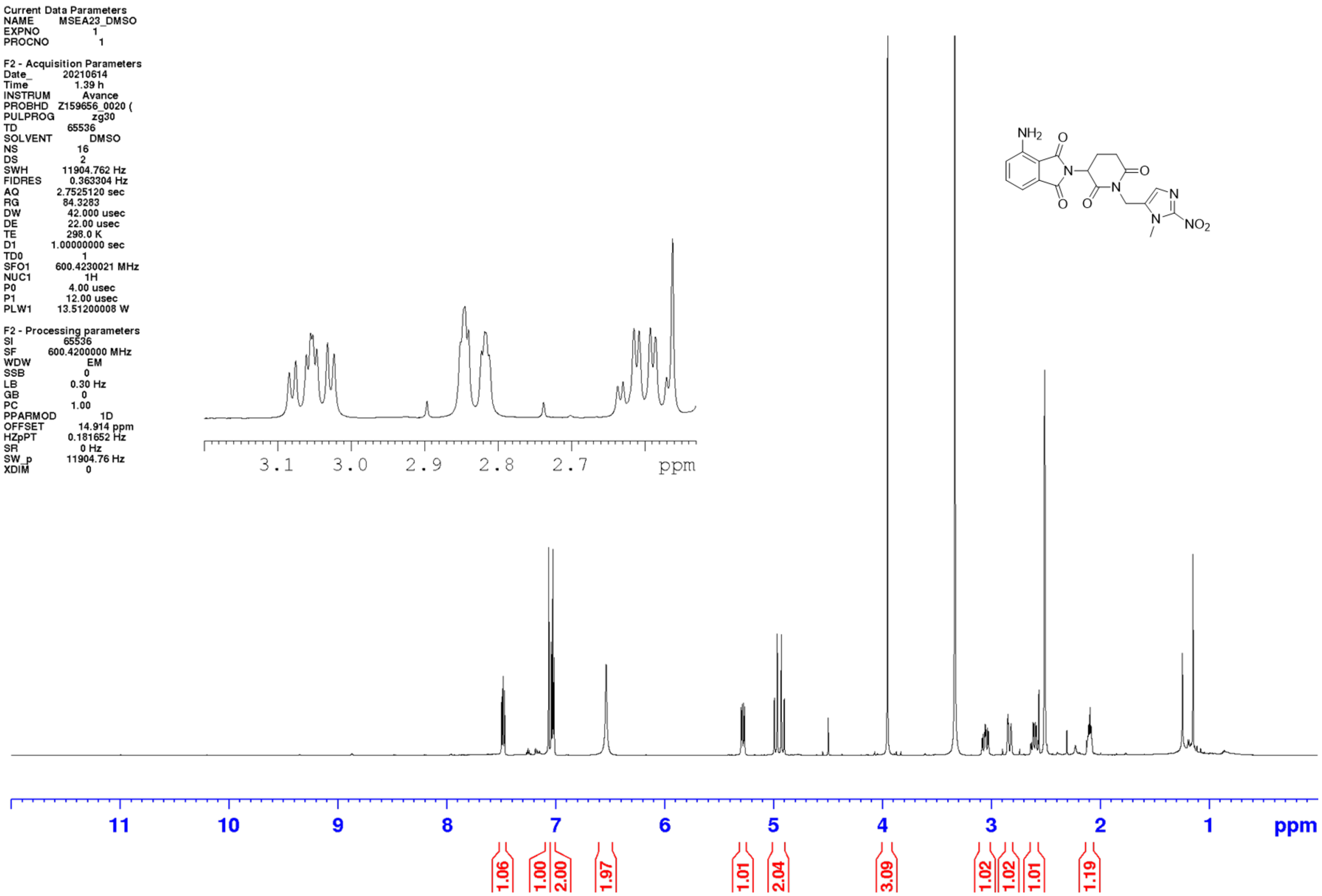

^{13}C NMR (151 MHz; D$_6$-DMSO): compound **12** (NI-POMA)

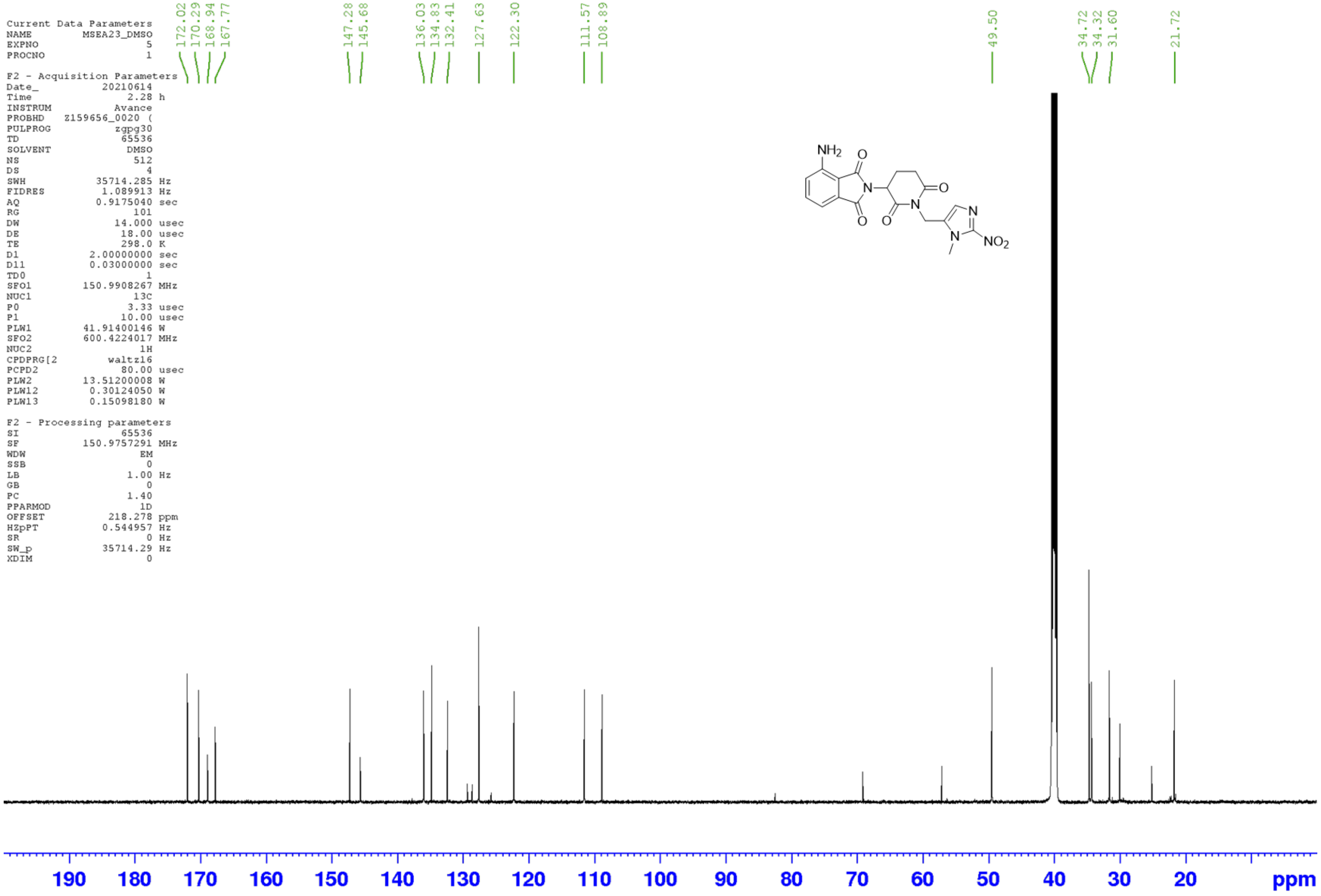

^{1}H NMR (600 MHz; CD_3OD): compound **17**

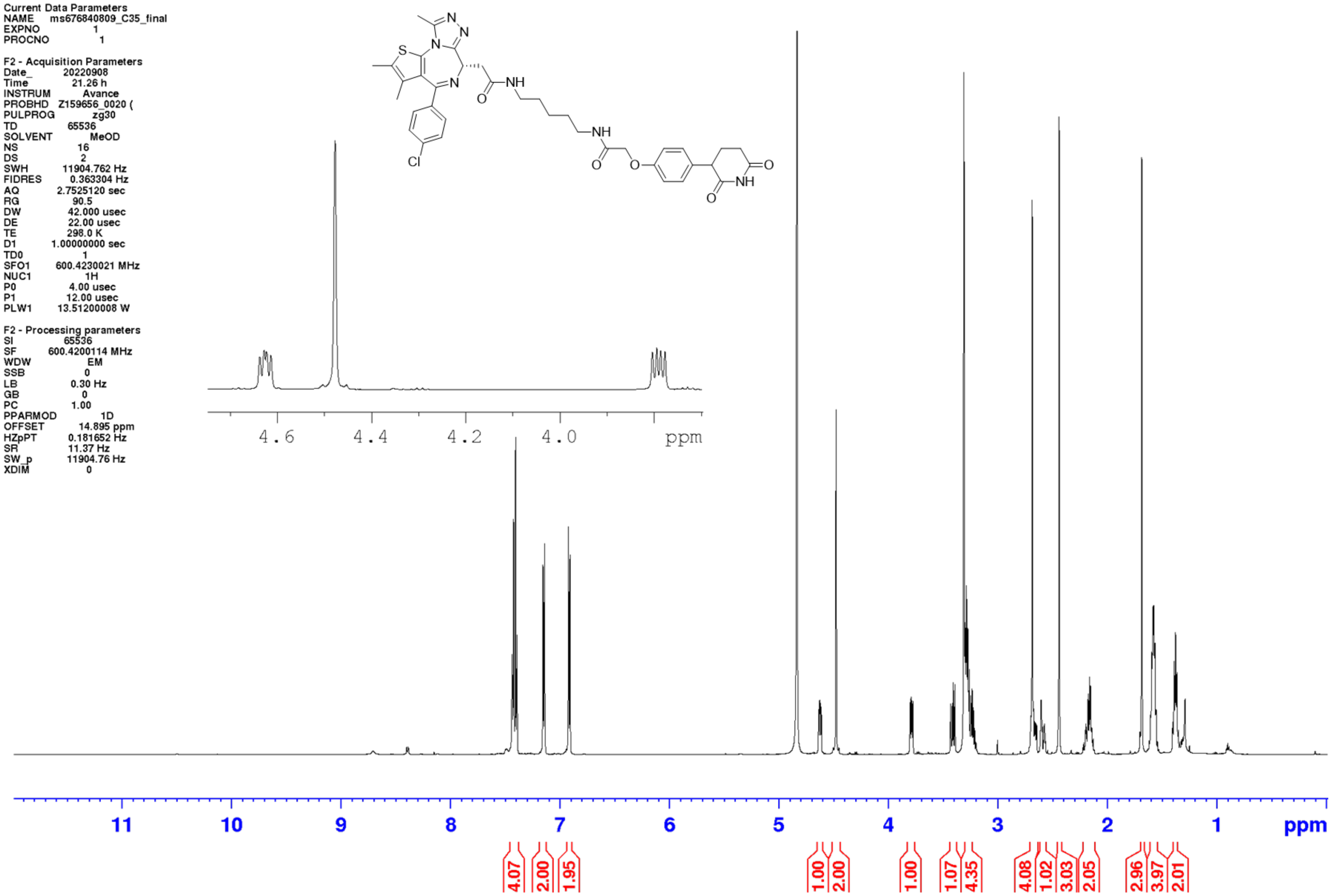

^{13}C NMR (151 MHz; CD_3OD): compound **17**

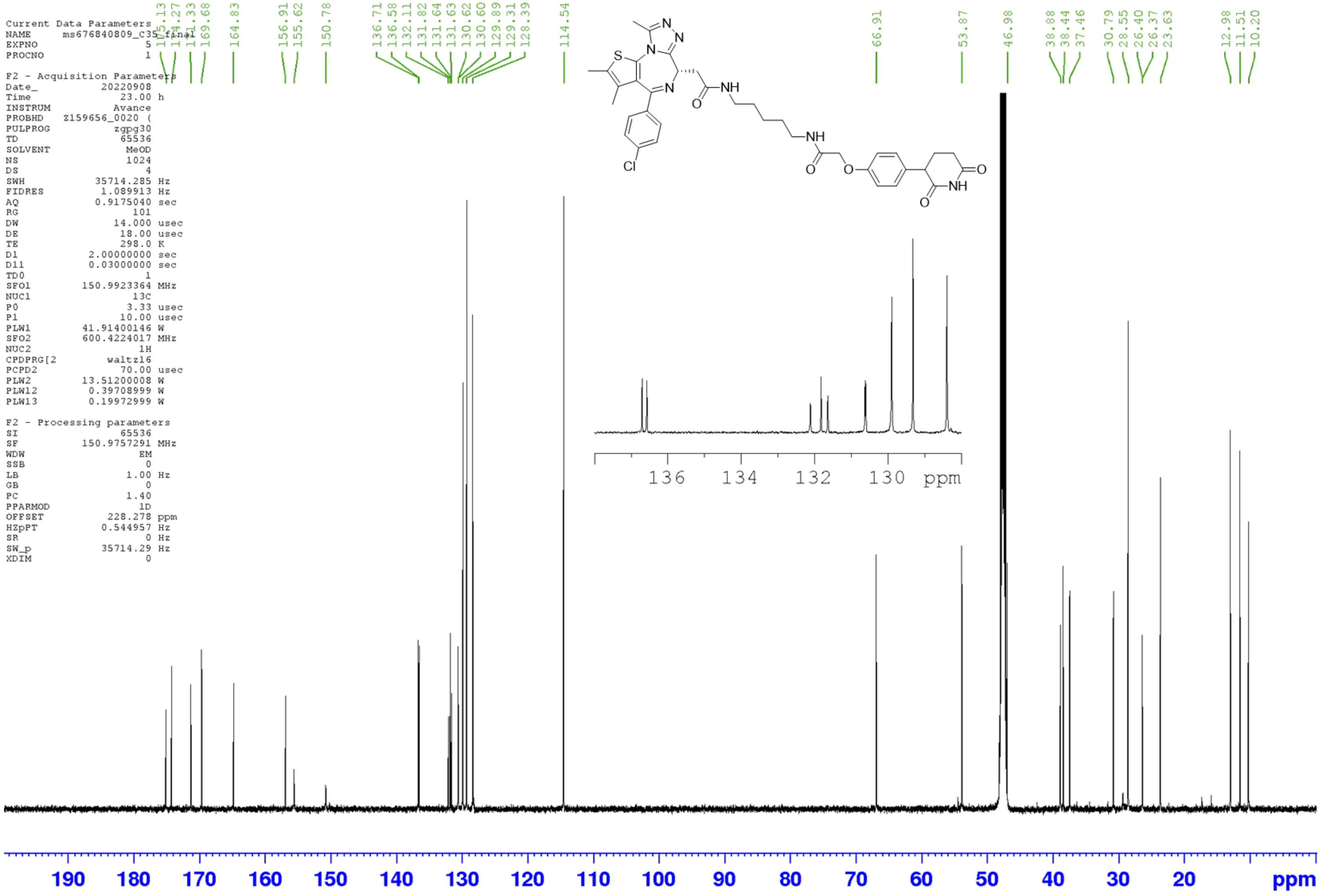

^{1}H NMR (400 MHz; $CDCl_3$): compound **56**

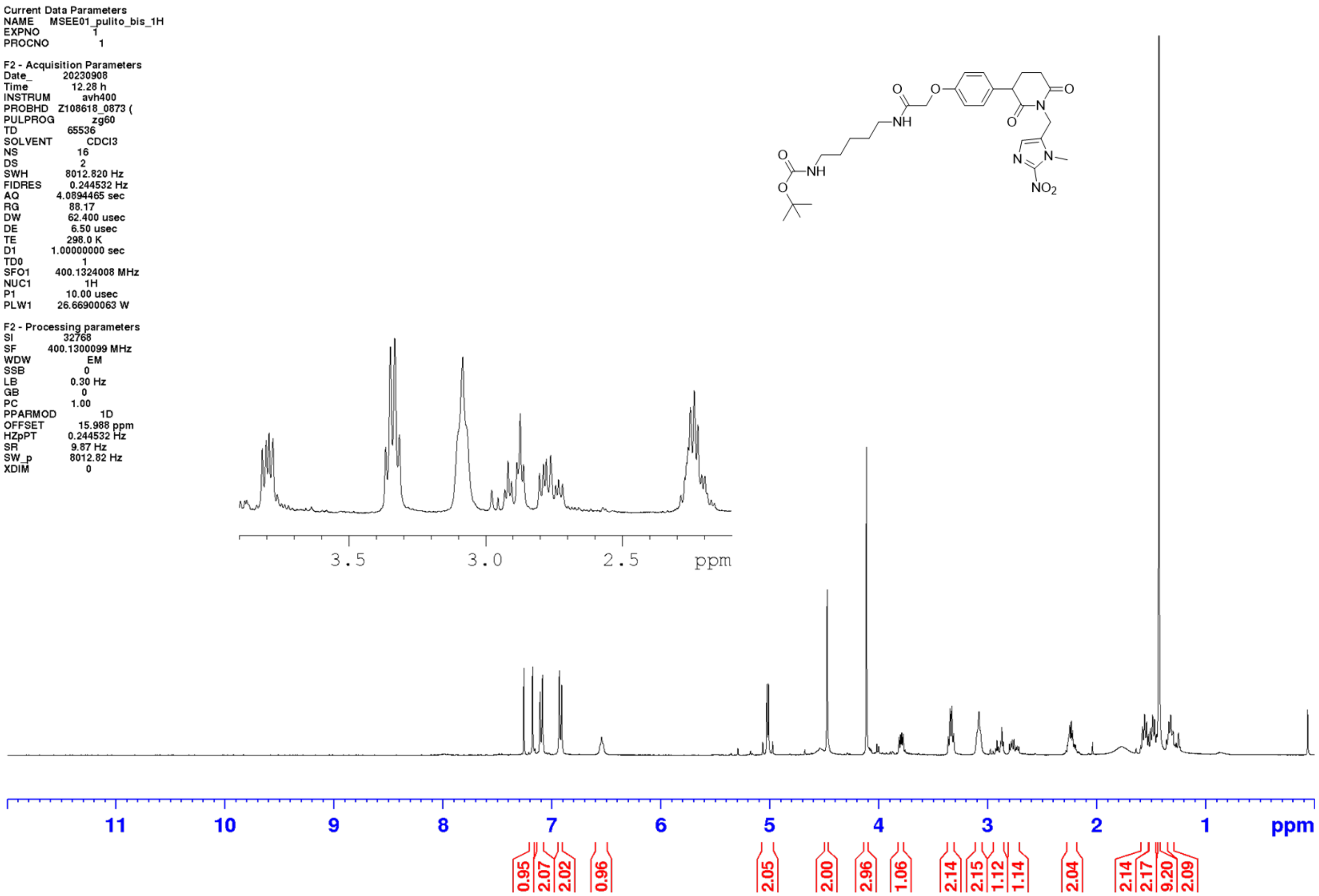

^{13}C NMR (126 MHz; $CDCl_3$): compound **56**

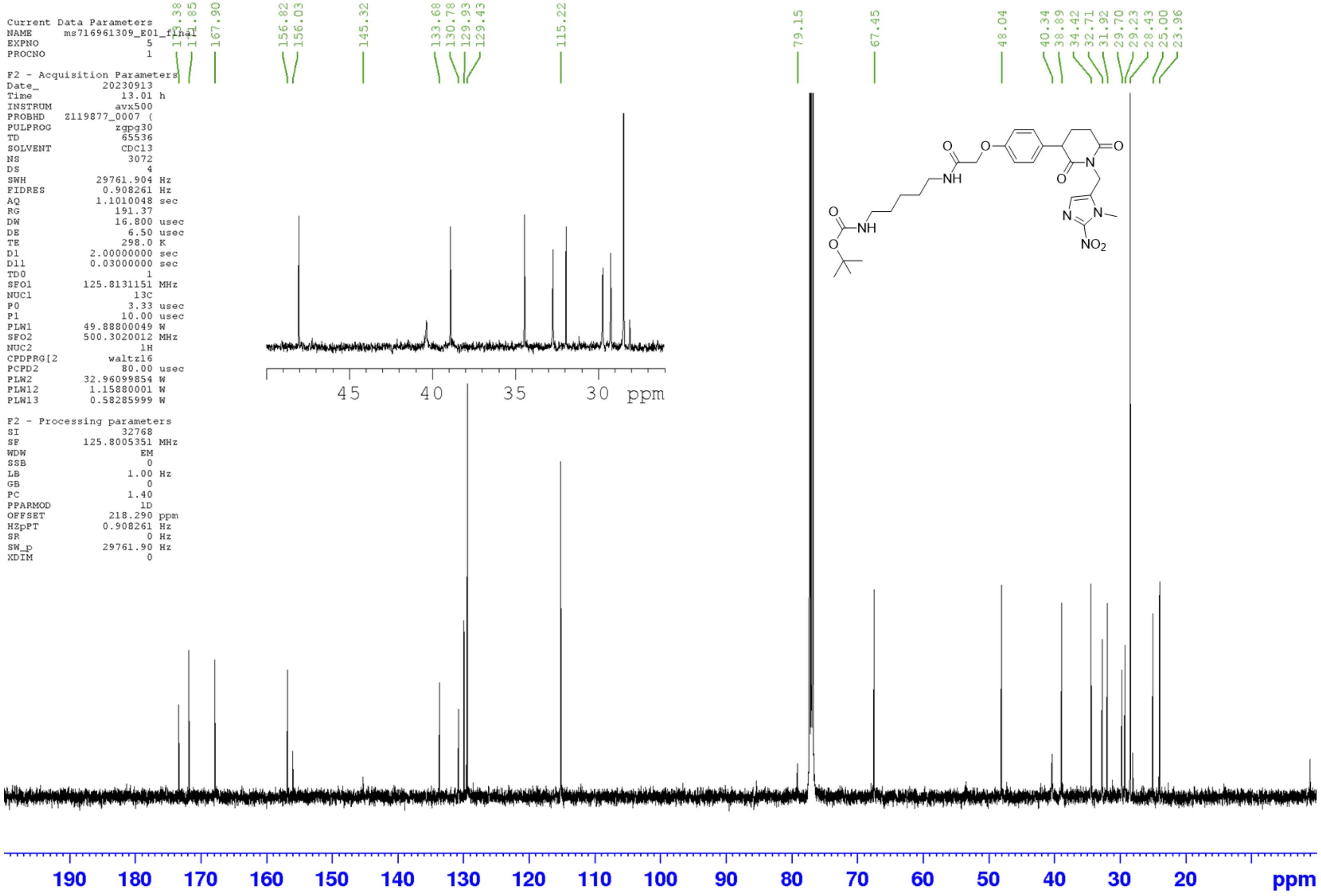

^{1}H NMR (600 MHz; $CDCl_3$): compound **57**

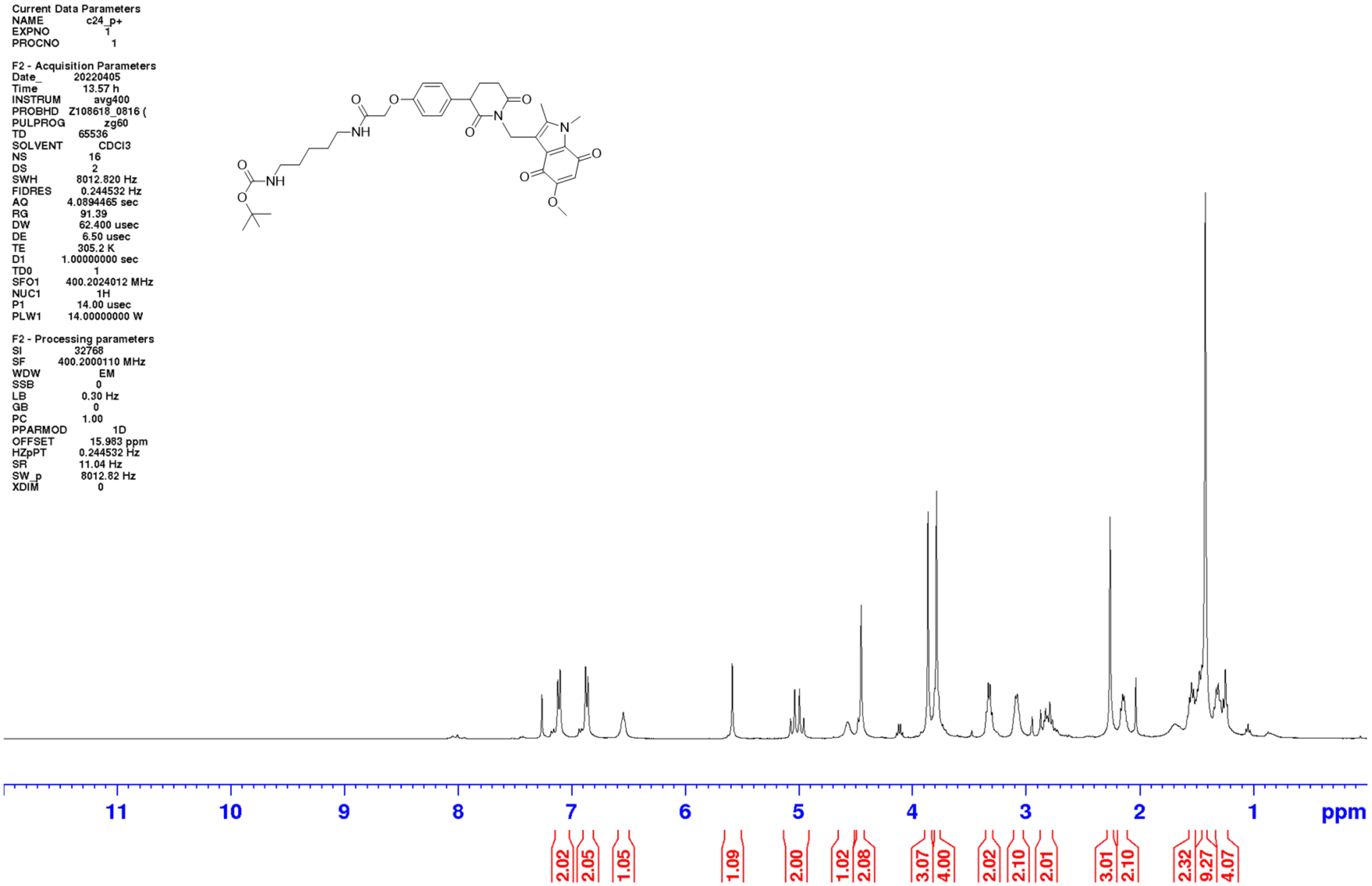

^{13}C NMR (151 MHz; $CDCl_3$): compound **57**

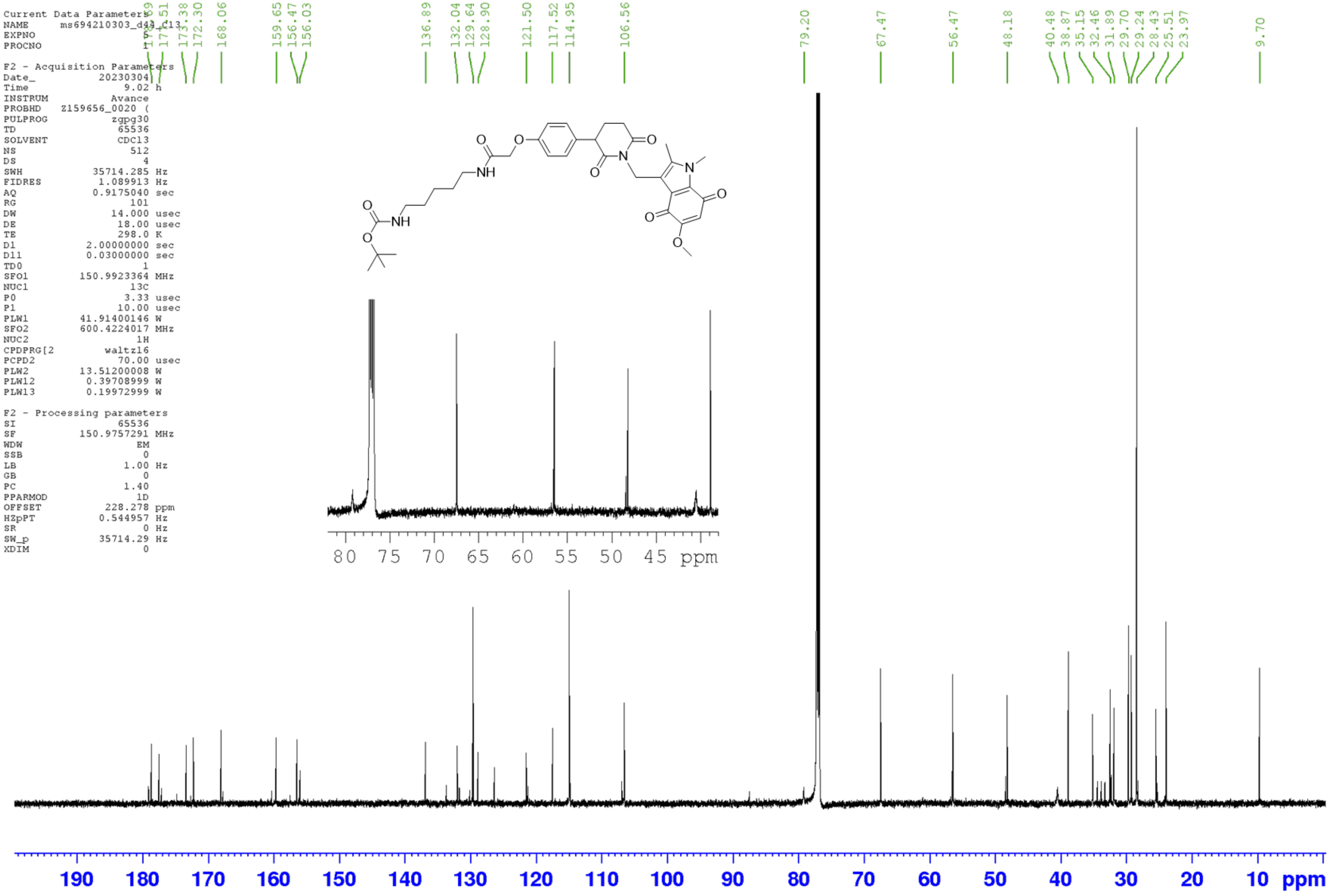

^{1}H NMR (400 MHz; CD_3OD): compound **58**

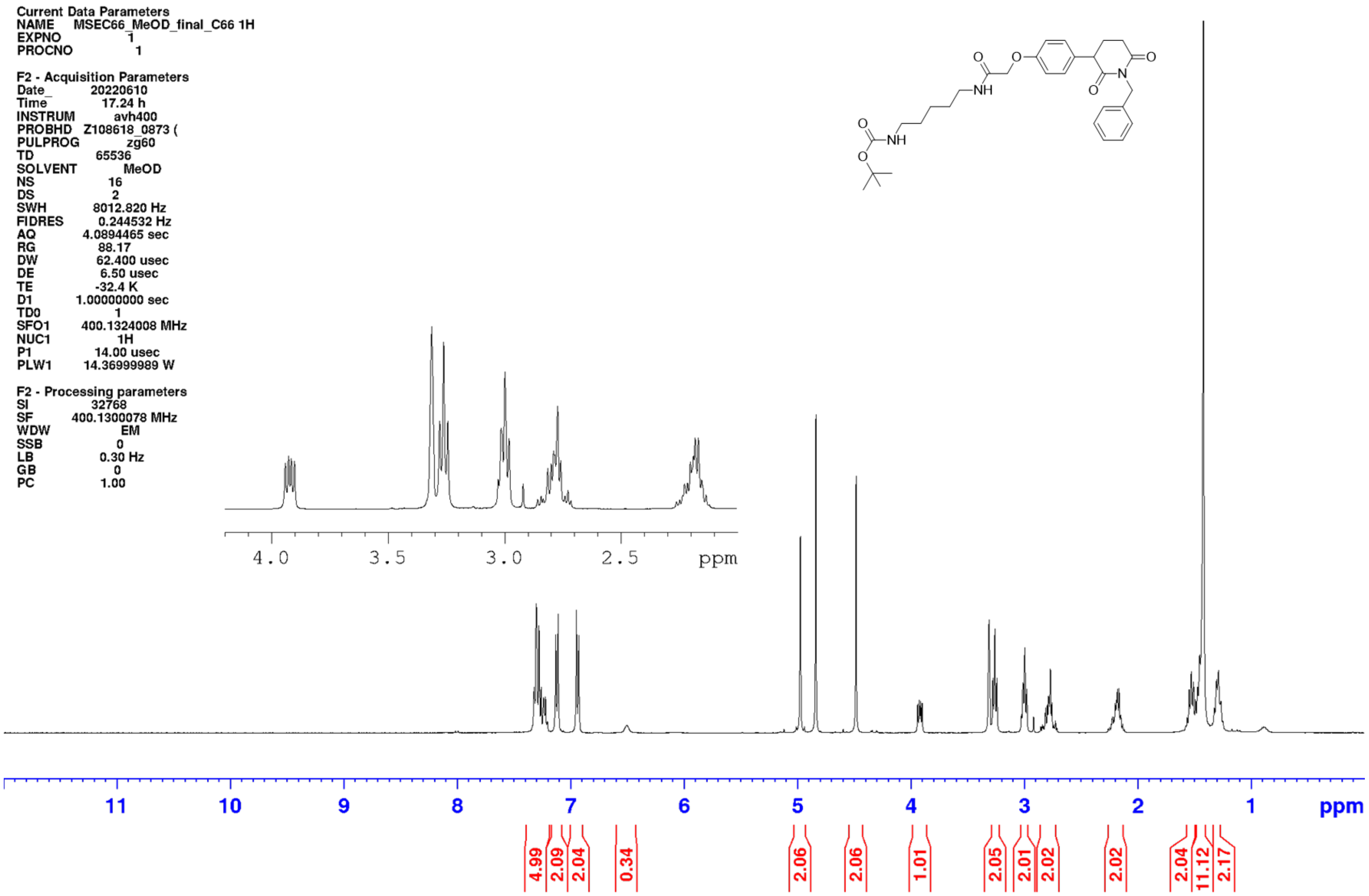

^{13}C NMR (101 MHz; CD_3OD): compound **58**

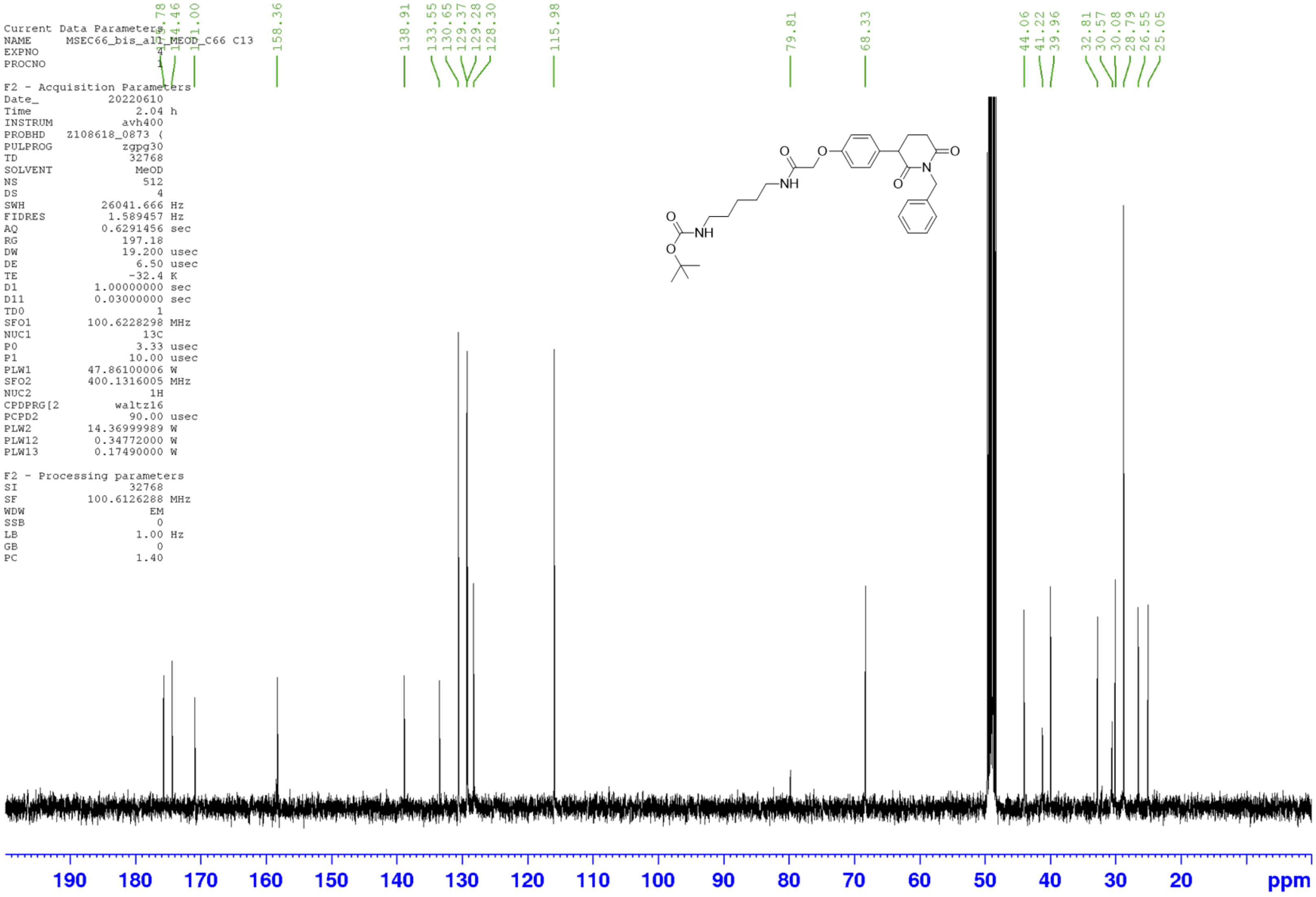

^{1}H NMR (400 MHz; CD_3OD): compound **15** (NI-CRBN)

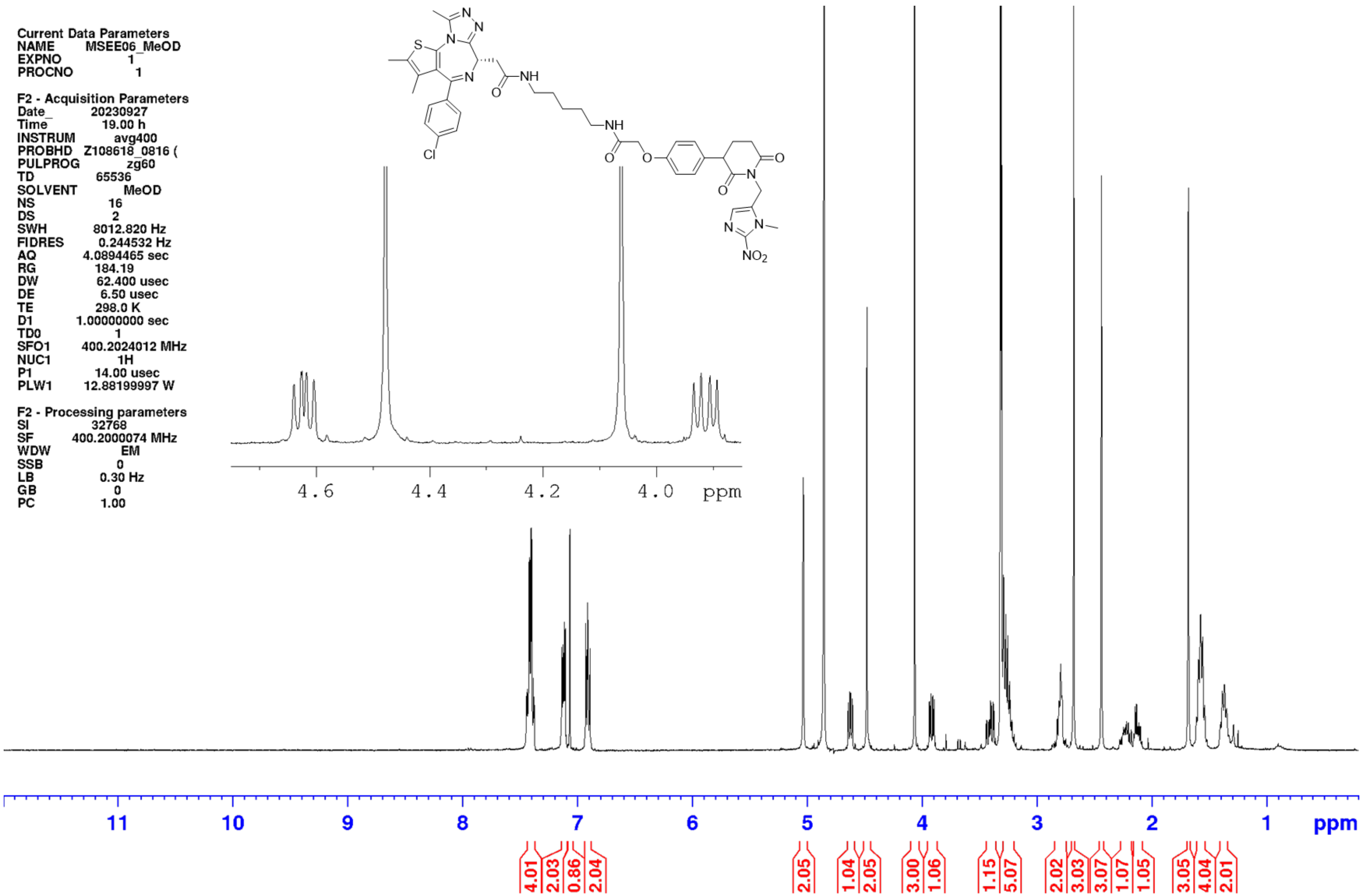

^{13}C NMR (151 MHz; CD_3OD): compound **15** (NI-CRBN)

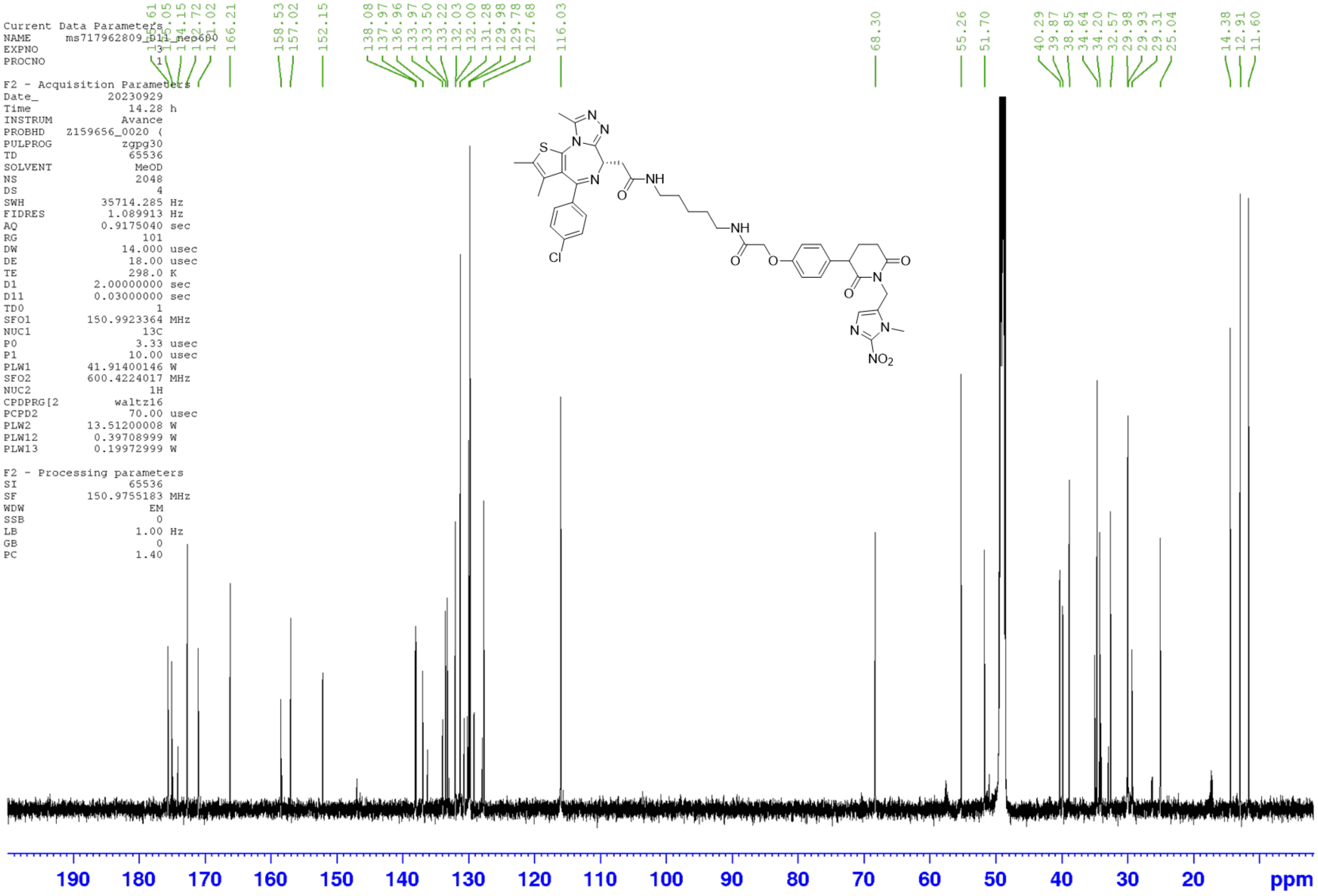

^{1}H NMR (600 MHz; CD_3OD): compound **16** (IQ-CRBN)

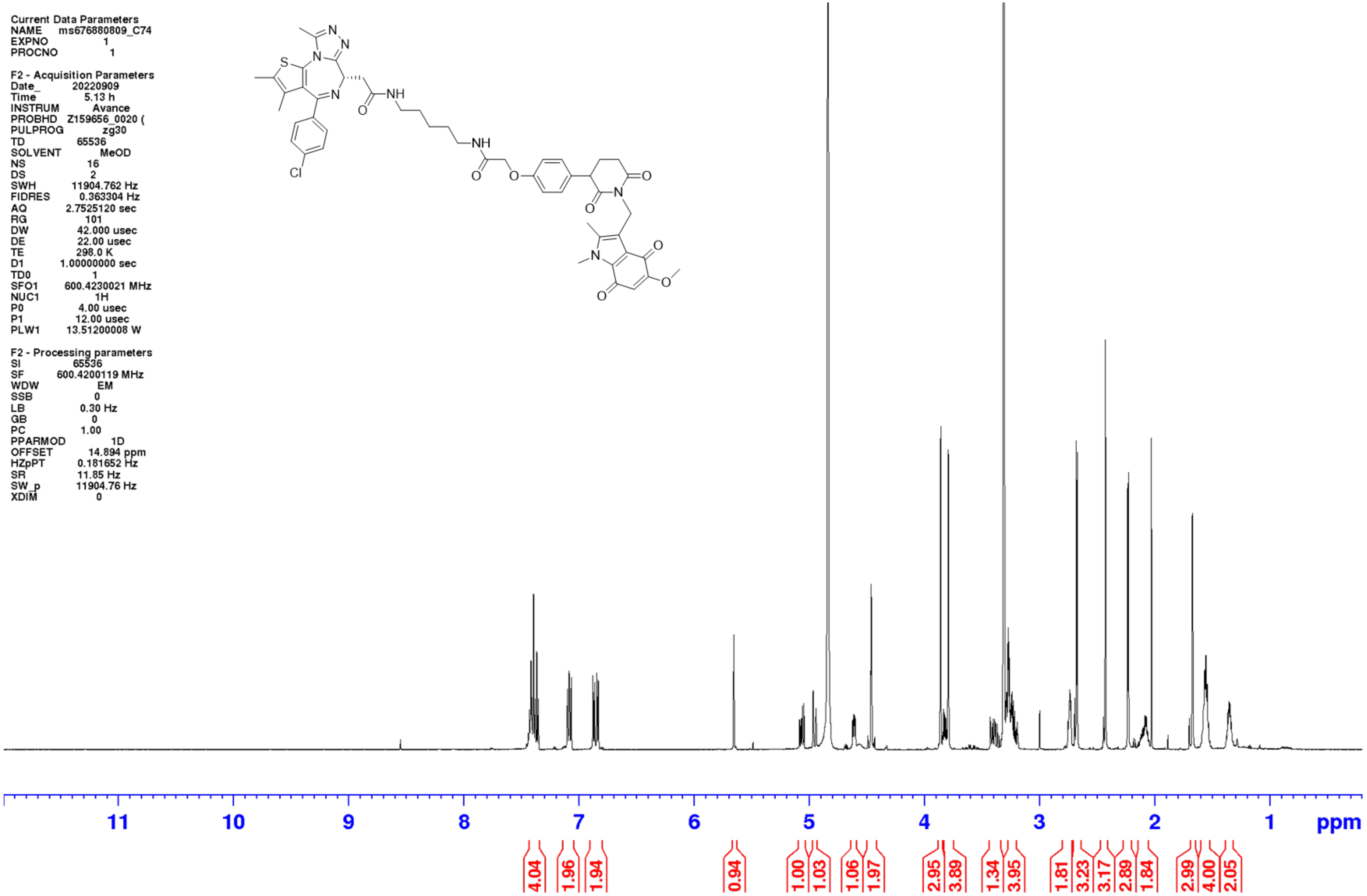

^{13}C NMR (151 MHz; CD_3OD): compound **16** (IQ-CRBN)

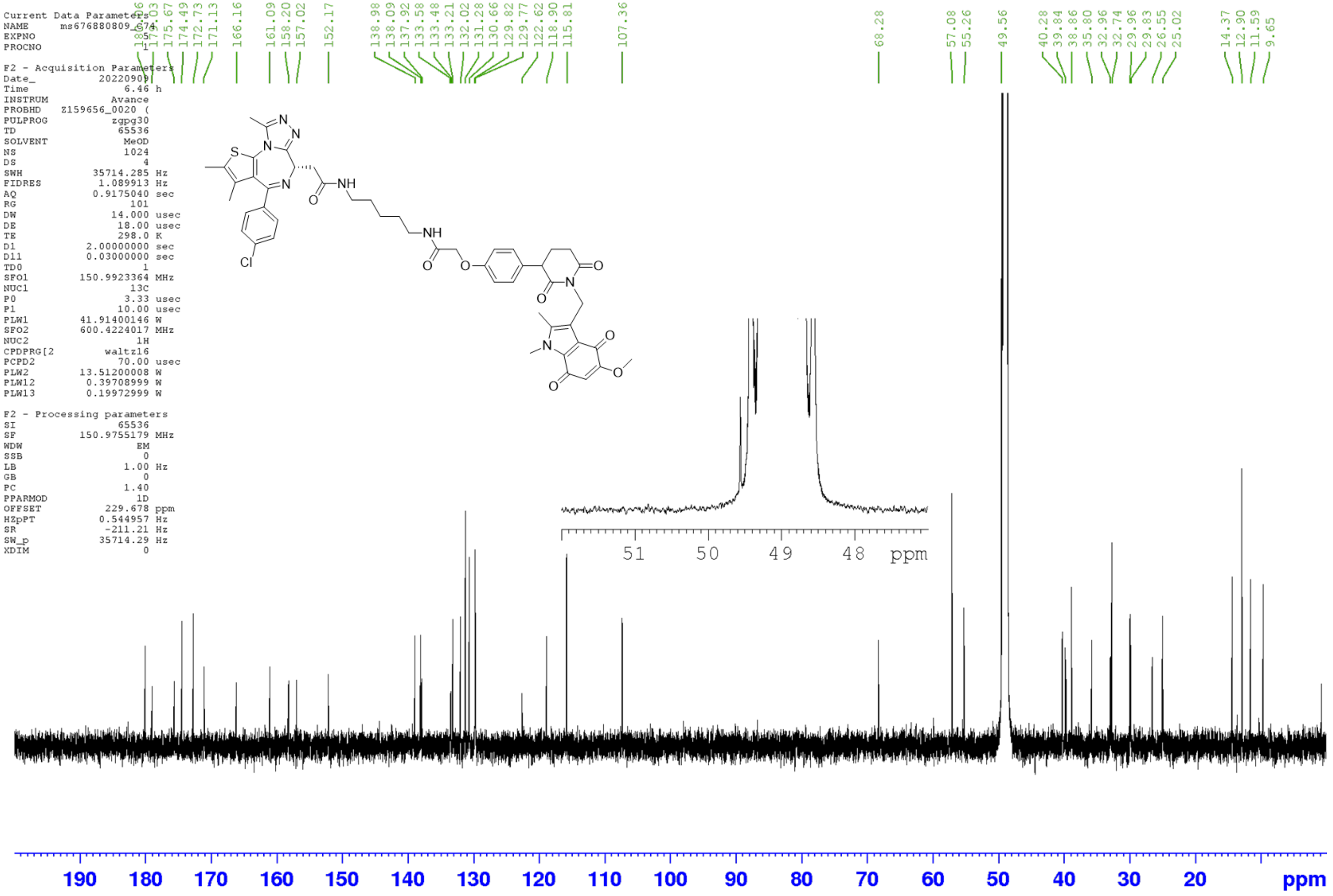

^{1}H NMR (600 MHz; CD_3OD): compound **18** (Bn-CRBN)

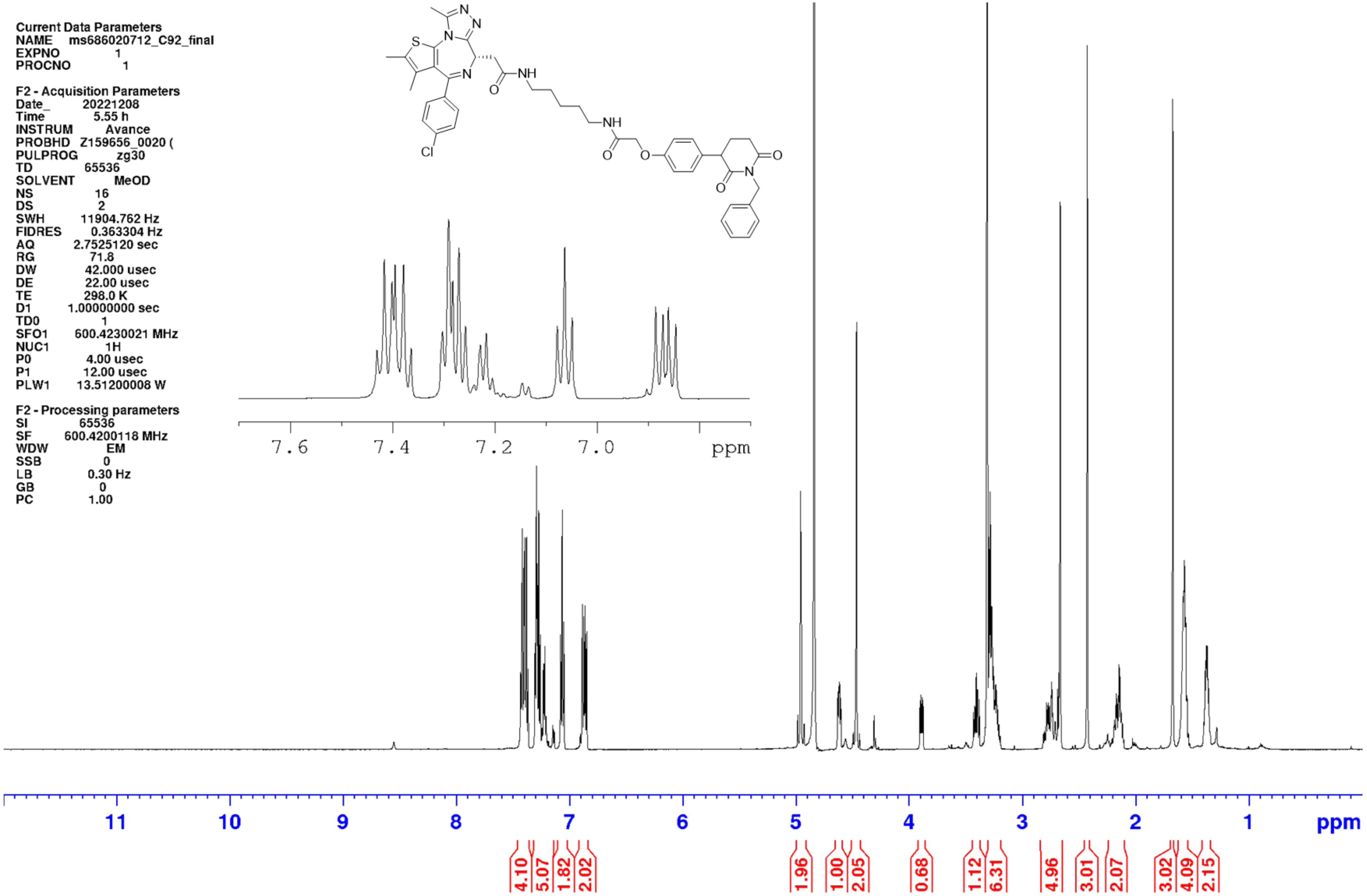

^{13}C NMR (151 MHz; CD_3OD): compound **18** (Bn-CRBN)

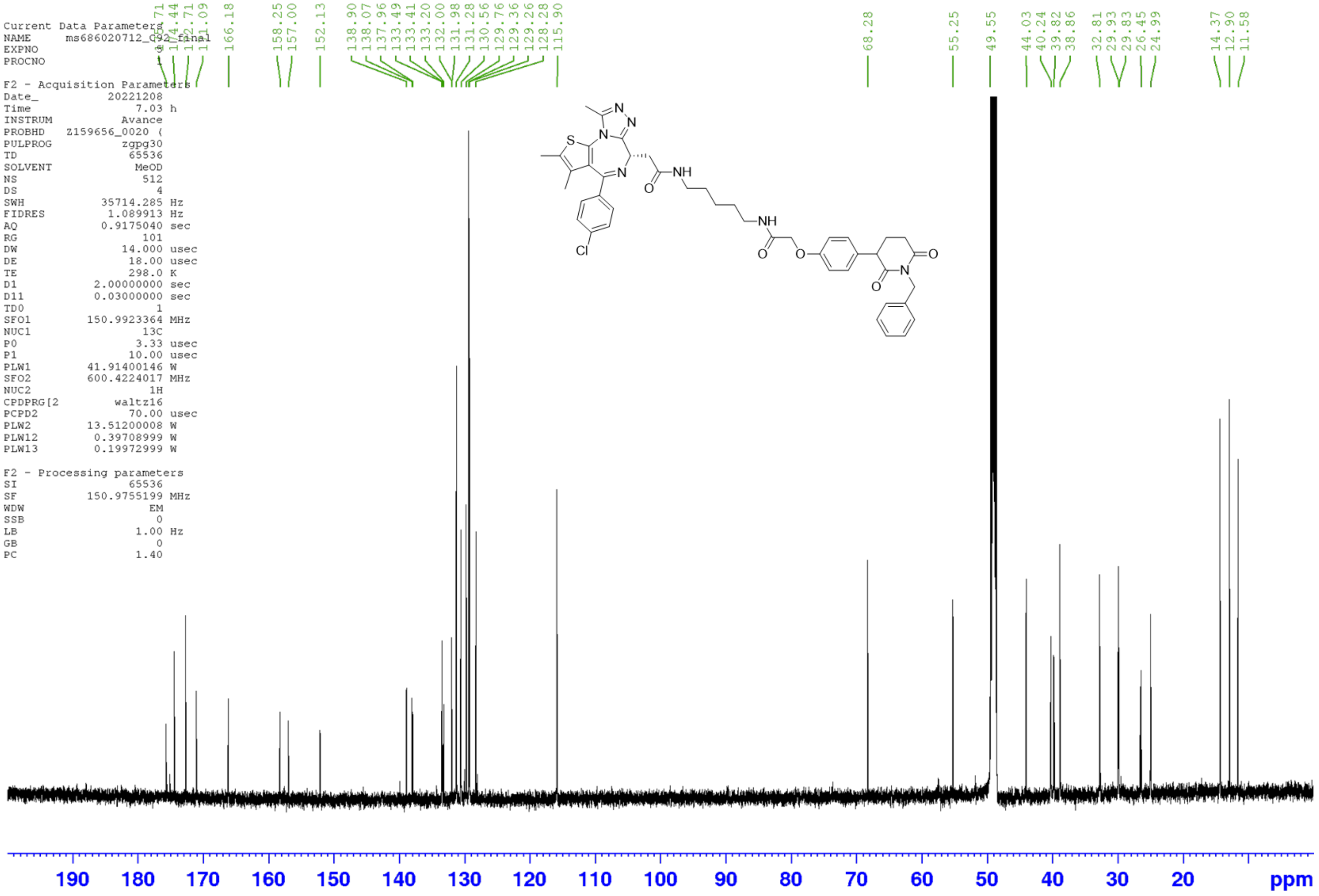

5. HPLC traces

Compound 6 (NI-VHL)

@ 254 and 220 nM - as described in the general chemistry method for analytical HPLC, method A.

**Instrument:**	Analytical HPLC
**Acq. method:**	MSE_PURITY_CHECK_10MICRO.M
**Injection date:**	2022-08-17 16:56:39+01:00
**Sample name:**	MSED09_postHPLC_final
**Injection volume:**	10.000

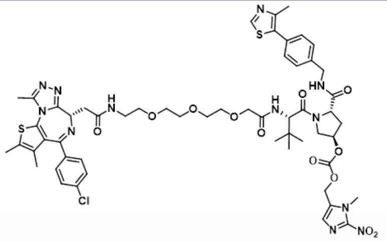

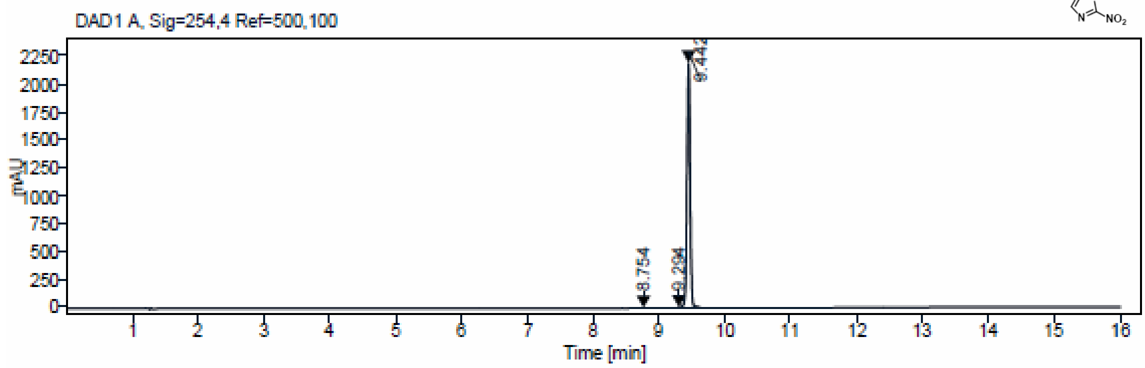

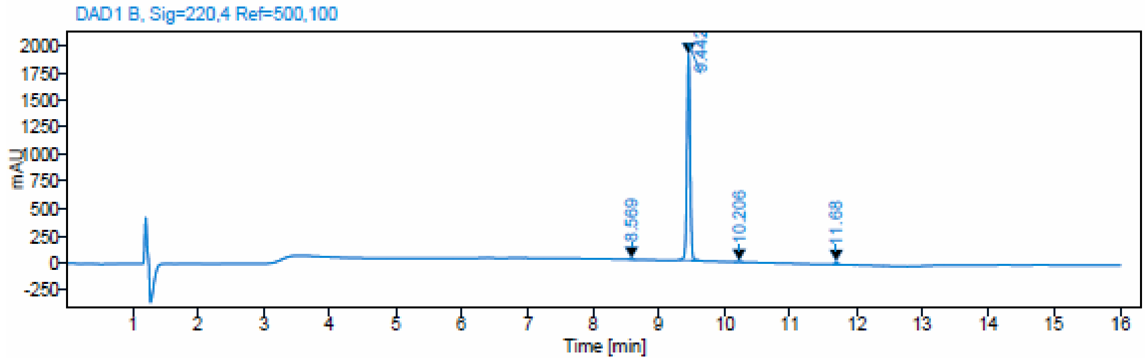

Signal: DAD1 A, Sig=254,4 Ref=500,100

RT [min]	Type	Width [min]	Area	Height	Area%	Name
8.754	BB	0.0510	5.8372	1.7030	0.0792	
9.294	BV E	0.0608	13.3781	2.9554	0.1815	
9.442	VB R	0.0510	7353.2383	2198.0854	99.7394	
		Sum	7372.4535			

Signal: DAD1 B, Sig=220,4 Ref=500,100

RT [min]	Type	Width [min]	Area	Height	Area%	Name
8.569	MM	0.0534	15.2732	4.7652	0.2351	
9.442	MM	0.0564	6385.4624	1885.9448	98.3069	
10.206	MM	0.0676	26.3766	6.5050	0.4061	
11.680	MM	0.0476	68.3249	23.9031	1.0519	
		Sum	6495.4371			

Compound 7 (IQ-VHL)

@ 254 and 220 nM - as described in the general chemistry method for analytical HPLC, method A.

**Instrument:**	Analytical HPLC
**Acq. method:**	MSE_220_254_280_310_5MICROL.M
**Injection date:**	2022-07-30 14:43:43+01:00
**Sample name:**	MSEC89_puritycheckafterHPLCpurification
**Injection volume:**	5.000

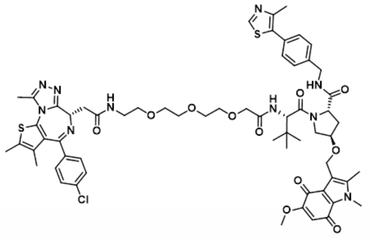

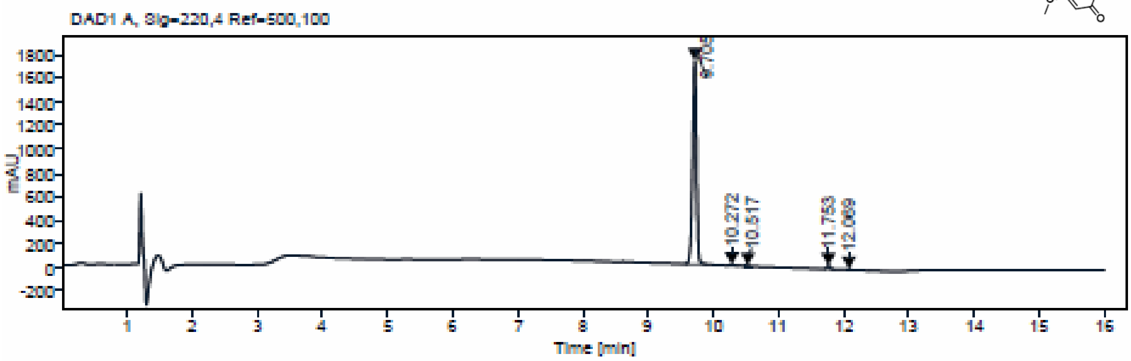

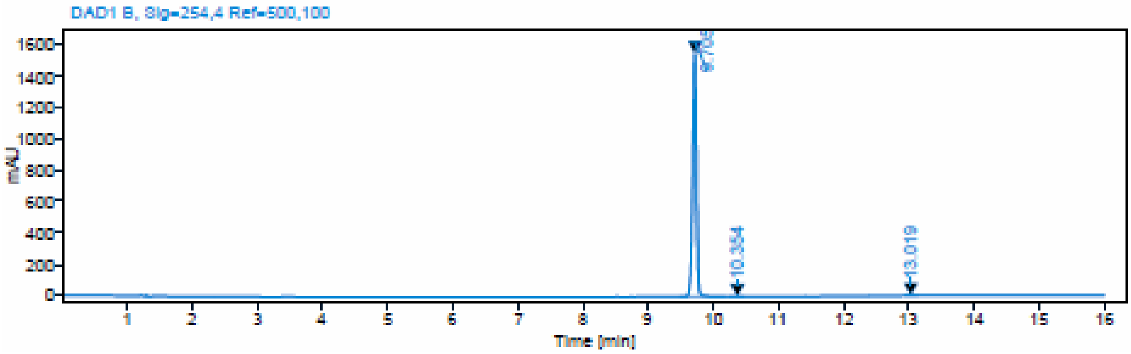

Signal: DAD1 A, Sig=220,4 Ref=500,100

RT [min]	Type	Width [min]	Area	Height	Area%	Name
9.705	MM	0.0677	7004.7178	1724.0402	97.2424	
10.272	MM	0.0727	48.3840	11.0914	0.6717	
10.517	MM	0.0595	34.7759	9.7336	0.4828	
11.753	MM	0.0514	93.8572	30.4587	1.3030	
12.069	MM	0.0500	21.6234	7.2123	0.3002	
		Sum	7203.3582			

Signal: DAD1 B, Sig=254,4 Ref=500,100

RT [min]	Type	Width [min]	Area	Height	Area%	Name
9.705	BV R	0.0595	6176.1753	1550.0900	99.7639	
10.354	BB	0.0866	8.7553	1.3520	0.1414	
13.019	BB	0.0586	5.8637	1.5003	0.0947	
		Sum	6190.7943			

Compound 9 (Bn-VHL)

@ 254 and 220 nM - as described in the general chemistry method for analytical HPLC, method A.

**Instrument:**	Analytical HPLC
**Acq. method:**	MSE_PURITY_CHECK_10MICRO.M
**Injection date:**	2022-08-18 10:31:00+01:00
**Sample name:**	MSED07_2_prep
**Injection volume:**	10.000

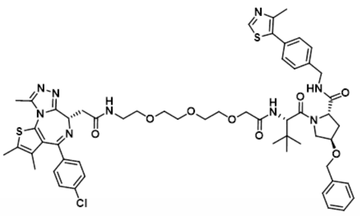

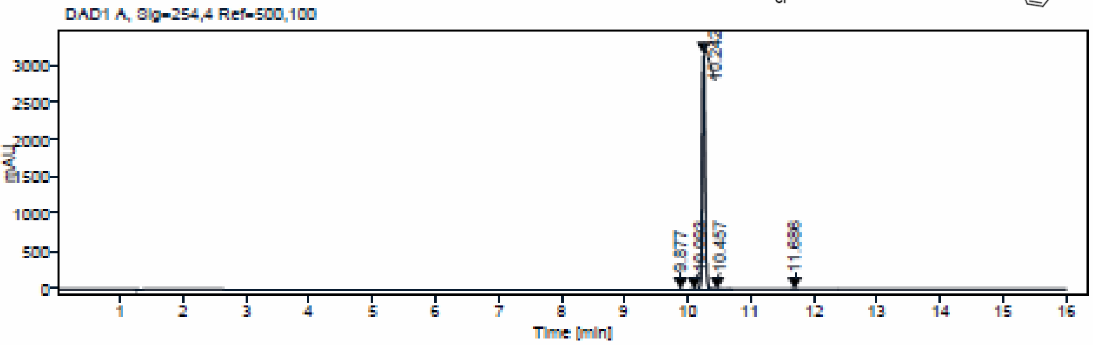

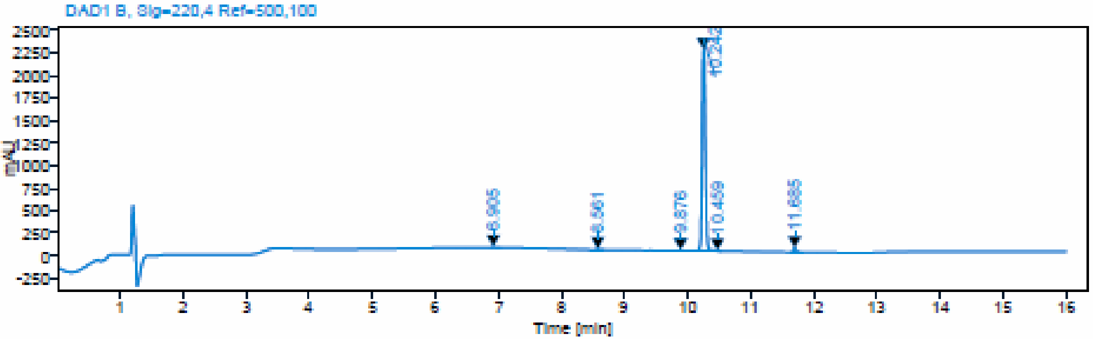

Signal: DAD1 A, Sig=254,4 Ref=500,100

RT [min]	Type	Width [min]	Area	Height	Area%	Name
9.877	VB R	0.0554	12.7508	3.3523	0.1262	
10.093	VV E	0.0522	7.9606	2.2031	0.0788	
10.242	VV R	0.0501	10057.4170	3159.9097	99.5427	
10.457	VB E	0.0651	19.5214	4.2123	0.1932	
11.686	BB	0.0447	5.9734	2.0670	0.0591	
		Sum	10103.6232			

Signal: DAD1 B, Sig=220,4 Ref=500,100

RT [min]	Type	Width [min]	Area	Height	Area%	Name
6.905	MM	0.0600	33.3836	9.2705	0.4143	
8.561	MM	0.0510	21.8517	7.1443	0.2712	
9.876	MM	0.0538	15.4487	4.7845	0.1917	
10.242	MM	0.0585	7837.0815	2232.1372	97.2672	
10.459	MM	0.0521	14.8926	4.7599	0.1848	
11.685	MM	0.0501	134.6153	44.7569	1.6707	
		Sum	8057.2734			

Compound 10 (NB-POMA)

@ 254 and 220 nM - as described in the general chemistry method for analytical HPLC, method B.

**Instrument:**	Analytical HPLC
**Acq. method:**	MSE_PURITY_CHECK_5MICRO.M
**Injection date:**	2023-04-13 13:21:00+01:00
**Sample name:**	NB-poma_purity_2023
**Injection volume:**	5.000

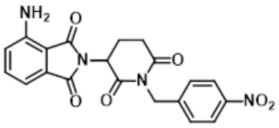

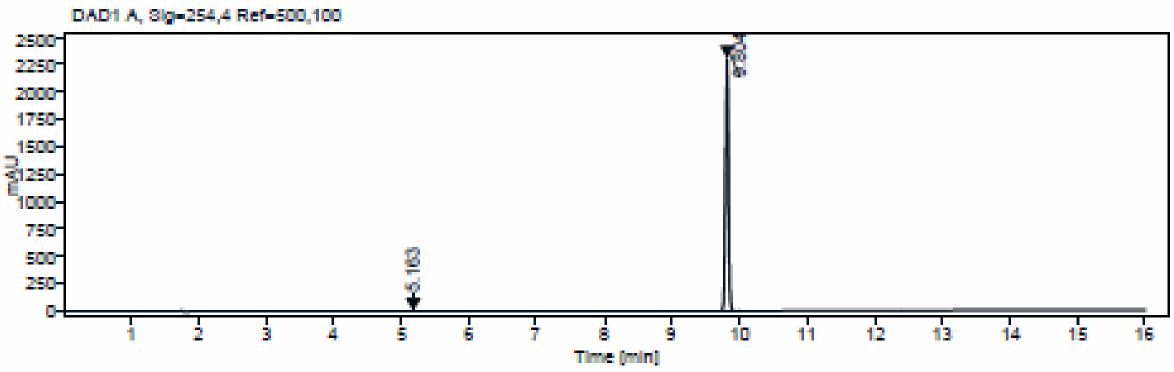

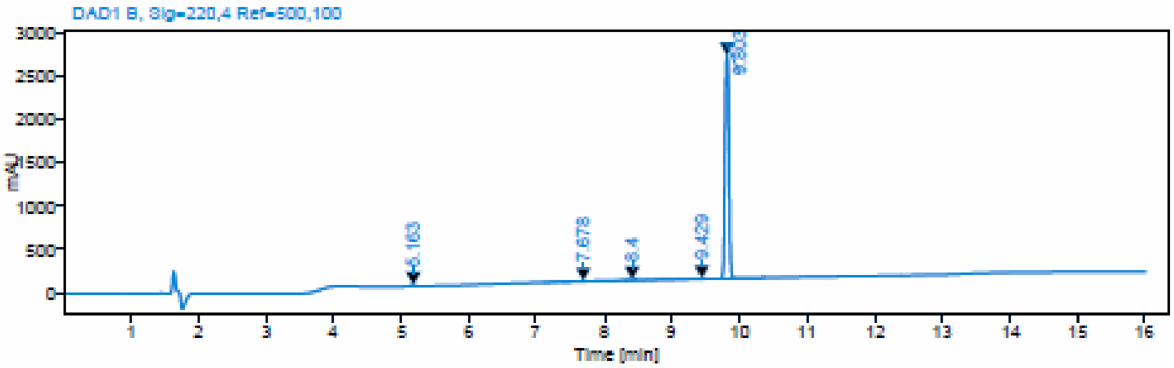

Signal: DAD1 A, Sig=254,4 Ref=500,100

RT [min]	Type	Width [min]	Area	Height	Area%	Name
5.163	BB	0.0591	20.7592	5.6158	0.2893	
9.804	BV R	0.0480	7154.6118	2320.6958	99.7107	
		Sum	7175.3710			

Signal: DAD1 B, Sig=220,4 Ref=500,100

RT [min]	Type	Width [min]	Area	Height	Area%	Name
5.163	BB	0.0591	69.5209	18.8181	0.6139	
7.678	VV X	0.1106	17.8261	2.0674	0.1574	
8.400	VV X	0.0518	13.6835	4.0105	0.1208	
9.429	VV E	0.0483	7.7329	2.3575	0.0683	
9.803	VV R	0.0701	11215.4102	2555.2595	99.0395	
		Sum	11324.1735			

Compound 11 (pomalidomide)

@ 254 and 220 nM - as described in the general chemistry method for analytical HPLC, method B.

**Instrument:**	Analytical HPLC
**Acq. method:**	MSE_PURITY_CHECK_5MICRO.M
**Injection date:**	2023-04-13 12:18:11+01:00
**Sample name:**	poma_purity_2023
**Injection volume:**	5.000

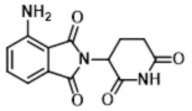

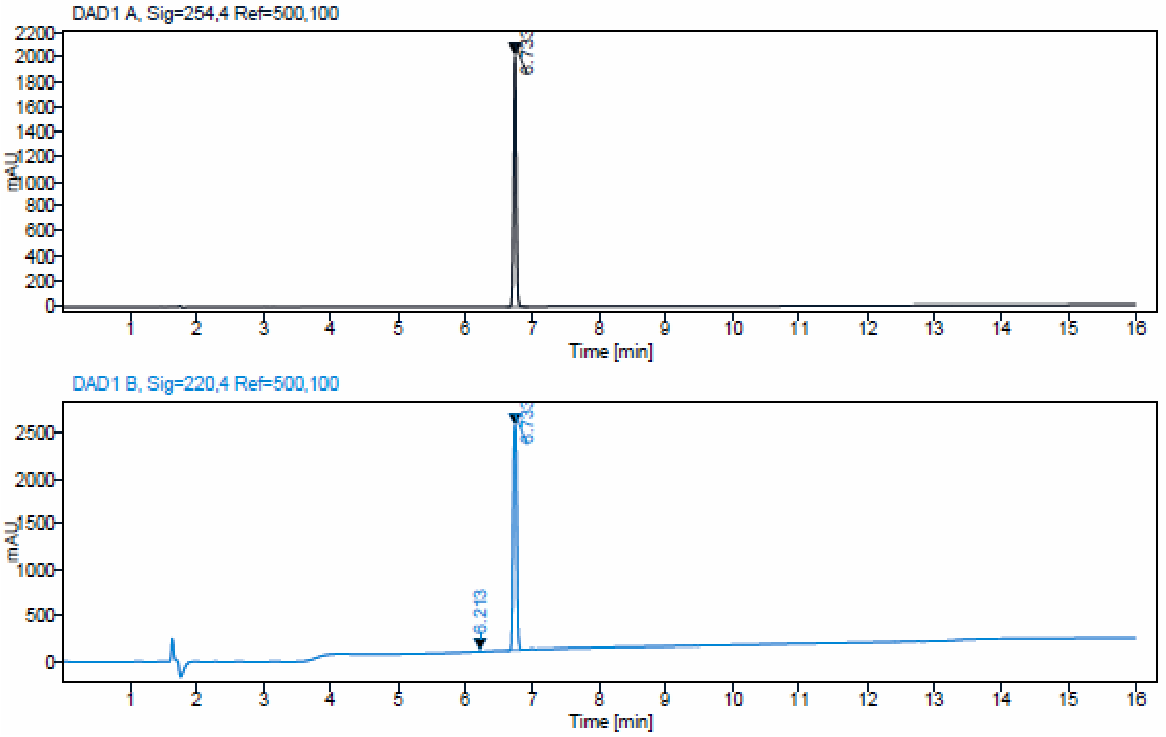

Signal: DAD1 A, Sig=254,4 Ref=500,100

RT [min]	Type	Width [min]	Area	Height	Area%	Name
6.733	BB	0.0436	5637.8555	2016.5194	100.0000	
		Sum	5637.8555			

Signal: DAD1 B, Sig=220,4 Ref=500,100

RT [min]	Type	Width [min]	Area	Height	Area%	Name
6.213	BB	0.0387	11.3157	4.5924	0.1169	
6.733	BB	0.0650	9667.1963	2452.8262	99.8831	
		Sum	9678.5120			

Compound 12 (NI-POMA)

@ 254 and 220 nM - as described in the general chemistry method for analytical HPLC, method B.

Instrument: Analytical HPLC
Acq. method: MSE_PURITY_CHECK_5MICRO.M
Injection date: 2023-04-13 13:00:04+01:00
Sample name: NI-poma_purity_2023
Injection volume: 5.000

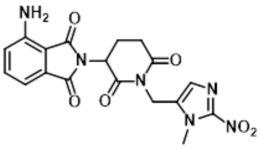

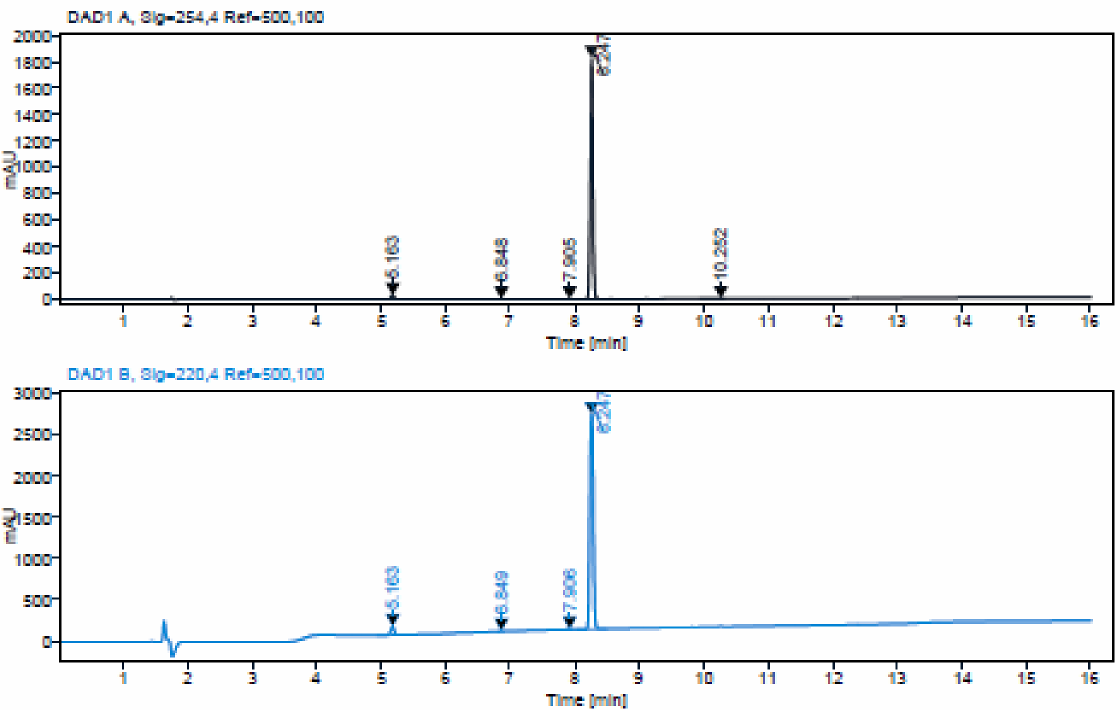

Signal: DAD1 A, Sig=254,4 Ref=500,100

RT [min]	Type	Width [min]	Area	Height	Area%	Name
5.163	BB	0.0591	111.0945	30.0678	1.9878	
6.848	BB	0.0424	6.8411	2.3888	0.1224	
7.905	VB	0.0505	5.4178	1.6020	0.0969	
8.247	BV R	0.0456	5455.5469	1839.4562	97.6138	
10.252	BV	0.0671	10.0080	2.1182	0.1791	
		Sum	5588.9083			

Signal: DAD1 B, Sig=220,4 Ref=500,100

RT [min]	Type	Width [min]	Area	Height	Area%	Name
5.163	BB	0.0592	370.8197	100.2592	3.3825	
6.849	MM	0.0466	31.3343	11.2133	0.2858	
7.906	MM	0.0412	13.1242	5.3144	0.1197	
8.247	VV R	0.0664	10547.5254	2589.1194	96.2119	
		Sum	10962.8037			

Compound 15 (NI-CRBN)

@ 254 and 220 nM - as described in the general chemistry method for analytical HPLC, method A.

**Instrument:**	Analytical HPLC
**Acq. method:**	MSE_PURITY_CHECK_20MICROL.M
**Injection date:**	2022-09-06 16:04:08+01:00
**Sample name:**	MSED11_prep_check_20uL
**Injection volume:**	20.000

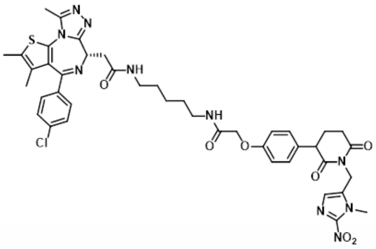

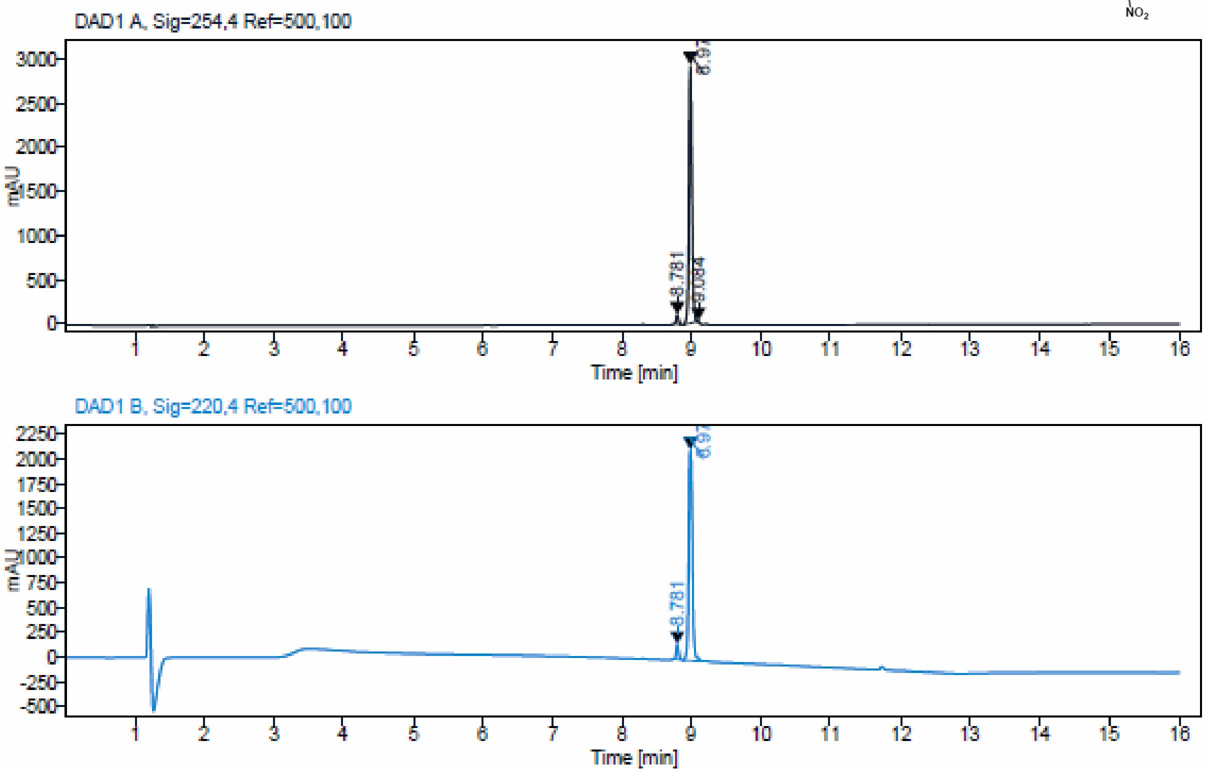

Signal: DAD1 A, Sig=254,4 Ref=500,100

RT [min]	Type	Width [min]	Area	Height	Area%	Name
8.781	MM	0.0420	257.3625	102.1546	2.9620	
8.970	MM	0.0480	8409.2012	2918.6543	96.7826	
9.084	MM	0.0270	22.1930	13.7159	0.2554	
		Sum	8688.7568			

Signal: DAD1 B, Sig=220,4 Ref=500,100

RT [min]	Type	Width [min]	Area	Height	Area%	Name
8.781	MM	0.0417	372.5149	148.7098	4.5554	
8.970	MM	0.0609	7804.9990	2136.0867	95.4446	
		Sum	8177.5139			

Compound 16 (IQ-CRBN)

@ 254 and 220 nM - as described in the general chemistry method for analytical HPLC, method A.

**Instrument:**	Analytical HPLC
**Acq. method:**	MSE_PURITY_CHECK_10MICRO.M
**Injection date:**	2022-08-17 16:35:12+01:00
**Sample name:**	MSEC74_postHPLC_final
**Injection volume:**	10.000

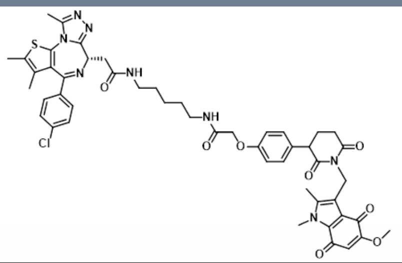

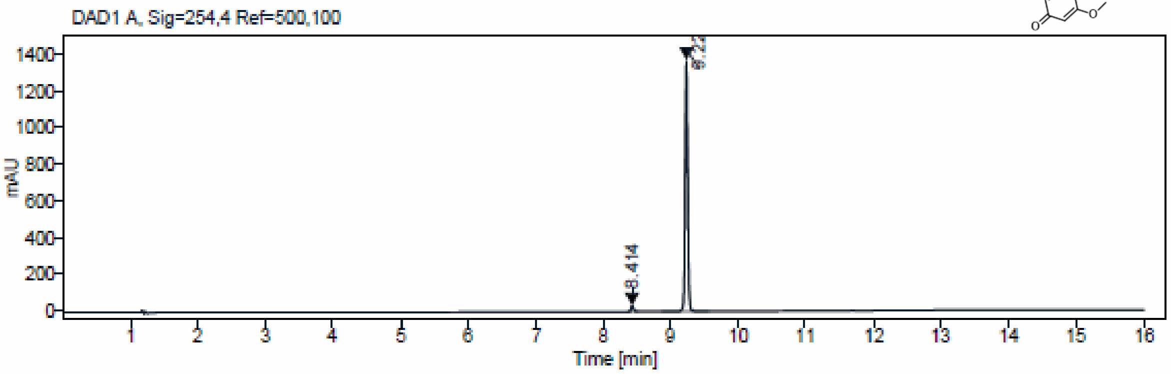

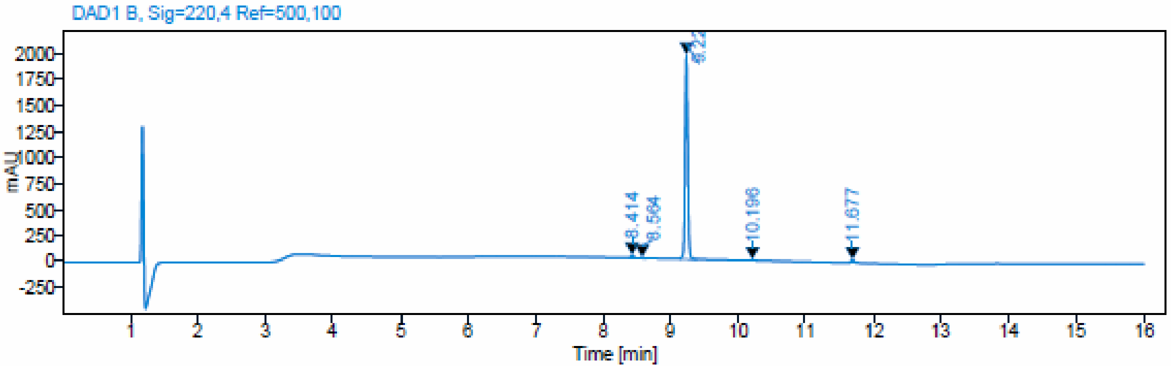

Signal: DAD1 A, Sig=254,4 Ref=500,100

RT [min]	Type	Width [min]	Area	Height	Area%	Name
8.414	BB	0.0428	89.7533	32.8858	2.3188	
9.220	BB	0.0422	3780.9734	1371.8975	97.6812	
		Sum	3870.7267			

Signal: DAD1 B, Sig=220,4 Ref=500,100

RT [min]	Type	Width [min]	Area	Height	Area%	Name
8.414	MM	0.0497	75.0976	25.1673	1.2634	
8.564	MM	0.0654	32.2221	8.2078	0.5421	
9.220	MM	0.0484	5703.5005	1963.6620	95.9524	
10.196	MM	0.0704	46.4136	10.9858	0.7808	
11.677	MM	0.0492	86.8624	29.4368	1.4613	
		Sum	5944.0963			

Compound 17 (PG-4c)

@ 254 and 220 nM - as described in the general chemistry method for analytical HPLC, method A.

**Instrument:**	Analytical HPLC
**Acq. method:**	MSE_PURITY_CHECK_20MICROL.M
**Injection date:**	2022-06-13 14:15:26+01:00
**Sample name:**	MSEC35_prep_20uL
**Injection volume:**	20.000

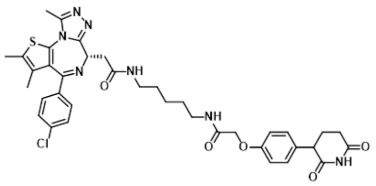

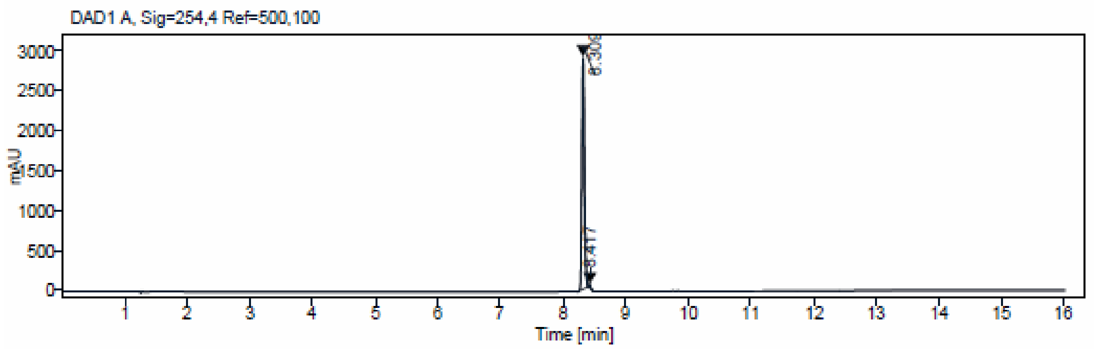

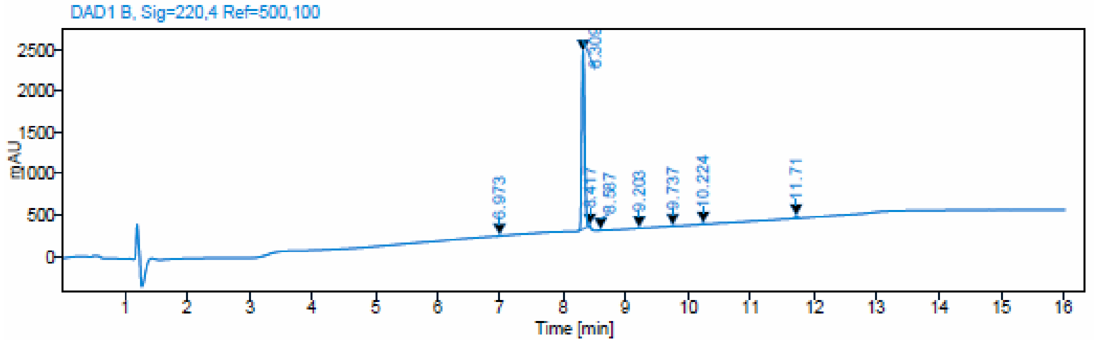

Signal: DAD1 A, Sig=254,4 Ref=500,100

RT [min]	Type	Width [min]	Area	Height	Area%	Name
8.309	MM	0.0443	7745.5537	2913.8542	98.6803	
8.417	MM	0.0347	103.5846	49.8084	1.3197	
		Sum	7849.1383			

Signal: DAD1 B, Sig=220,4 Ref=500,100

RT [min]	Type	Width [min]	Area	Height	Area%	Name
6.973	MM	0.0515	35.3813	11.4478	0.5088	
8.309	MM	0.0516	6623.8887	2138.5952	95.2611	
8.417	MM	0.0329	62.4398	31.6141	0.8980	
8.587	MM	0.0509	24.1721	7.9183	0.3476	
9.203	MM	0.0641	27.6296	7.1811	0.3974	
9.737	MM	0.0583	26.6375	7.6096	0.3831	
10.224	MM	0.0864	48.1091	9.2751	0.6919	
11.710	MM	0.0538	105.1443	32.5566	1.5121	
		Sum	6953.4023			

Compound 18 (Bn-CRBN)

@ 254 and 220 nM - as described in the general chemistry method for analytical HPLC, method A.

**Instrument:**	Analytical HPLC	
**Acq. method:**	MSE_PURITY_CHECK_10MICRO.M	
**Injection date:**	2022-08-18 10:52:01+01:00	
**Sample name:**	MSEC92_2_prep	
**Injection volume:**	10.000	

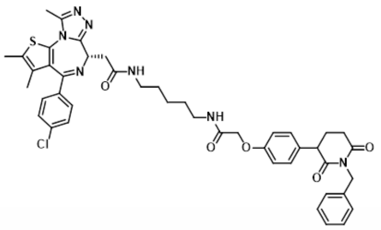

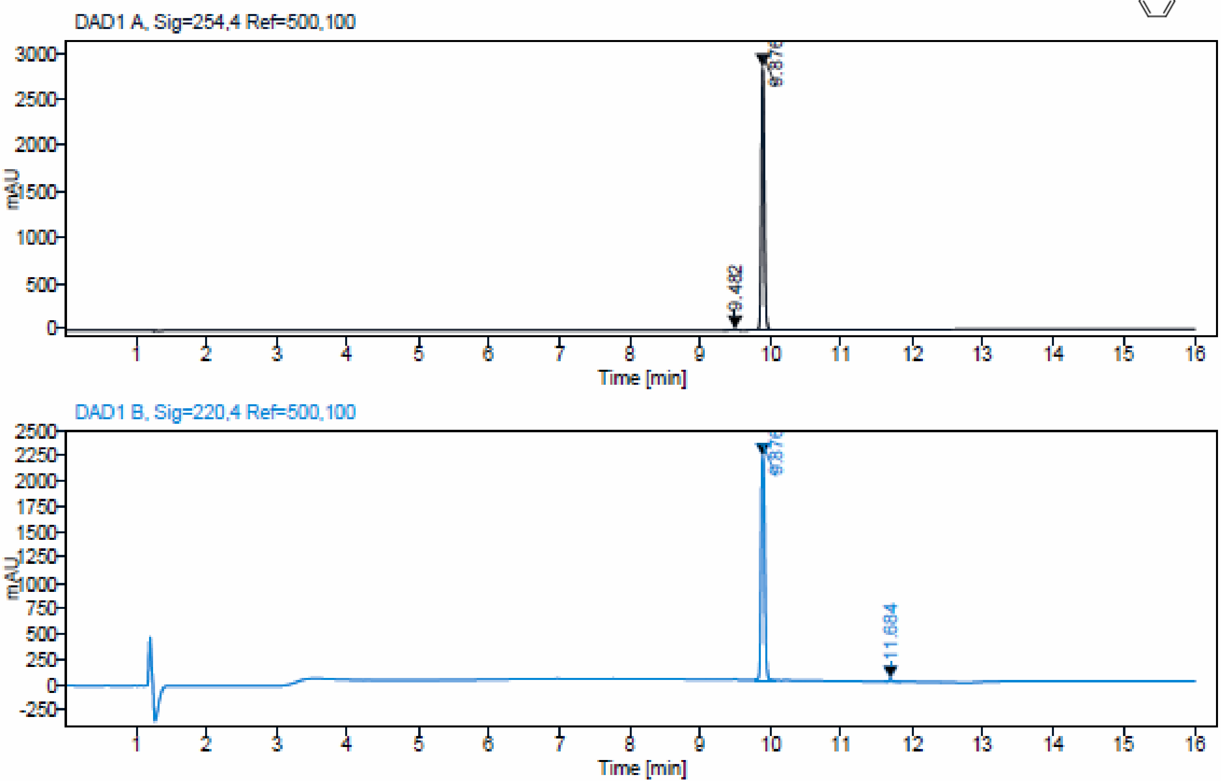

Signal: DAD1 A, Sig=254,4 Ref=500,100

RT [min]	Type	Width [min]	Area	Height	Area%	Name
9.482	VB R	0.0476	31.4086	10.0120	0.3784	
9.876	BB	0.0448	8267.9043	2855.7415	99.6216	
		Sum	8299.3129			

Signal: DAD1 B, Sig=220,4 Ref=500,100

RT [min]	Type	Width [min]	Area	Height	Area%	Name
9.876	MM	0.0605	7957.5581	2193.2231	99.0019	
11.684	MM	0.0566	80.2223	23.6146	0.9981	
		Sum	8037.7804			

6. Abbreviations

Bn, benzyl; CRBN, cereblon; DIPEA, *N*,*N*-diisopropylethylamine; DIAD, diisopropyl azodicarboxylate; DMAP, 4-dimethylaminopyridine; DMA, dimethylacetamide; DMEM, Dulbecco's Modified Eagle Medium; DMF, dimethylformamide; DMSO, dimethyl sulfoxide; EtOAc, ethyl acetate; EtOH, ethanol; FBS, foetal bovine serum; HAP-TAC, hypoxia-activated PROTAC; HATU, 1-[bis(dimethylamino)methylene]-1*H*-1,2,3-triazolo[4,5-*b*]pyridinium 3-oxide hexafluorophosphate; HPLC, high-performance liquid chromatography; HRMS, high resolution mass spectrometry; IQ, indolequinone; IR, infrared; (+)-JQ1, (*S*)-(+)-*tert*-butyl 2-(4-(4-chlorophenyl)-2,3,9-trimethyl-6*H*-thieno[3,2-*f*][1,2,4]triazolo[4,3-*a*][1,4]diazepin-6-yl)acetate; KOAc, potassium acetate; LCMS, liquid chromatography–mass spectrometry; LRMS, low-resolution mass spectrometry; MTT, (3-(4,5-dimethylthiazol-2-yl)-2,5-diphenyl tetrazolium bromide); NBS, *N*-bromosuccinimide; NB, 4-nitrobenzyl; NI, 2-nitroimidazol; $Pd(OAc)_2$, palladium(II) acetate; PE, petroleum ether; PG, phenyl glutarimide; PROTAC, PROteolysis TArgeting Chimera; PS, polymer-supported; PyBOP, (benzotriazol-1-yloxy)tri(1-pyrrolidinyl)phosphonium hexafluorophosphate; TBAI, tetra-*n*-butylammonium iodide; TBS-T, Tris-buffered saline + Tween20; TFA, trifluoroacetic acid; THF, tetrahydrofuran; TLC, thin layer chromatography; UV/vis, ultraviolet–visible; VHL, von Hippel- Lindau.

7. References

(1) O'Connor, L. J.; Cazares-Körner, C.; Saha, J.; Evans, C. N. G.; Stratford, M. R. L.; Hammond, E. M.; Conway, S. J. Design, Synthesis and Evaluation of Molecularly Targeted Hypoxia-Activated Prodrugs. *Nat. Protoc.* **2016**, *11*, 781–794.

(2) Wallabregue, A. L. D.; Bolland, H.; Faulkner, S.; Hammond, E. M.; Conway, S. J. Two Color Imaging of Different Hypoxia Levels in Cancer Cells. *J. Am. Chem. Soc.* **2023**, *145*, 2572–2583.

(3) Han, X.; Wang, C.; Qin, C.; Xiang, W.; Fernandez-Salas, E.; Yang, C. Y.; Wang, M.; Zhao, L.; Xu, T.; Chinnaswamy, K.; Delproposto, J.; Stuckey, J.; Wang, S. Discovery of ARD-69 as a Highly Potent Proteolysis Targeting Chimera (PROTAC) Degrader of Androgen Receptor (AR) for the Treatment of Prostate Cancer. *J. Med. Chem.* **2019**, *62*, 941–964.

(4) Shi, S.; Du, Y.; Zou, Y.; Niu, J.; Cai, Z.; Wang, X.; Qiu, F.; Ding, Y.; Yang, G.; Wu, Y.; Xu, Y.; Zhu, Q. Rational Design for Nitroreductase (NTR)-Responsive Proteolysis Targeting Chimeras (PROTACs) Selectively Targeting Tumor Tissues. *J. Med. Chem.* **2022**, *65*, 5057–5071.

(5) Min, J.; Mayasundari, A.; Keramatnia, F.; Jonchere, B.; Yang, S. W.; Jarusiewicz, J.; Actis, M.; Das, S.; Young, B.; Slavish, J.; Yang, L.; Li, Y.; Fu, X.; Garrett, S. H.; Yun, M. K.; Li, Z.; Nithianantham, S.; Chai, S.; Chen, T.; Shelat, A.; Lee, R. E.; Nishiguchi, G.; White, S. W.; Roussel, M. F.; Potts, P. R.; Fischer, M.; Rankovic, Z. Phenyl-Glutarimides: Alternative Cereblon Binders for the Design of PROTACs. *Angew. Chem. Int. Ed. Engl.* **2021**, *60*, 26663–26670.

(6) Karnthaler-Benbakka, C.; Groza, D.; Koblmüller, B.; Terenzi, A.; Holste, K.; Haider, M.; Baier, D.; Berger, W.; Heffeter, P.; Kowol, C. R.; Keppler, B. K. Targeting a Targeted Drug: An Approach Toward Hypoxia-Activatable Tyrosine Kinase Inhibitor Prodrugs. *ChemMedChem* **2016**, *11*, 2410–2421.

(7) Huang, B.; Desai, A.; Tang, S.; Thomas, T. P.; Baker, J. R. The Synthesis of a c(RGDyK) Targeted SN38 Prodrug with an Indolequinone Structure for Bioreductive Drug Release. *Org. Lett.* **2010**, *12*, 1384–1387.

(8) Torisu, K.; Kobayashi, K.; Iwahashi, M.; Egashira, H.; Nakai, Y.; Okada, Y.; Nanbu, F.; Ohuchida, S.; Nakai, H.; Toda, M. Development of a Prostaglandin D2 Receptor Antagonist: Discovery of a New Chemical Lead. *Eur. J. Med. Chem.* **2005**, *40*, 505–519.

(9) Huang, B.; Tang, S.; Desai, A.; Cheng, X. min; Kotlyar, A.; Spek, A. Van Der; Thomas, T. P.; Baker, J. R. Human Plasma-Mediated Hypoxic Activation of Indolequinone-Based Naloxone pro-Drugs. *Bioorg. Med. Chem. Lett.* **2009**, *19*, 5016–5020.

(10) Naylor, M. A.; Jaffar, M.; Nolan, J.; Stephens, M. A.; Butler, S.; Patel, K. B.; Everett, S. A.; Adams, G. E.; Stratford, I. J. 2-Cyclopropylindoloquinones and Their Analogues as Bioreductively Activated Antitumor Agents: Structure-Activity in Vitro and Efficacy in Vivo. *J. Med. Chem.* **1997**, *40*, 2335–2346.

(11) Cotterill, A. S.; Moody, C. J.; Mortimer, R. J.; Norton, C. L.; O'Sullivan, N.; Stephens, M. A.; Stradiotto, N. R.; Swann, E.; Stratford, I. J. Cyclopropamitosenes, Novel Bioreductive Anticancer Agents. Synthesis, Electrochemistry, and Biological Activity of 7-Substituted Cyclopropamitosenes and Related Indolequinones. *J. Med. Chem.* **1994**, *37*, 3834–3843.

(12) Sharma, K.; Iyer, A.; Sengupta, K.; Chakrapani, H. INDQ/NO, a Bioreductively Activated Nitric Oxide Prodrug. Org. Lett. 2013, 15, 2636–2639.

(13) Yamazaki, Y.; Kohno, K.; Yasui, H.; Kiso, Y.; Akamatsu, M.; Nicholson, B.; Deyanat-Yazdi, G.; Neuteboom, S.; Potts, B.; Lloyd, G. K.; Hayashi, Y. Tubulin Photoaffinity Labeling with Biotin-Tagged Derivatives of Potent Diketopiperazine Antimicrotubule Agents. *ChemBioChem* **2008**, *9*, 3074–3081.

(14) Steinebach, C.; Voell, S. A.; Vu, L. P.; Bricelj, A.; Sosič, I.; Schnakenburg, G.; Gütschow, M. A Facile Synthesis of Ligands for the von Hippel–Lindau E3 Ligase. *Synthesis* **2020**, *52*, 2521–2527.

(15) Galdeano, C.; Gadd, M. S.; Soares, P.; Scaffidi, S.; Van Molle, I.; Birced, I.; Hewitt, S.; Dias, D. M.; Ciulli, A. Structure-Guided Design and Optimization of Small Molecules Targeting the Protein-Protein Interaction between the von Hippel-Lindau (VHL) E3 Ubiquitin Ligase and the Hypoxia Inducible Factor (HIF) Alpha Subunit with in Vitro Nanomolar Affinities. *J. Med. Chem.* **2014**, *57*, 8657–8663.

(16) Zengerle, M.; Chan, K. H.; Ciulli, A. Selective Small Molecule Induced Degradation of the BET Bromodomain Protein BRD4. *ACS Chem. Biol.* **2015**, *10*, 1770–1777.

(17) Muller, G. W.; Stirling, D. I.; Chen, R. S.-C. Methods for the Treatment of Cachexia and Graft v. Host Disease. 2005. US7629360B2.

(18) Steinebach, C.; Lindner, S.; Udeshi, N. D.; Mani, D. C.; Kehm, H.; Köpff, S.; Carr, S. A.; Gütschow, M.; Krönke, J. Homo-PROTACs for the Chemical Knockdown of Cereblon. *ACS Chem. Biol.* **2018**, *13*, 2771–2782.

(19) Ge, C.; Muller, G. W.; Chen, R.; Saindane, M. T. Processes for the Preparation of 4-Amino-2-(2,6-Dioxopiperidin-3-Yl)Isoindoline-1,3-Dione Compounds. 2016. US20160297791A1.
